# Peptide-Alkoxyamine Drugs: An Innovative Approach to Fight Schistosomiasis: “Digging Their Graves with Their Forks”

**DOI:** 10.3390/pathogens13060482

**Published:** 2024-06-06

**Authors:** Ange W. Embo-Ibouanga, Michel Nguyen, Jean-Patrick Joly, Mathilde Coustets, Jean-Michel Augereau, Lucie Paloque, Nicolas Vanthuyne, Raphaël Bikanga, Anne Robert, Françoise Benoit-Vical, Gérard Audran, Philippe Mellet, Jérôme Boissier, Sylvain R. A. Marque

**Affiliations:** 1 Aix-Marseille University, CNRS, UMR 7273, Case 551, Avenue Escadrille Normandie-Niemen, CEDEX 20, 13397 Marseille, France; eangewilfrid@gmail.com (A.W.E.-I.); jean-patrick.joly@univ-amu.fr (J.-P.J.); 2 Laboratoire de Chimie de Coordination (LCC-CNRS) and, New Antimalarial Molecules and Pharmacological Approaches (MAAP), Inserm ERL 1289, Université de Toulouse, CNRS, 31077 Toulouse, France; michel.nguyen@lcc-toulouse.fr (M.N.); mathilde.coustets@lcc-toulouse.fr (M.C.); jean-michel.augereau@lcc-toulouse.fr (J.-M.A.); lucie.paloque@lcc-toulouse.fr (L.P.); anne.robert@lcc-toulouse.fr (A.R.); francoise.vical@inserm.fr (F.B.-V.); 3 Institut de Pharmacologie et de Biologie Structurale (IPBS), Université de Toulouse, CNRS, Université Toulouse III—Paul Sabatier (UPS), 31077 Toulouse, France; 4 Aix-Marseille University, CNRS, Centrale Marseille ISM2, Case 531, Avenue Escadrille Normandie-Niemen, CEDEX 20, 13397 Marseille, France; 5 Université des Sciences et Techniques de Masuku, LASNSOM, Franceville BP 901, Gabon; brbikanga@hotmail.fr; 6 Magnetic Resonance of Biological Systems, UMR 5536 CNRS-University of Bordeaux, 146 rue Leo Saignat, CEDEX, 33076 Bordeaux, France; 7 INSERM, 146 rue Leo Saignat, CEDEX, 33076 Bordeaux, France; 8 IHPE, CNRS, Ifremer, University Perpignan Via Domitia, 66860 Perpignan, France

**Keywords:** schistosoma, alkoxyamines, prodrug, new concept

## Abstract

The expansion of drug resistant parasites sheds a serious concern on several neglected parasitic diseases. Our recent results on cancer led us to envision the use of peptide-alkoxyamines as a highly selective and efficient new drug against schistosome adult worms, the etiological agents of schistosomiasis. Indeed, the peptide tag of the hybrid compounds can be hydrolyzed by worm’s digestive enzymes to afford a highly labile alkoxyamine which homolyzes spontaneously and instantaneously into radicals—which are then used as a drug against Schistosome adult parasites. This approach is nicely summarized as *digging their graves with their forks*. Several hybrid peptide-alkoxyamines were prepared and clearly showed an activity: two of the tested compounds kill 50% of the parasites in two hours at a concentration of 100 µg/mL. Importantly, the peptide and alkoxyamine fragments that are unable to generate alkyl radicals display no activity. This strong evidence validates the proposed mechanism: a specific activation of the prodrugs by the parasite proteases leading to parasite death through in situ alkyl radical generation.

## 1. Introduction

**Scheme 1:**
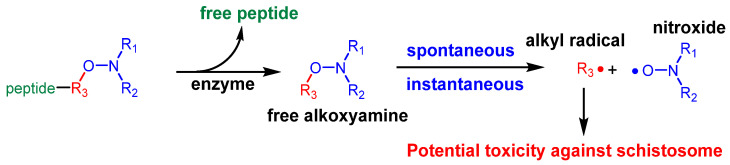
Scheme for the concept: digging their graves with their forks.

Schistosomiasis (or bilharziasis) is a major parasitic disease in terms of mortality and morbidity, as almost 200 million people are infected, and at least 200,000 deaths per year are associated with this disease. This disease is caused by worms of the *Schistosoma* genus present in freshwater bodies of subtropical regions [1]. Consequences of chronic infection include fibrosis and calcification of the urinary tract, renal failure or bladder cancer and acute hepatitis, liver and intestine fibrosis, and portal hypertension. On children, it may cause poor growth and learning difficulty. Up to now, a single drug against schistosomiasis, praziquantel (PZQ), has been used for more than 50 years, and many cases of resistance or decrease in sensitivity have been evidenced [2,3]. Moreover, praziquantel demonstrates activity against adult worms (after 5–7 weeks post-infection) but lacks efficacy against early human stages (i.e., schistosomula). Because individuals living in endemic areas are continually re-infected, the strategy for reducing the worm burden in each person necessitates the development of novel drugs capable of efficiently targeting all life stages of schistosomes in humans. Consequently, research on new drugs able to circumvent the praziquantel™ (PZQ) resistance and to kill all life stages of the parasite is an absolute priority. Schistosome parasites ingest host red blood cells and digest hemoglobin to obtain the amino-acids they need for the synthesis of their own proteins [4]. A molecule targeting the parasite’s mandatory hemoglobin digestion activity could therefore be efficient at several stages of its development [5]. Recently, we proposed an approach based on the use of a mandatory enzymatic activity of cancer cells to deliver specifically to the intended target a drug known to have a non-selective activity [6]. Here we propose to test such an approach on *Schistosoma mansoni* adult worms, an approach which we could not resist the pun of naming *digging their graves with their forks* (Scheme 1). Indeed, any parasite has specific enzymatic activities different from the host’s. Then, hybrid peptide-alkoxyamines are specifically hydrolyzed by enzymes (here proteases, due to their peptide tags) into free peptides and free alkoxyamines. The latter homolyzed spontaneously and instantaneously at room temperature into nitroxyl (blue part) and alkyl (red part) radicals. The latter are potentially cytotoxic for their environment, leading to the death of the parasites, i.e., here schistosomes.

It is expected that the schistosomes’ hemoglobin-digesting proteases, which are not naturally present in human cells, are specific parasite enzymes responsible for the recycling of amino acids from the host’s hemoglobin. Moreover, it has been demonstrated that within the same protease family, such as cathepsin D, the differences in cleavage sites between schistosomes and humans are so great that it is possible to develop specific inhibitors targeting specifically the hemoglobin-ingesting parasite [7]. In schistosome, no serine protease has been identified in the gut of the parasite, suggesting that the proteases involved in the digestion are totally different from those of the vertebrate host. However, unlike *Plasmodium*’s, the schistosome’s hemoglobin digestion cascade has not been fully characterized. The hemoglobin digestion implies at least three classes of proteases present in the parasite gut lumen: cathepsin (B, C and L), metallo-aminopeptidases and asparaginyl endopeptidases [8]. Considering that both parasites produce the same waste product (i.e., hemozoin) from the same nutrient (i.e., hemoglobin), we hypothesized that there might be a convergence between *Plasmodium* and *Schistosoma* in the enzymes involved in the proteolytic activity. Indeed, the proteolytic cleavage profiles of crude hemoglobin at pH around 5 are almost identical when obtained by *Plasmodium* digestive plasmepsins or by schistosome digestive cathepsins [9]. This convergence is probably due mostly to the structural resistance to proteolysis of blood proteins, which allows them to be less sensitive to microbial infections. Thus, proteolysis can only occur on a few less structured sites as exemplified by the main proteolysis site 33–34 at the C-terminal extremity of a helix. Substrate specificity profiles for the digestive plasmepsins of *Plasmodium* are much better characterized than for schistosome digestive enzymes [10]. Thus, we selected the short peptide Phe-Val-Phe extracted from the substrate specificity study of *Plasmodium* plasmepsin II [11] because the synthesis did not require further protection/deprotection steps.

**Figure 1:**
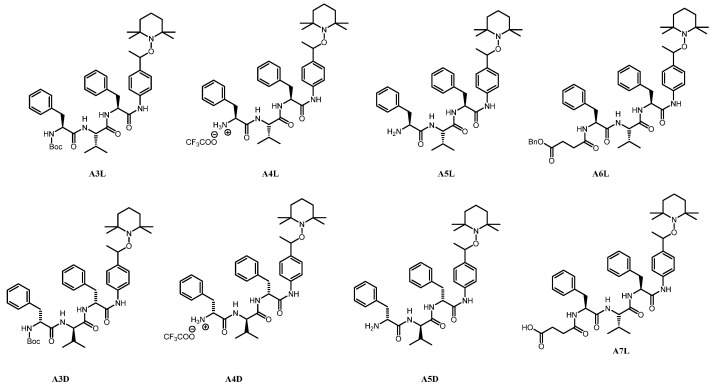
Peptide-alkoxyamines investigated as anti-schistosomal drugs. L and D are for the configuration of the residues on the peptides as given by Fischer’s rules.

Therefore, drawing on our experience with alkoxyamines’ efficiency against tumors [6] and *Plasmodium* [12], alkoxyamines **A3L**–**A7L** (Figure 1) carrying the peptide sequence L-Phe-Val-Phe-OH are tested (More details are provided in [12]). For comparison, the series **D** made of unnatural peptides is also investigated (Figure 1).

## 2. Materials and Methods

*Preparation of alkoxyamines and reference samples.* All reagents including anhydrous solvents used for the chemical synthesis were purchased from commercially available sources such as Merck, TCI, and Fluorochem, and were used without further purification. All air and/or water-sensitive reactions were carried out under an argon atmosphere with dry solvents using standard syringe-cannula/septa techniques. Routine monitoring of reactions was performed using Merck Silica gel 60 F_254_, aluminum-supported TLC plates; spots were visualized using UV light and ethanolic acidic para-anisaldehyde solution or ethanolic phosphomolybdic solution, followed by heating. Purifications by means of column chromatography were performed with Silica gel 60 (230–400 mesh). ^1^H, ^13^C, and ^19^F NMR spectra were obtained in CDCl_3_, MeOD (with residual H_2_O), D_2_O, DMSO-d_6_, or C_6_D_6_ solutions on by using Bruker AC400 (400 MHz) and Bruker AC300 (300 MHz) spectrometers. High-resolution mass spectra (HRMS) have been performed using a SYNAPT G2 HDMS (Waters) mass spectrometer equipped with a pneumatically assisted atmospheric pressure ionization (API) source. The sample was ionized in positive electrospray mode under the following conditions: electrospray voltage: 2.8 kV; port voltage: 20 V; Nebulizing gas flow rate (nitrogen): 100 L/h or in negative electrospray mode under the following conditions: electrospray voltage: −2.27 kV; port voltage: −20 V; Nebulizing gas flow rate (nitrogen): 100 L/h. The mass spectra (MS) were obtained with a time-of-flight (TOF) analyzer. The exact mass measurements were carried out in triplicate with external calibration. Homolysis constants were measured by EPR on an EMX Bruker spectrometer from 10^−4^ M samples in tertbutyl benzene or in a mixture of H_2_O-*n*PrOH (1:1; *v*:*v*).

**General ethyl chloroformate/triethylamine procedure GP1.** A solution of *N*-Boc-phenylalanine (1.3 eq) in THF (150 mL) is cooled to −15 °C under an argon atmosphere. Triethylamine (1.3 eq) is added, and then the ethyl chloroformate (1.3 eq). The reaction mixture is stirred at −15 °C for 1 h. Then, alkylaniline (1.0 eq) dissolved in THF (20 mL) is added and the reaction is stirred for 1 h at −15 °C, then at room temperature overnight. The solvent is removed in vacuo, and the residue is taken up in DCM. The organic layer is washed sequentially with 1 M HCl twice, saturated NaHCO_3_ solution twice and brine twice, dried over MgSO_4_, and evaporated under reduced pressure. The purification is performed by flash column chromatography (EtOAc/Petroleum ether).

**General Salen/MnCl_2_ alkoxyamine synthesis procedure GP2**. To a stirred solution of *N,N*’-bis(salicylidene)ethylene diamine (salen ligand, 0.25 eq.) in THF (10 mL) is added MnCl_2_ (0.25 eq) in an open flask. After 10 min of stirring at room temperature, a solution of TEMPO (1.0 eq) and 4-vinylanilido phenylalanine-Boc (1.2 eq) in THF (50 mL) is added, and then solid NaBH_4_ is added (4.0 eq) in four portions, every 10 min. The mixture is stirred between 24 h and 48 h at room temperature. At the end, the solution is dissolved in EtOAc (100 mL) in an Erlenmeyer and carefully quenched at 0 °C by the addition of 1 M HCl until the solution becomes colorless or slightly orange. Then, NaHCO_3_ is added up to neutral pH and the organic layer is washed with water (80 mL) and brine (80 mL). The organic layer is dried on MgSO_4_, filtered, and evaporated to dryness. The purification is performed by flash column chromatography (EtOAc/Petroleum ether).

**General Boc deprotection procedure GP3.** The Boc-residue (1.0 eq) is dissolved in DCM (45 mL, V_DCM_ = 4V_TFA_) and TFA (10.0 eq) is added under air. The reaction mixture is stirred at room temperature. Once the substrate is fully consumed, toluene (25 mL) is added, and the solvents are removed *in vacuo*. The co-evaporation is repeated twice. The purification by flash column chromatography (EtOAc/Petroleum ether) is performed.

**General DCC/HOBt procedure GP4.** Diisopropylethylamine (1.0 eq) is added to a stirred solution of TFA salt (1.0 eq) under argon. The mixture is stirred for 10 min at room temperature, then Boc-dipeptide (1.1 eq) and HOBt (1.1 eq) are added and stirred until dissolved. The mixture is cooled to 0 °C, and DCC or EDCI (1.1 eq) is added. The mixture is stirred overnight at room temperature. The mixture is filtered with cold DCM, and washed with 1 M HCl, NaHCO_3_ (saturated solution), and brine. The purification is performed by flash column chromatography (EtOAc/Petroleum ether).

**General TFA removing procedure GP5.** 1 M NaOH solution (6 mL) is added to a stirred solution of TFA salt (1.0 eq) under air. The mixture is stirred for 10 min at room temperature, the mixture becomes cloudy. Then, a few drops of MeOH are added and the aqueous phase is extracted three times with DCM (5 mL). The organic layer is dried with MgSO_4_, filtered, and the solvent is evaporated to dryness.

**General hydrogenation procedure GP6.** To a solution of the benzylated compound (1.0 eq) in MeOH (20 mL) is added Pd/(C) (10% weight). Then, the atmosphere in the flask is replaced by the H_2_ atmosphere and stirred. Once the substrate is fully consumed, the mixture is filtrated through a pad of celite, rinsed with MeOH, and the solvent is evaporated to dryness.

*tert-butyl (S)-(1-oxo-3-phenyl-1-((4-vinylphenyl)amino)propan-2-yl)carbamate* **P3L**. By using the general ethyl chloroformate/triethylamine procedure on N-Boc-L-phenylalanine **P1L** (12.44 g, 46.910 mmol, 1.3 eq) and 4-vinylaniline (4.30 g, 36.085 mmol, 1.0 eq) afforded **P3L** as a white solid (11.89 g, yield: 90%).

*Benzyl (tert-butoxycarbonyl)-L-phenylalanyl-L-valinate* **P4L.** Using GP4 with HCl.H_2_N-L-Val-Bn **P2L** (10.00 g, 41.03 mmol, 1.0 eq) and BocHN-L-Phe-OH **P1L** (11.97 g, 45.13 mmol, 1.1 eq). The purification by column chromatography (EtOAc/Petroleum ether) of crude **P4L** affords a white solid (18.07 g, 97%) as reported in the literature [13].

*(Tert-butoxycarbonyl)-L-phenylalanyl-L-valine* **P5L.** Using GP6 with **P4L** (12.00 g, 26.417 mmol, 1.0 eq) affords **P5L** as a white solid (9.62 g, quantitative yield) as described in the literature [14].

*Benzyl (tert-butoxycarbonyl)-L-phenylalanyl-L-valyl-L-phenylalaninate* **P6L.** Using GP4 with L-Phenylalanine benzyl ester hydrochloride (2.000 g, 6.854 mmol, 1.0 eq) and **P5L**. The purification by flash column chromatography (DCM/MeOH, 98: 2, *v*/*v*) of the crude **P6L** affords a white solid (3.00 g, yield 73%).

*(Tert-butoxycarbonyl)-L-phenylalanyl-L-valyl-L-phenylalanine* **P7L.** Using GP6 with **P6L** (0.53 g, 0.881 mmol, 1.0 eq) and Pd/(C) (0.053 g, 10% weight) and the solvent is evaporated to dryness to afford the pure **P7L** as white solid (0.44 g, 90%) as reported in the literature [15].

*(S)-1-(((S)-1-(((S)-1-(benzyloxy)-1-oxo-3-phenylpropan-2-yl)amino)-3-methyl-1-oxobutan-2-yl)amino)-1-oxo-3-phenylpropan-2-aminium trifluoroacetate* **P8L**. By usingGP3 with **P6L** (0.60 g, 0.997 mmol, 1.0 eq) afforded **P8L** as a white solid (0.49 g, yield 79%).

*Benzyl 4-(((S)-1-(((S)-1-(((S)-1-(benzyloxy)-1-oxo-3-phenylpropan-2-yl)amino)-3-methyl-1-oxobutan-2-yl)amino)-1-oxo-3-phenylpropan-2-yl)amino)-4-oxobutanoate* **P9L**. Using GP4 (DCC is replaced by EDCI.HCl) with **P8L** (0.20 g, 0.391 mmol, 1.0 eq) affords crude **P9L**. The purification by column chromatography (DCM/MeOH) affords **P9L** as a white solid (0.17 g, yield 62%).

*4-(((S)-1-(((S)-1-(((S)-1-carboxy-2-phenylethyl)amino)-3-methyl-1-oxobutan-2-yl)amino)-1-oxo-3-phenylpropan-2-yl)amino)-4-oxobutanoic acid* **P10L**. Using GP6 with **P9L** (0.12 g, 0.173 mmol, 1.0 eq) affords **P10L** as a white solid (0.08 g, yield 94%).

*(S)-1-(((S)-1-(((S)-1-carboxy-2-phenylethyl)amino)-3-methyl-1-oxobutan-2-yl)amino)-1-oxo-3-phenylpropan-2-aminium trifluoroacetate* **P11L**. Using GP6 with **P8L** (2.50 g, 4.063 mmol, 1.0 eq) affords **P11L** as a white solid (2.00 g, yield 94%).

*(4-(benzyloxy)-4-oxobutanoyl)-L-phenylalanyl-L-valyl-L-phenylalanine* **P12L.** 4-(benzyloxy)-4-oxobutanoic acid (0.087 g, 0.418 mmol, 1.1 eq) and HOBt (0.056 g, 0.418 mmol, 1.1 eq) are dissolved in DCM, under argon, and stirred until clearance. The mixture is cooled to 0 °C and EDCI.HCl (0.080 g, 0.418 mmol, 1.1 eq) was added. The mixture is stirred for 1 h at 0 °C. Then, a solution of diisopropylethylamine (0.06 mL, 0.380 mmol, 1.0 eq) and peptide TFA salt **P11L** (0.20 g, 0.380 mmol, 1.0 eq) in DCM is added dropwise. The mixture is stirred for 30 min at 0 °C and overnight at room temperature. The mixture is washed with 1 M HCl and brine. The organic layer was dried with MgSO_4_, filtered, and the solvent was evaporated to dryness. The purification by column chromatography (DCM/MeOH) affords **P12L** as a white solid (0.108 g, 47%).

*Benzyl (((9H-fluoren-9-yl)methoxy)carbonyl)-L-phenylalanyl-L-valinate* **P14L.** Using GP4 with HCl.H_2_N-L-Val-Bn **P2L** (2.00 g, 8.205 mmol, 1.0 eq), Fmoc-L-Phe-OH **P13L** (3.49 g, 9.025 mmol, 1.1 eq) and HOBt (1.22 g, 9.025 mmol, 1.1 eq) affords **P14L**. The purification by column chromatography (EtOAc/Petroleum ether) affords **P14L** as a white solid (4.06 g, 86%).

*(((9H-fluoren-9-yl)methoxy)carbonyl)-L-phenylalanyl-L-valine* **P15L.** Using GP6 with **P14L** (3.80 g, 6.589 mmol, 1.0 eq) affords **P15L** as a white solid (3.15 g, yield 98%). Already described in the literature [16].

*Benzyl (((9H-fluoren-9-yl)methoxy)carbonyl)-L-phenylalanyl-L-valyl-L-phenylalaninate* **P16L.** Using GP4 with Fmoc-L-Phe-L-Val-OH **P15L** (0.16 mg, 0.328 mmol, 1.0 eq) and HCl.H_2_N-L-Phe-Bn (0.11 mg, 0.360 mmol, 1.1 eq) affords crude **P16L**. The purification by column chromatography (DCM/MeOH) yields **P16L** as a white solid (0.17 g, 71%).

***L-phenylalanyl-L-valyl-L-phenylalanine* P18L.** To a solution of the amino acid Fmoc-L-Phe-L-Val-L-Phe-OBn **P16L** (1.300 g; 1.795 mmol; 1.0 eq.) in DCM (15 mL) at 0 °C under Argon was added in one fraction DBU (0.29 mL; 1.975 mmol; 1.1 eq.). After 3 h, the mixture was directly loaded on a silica gel column chromatography (DCM/MeOH) to obtain the primary amine. Then, GP6 is applied to this product (0.25 g, 0.498 mmol, 1.0 eq) affording **P18L** as a white solid (0.11 g, yield 52%).

*tert-butyl (S)-(1-oxo-3-phenyl-1-((4-vinylphenyl)amino)propan-2-yl)carbamate* **P19L**. Using GP1, N-Boc-phenylalanine **P1L** (11.38 g, 42.911 mmol, 1.3 eq), triethylamine (5.79 mL, 42.911 mmol, 1.3 eq) and ethyl chloroformate (3.81 mL, 42.911 mmol, 1.3 eq) provides **P19L** as a white solid (10.72 g, 88%).

*(S)-1-((4-ethylphenyl)amino)-1-oxo-3-phenylpropan-2-aminium trifluoroacetate* **P20L:** Using GP3 with the Boc-residue **P19L** (5.00 g, 13.58 mmol, 1.0 eq) and TFA (11.65 mL, 152.23 mmol, 10.0 eq) afforded **P20L** as a white solid (5.20 g, quant).

*tert-butyl ((S)-1-(((S)-1-(((S)-1-((4-ethylphenyl)amino)-1-oxo-3-phenylpropan-2-yl)amino)-3-methyl-1-oxobutan-2-yl)amino)-1-oxo-3-phenylpropan-2-yl)carbamate* **P21L**. By using GP4 with **P20L** (2.00 g, 5.233 mmol, 1.0 eq), the Purification by flash column chromatography (EtOAc/Petroleum ether) of the crude product afforded a white solid (3.00 g, yield 93%). Product containing less than 8% residual DCU.

*(S)-1-(((S)-1-(((S)-1-((4-ethylphenyl)amino)-1-oxo-3-phenylpropan-2-yl)amino)-3-methyl-1-oxobutan-2-yl)amino)-1-oxo-3-phenylpropan-2-aminium trifluoroacetate* **P22L**. By GP3 with **P21L** (3.20 g, 5.208 mmol, 1.0 eq) afforded **P22L** as a white solid (1.96 g, yield 60%).

*Benzyl 4-(((S)-1-(((S)-1-(((S)-1-((4-ethylphenyl)amino)-1-oxo-3-phenylpropan-2-yl)amino)-3-methyl-1-oxobutan-2-yl)amino)-1-oxo-3-phenylpropan-2-yl)amino)-4-oxobutanoate* **P23L**. Using GP4 (DCC is replaced by EDCI.HCl) with **P22L** (1.83 g, 2.917 mmol, 1.0 eq) affords **P23L** as a white solid (1.05 g, yield 50%).

*4-(((S)-1-(((S)-1-(((S)-1-carboxy-2-phenylethyl)amino)-3-methyl-1-oxobutan-2-yl)amino)-1-oxo-3-phenylpropan-2-yl)amino)-4-oxobutanoic acid* **P24L**. Using GP6 with **P23L** (0.12 g, 0.173 mmol, 1.0 eq) affords **P24L** as a white solid (0.08 g, yield 94%).

*(S)-2-((S)-2-amino-3-phenylpropanamido)-N-((S)-1-((4-ethylphenyl)amino)-1-oxo-3-phenylpropan-2-yl)-3-methylbutanamide* **P25L**. Using GP5 with **P22L** (0.33 g, 0.525 mmol, 1.0 eq) affords **P25L** as a white solid (0.25 g, yield 92%).

*tert-butyl (R)-(1-oxo-3-phenyl-1-((4-vinylphenyl)amino)propan-2-yl)carbamate* **P3D**. By using GP1 with N-Boc-D-phenylalanine **P1D** (12.44 g, 46.910 mmol, 1.3 eq) and 4-vinylaniline (4.30 g, 36.085 mmol, 1.0 eq) afforded **P3D** as a white solid (11.23 g, yield 85%).

*Benzyl (tert-butoxycarbonyl)-D-phenylalanyl-D-valinate* **P4D.** Using GP4 with Boc-D-Phenylalanine **P1D** (7.69 g, 28.99 mmol, 1.1 eq) and D-valine benzyl ester p-toluenesulfonate **P2D** (10.00 g, 26.35 mmol, 1.0 eq). The purification by column chromatography (EtOAc/Petroleum ether) of crude P4D affords a white solid (10.30 g, 86%).

*(Tert-butoxycarbonyl)-D-phenylalanyl-D-valine* **P5D.** Using GP6 with **P4D** (9.00 g, 19.808 mmol, 1.0 eq) affords **P4D** as a white solid (6.49 g, yield 90%) as described in the literature [17].

*Benzyl (tert-butoxycarbonyl)-D-phenylalanyl-D-valyl-D-phenylalaninate* **P6D.** Using GP4 with L-Phenylalanine benzyl ester hydrochloride **P5D** (3.00 g, 8.237 mmol, 1.0 eq) affords crude **P6D**. The purification by flash column chromatography (DCM/MeOH, 98:2, *v*/*v*) yields **P6D** as a white solid (4.22 g, yield 84%).

*(Tert-butoxycarbonyl)-D-phenylalanyl-D-valyl-D-phenylalanine* **P7D.** Using GP6 with **P6D** (2.50 g, 4.157 mmol, 1.0 eq) affords **P7D** as a white solid (2.12 g, quantitative yield).

*(R)-1-(((R)-1-(((R)-1-(benzyloxy)-1-oxo-3-phenylpropan-2-yl)amino)-3-methyl-1-oxobutan-2-yl)amino)-1-oxo-3-phenylpropan-2-aminium trifluoroacetate* **P8D**. By using GP3 with P6D (0.60 g, 0.997 mmol, 1.0 eq) afforded **P8D** as a crude white solid (0.31 g, yield 50%).

*(R)-1-(((R)-1-(((R)-1-carboxy-2-phenylethyl)amino)-3-methyl-1-oxobutan-2-yl)amino)-1-oxo-3-phenylpropan-2-aminium trifluoroacetate* **P11D**. Using GP6 with **P8D** (0.24 g, 0.377 mmol, 1.0 eq) affords **P11D** as a white solid (0.17 g, yield 85%).

*Tert-butyl ((R)-1-(((R)-1-(((R)-1-((4-ethylphenyl)amino)-1-oxo-3-phenylpropan-2-yl)amino)-3-methyl-1-oxobutan-2-yl)amino)-1-oxo-3-phenylpropan-2-yl)carbamate* **P21D**. A solution of the *N*-Boc-D-Phe-D-Val-D-Phe-OH P7D (2.14 g, 4.185 mmol, 1.2 eq) in THF (50 mL) is cooled to −15 °C under argon atmosphere. Triethylamine (0.56 mL, 4.185 mmol, 1.2 eq) is added, and then ethyl chloroformate (0.37 mL, 4.185 mmol, 1.2 eq). The reaction mixture is stirred at −15 °C. After 1 h, 4-ethylaniline (0.43 g, 3.487 mmol, 1.0 eq) dissolved in THF (15 mL) was added and the reaction was allowed to stir for 1 h at −15 °C, then at room temperature overnight. The solvent is removed in vacuo, and the residue is taken up in DCM. The organic layer is washed sequentially with 1 M HCl twice, saturated NaHCO_3_ solution twice and brine twice, dried over MgSO_4_, and evaporated under reduced pressure. The purification by flash column chromatography (EtOAc/Petroleum ether) affords **P21D** as a white solid (0.47 mg, 22%).

*(R)-1-(((R)-1-(((R)-1-((4-ethylphenyl)amino)-1-oxo-3-phenylpropan-2-yl)amino)-3-methyl-1-oxobutan-2-yl)amino)-1-oxo-3-phenylpropan-2-aminium trifluoroacetate* **P22D**. By using GP3 with **P21D** (0.27 g, 0.439 mmol, 1.0 eq) afforded **P22D** as a white solid; (0.25 g, yield 92%).

*tert-butyl ((2S)-1-oxo-3-phenyl-1-((4-(1-((2,2,6,6-tetramethylpiperidin-1-yl)oxy)ethyl)phenyl)amino)propan-2-yl)carbamate* **A1L**. Using GP2 with salen ligand (0.64 g, 2.389 mmol, 0.25 eq), MnCl_2_ (0.47 g, 2.389 mmol, 0.25 eq), TEMPO (1.49 g, 9.56 mmol, 1.0 eq) and 4-vinylanilido phenylalanine-Boc **P3L** (4.20 g, 11.469 mmol, 1.2 eq) afforded **A1L** as a white solid (2.70 mg, 54%, mixture of diastereomers, 1:1).

*(2S)-1-oxo-3-phenyl-1-((4-(1-((2,2,6,6-tetramethylpiperidin-1-yl)oxy)ethyl)phenyl)amino)propan-2-aminium trifluoroacetate* **A2L**. By using GP3 with **A1L** (0.86 g, 1.643 mmol, 1.0 eq) afforded **A2L** as a white solid (0.71 g, 81%). The product was directly used in the coupling reaction without characterization.

*tert-butyl ((2S)-1-(((2S)-3-methyl-1-oxo-1-(((2S)-1-oxo-3-phenyl-1-((4-(1-((2,2,6,6-tetramethylpiperidin-1-yl)oxy)ethyl)phenyl)amino)propan-2-yl)amino)butan-2-yl)amino)-1-oxo-3-phenylpropan-2-yl)carbamate* **A3L**. Using GP4 with **A2L** (0.72 g, 1.336 mmol, 1.0 eq) and DCC was replaced by EDCI.HCl to prevent the formation of DCU (a by-product that is difficult to remove entirely) affords crude **A3L**. The Purification by flash column chromatography (EtOAc/Petroleum ether) of the crude product afforded a white solid (0.76 g, yield 74%).

*(2S)-1-(((2S)-3-methyl-1-oxo-1-(((2S)-1-oxo-3-phenyl-1-((4-(1-((2,2,6,6-tetramethylpiperidin-1-yl)oxy)ethyl)phenyl)amino)propan-2-yl)amino)butan-2-yl)amino)-1-oxo-3-phenylpropan-2-aminium trifluoroacetate* **A4L**. By using GP3 with **A3L** (0.15 g, 0.195 mmol, 1.0 eq) afforded **A4L** as a white solid (0.091 g, yield 60%).

*(2S)-2-((S)-2-amino-3-phenylpropanamido)-3-methyl-N-((2S)-1-oxo-3-phenyl-1-((4-(1-((2,2,6,6-tetramethylpiperidin-1-yl)oxy)ethyl)phenyl)amino)propan-2-yl)butanamide* **A5L**. Using GP5 with **A4L** (0.045 mg, 0.057 mmol, 1.0 eq) affords **A5L** as a white solid (0.033 g, 87%).

*Benzyl 4-(((2S)-1-(((2S)-3-methyl-1-oxo-1-(((2S)-1-oxo-3-phenyl-1-((4-(1-((2,2,6,6-tetramethylpiperidin-1-yl)oxy)ethyl)phenyl)amino)propan-2-yl)amino)butan-2-yl)amino)-1-oxo-3-phenylpropan-2-yl)amino)-4-oxobutanoate* **A6L**. Using GP4 (DCC is replaced by EDCI.HCl) with **A4L** (1.48 g, 1.887 mmol, 1.0 eq) and 4-(benzyloxy)-4-oxobutanoic acid (0.43 g, 2.075 mmol, 1.1 eq) affords crude **A6L**. The purification by column chromatography (DCM/MeOH) affords **A6L** as a white solid (1.20 g, 73%).

*4-(((2S)-1-(((2S)-3-methyl-1-oxo-1-(((2S)-1-oxo-3-phenyl-1-((4-(1-((2,2,6,6-tetramethylpiperidin-1-yl)oxy)ethyl)phenyl)amino)propan-2-yl)amino)butan-2-yl)amino)-1-oxo-3-phenylpropan-2-yl)amino)-4-oxobutanoic acid* **A7L**. Using GP6 with **A6L** (0.521 g, 0.605 mmol, 1.0 eq) affords **A7L** as a white solid (0.422 g, yield 90%).

*tert-butyl ((2R)-1-oxo-3-phenyl-1-((4-(1-((2,2,6,6-tetramethylpiperidin-1-yl)oxy)ethyl)phenyl)amino)propan-2-yl)carbamate* **A1D**. By using GP2 with salen ligand (0.64 g, 2.39 mmol, 0.25 eq.), MnCl2 (0.47 g, 2.389 mmol, 0.25 eq), TEMPO (1.49 g, 9.56 mmol, 1.0 eq) and 4-vinylaniline D-phenylalanine-Boc **P3D** (3.50 g, 9.557 mmol, 1.2 eq) afforded **A1D** as a white solid (2.23 g, 58%, mixture of diastereomers, 1:1).

*(2R)-1-oxo-3-phenyl-1-((4-(1-((2,2,6,6-tetramethylpiperidin-1-yl)oxy)ethyl)phenyl)amino)propan-2-aminium trifluoroacetate* **A2D**. Using GP3 with **A1D** (1.50 g, 2.866 mmol, 1.0 eq) is afforded **A2D** as a white solid (1.33 g, yield: 86%). The product was used in coupling reaction without characterization.

*tert-butyl ((2R)-1-(((2R)-3-methyl-1-oxo-1-(((2R)-1-oxo-3-phenyl-1-((4-(1-((2,2,6,6-tetramethylpiperidin-1-yl)oxy)ethyl)phenyl)amino)propan-2-yl)amino)butan-2-yl)amino)-1-oxo-3-phenylpropan-2-yl)carbamate* **A3D**. Using GP4 with **A2D** (1.333 g, 2.481 mmol, 1.0 eq) affords crude **A3D**. The Purification by flash column chromatography (EtOAc/Petroleum ether) yields **A3D** as a white solid (1.300 g, yield 68%, mixture of diastereomers, 1:1).

*(2R)-1-(((2R)-3-methyl-1-oxo-1-(((2R)-1-oxo-3-phenyl-1-((4-(1-((2,2,6,6-tetramethylpiperidin-1-yl)oxy)ethyl)phenyl)amino)propan-2-yl)amino)butan-2-yl)amino)-1-oxo-3-phenylpropan-2-aminium trifluoroacetate* **A4D**. Using GP3 with **A3D** (0.30 g, 0.389 mmol, 1.0 eq) is afforded **A4D** as a white solid (0.16 g, 52%).

*(2R)-2-((R)-2-amino-3-phenylpropanamido)-3-methyl-N-((2R)-1-oxo-3-phenyl-1-((4-(1-((2,2,6,6-tetramethylpiperidin-1-yl)oxy)ethyl)phenyl)amino)propan-2-yl)butanamide* **A5D**. Using GP5 with **A4D** (0.035 g, 0.052 mmol, 1.0 eq) affords **A5D** as a white solid (0.024 g, yield 80%).

*4-(Benzyloxy)-4-oxobutanoic acid***.** Succinic anhydride (8.00 g, 79.944 mmol, 1.0 eq) was dissolved in the mixture of anhydrous dichloromethane (10 mL) and Benzyl alcohol (12 mL). Triethylamine (3.2 mL, 23.983 mmol, 0.3 eq) and DMAP (9.76 g, 79.944 mmol, 1.0 eq) were added to this solution. The resulting clear solution was stirred at ambient temperature overnight. After which time, the mixture was dissolved in EtOAc (150 mL) and washed with a saturated solution of NaHCO_3_ (3 × 50 mL). The aqueous extracts were carefully acidified to pH 2 with concentrated HCl and then extracted with DCM (3 × 50 mL) and subsequently dried (Mg_2_SO_4_), filtered, and concentrated to afford the title compound (16.23 g, 97%) as a white solid with no further purification needed.

**Scheme 2:**
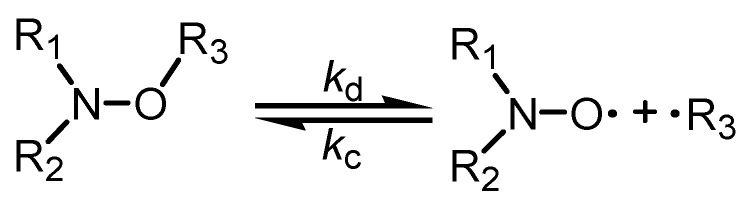
Dynamic Covalent Bond in alkoxyamines: *k*_d_ for the homolysis rate constant and *k*_c_ for the re-formation reaction.

*Kinetic experiments*. Kinetics of **A1**–**A7** (Scheme 2) for series L and D are performed by monitoring the growth of nitroxide by Electron Paramagnetic Resonance (X-band EMX Bruker machine) in *tert*-butylbenzene and a water/*n*-PrOH (for a good solubility of the alkoxyamine) as solvents and using O_2_ as alkyl radical scavenger to suppress the back reaction (*k*_c_ in Scheme 2) as already reported [18]. Homolysis rate constants *k*_d_ are given by Equation (1) ([nitroxide]_∞_ = [alkoxyamine]_0_ = 0.1 mM, see Table S1) and the subsequent activation energies *E*_a_ given by Equation (2).

(1) $$ln\left(\frac{{\left[nitroxide\right]}_{\infty }-{\left[nitroxide\right]}_{t}}{{\left[nitroxide\right]}_{\infty }}\right)=-k_{d}\cdot t,$$

(2) $$E_{a}=-RTln\left(\frac{k_{d}}{A}\right).$$

*Cytotoxicity experiments.*

A non-cancer line of Vero cells was used to determine the cytotoxicity of the compound against mammalian cells. They were evaluated at a final DMSO concentration of 0.5%. The culture medium was MEM (Dutscher, Bernolsheim, France) supplemented with 10% fetal bovine serum (Fisher Scientific, Illkirch, France), 1X non-essential amino acids (Fisher Scientific, Illkirch, France), 100 U/mL, 100 µg/mL penicillin/streptomycin (Fisher Scientific, Illkirch, France), and 2 mM L-glutamine (Fisher Scientific, Illkirch, France) at 37 °C in a humidified 5% CO_2_ atmosphere [19]. Vero cells (100 µL of 10^5^ cells/mL per well) were plated in 96-well plates for 24 h then treated with 100 µL of compound dilutions (in duplicate) during 48 h. All molecules were tested from 5 nM to 50 µM. Each well was then examined under the microscope to detect any precipitate formation before the supernatant was removed by flicking the plate. A volume of 100 µL of 0.5 mg/mL in MEM from a stock solution at 5 mg/mL of PBS-dissolved MTT (3-(4,5-dimethylthiazol-2-yl)-2,5-diphenyltetrazolium bromide, Sigma Aldrich/Merck, Darmstadt, Germany) was then added to each well [20].

After incubation for 1 h at 37 °C and 5% CO_2_ the supernatant was removed and 100 µL of DMSO were added. Plates were gently shaken to dissolve formazan crystals resulting from the MTT reduction by living cells and read at 570 nm with the VICTOR Nivo plate reader (Perkin Elmer, Waltham, MA, USA). CC_50_ values were determined using GraphPad Prism 7 software (San Diego, CA, USA).

*Experiments on the viability of Schistosoma mansoni adult worms*. In vitro tests were performed on *Schistosoma mansoni* NMRI strains. This parasite is lab maintained on *Biomphalaria glabrata* (BRE strain) as intermediate mollusc host and Golden Hamster (Janvier Labs, Le Genest-Saint-Isle, France) as definitive vertebrate host. Methods, for molluscs’ and hamsters’ infection and for parasite recovery, were previously described [21]. Adult worms were recovered by hepatic perfusion technique between 42 and 47 days post hamster exposition to parasite larvae. Worms were carefully collected and disposed in 12-well Falcon^®^ plate containing 2 mL of RPMI 1640 (supplemented with L-glutamine and Hepes 25 mM). The plates containing a minimum of 15 worms by well were then placed in an incubator chamber at 37 °C and 5% CO_2_. The worms sex ratio was almost balanced in each well. The drug was first dissolved in DMSO (Sigma-Aldrich) to give a 100 mg/mL mother solution. Then the stock solution was complemented with Tween 80 and RPMI to obtain the following final ratio dilution: RPMI 1640/Tween 80/DMSO and 1000/0.95/3.8, *v*/*v*/*v*.

All molecules were tested at a final concentration of 100 µg/mL and 10 µg/mL. A negative control consisted in adding the same RPMI 1640/Tween 80/DMSO solution, but without any drug and a positive control consisted in adding praziquantel [22]. All molecules and control were performed in duplicates of well. The worm viability was checked after 1, 2, 3, 4, 5, 6, 7, 8, 24, 48, and 72 h after adding the molecule. Parasites exhibiting no body contractions during a 30 s observation were considered as dead.

## 3. Results

### 3.1. Synthesis

**Scheme 3:**
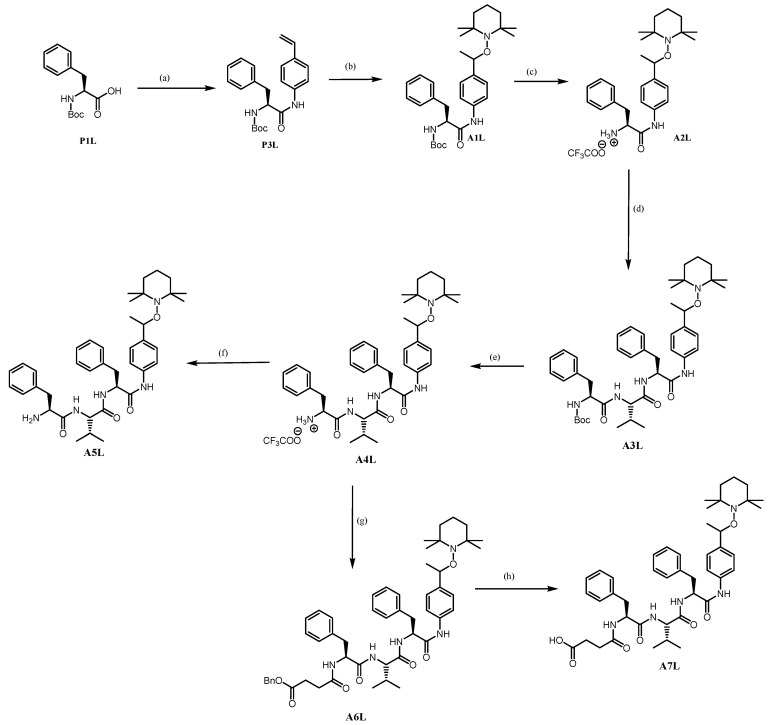
Preparation of alkoxyamines **A3L**–**A7L**: (a) ClCOOEt, Et_3_N, 4-vinylaniline, DCM, 0 °C to −15 °C to rt, overnight, 90%; (b) salen, MnCl_2_, TEMPO, O_2_ (air), THF, rt, overnight, 54%; (c) CF_3_COOH, DCM, rt, 2 h, 81%; (d) DIPEA, HOBT, Boc-L-Phe-L-Val-OH **P5L**, DCC, DCM, 0 °C to rt, overnight, 74%; (e) CF3COOH, DCM, rt, 4 h, 60%; (f) NaOH 1 M, DCM, 86%; (g) DIPEA, HOBT, HO-Suc-OBn, EDC.HCl, DCM, 0 °C to rt, overnight, 73%; (h) H2/Pd(C), DCM/MeOH (*v*:*v* 1:1), rt, 3 h, 90%.

**Scheme 4:**
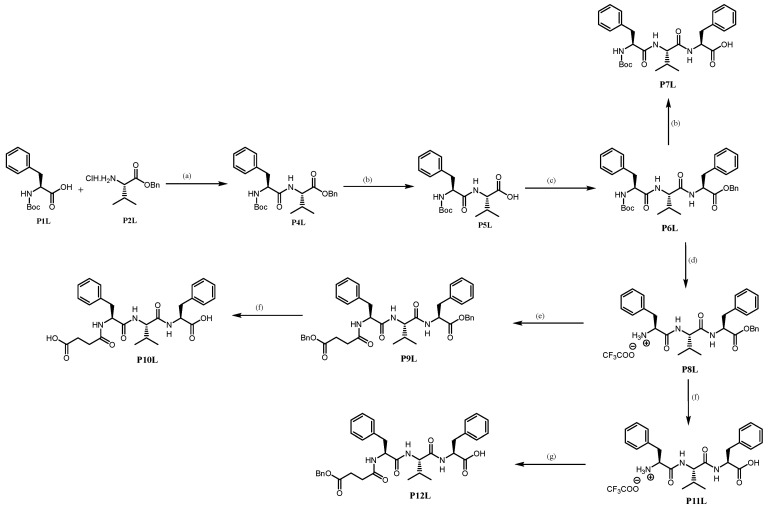
Preparation of the peptide tag: (a) DIPEA, HOBT, DCC, DCM, 0 °C to rt, overnight, 97%; (b) H_2_/Pd(C), DCM/MeOH (*v*/*v* 1:1), rt, 3 h, 94%; (c) DIPEA, HOBT, HCl.NH_2_-L-Phe-OBn, DCC, DCM, 0 °C to rt, overnight, 73%; (d) CF_3_COOH, DCM, rt, 4 h, 79%; (e) DIPEA, HOBT, HO-Suc-OBn, EDC.HCl, DCM, 0 °C to rt, overnight, 62%; (f) H_2_/Pd(C), MeOH, rt, 3 h, 94%; (g) HOBT, HO-Suc-OBn, EDC.HCl, DCM, 0 °C to rt, overnight, 47%.

**A3L**–**A7L**. Alkoxyamines **A3L**–**A7L** were prepared in 4 steps for **A3L** and a few more for **A4L**–**A7L** (Scheme 3) starting from the amino acid **P1L** transformed into amide **P3L**. The latter was coupled to TEMPO using the conventional Mn/salen procedure (see experimental section and references [23,24]) to afford alkoxyamine **A1L** which was hydrolyzed as salt **A2L**. Then, it was coupled to dipeptide **P5L** (Scheme 4) affording peptide-alkoxyamine **A3L**. The latter was deprotected as salt **A4L** which was, in turn, either neutralized as **A5L** or homologated in succinate and succinic derivatives **A6L** and **A7L**, respectively. The same procedure (see Supplementary Materials) was applied for the preparation of the unnatural series **A3D**–**A5D** in similar yields for each step.

**Scheme 5:**
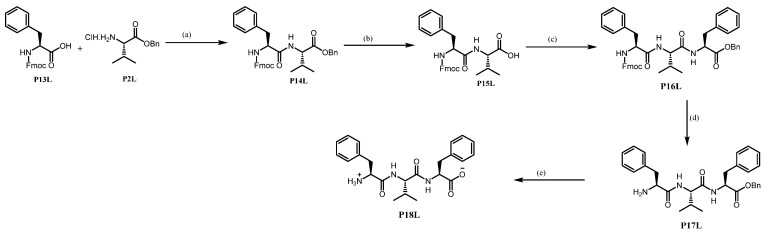
Preparation of the peptide tag H_2_N-L-Phe-L-Val-L-Phe-OH **P18L**: (a) DIPEA, HOBT, DCC, DCM, 0 °C to rt, overnight, 86%; (b) H_2_/Pd(C), DCM, rt, 4 h, 98%; (c) DIPEA, HOBT, HCl.NH_2_-L-Phe-OBn, DCC, DCM, 0 °C to rt, overnight, 71%; (d) DBU, DCM, rt, 4 h, 57%; (e) H_2_/Pd(C), DCM/MeOH (*v*:*v* 1:1), rt, 3 h, 52%.

**Scheme 6:**
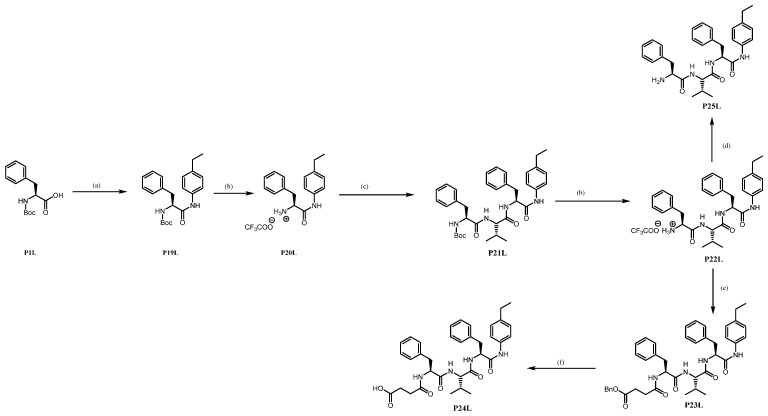
Preparation of models. (a) ClCOOEt, Et_3_N, 4-ethylaniline, DCM, 0 °C to −15 °C to rt, overnight, 88%; (b) CF_3_COOH, DCM, rt, 1 h, quantitative for **P20L** and 60% for **P22L**; (c) DIPEA, HOBT, Boc-L-Phe-L-Val-OH, DCC, DCM, 0 °C to rt, overnight, 93%; (d) NaOH 1 M, DCM, 92%; (e) HOBT, HO-Suc-OBn, EDC.HCl, DCM, 0 °C to rt, overnight, 50%; (f) H_2_/Pd(C), DCM/MeOH (*v*:*v* 1:1), rt, overnight, 96%.

To investigate the potential effect of peptides or peptide analogues potentially observed during the decomposition of **A3L**–**A7L**, derivatives **P6L**–**P12L**, **P17L**, **P18L**, and **P21L**–**P25L** were prepared as depicted in Scheme 4, Scheme 5 and Scheme 6. The same procedure (see Supplementary Materials) was applied for the preparation of the unnatural series **D** in similar yields for each step.

### 3.2. Kinetic Analysis

All homolysis rate constants *k*_d_ are collected in Table 1. Activation energies *E*_a_ (given by Equation (2)) are around 130 kJ/mol as expected [25] for *para*-substituted amino-group-aryl-TEMPO-based alkoxyamines meaning that alkoxyamines are stable under our experimental conditions (*t*_1/2_ > 170 day at 37 °C). As expected, *E*_a_ does not depend on the configuration of the peptide chain. Moreover, no difference in *E*_a_ is observed between diastereoisomers.

### 3.3. Cytotoxicity Activity

**Scheme 7:**
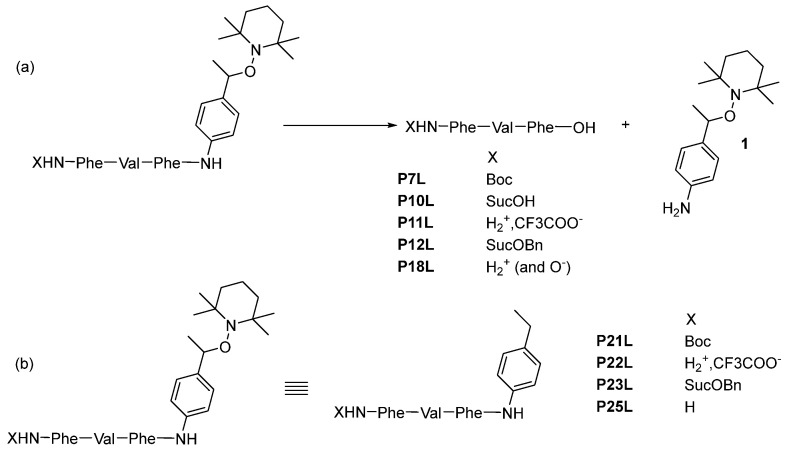
Enzymatic activity affording potentially toxic peptides (**a**) and molecules mimicking alkoxyamines (**b**).

Due to technical problems, it was not possible to perform cytotoxicity in the same concentrations as those used for toxicity experiments on schistosomes. Nevertheless, the cytotoxicity (CC_50_ > 50 μM) obtained on VERO cells after 48 h of treatment and reported for *Plasmodium* [12] shows that these molecules are rather inactive in mammalian cells [26]. Then, the approach developed in Scheme 1 may have involved different parts of the alkoxyamines in the inhibition processes both for toxicity and cytotoxicity. Hence, activity might be due to (a) the activity proposed in Scheme 1; (b) the toxicity of the released peptide (Scheme 7a); or (c) a non-radical activity of alkoxyamines which is mimicked with structures displayed in Scheme 7b. To address items (b) and (c), peptides **P7L**, **P10L**–**P12L**, and **P18L** and **P21L**–**P25L** were then prepared, respectively, as described in Scheme 4, Scheme 5 and Scheme 6.

Interestingly, all models from the **L** series, whether peptides alone or hybrid compounds, are non-cytotoxic (cytotoxicity concentration CC_50_ > 50 μM) [12]. Importantly, peptide-alkoxyamine hybrids in the natural series **L** are stable in our experimental conditions and not cytotoxic at 50 μM, confirming their specificity on schistosome. However, peptides **P8L** and **P9L**, which display the benzylated end-carboxy function, show an unexpected toxicity contrary to **P6L** (Table S1). The latter is tentatively ascribed to an inhibition effect due to a binding constant enhanced by the presence of the benzyl group on the carboxy-terminal function.

Peptide-D models **P6D**, **P8D**, **P21D**, and **P22D** are unexpectedly toxic (Table S1) whereas only **P6D** and **P22D** are also cytotoxic (Table S1). This activity is tentatively ascribed to an inhibition effect due to the wrong D configuration. Alkoxyamines **A4D**, and **A5D** (Table S1), which have been developed because eucaryote cells are not able to hydrolyze peptides of D-series, are surprisingly both cytotoxic for VERO cells and toxic to schistosomes. We tentatively ascribed these unexpected results either to the inhibition of the D-configuration of peptides (*vide supra*) or to the activity of bacteria in the digestive tube of schistosomes. Recent studies have evidenced the presence of bacteria interacting with schistosomes which could lead to the development of new control strategies targeting microbiomes [27]. However, at this stage, no better reasons can be provided and these alkoxyamines and peptides are not further discussed as they are not the focus of this article.

### 3.4. Schistosomicidal Activity

By analogy with plasmepsins of *Plasmodium,* which is also a hemoglobin-ingesting parasite, the peptide sequence Boc-Phe-Val-Phe can be considered as a potential substrate for the schistosome’s digestive enzymes. **A3L**–**A7L** should be hydrolyzed by schistosome and should exhibit activities, while they should not display any activity in mammalian cells because they do not have any chymotrypsin-like secreted activity. Table 2 shows that **A4L** and **A5L** are the most efficient molecules, **A3L** is inactive, and **A6L** and **A7L** are less active. The inactivity of **A3L** can be explained by its poor solubility. In the context of the schistosome digestive tube at pH 5.5, **A6L** is not protonated until the Boc group is enzymatically removed and **A7L** is less protonated than at pH 7, thus also poorly soluble**. A4L** and **A5L** however are fully protonated at pH 5.5, thus more soluble.

As mentioned above, peptide models are not cytotoxic for VERO cells. Surprisingly, peptide **P10L** displays some activity towards schistosomes which cannot be understood at this stage of our investigations. However, the cytotoxicity of **P10L** impacts only the discussion concerning **AL7**. On the other hand, and as expected, peptide models **P21L**–**P25L** carrying the phenylethyl moiety display no activity (closest non-reactive models of alkoxyamines **A3L**–**A7L,** that cannot produce alkyl radicals, see Scheme 7b and Table 2), which suggests that non-activated alkoxyamine are likely to be non-cytotoxic. Importantly, all alkoxyamines are thermally stable under our experimental conditions (see Table 1) meaning that the activities observed are likely due to their activation by schistosome enzymes.

None of the peptide-alkoxyamine hybrids displays any activity at concentrations of 10 μg/mL.

## 4. Discussion

As mentioned above, peptide models are not cytotoxic for VERO cells. Then, matching the activities of peptide-alkoxyamine hybrids with the activities of peptides **P11L** and **P22L** for **A4L**, **P18L** and **P25L** for **A5L**, and **P23L** for **A6L** shows that all these peptides display no activity. Therefore, the activity observed is due to the alkoxyamine moiety.

The lower activity of **A6L** and the non-activity of **A3L** highlight the importance of the substituent at the *N*-terminal position since, namely, a Boc-group cancels the activity (see **A3L** in Table 2), Benzylated succinyl groups lower the activity, and a succinyl moiety affords a peptide **10L** displaying some activity as in **A7L**. Nevertheless, **A6L** is able to kill schistosomes by releasing alkyl radicals as expected.

In sharp contrast to **A6L**, alkoxyamines **A4L** and **A5L** display a higher activity which is similar, i.e., the time to kill 50% of parasites is 2 h for **A4L** and **A5L**, vs. 24 h for **A6L** (Table 2).

**Scheme 8:**
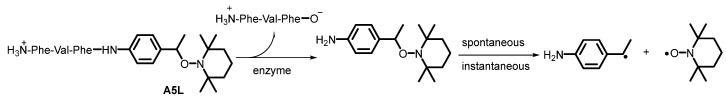
Scheme for the activation of alkoxyamine **A5L** by enzyme.

Importantly, the free-amino-peptide-based alkoxyamine **A5L** and the TFE-salt-peptide-based alkoxyamine **A4L** display the same structure in water solution, i.e., the protonated amino-terminal group, and as expected both alkoxyamines display the same activity (Table 2) (For technical reasons, it was not possible to record the same after 8 h for A4L. Moreover, the discussion of the expected reactivity is spoiled by the unexpected activity of P8L). Therefore, these results highlight the need for positively charged NH_2_-terminal groups (Table 2) to observe the activity displayed in Scheme 1, which makes sense at pH 5.5. Consequently, alkoxyamines **A4L**–**A6L** nicely support the proposed strategy, as displayed in Scheme 1 and Scheme 8.

Peptide-alkoxyamine hybrid activity targets the parasite’s mandatory digestion cascade. This strategy has already been used with other molecules such as trioxaquines, which have shown both in vitro and in vivo activities [28]. Our strategy considered that since there was a convergence with *Plasmodium* in the degradation chain from hemoglobin to hemozoin production, there could be a convergence in the actors involved in this proteolysis. This strategy proved effective, as our molecules are effective against schistosomes even though the plasmepsins are not present in their genome. This does not prevent convergent activity from a different family of proteases from occurring. Indeed, in a study comparing the genomic structures of the aspartic proteinase family genes, including the cathepsin D gene of schistosome, it was shown that there were exons that were common to both plasmepsins I, II, and IV from *Plasmodium falciparum* and cathepsin D from schistosome. Both plasmepsins I, II, and IV and cathepsin D have been evidenced to be major actors in hemoglobin degradation in *Plasmodium* [10] and schistosome [29], respectively. In vitro, the parasite’s digestive tract probably contains only a few globules, which could lead to low protease secretion in the digestive tract and hence slow activation of the alkoxyamine prodrugs. We would expect better activity by using parasites fed with red blood cells in in vitro or in in vivo experiments.

## 5. Conclusions

Although the activity of **A5L** and **A4L** (time to kill 50% = 2 h) is lower than that of praziquantel (time to kill 50% < 1 h), our results highlight the potential of our approach (Scheme 1 and Scheme 8), i.e., that the *Schistosoma* parasite could ***dig its grave with its fork***. That is, the mandatory feeding enzymatic activity of worms can be used to activate a prodrug able to release specifically in the desired target, due to the specific peptide, a non-selective and highly toxic free radical drug leading to the death of parasites. This original approach could lead to the development of antiparasitic compounds that are particularly safe for patients, with no or limited side effects.

## Figures and Tables

**Table 1:** Homolysis rate constants *k*_d_ for **3**–**9** (0.1 mM) of series L and D in *t*-BuPh and a water/*n*-PrOH mixture as solvents at a temperature *T* using *O*_2_ as alkyl radical scavenger, and the subsequent activation energies *E*_a_.

Alkoxyamine	*T* (°C) ^a^	*k*_d_ (10^−4^ s^−1^) ^a,b,c^	*E*_a_ (kJ/mol) ^b,d^	*T* (°C) ^e^	*k*_d_ (10^−4^ s^−1^) ^b,c,e^	*E_a_* (kJ/mol) ^b,d,e^
**A1L**	128.0	11.0	133.1	95.1	1.5	128.4
**A3L**	125.0	8.4	133.0	94.5	1.8	128.8
**A4L** *^f^*	^g^	^g^	^g^	94.6	1.0	129.3
**A5L**	130.0	18	132.1	94.0	1.3	128.3
**A6L**	125	11.0	132.1	94.8	1.1	129.1
**A7L**	126.5	15.0	131.6	94.3	9.5	129.4
**A1D**	122.5	9.6	132.2	93.5	1.0	129.0
**A3D**	119.8	9.6	132.2	92.6	0.45	131.0
**A4D** *^f^*	^g^	^g^	^g^	95.0	1.2	128.9
**A5D**	130	11.6	132.7	94.6	1.3	128.5

^a^ In *t*-BuPh ^b^ Value measured for a mixture of diastereosisomers. ^c^ Given by Equation (1). ^d^ Given by Equation (2) and an averaged frequency factor is used *A* = 2.4 10^14^ s^−1^, *R* = 8.314 J·K^−1^·mol^−1^, *k*_d_ and *T* are given in columns 2, 3, 5, and 6, see reference [17]. ^e^ In water/*n*-PrOH, *v*/*v*: 1:1. ^f^ Not soluble. ^g^ Not estimated.

**Table 2:** Alkoxyamines and peptides are provided to schistosome at 37 °C at massic concentration (100 μg/mL) *^a^*, time to reach the death of half of the population of worms, and time to kill all parasites.

	Time to Kill 50% of Parasites (h)	Time to Kill 100% of Parasites (h)
**A3L**	NA	NA
**A4L**	2	24
**A5L**	2	7
**A6L**	24	48
**A7L**	24	48
**P6L**	NA	NA
**P7L**	NA	NA
**P8L**	7	24
**P9L**	3	5
**P10L**	6	24
**P11L**	NA	NA
**P12L**	NA	NA
**P18L**	NA	NA
**P21L**	NA	NA
**P22L**	NA	NA
**P23L**	NA	NA
**P24L**	NA	NA
**P25L**	NA	NA
**PZQ**	1	1
**TFA**	7	24

*^a^.* NA: No Activity. Concentrations span from 100 to 200 μM for peptide-alkoxyamine hybrids from 200 to 400 μM for peptides, and 300 μM for PZQ.

## Data Availability

Data are contained within the article and Supplementary Materials.

## Supplementary Materials

The following supporting information can be downloaded at: https://www.mdpi.com/article/10.3390/pathogens13060482/s1, Table S1: Doses of alkoxyamines provided to *Schistosoma* at 37 °C, time to reach the death of half of the population of worms, time to kill all parasites, and their cytotoxicity on Vero cells (CC_50_ in μM). Table S2: Biological activity/ raw data. Analatical data, NMR and HRMS data of all chemiscals prepared in the article are provided in SM.
