# Correction: Embo-Ibouanga et al. Peptide-Alkoxyamine Drugs: An Innovative Approach to Fight Schistosomiasis: “Digging Their Graves with Their Forks”. *Pathogens* 2024, *13*, 482

**DOI:** 10.3390/pathogens14070669

**Published:** 2025-07-08

**Authors:** Ange W. Embo-Ibouanga, Michel Nguyen, Jean-Patrick Joly, Mathilde Coustets, Jean-Michel Augereau, Lucie Paloque, Nicolas Vanthuyne, Raphaël Bikanga, Anne Robert, Françoise Benoit-Vical, Gérard Audran, Philippe Mellet, Jérôme Boissier, Sylvain R. A. Marque

**Affiliations:** 1 Aix-Marseille University, CNRS, UMR 7273, Case 551, Avenue Escadrille Normandie-Niemen, CEDEX 20, 13397 Marseille, France; 2 Laboratoire de Chimie de Coordination (LCC-CNRS) and, New Antimalarial Molecules and Pharmacological Approaches (MAAP), Inserm ERL 1289, Université de Toulouse, CNRS, 31077 Toulouse, France; 3 Institut de Pharmacologie et de Biologie Structurale (IPBS), Université de Toulouse, CNRS, Université Toulouse III—Paul Sabatier (UPS), 31077 Toulouse, France; 4 Aix-Marseille University, CNRS, Centrale Marseille ISM2, Case 531, Avenue Escadrille Normandie-Niemen, CEDEX 20, 13397 Marseille, France; 5 Université des Sciences et Techniques de Masuku, LASNSOM, Franceville BP 901, Gabon; 6 Magnetic Resonance of Biological Systems, UMR 5536 CNRS-University of Bordeaux, 146 rue Leo Saignat, CEDEX, 33076 Bordeaux, France; 7 INSERM, 146 rue Leo Saignat, CEDEX, 33076 Bordeaux, France; 8 IHPE, CNRS, Ifremer, University Perpignan Via Domitia, 66860 Perpignan, France

There was an error in the original publication [1]. The conflict of interest statement was incomplete as the authors forgot to include this statement.

The updated statement is as follows: 

**Conflicts of Interest:** Jerome Boissier is the cofounder of ParaDev company that was subcontracted to carry out the antiparasitic tests on the Schistosome.

The authors state that the scientific conclusions are unaffected. This correction was approved by the Academic Editor. The original publication has also been updated.
