# Supplementary material for: Peptide-Alkoxyamine Drugs: An Innovative Approach to Fight Schistosomiasis: “Digging Their Graves with Their Forks”
Source: Pathogens. 2024 Jun 6;13(6):482. doi: 10.3390/pathogens13060482 (PMC11206678; doi:10.3390/pathogens13060482)
Supplement: Supplementary file 1 [file pathogens-13-00482-s001.zip › pathogens-2963059-supplementary.pdf]

Supplementary Materials

Peptide-Alkoxyamine Drugs: An Innovative Approach to Fight Schistosomiasis: "Digging Their Graves with Their Forks"

Ange W. Embo-Ibouanga [1], Michel Nguyen [2,3], Jean-Patrick Joly [1], Mathilde Coustets [2,3], Jean-Michel Augereau [2,3], Lucie Paloque [2,3], Nicolas Vanthuyne [4], Raphaël Bikanga [5], Anne Robert [2], Françoise Benoit-Vical [2,3], Gérard Audran [1,*], Philippe Mellet [6,7,*], Jérôme Boissier [8,*] and Sylvain R. A. Marque [1,*]

[1] Aix-Marseille University, CNRS, UMR 7273, Case 551, Avenue Escadrille Normandie-Niemen, CEDEX 20, 13397 Marseille, France; eangewilfrid@gmail.com (A.W.E.-I.); jean-patrick.joly@univ-amu.fr (J.-P.J.)

[2] Laboratoire de Chimie de Coordination (LCC-CNRS) and, New Antimalarial Molecules and Pharmacological Approaches (MAAP), Inserm ERL 1289, Université de Toulouse, CNRS, 31077 Toulouse, France; michel.nguyen@lcc-toulouse.fr (M.N.); mathilde.coustets@lcc-toulouse.fr (M.C.); jean-michel.augereau@lcc-toulouse.fr (J.-M.A.); lucie.paloque@lcc-toulouse.fr (L.P.); anne.robert@lcc-toulouse.fr (A.R.); francoise.vical@inserm.fr (F.B.-V.)

[3] Institut de Pharmacologie et de Biologie Structurale (IPBS), Université de Toulouse, CNRS, Université Toulouse III—Paul Sabatier (UPS), 31077 Toulouse, France

[4] Aix-Marseille University, CNRS, Centrale Marseille ISM2, Case 531, Avenue Escadrille Normandie-Niemen, CEDEX 20 ,13397 Marseille, France; nicolas.vanthuyne@univ-amu.fr

[5] Université des Sciences et Techniques de Masuku, LASNSOM, Franceville BP 901, Gabon; brbikanga@hotmail.fr

[6] Magnetic Resonance of Biological Systems, UMR 5536 CNRS-University of Bordeaux, 146 rue Leo Saignat, CEDEX, 33076 Bordeaux, France

[7] INSERM, 146 rue Leo Saignat, CEDEX, 33076 Bordeaux, France

[8] IHPE, CNRS, Ifremer, University Perpignan Via Domitia, 66860 Perpignan, France

* Correspondence: g.audran@univ-amu.fr (G.A.); philippe.mellet@rmsb.u-bordeaux.fr (P.M.); boissier@univ-perp.fr (J.B.); sylvain.marque@univ-amu.fr (S.R.A.M.)

Table of contents

(P3L) 3

(P4L) 4

(P5L) 5

(P6L) 6

(P7L) 8

(P8L) 9

4-(benzyloxy)-4-oxobutanoic acid 11

(P9L) 12

(P10L) 14

(P11L) 16

(P12L) 18

(P14L) 20

(P15L) 23

(P16L) 24
(P18L) 26
(P19L) 28
(P20L) 30
(P21L) 32
(P22L) 34
(P23L) 36
(P24L) 39
(P25L) 41
(P3D) 43
(P4D) 45
(P5D) 45
(P6D) 46
(P7D) 49
(P8D) 51
(P11D) 53
(P21D) 55
(P22D) 57
(A1L) 59
(A3L) 61
(A4L) 63
(A5L) 65
(A6L) 68
(A7L) 70
(A1D) 72
(A3D) 74
(A4D) 76
(A5D) 78
HPLC of hybrid-alkoxyamines 81
Kinetics and homolysis constants of alkoxyamines A3L 84
Table 1SI 85
Analitycal data 83

tert-butyl (S)-(1-oxo-3-phenyl-1-((4-vinylphenyl)amino)propan-2-yl)carbamate

(P3L)

^{1}H NMR (400 MHz, $CDCl_3$)

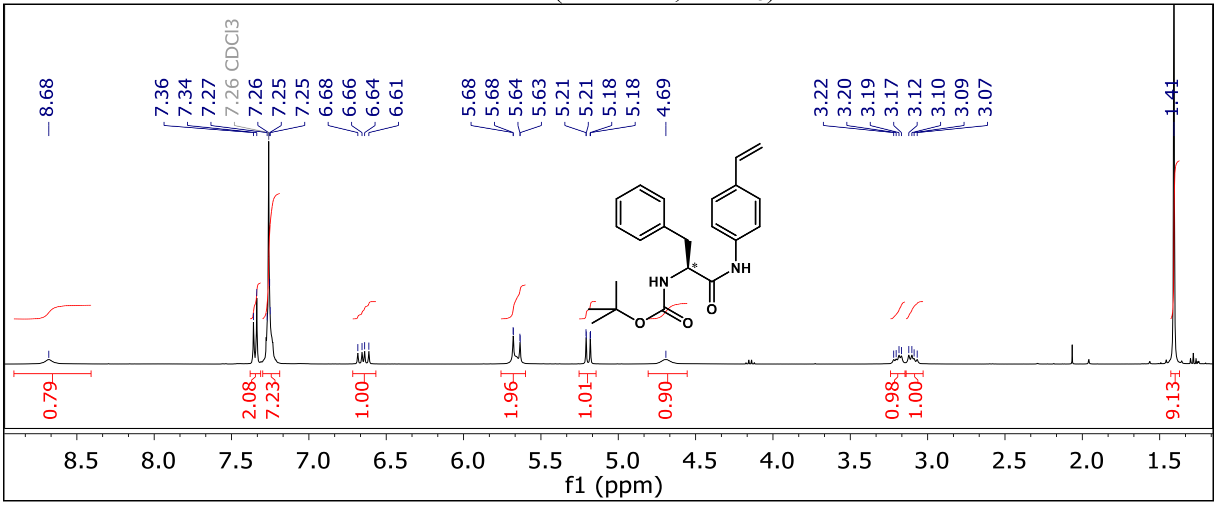

^{13}C NMR (101 MHz, $CDCl_3$)

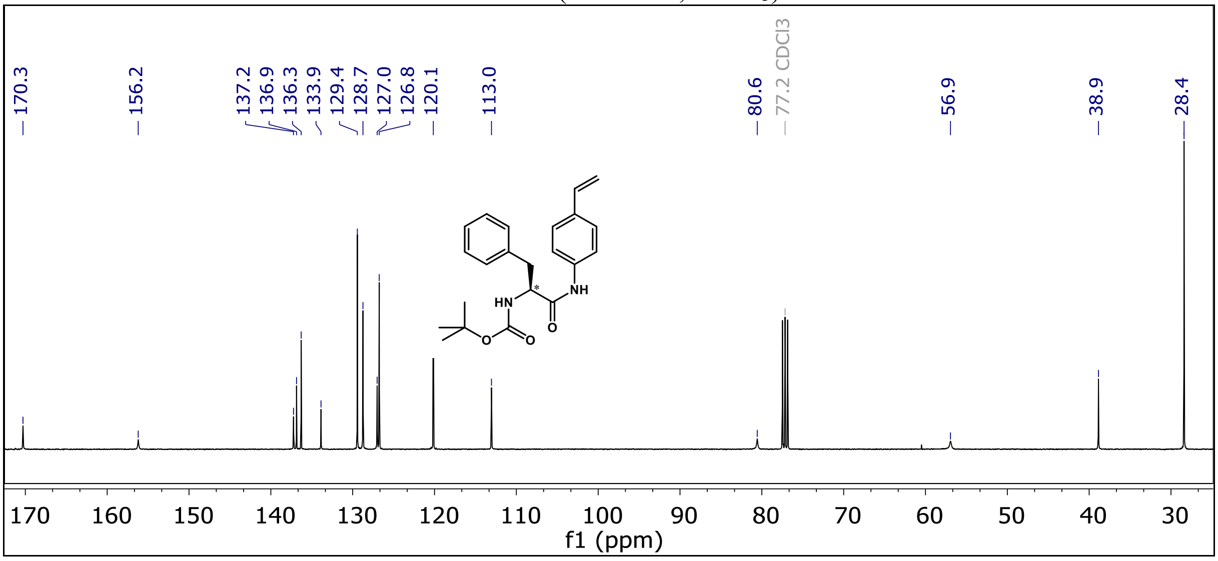

DEPT 135 (101 MHz, $CDCl_3$)

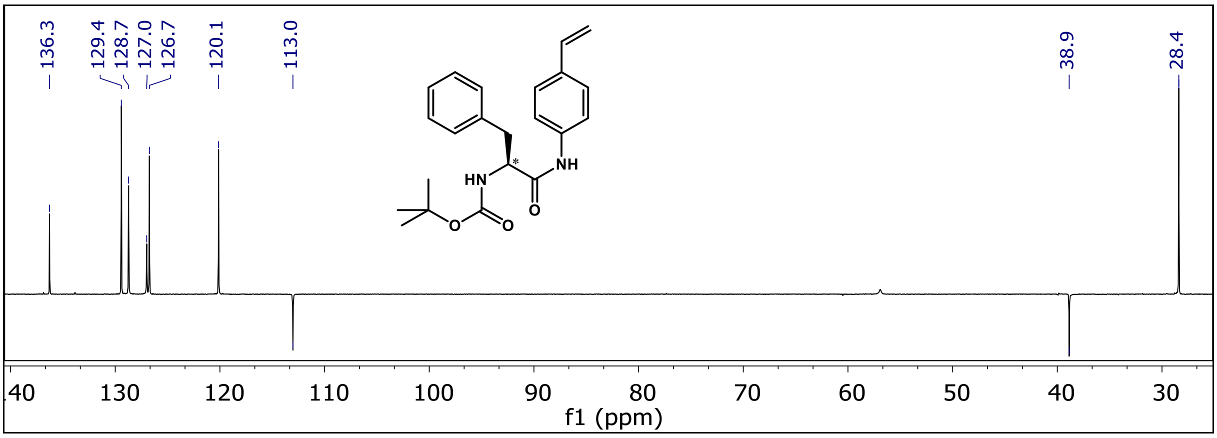

benzyl (tert-butoxycarbonyl)-L-phenylalanyl-L-valinate

(P4L)

^{1}H NMR (300 MHz, $CDCl_3$)

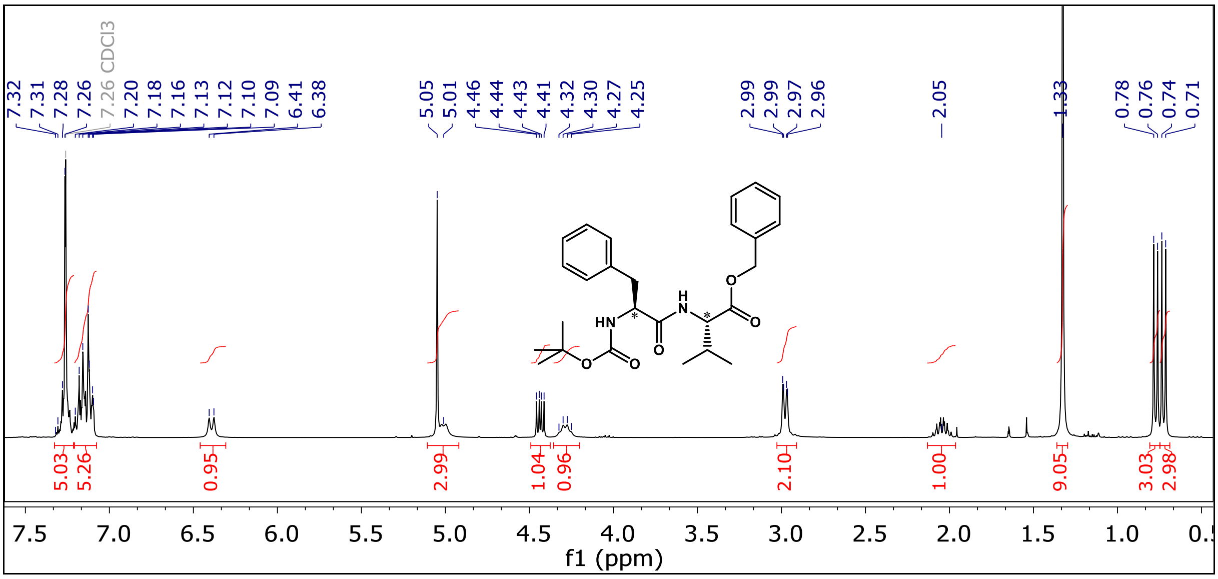

^{13}C NMR (75 MHz, $CDCl_3$)

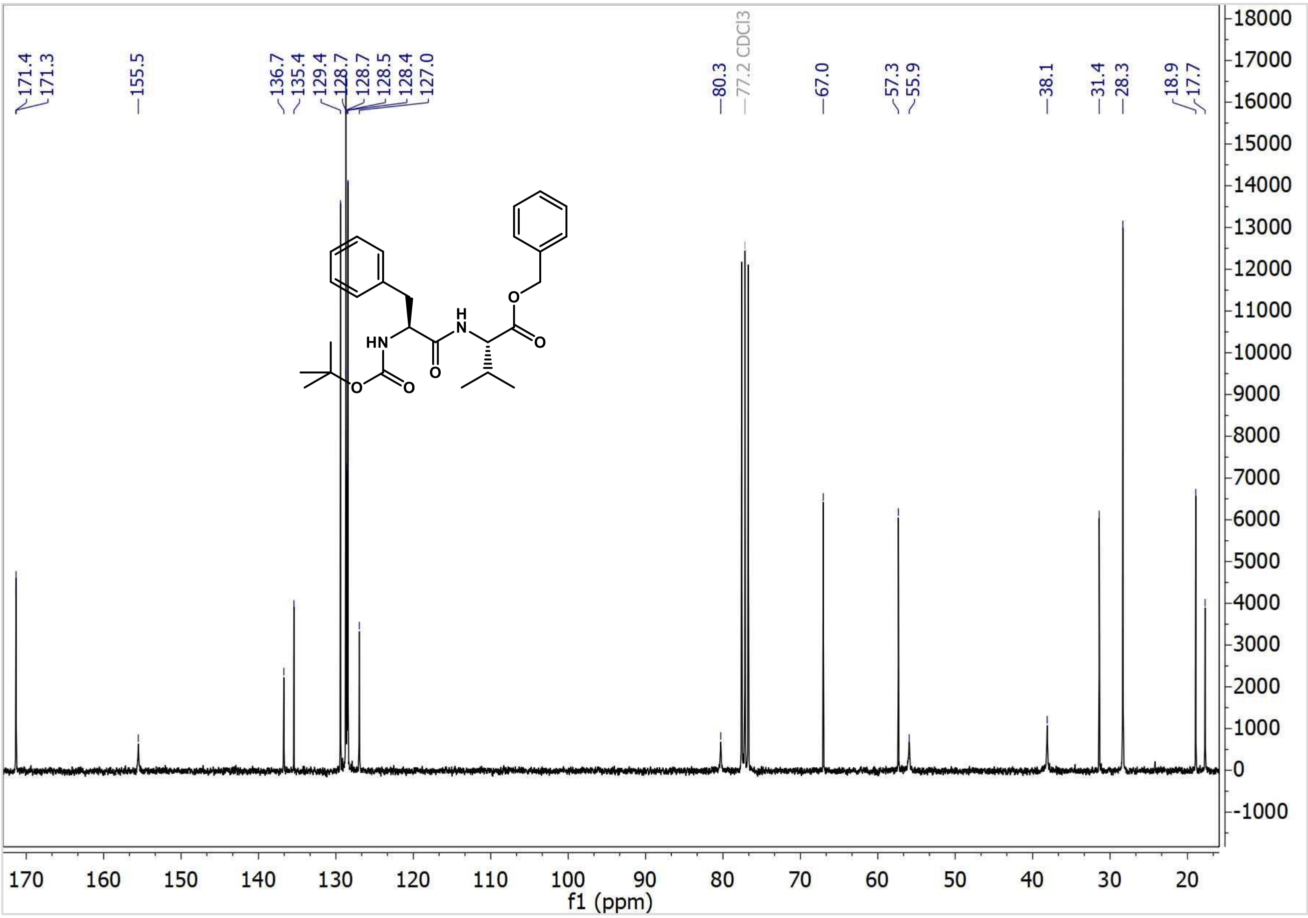

(tert-butoxycarbonyl)-L-phenylalanyl-L-valine

(P5L)

^{1}H NMR (400 MHz, MeOD)

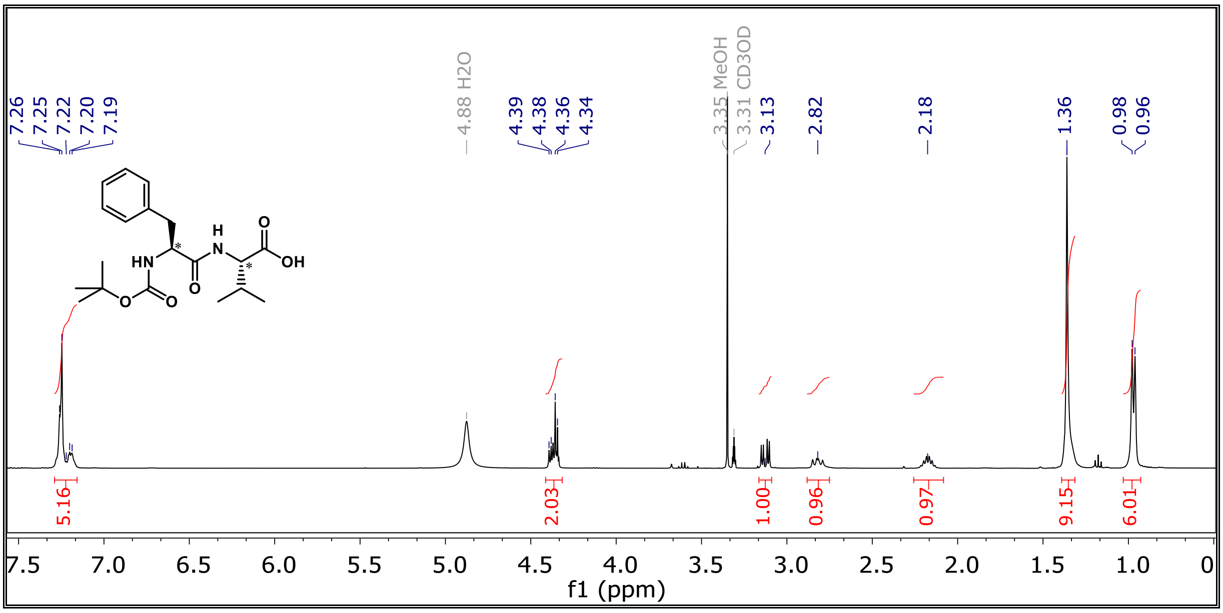

^{13}C NMR (75 MHz, MeOD)

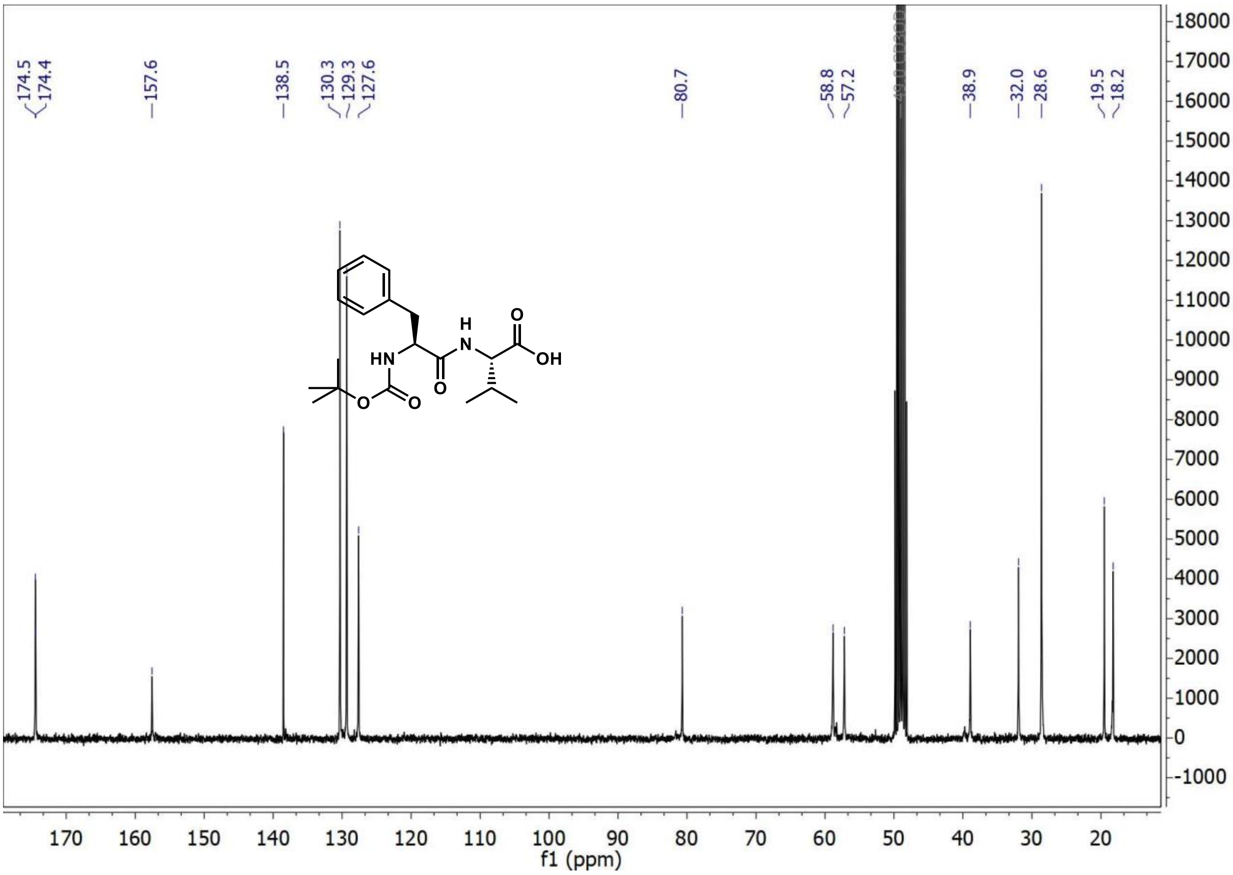

benzyl (tert-butoxycarbonyl)-L-phenylalanyl-L-valyl-L-phenylalaninate

(P6L)

^{1}H NMR (400 MHz, $CDCl_3$)

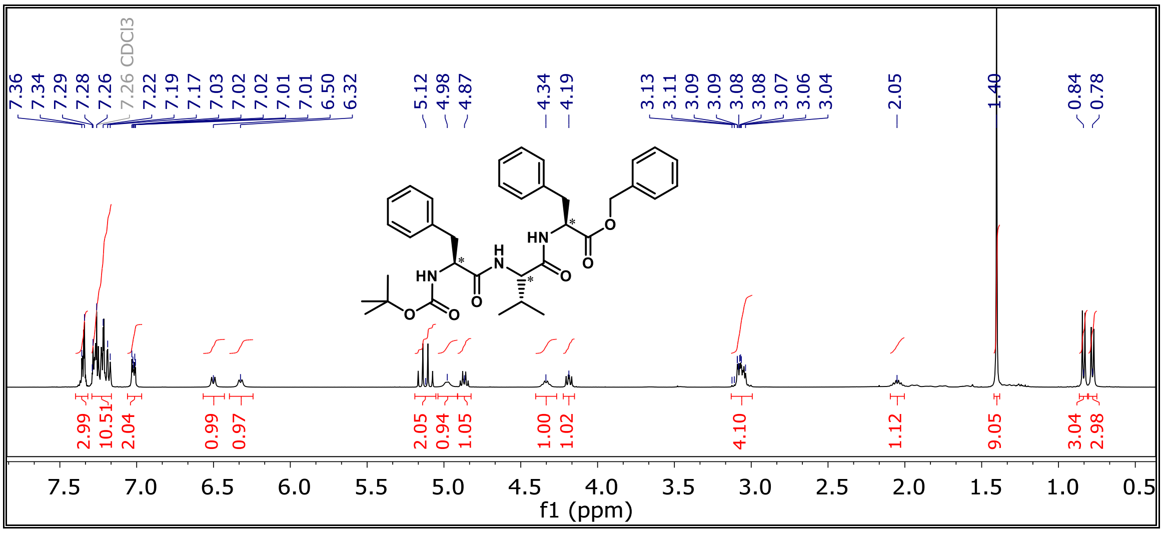

^{13}C NMR (75 MHz, $CDCl_3$)

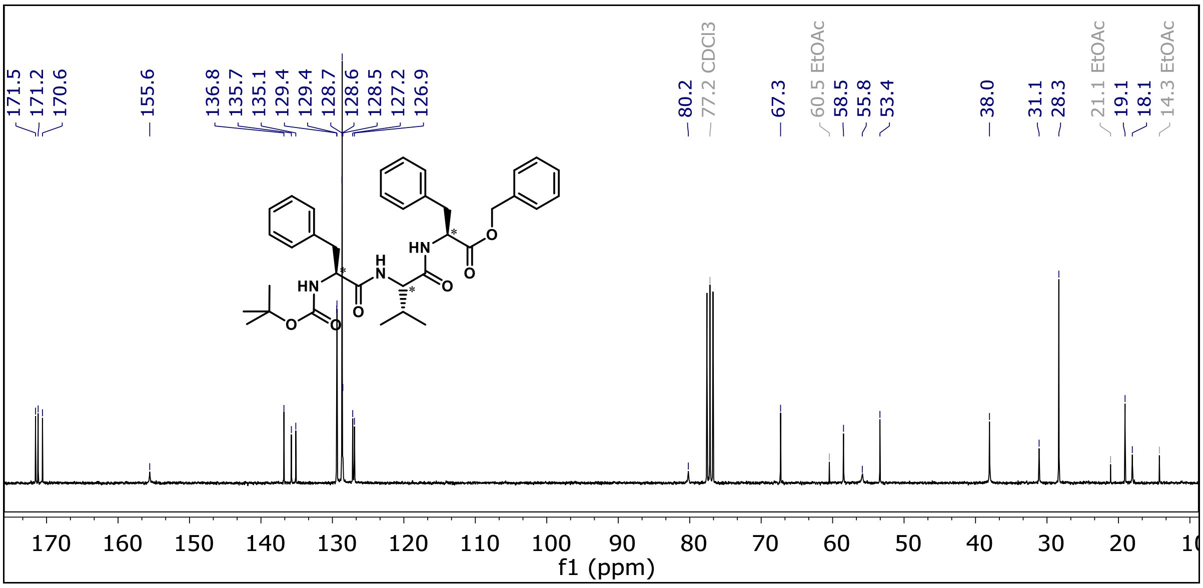

DEPT 135 (75 MHz, $CDCl_3$)

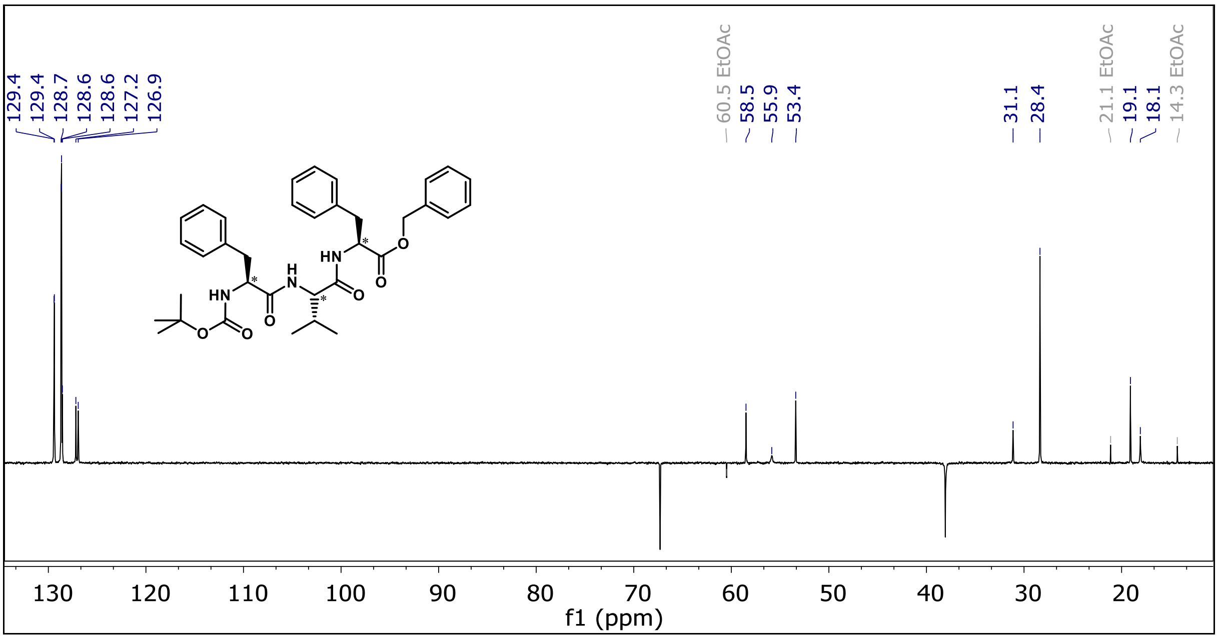

HRMS (ESI)

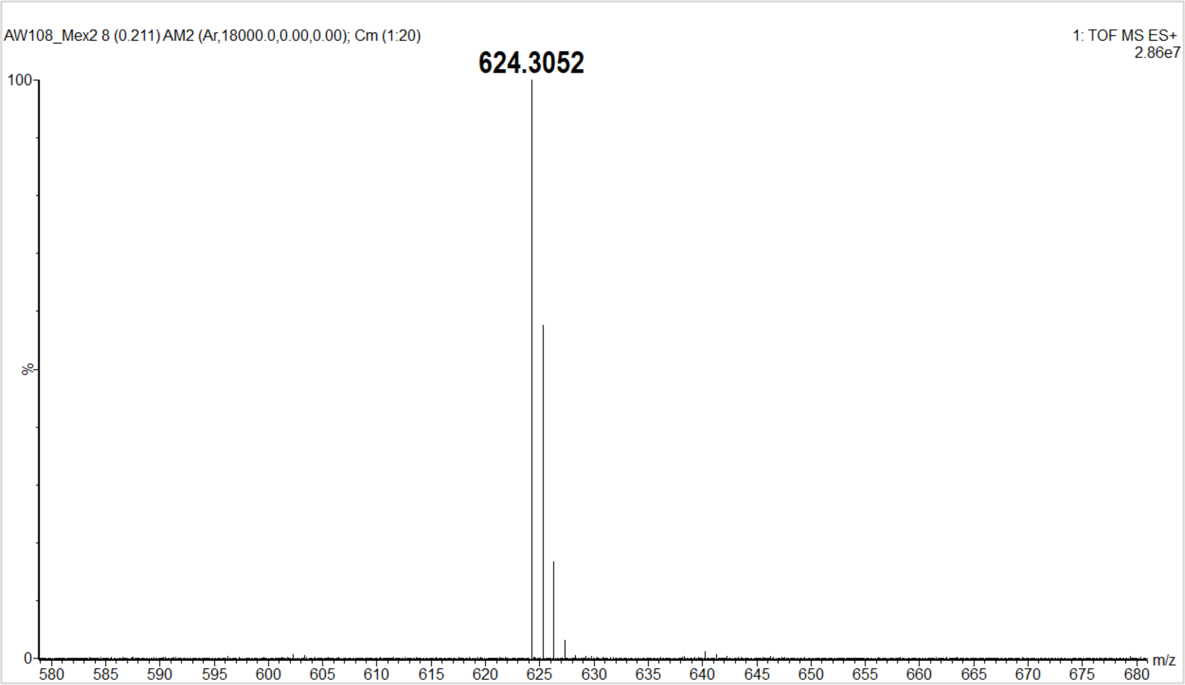

(tert-butoxycarbonyl)-L-phenylalanyl-L-valyl-L-phenylalanine

(P7L)

^{1}H NMR (300 MHz, $CDCl_3$)

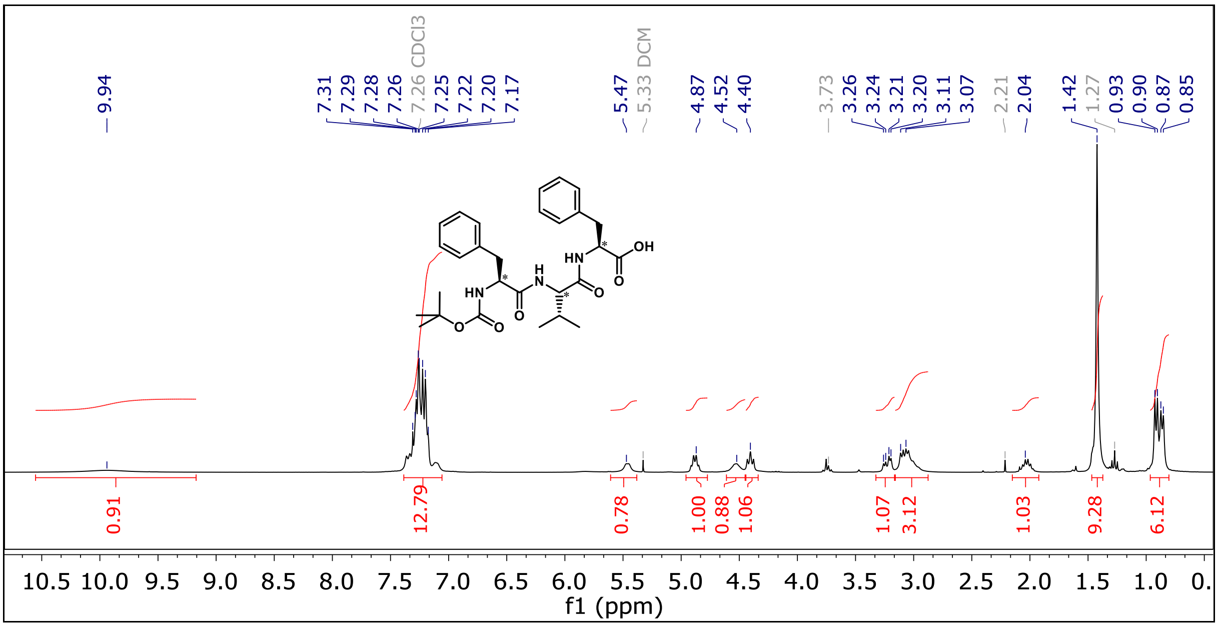

^{13}C NMR (75 MHz, $CDCl_3$)

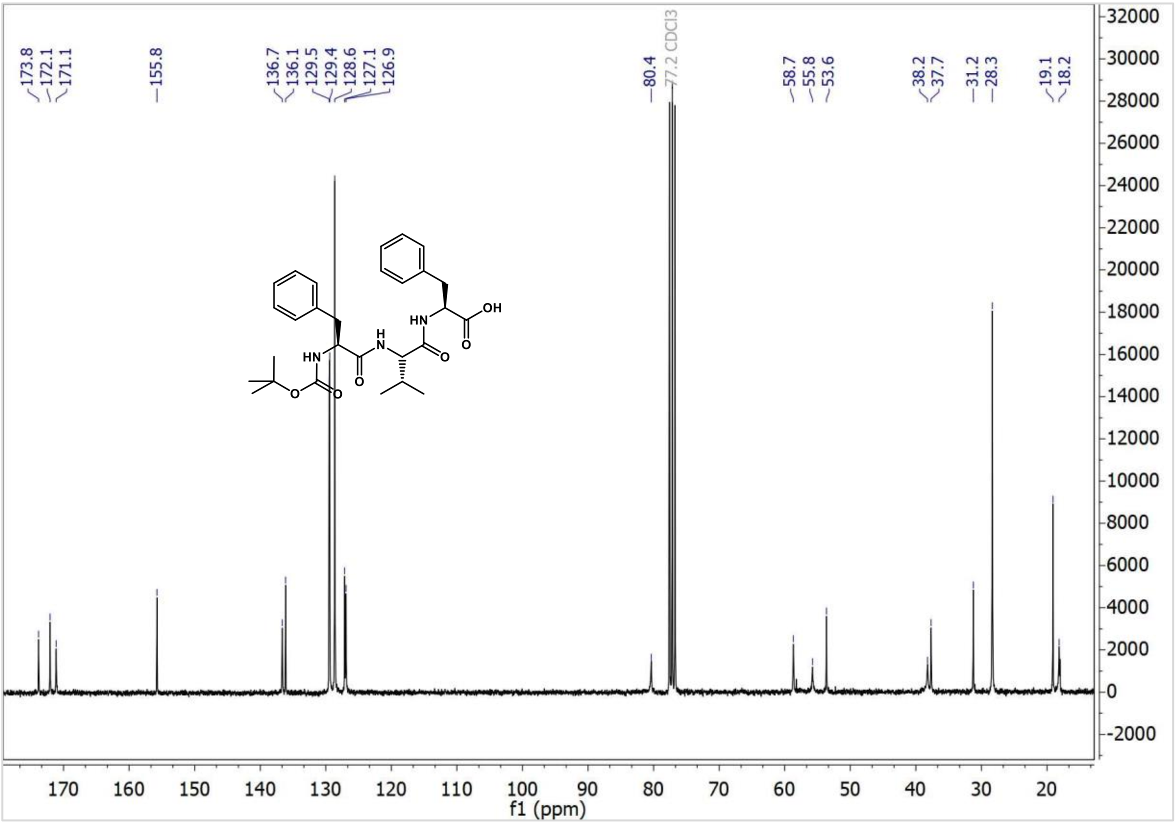

(S)-1-(((S)-1-(((S)-1-(benzyloxy)-1-oxo-3-phenylpropan-2-yl)amino)-3-methyl-1-oxobutan-2-yl)amino)-1-oxo-3-phenylpropan-2-aminium trifluoroacetate

(P8L)

^{1}H NMR (400 MHz, MeOD)

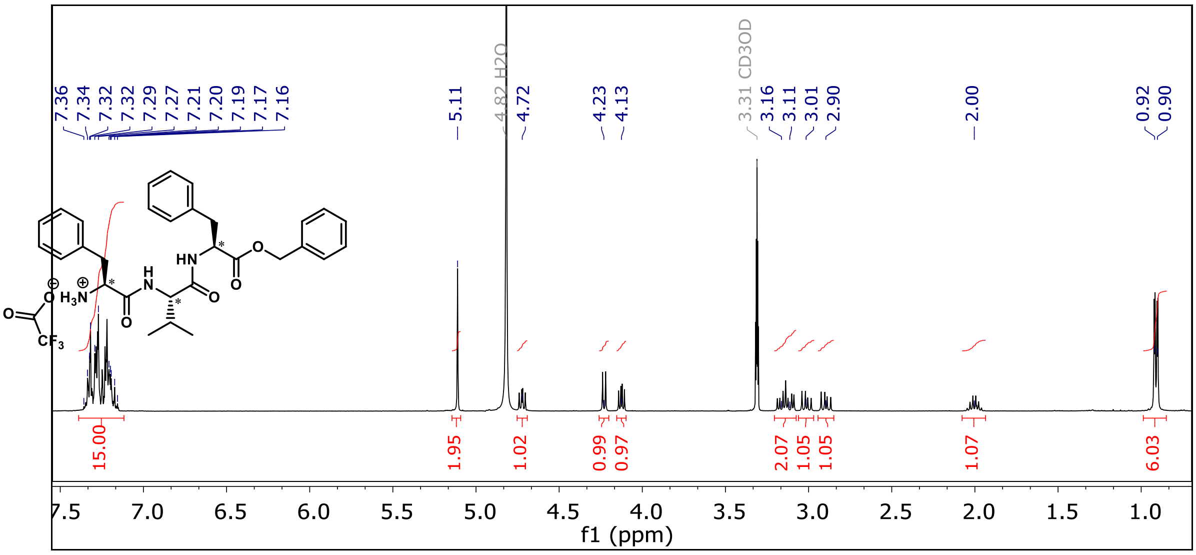

^{19}F NMR (376 MHz, MeOD)

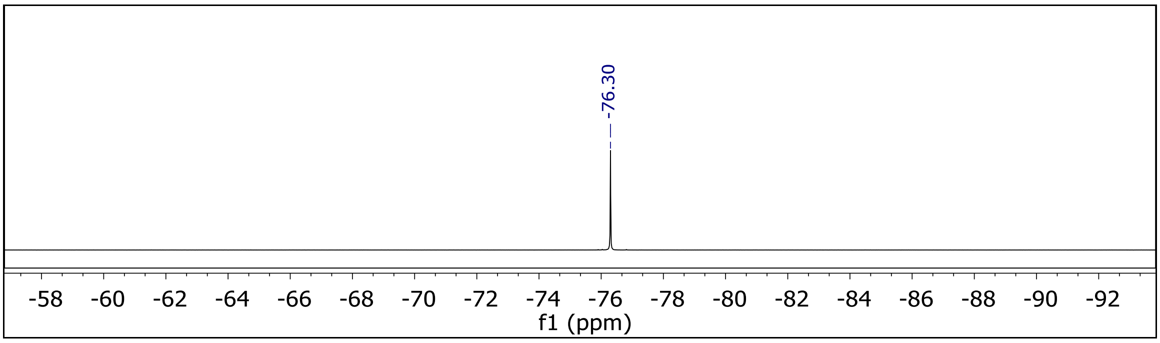

^{13}C NMR (101 MHz, MeOD-$CDCl_3$ (1 : 1 ; v : v))

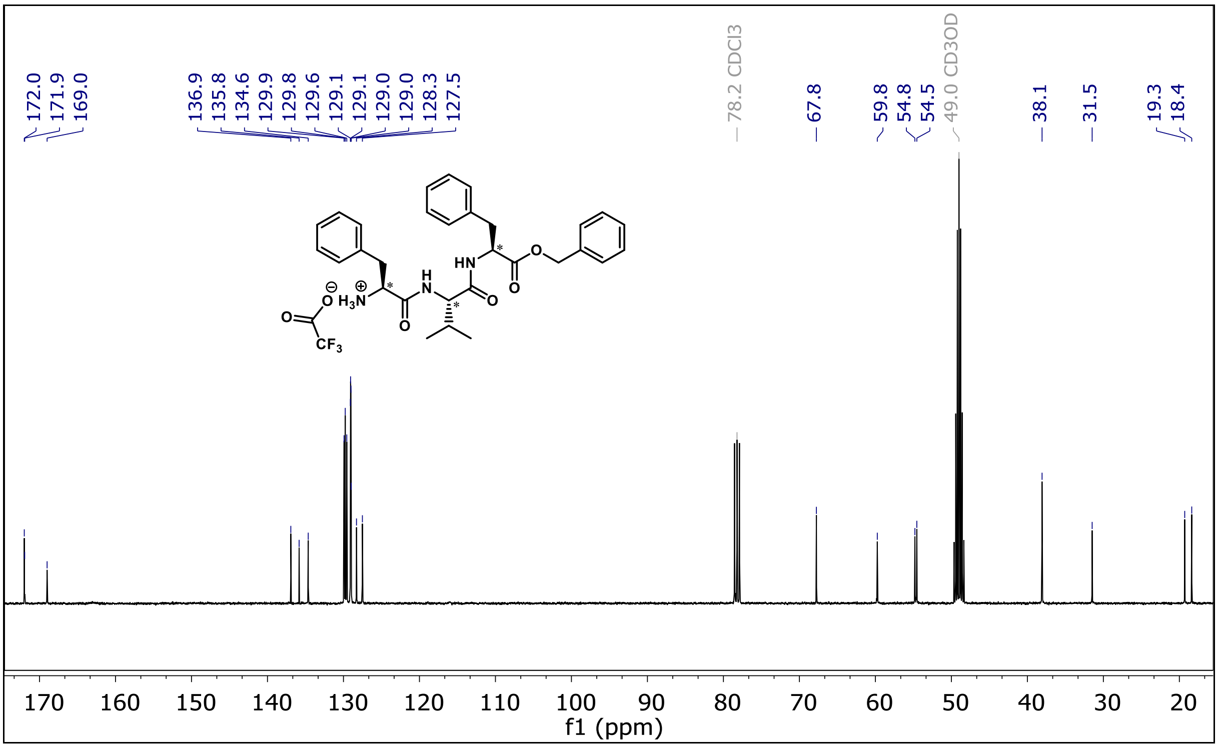

DEPT 135 (101 MHz, MeOD-$CDCl_3$ (1 : 1 ; v : v))

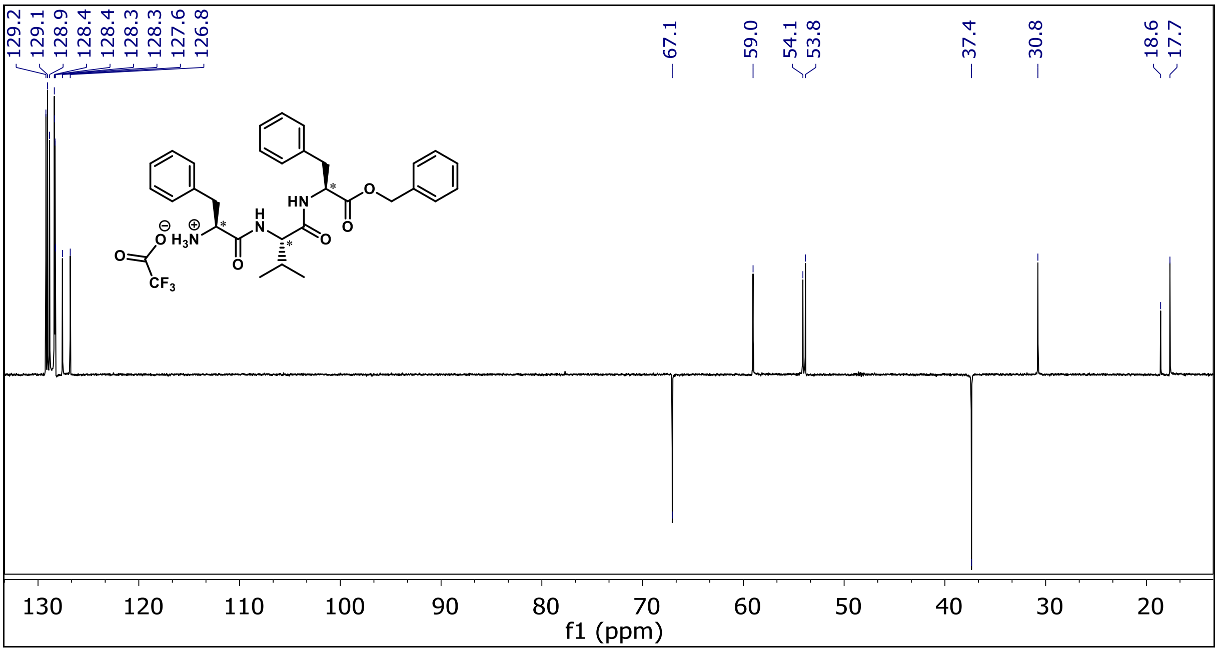

HMRS (ESI)

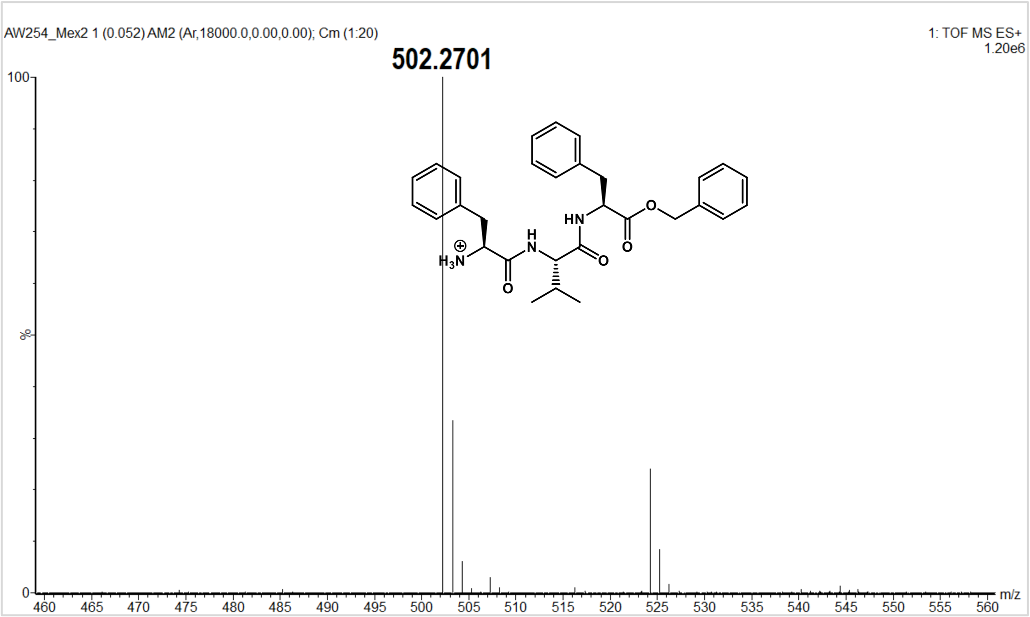

4-(benzyloxy)-4-oxobutanoic acid

^{1}H NMR (400 MHz, MeOD)

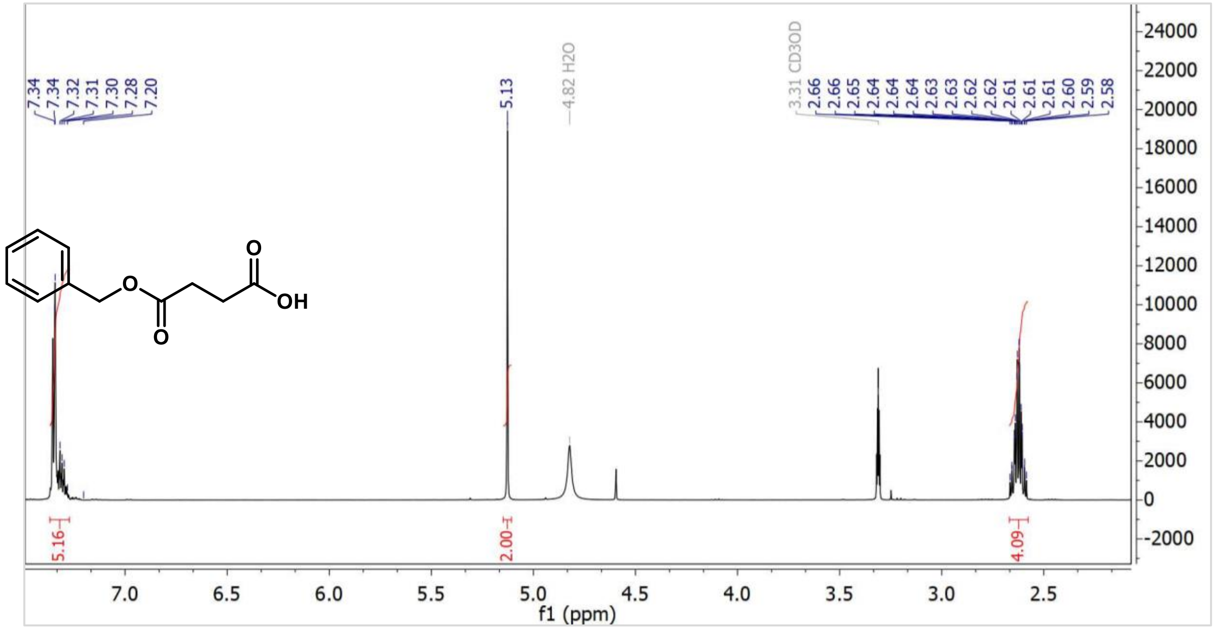

benzyl 4-(((S)-1-(((S)-1-(((S)-1-(benzyloxy)-1-oxo-3-phenylpropan-2-yl)amino)-3-methyl-1-oxobutan-2-yl)amino)-1-oxo-3-phenylpropan-2-yl)amino)-4-oxobutanoate

(P9L)

^{1}H NMR (400 MHz, $CDCl_3$)

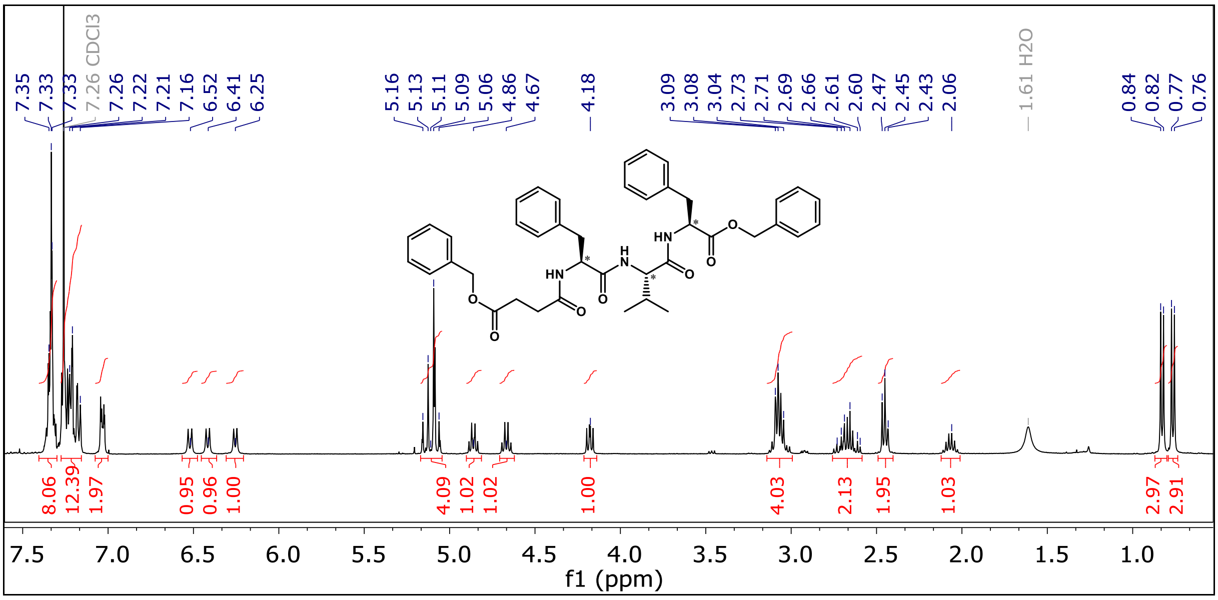

^{13}C NMR (101 MHz, $CDCl_3$)

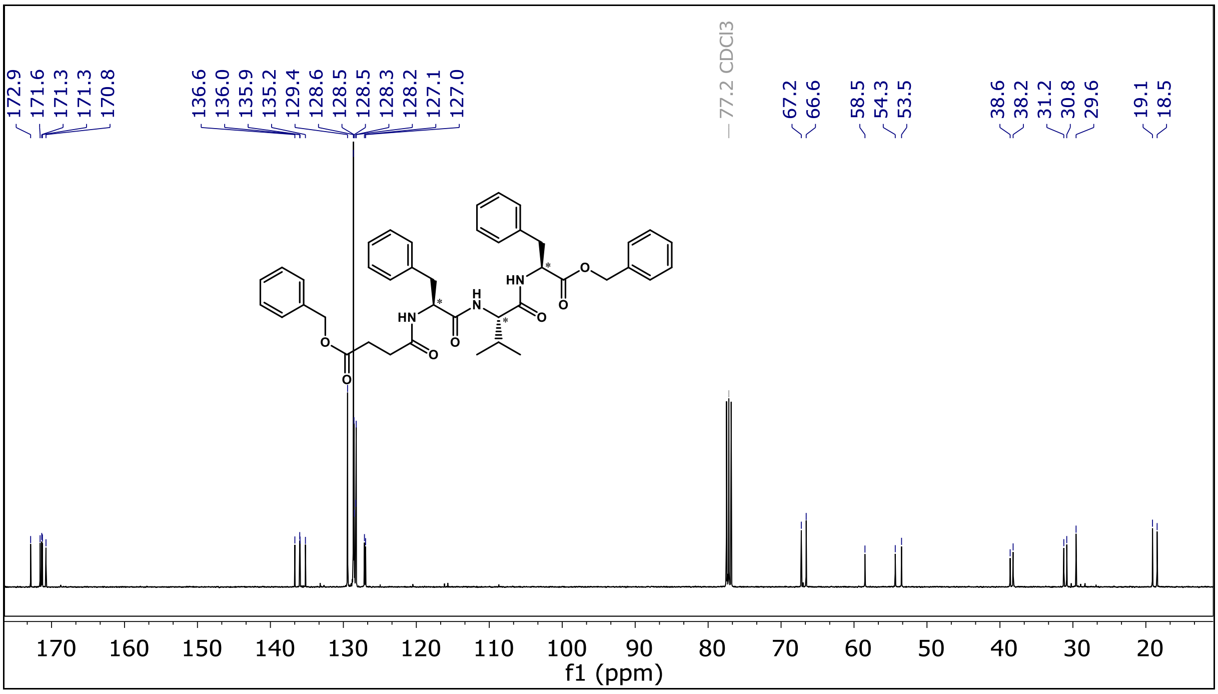

DEPT 135 (101 MHz, $CDCl_3$)

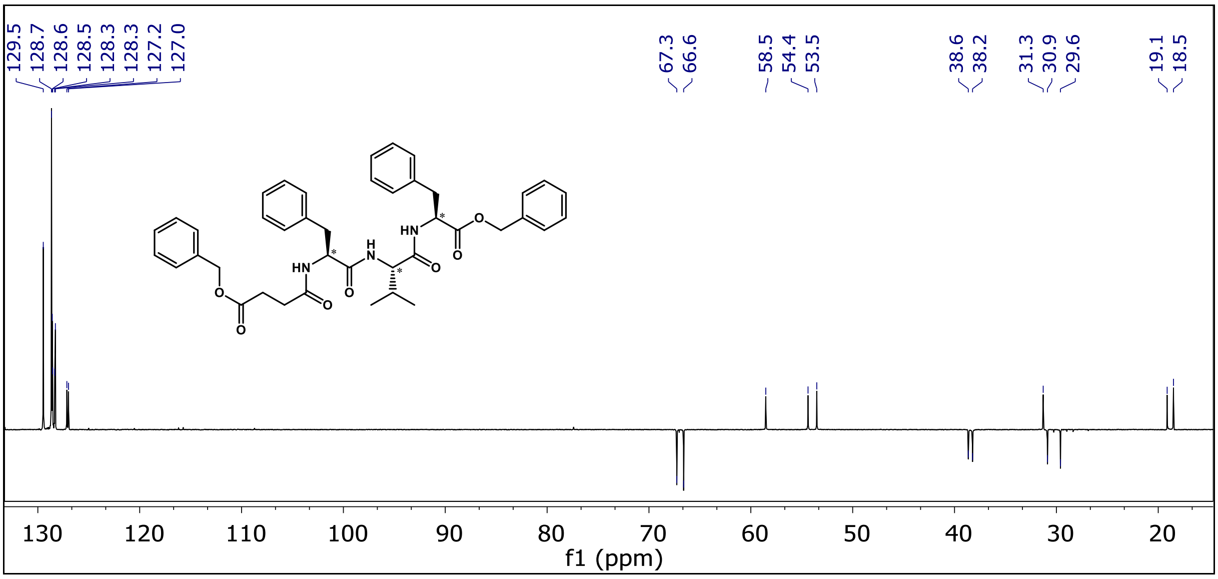

HRMS (ESI)

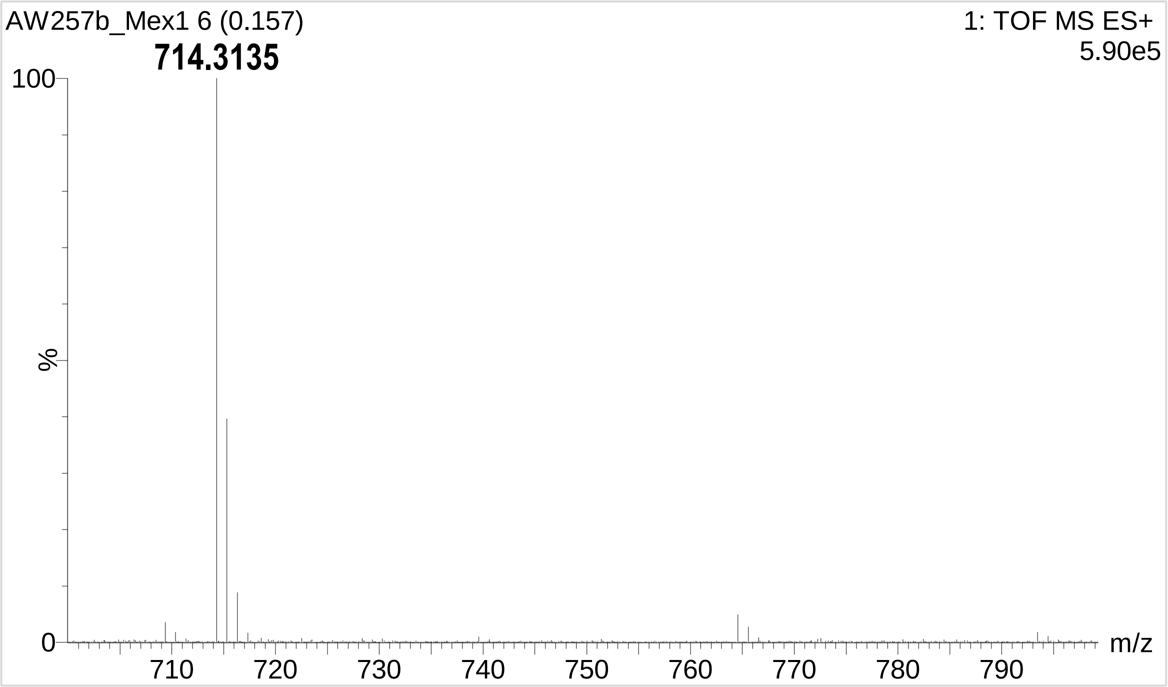

4-(((S)-1-(((S)-1-(((S)-1-carboxy-2-phenylethyl)amino)-3-methyl-1-oxobutan-2-yl)amino)-1-oxo-3-phenylpropan-2-yl)amino)-4-oxobutanoic acid

(P10L)

^{1}H NMR (400 MHz, MeOD)

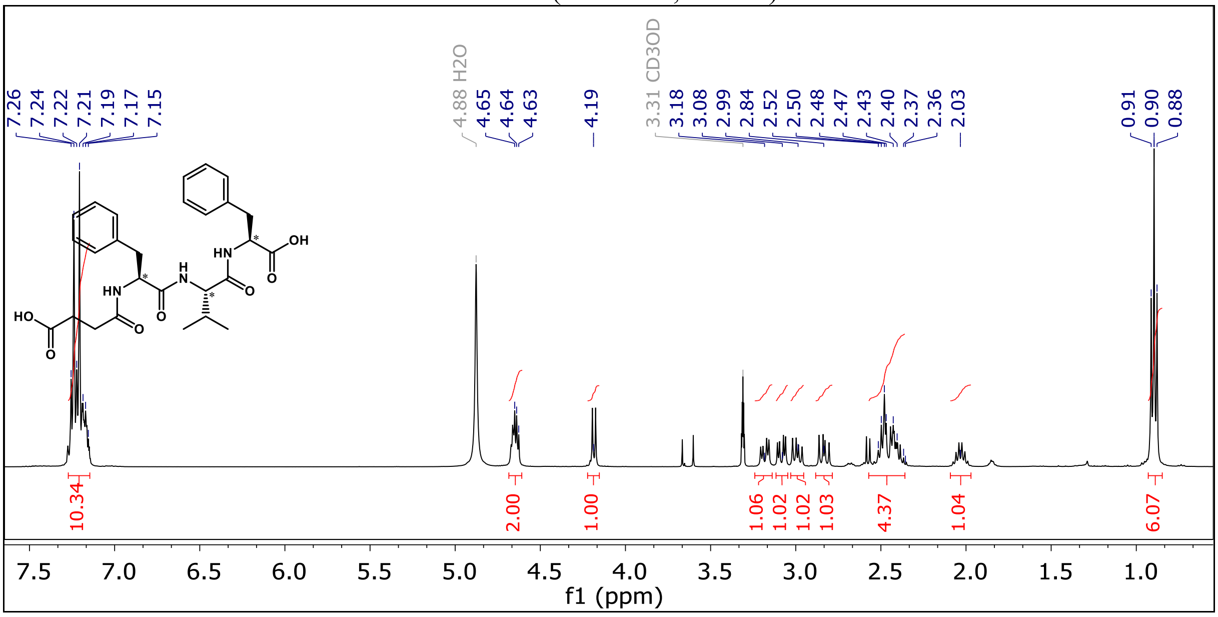

^{13}C NMR (101 MHz, MeOD)

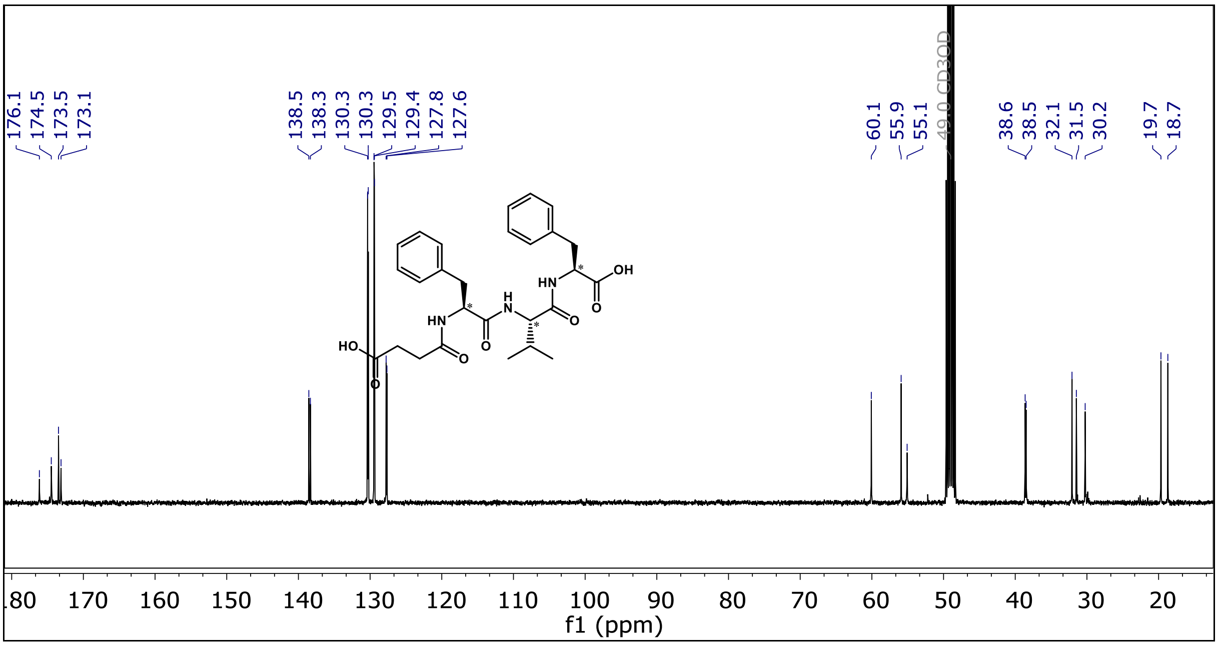

DEPT 135 (101 MHz, MeOD)

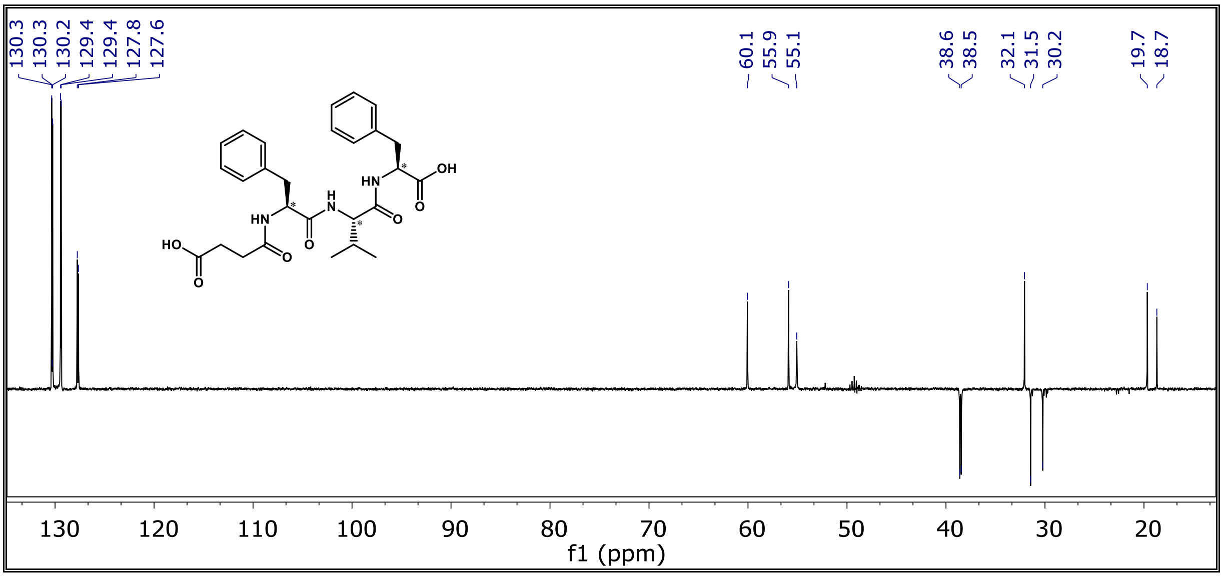

HRMS (ESI)

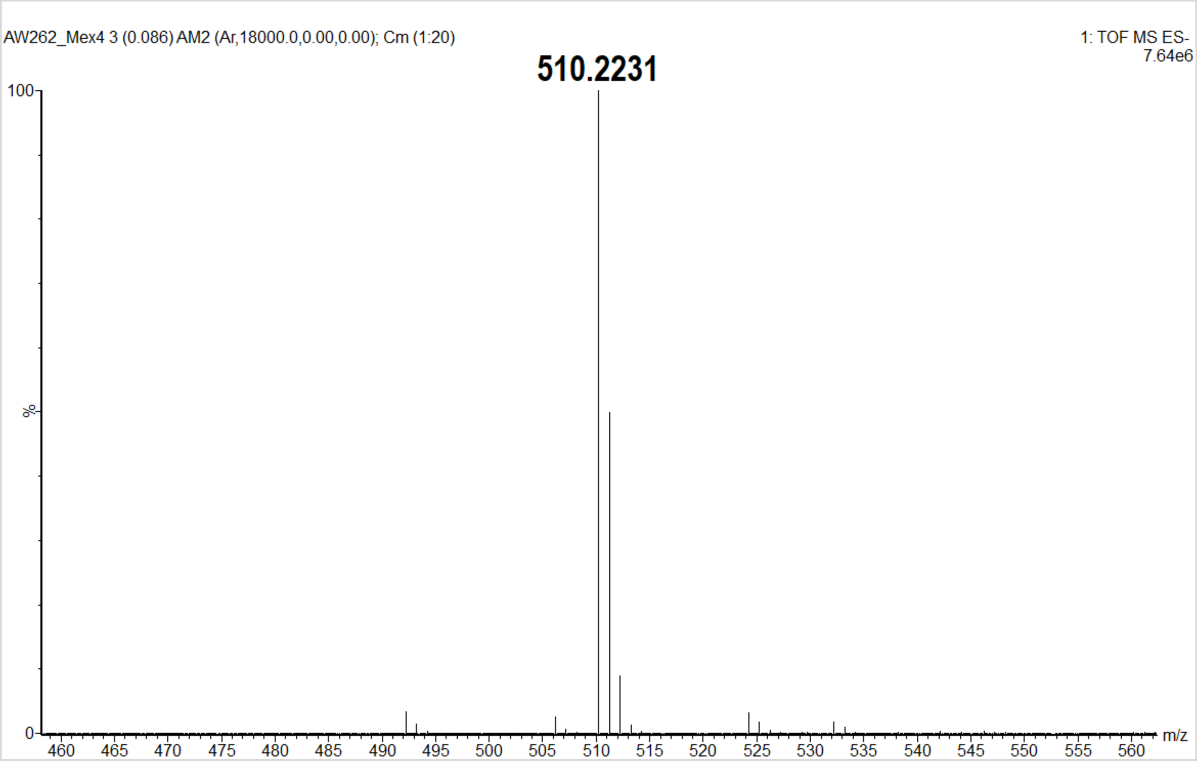

(S)-1-(((S)-1-(((S)-1-carboxy-2-phenylethyl)amino)-3-methyl-1-oxobutan-2-yl)amino)-1-oxo-3-phenylpropan-2-aminium trifluoroacetate

(P11L)

^{1}H NMR (400 MHz, MeOD)

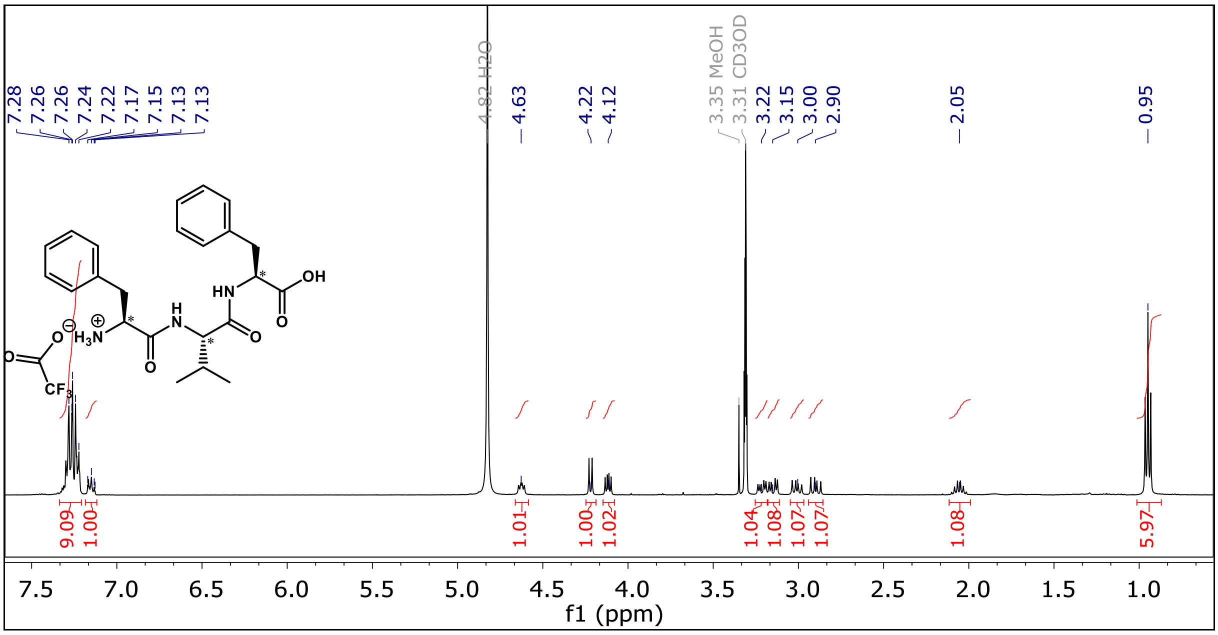

^{19}F NMR (376 MHz, MeOD)

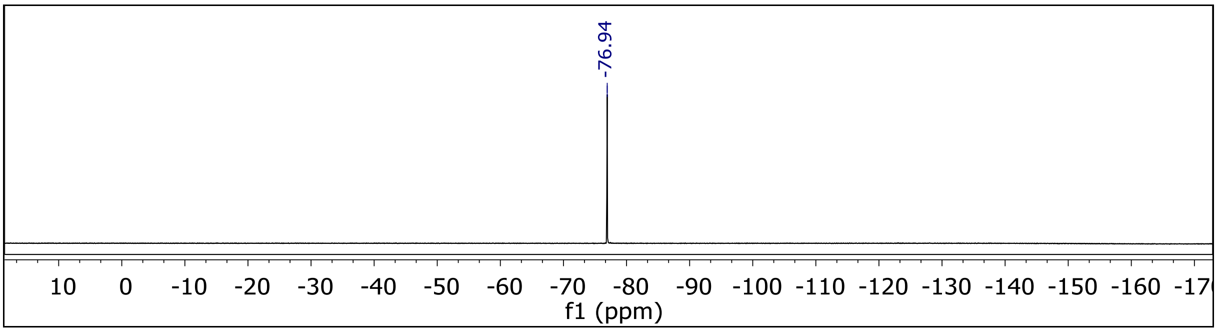

^{13}C NMR (101 MHz, DMSO)

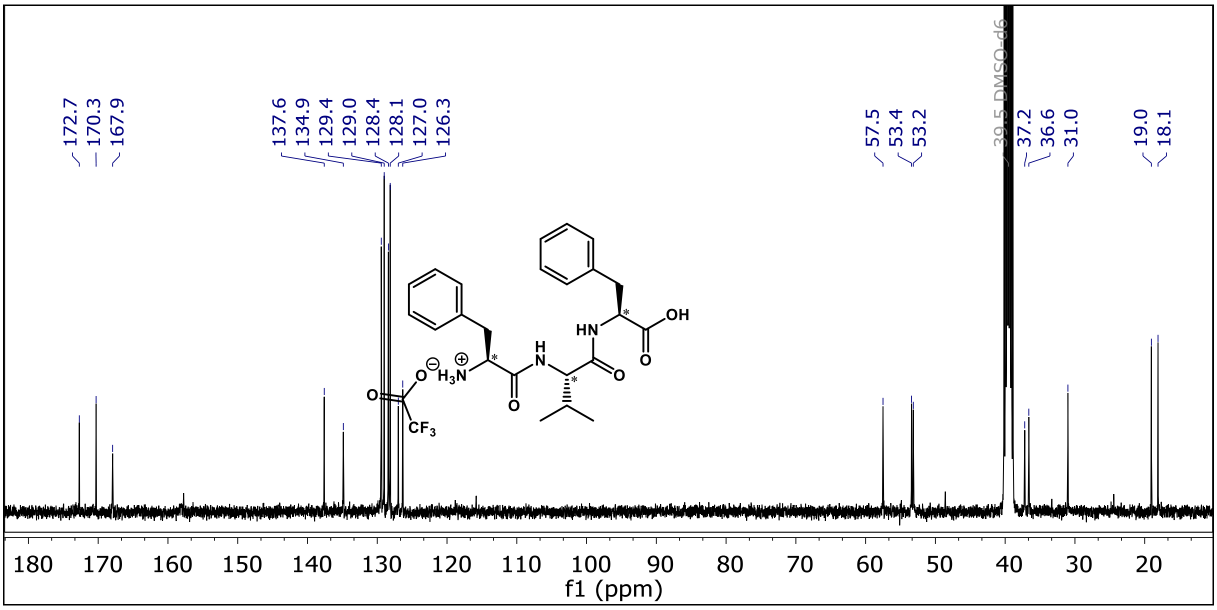

DEPT 135 (101 MHz, DMSO)

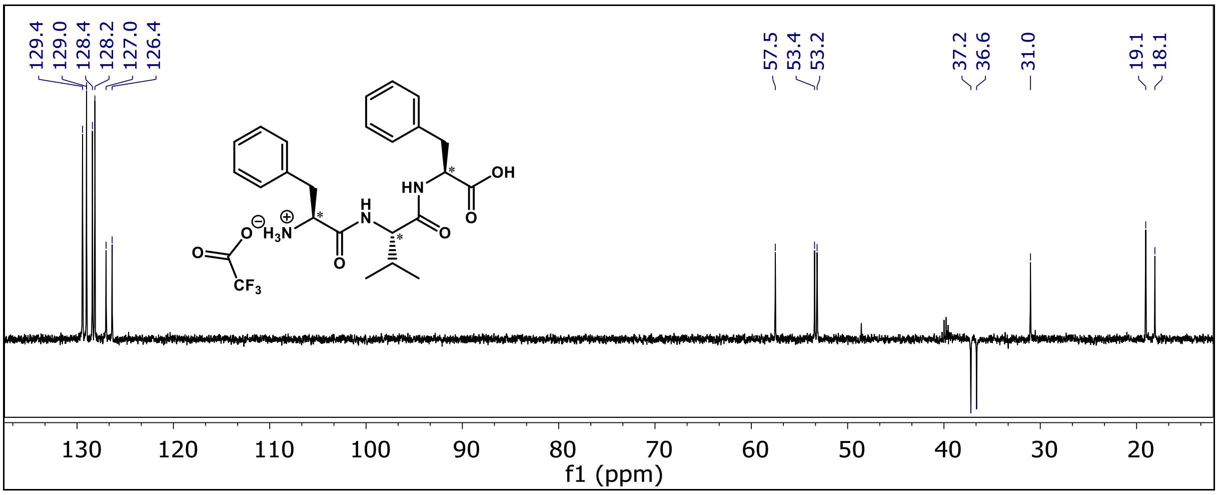

HRMS (ESI)

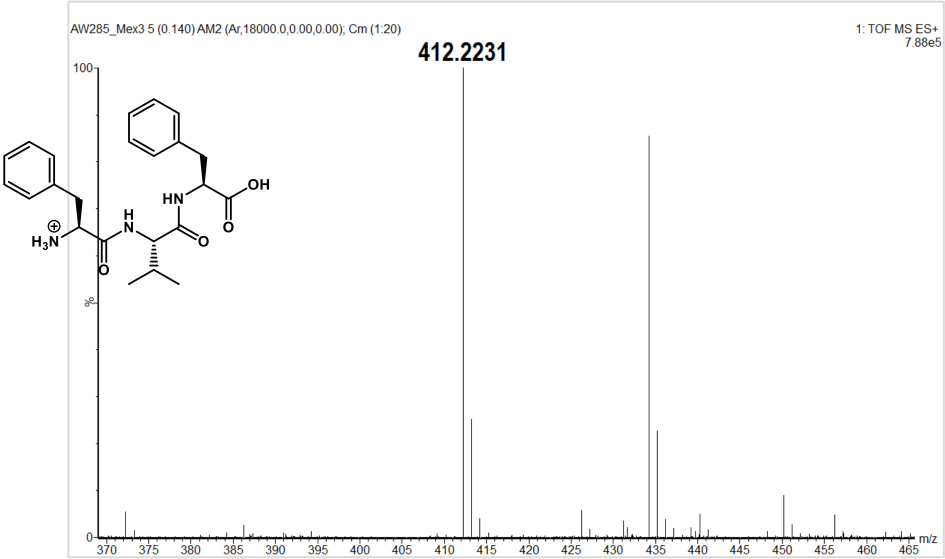

(4-(benzyloxy)-4-oxobutanoyl)-L-phenylalanyl-L-valyl-L-phenylalanine

(P12L)

^{1}H NMR (400 MHz, MeOD)

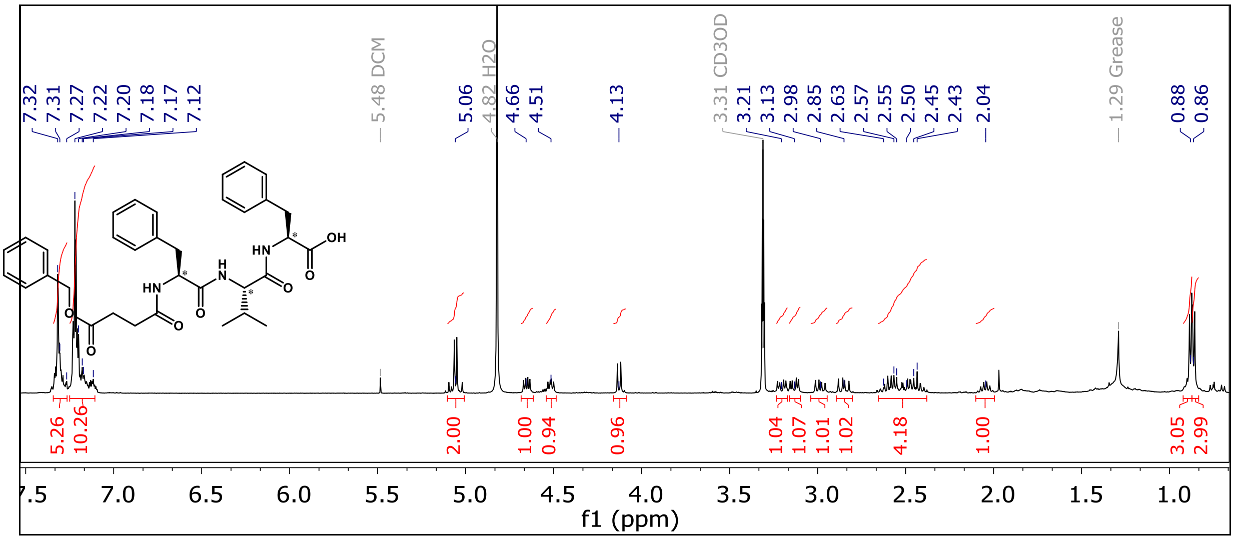

^{13}C NMR (101 MHz, MeOD-$CDCl_3$ (1 : 1 ; v : v))

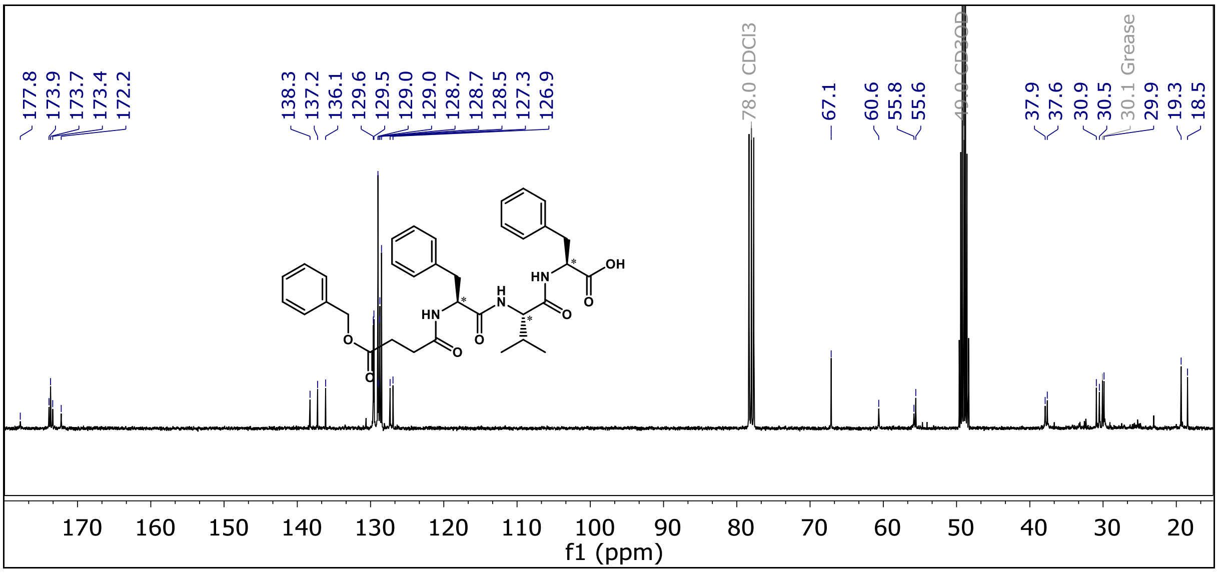

DEPT 135 (101 MHz, MeOD-CDCl$_3$ (1 : 1 ; v : v))

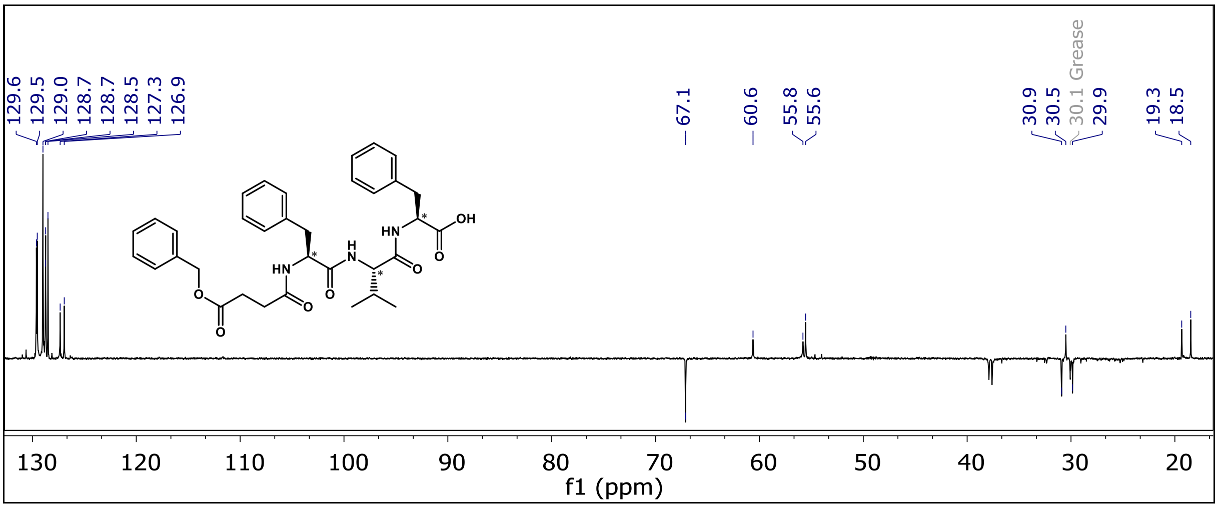

HRMS (ESI)

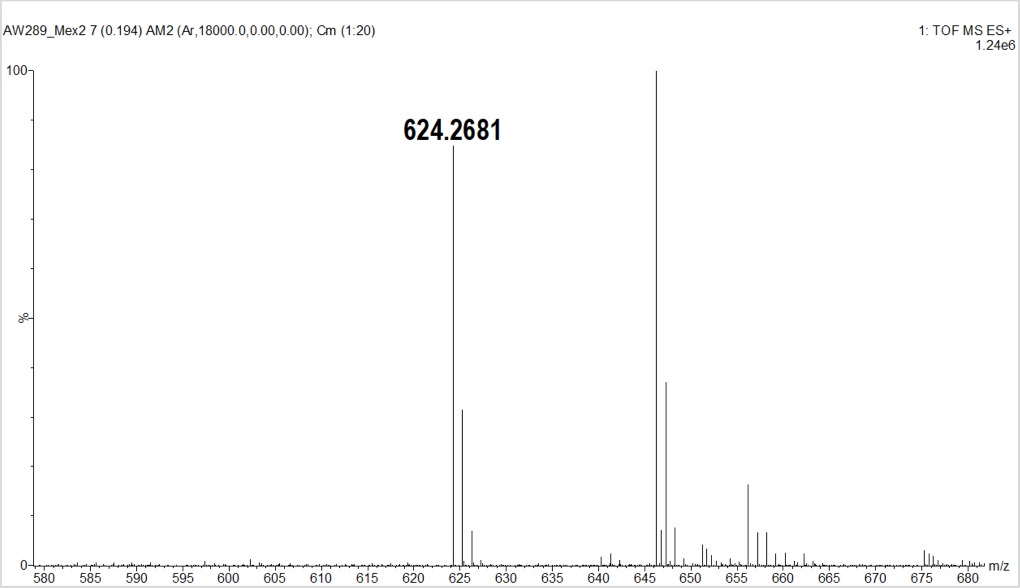

benzyl (((9H-fluoren-9-yl)methoxy)carbonyl)-L-phenylalanyl-L-valinate

(P14L)

^{1}H NMR (400 MHz, MeOD)

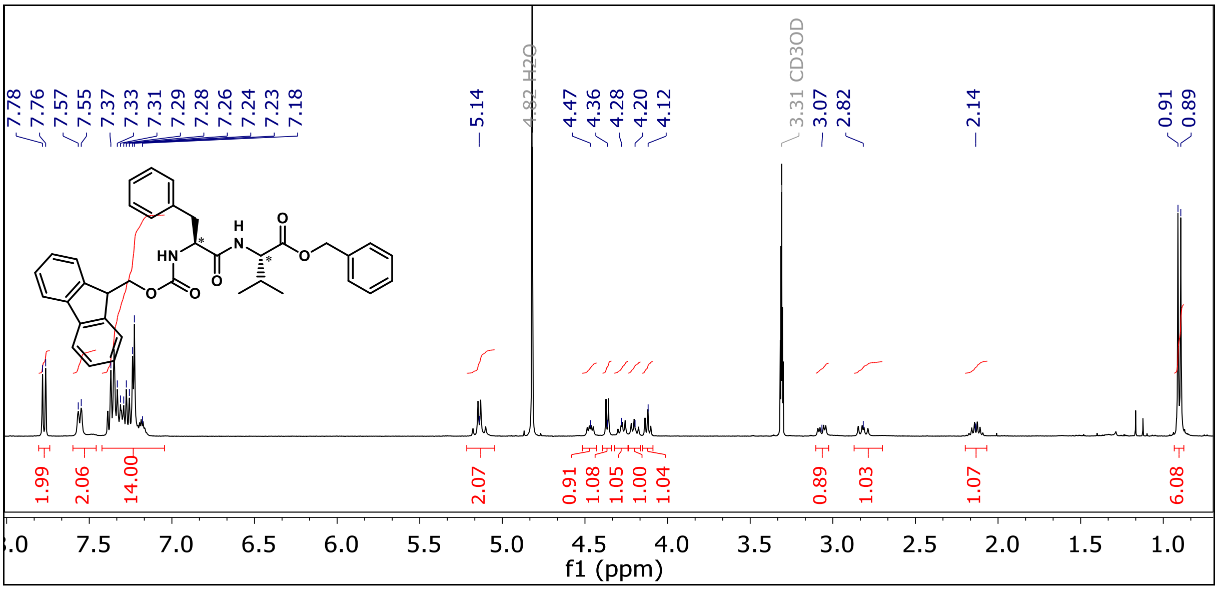

^{13}C NMR (75 MHz, $CDCl_3$)

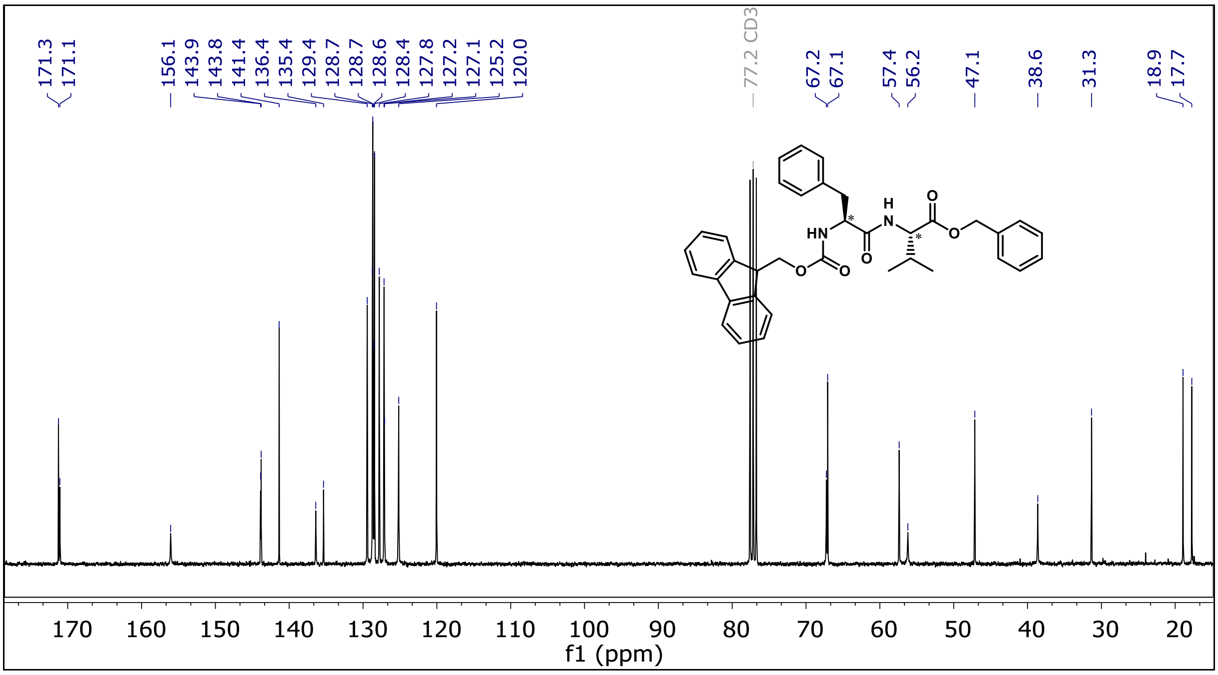

DEPT 135 (75 MHz, $CDCl_3$)

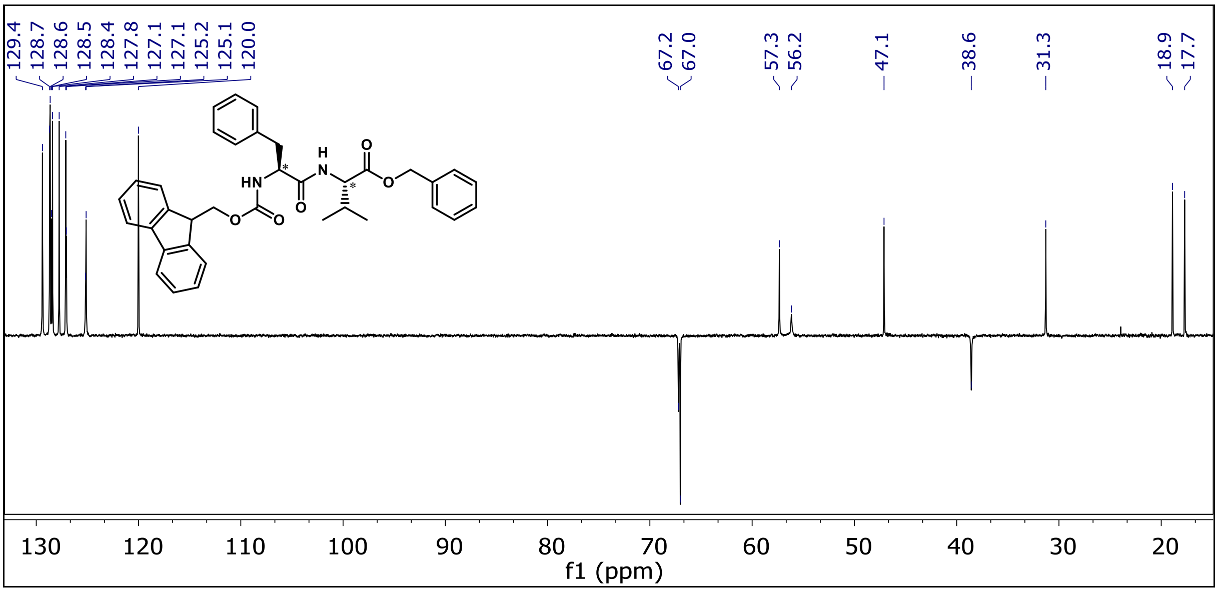
129.4
128.7
128.6
128.5
128.4
127.8
127.1
127.1
125.2
125.1
120.0
67.2
67.0
57.3
56.2
47.1
38.6
31.3
18.9
17.7
130
120
110
100
90
80
70
60
50
40
30
20
f1 (ppm)

HMRS (ESI)

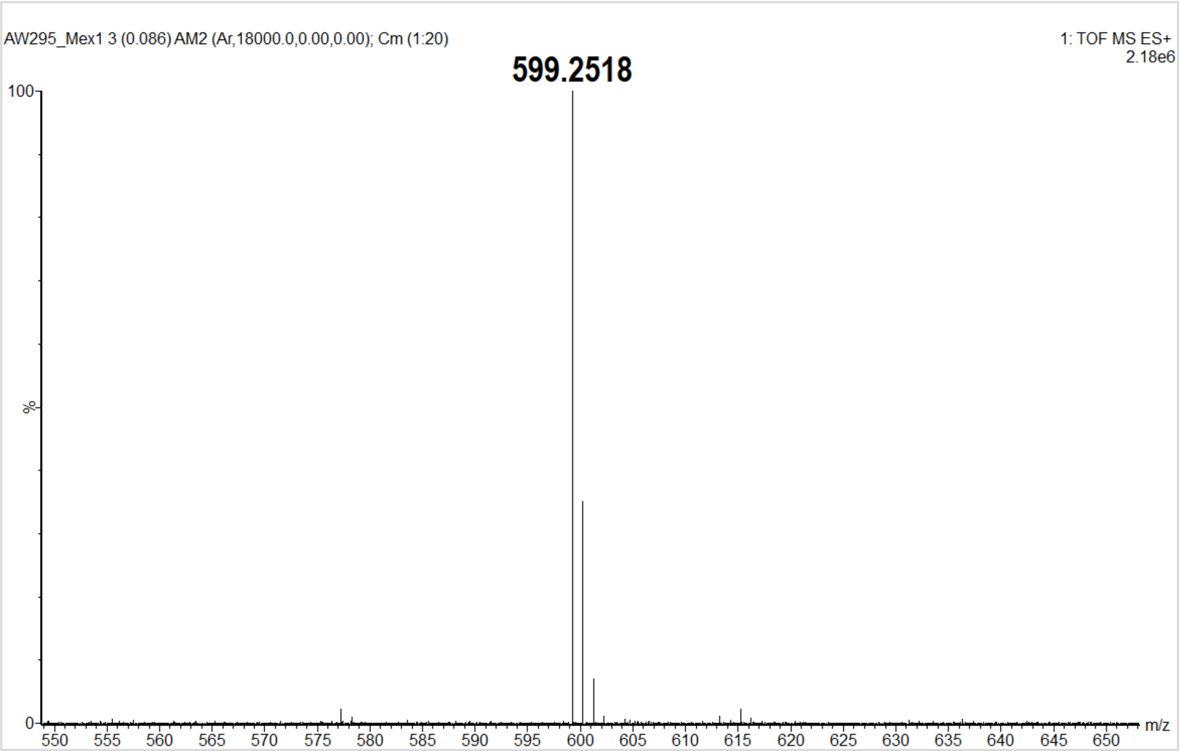
AW295_Mex1 3 (0.086) AM2 (Ar,18000.0,0.00,0.00); Cm (1:20)
1: TOF MS ES+
2.18e6
599.2518
100
%
0
550
555
560
565
570
575
580
585
590
595
600
605
610
615
620
625
630
635
640
645
650
m/z

(((9H-fluoren-9-yl)methoxy)carbonyl)-L-phenylalanyl-L-valine

(P15L)

^{1}H NMR (400 MHz, MeOD)

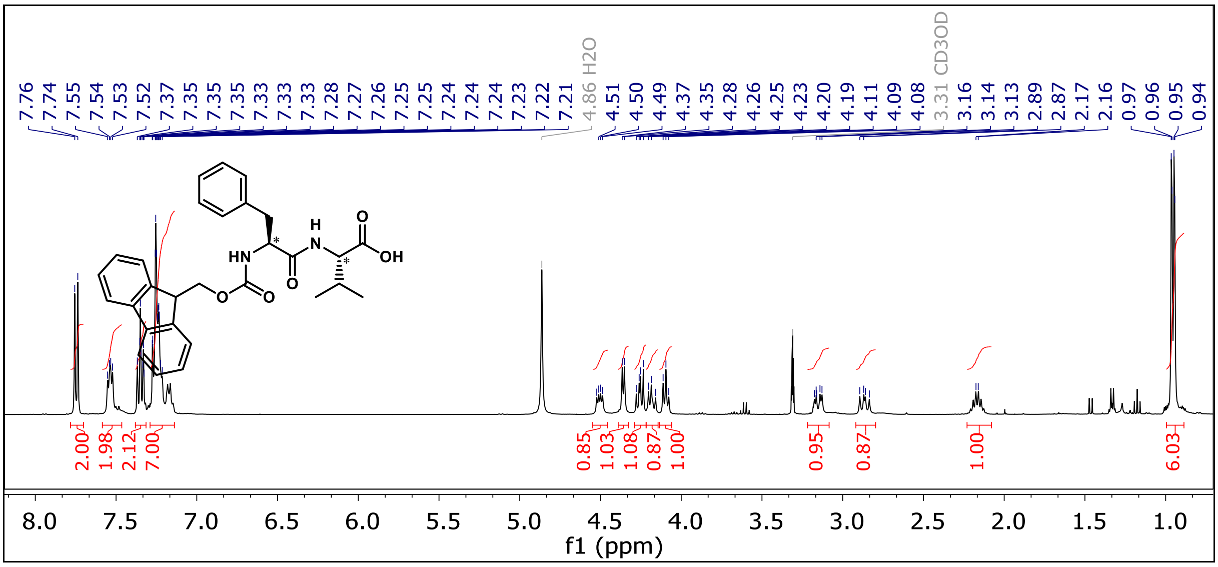

^{13}C NMR (101 MHz, MeOD)

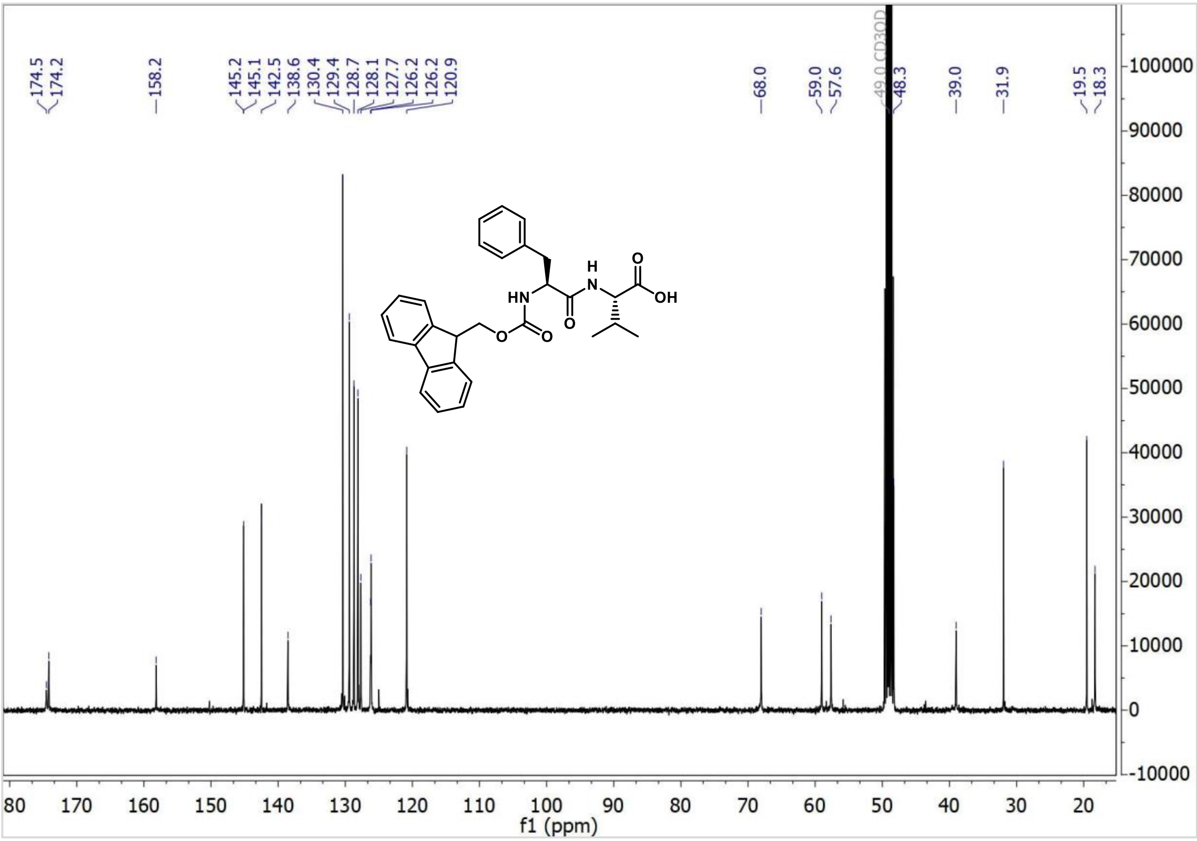

benzyl (((9H-fluoren-9-yl)methoxy)carbonyl)-L-phenylalanyl-L-valyl-L-phenylalaninate

(P16L)

^{1}H NMR (400 MHz, $CDCl_3$)

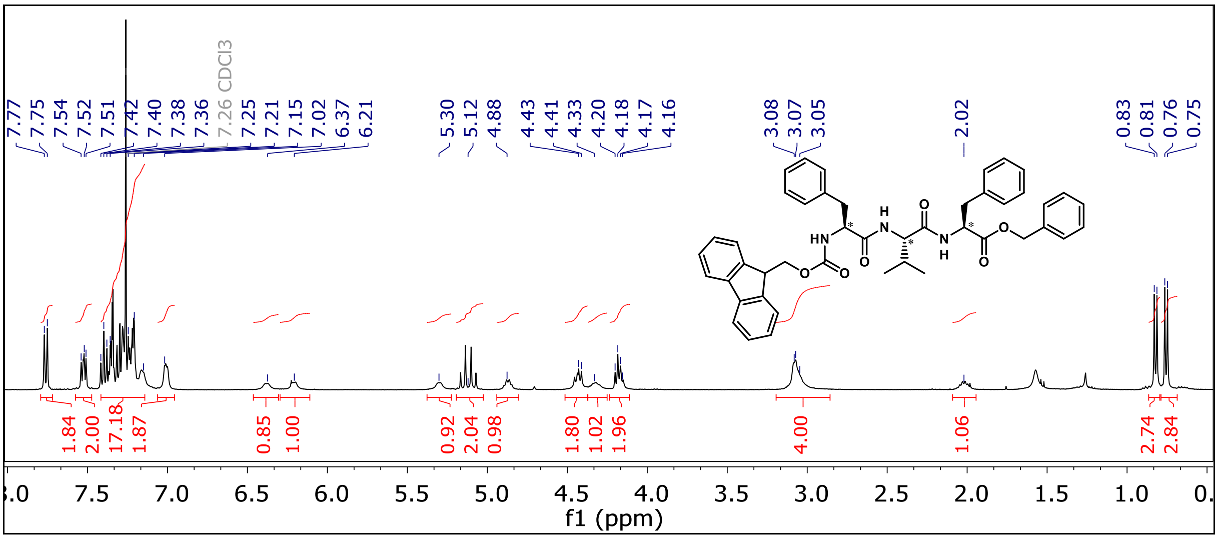

^{13}C NMR (101 MHz, $CDCl_3$)

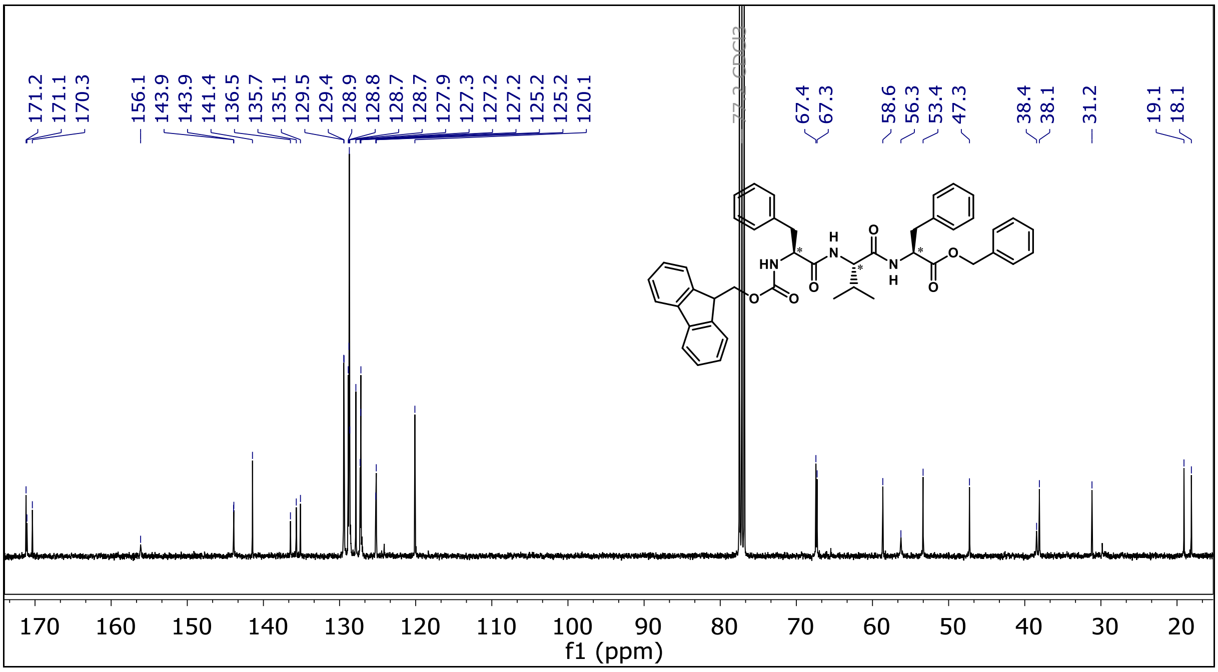

DEPT 135 (101 MHz, $CDCl_3$)

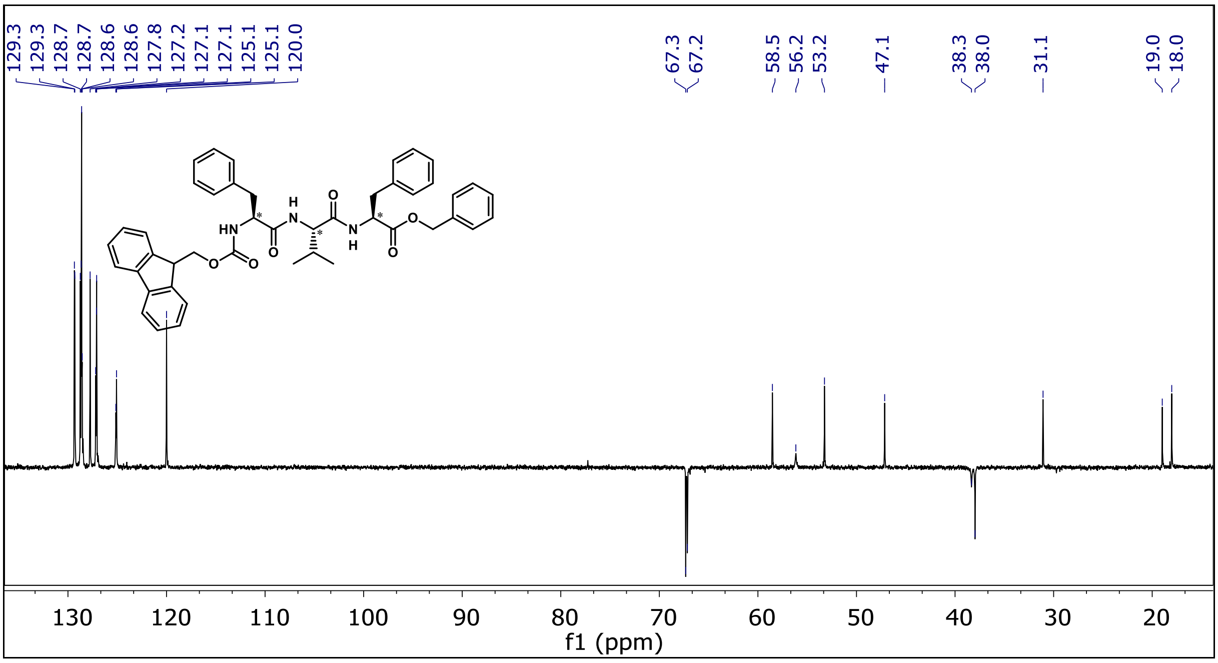
129.3
129.3
128.7
128.7
128.6
128.6
127.8
127.2
127.1
127.1
125.1
125.1
120.0
67.3
67.2
58.5
56.2
53.2
47.1
38.3
38.0
31.1
19.0
18.0
130
120
110
100
90
80
70
60
50
40
30
20
f1 (ppm)

HRMS (ESI)

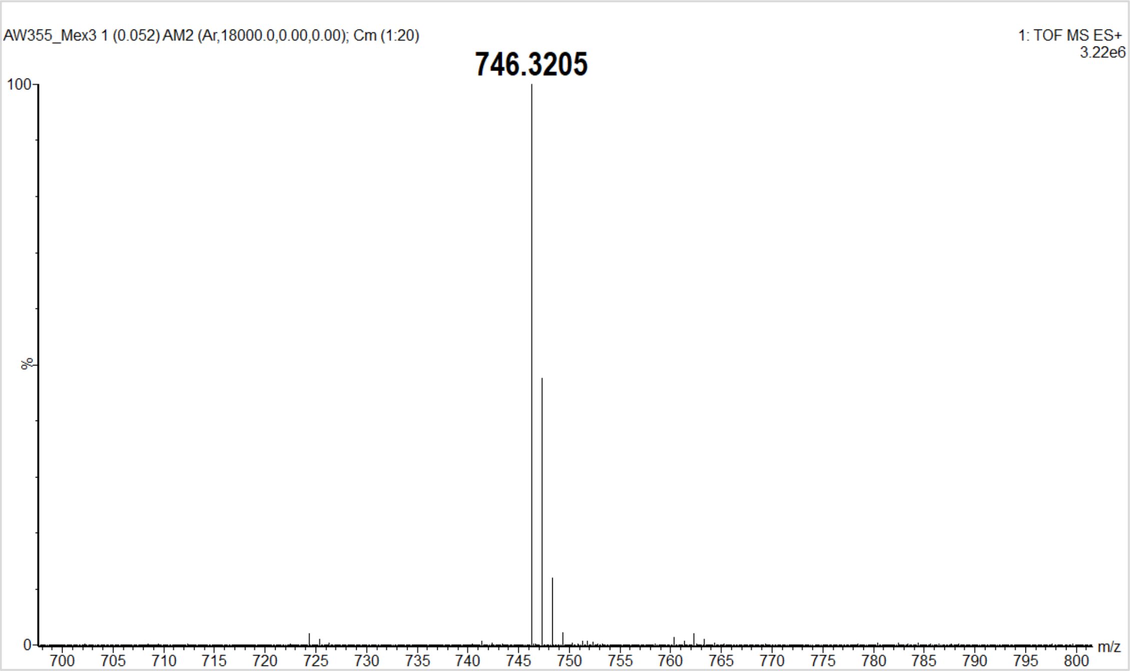
AW355_Mex3 1 (0.052) AM2 (Ar,18000.0,0.00,0.00); Cm (1:20)
1: TOF MS ES+
3.22e6
746.3205
100
%
0
700
705
710
715
720
725
730
735
740
745
750
755
760
765
770
775
780
785
790
795
800
m/z

L-phenylalanyl-L-valyl-L-phenylalanine

(P18L)

^{1}H NMR (400 MHz, D_2O)

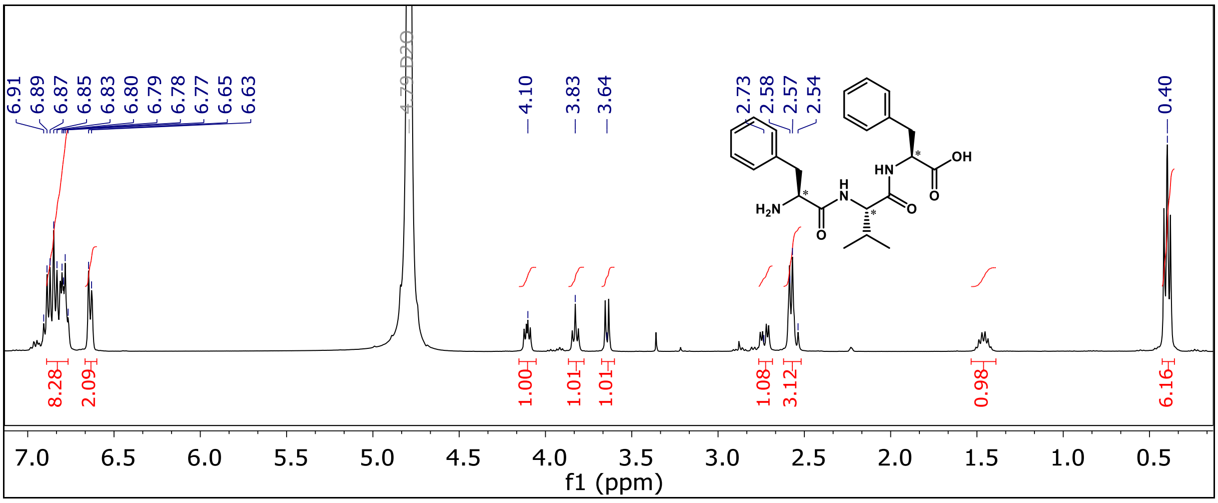

^{13}C NMR (101 MHz, D_2O-DMSO, 5% DCl)

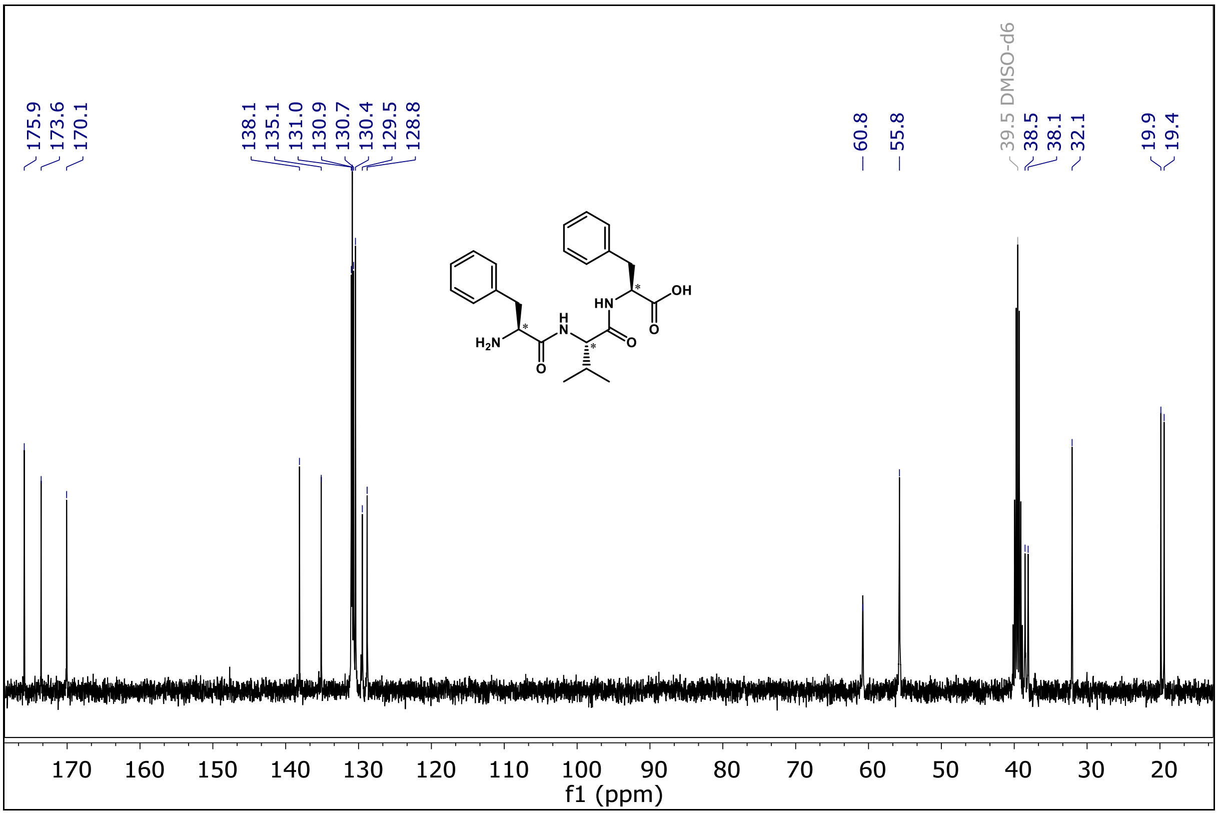

DEPT 135 (101 MHz, D_2O-DMSO, 5% DCl)

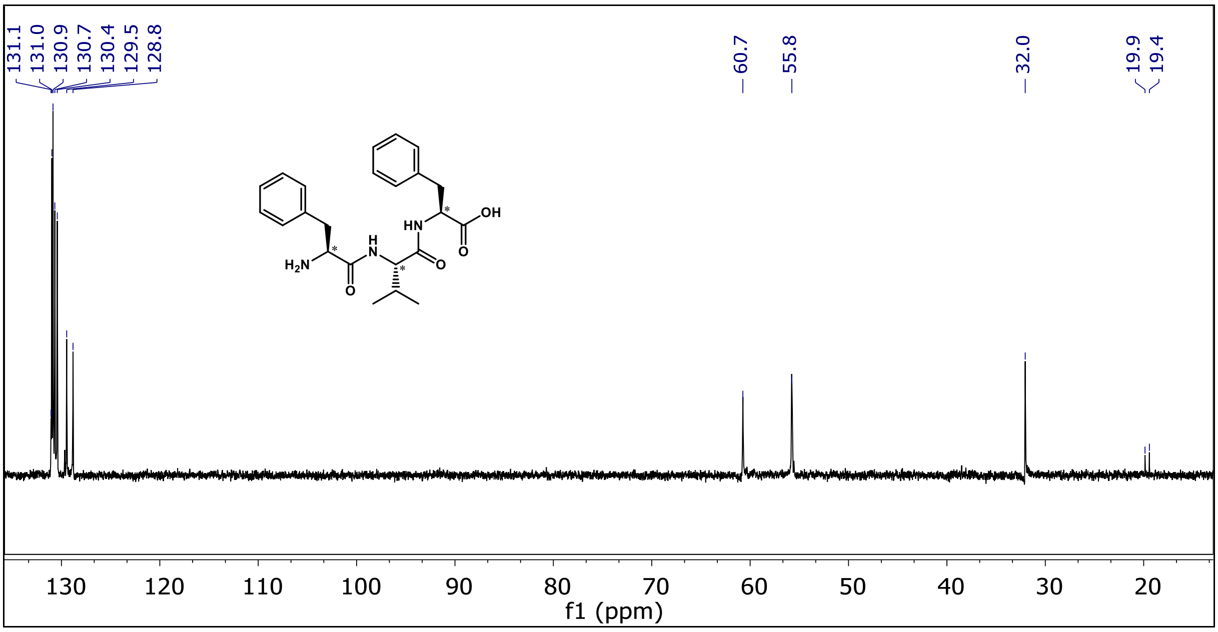

HMRS (ESI)

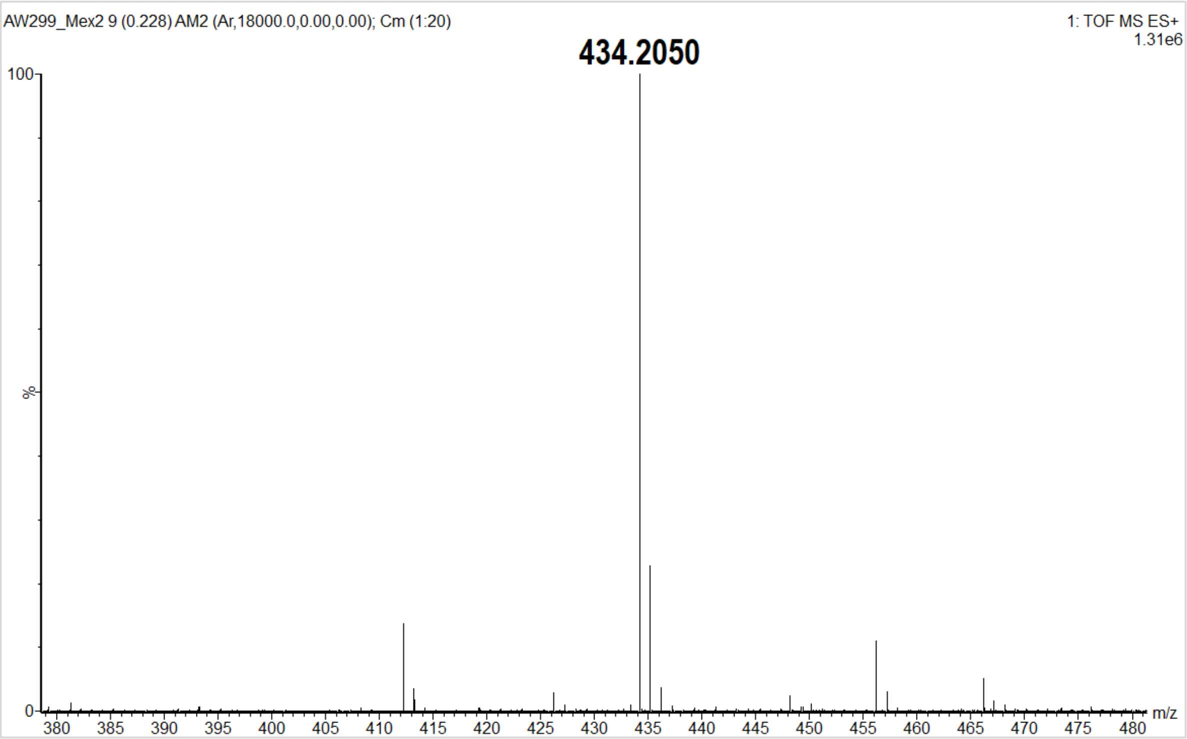

tert-butyl(S)-(1-((4-ethylphenyl)amino)-1-oxo-3-phenylpropan-2-yl)carbamate

(P19L)

^{1}H NMR (400 MHz, MeOD)

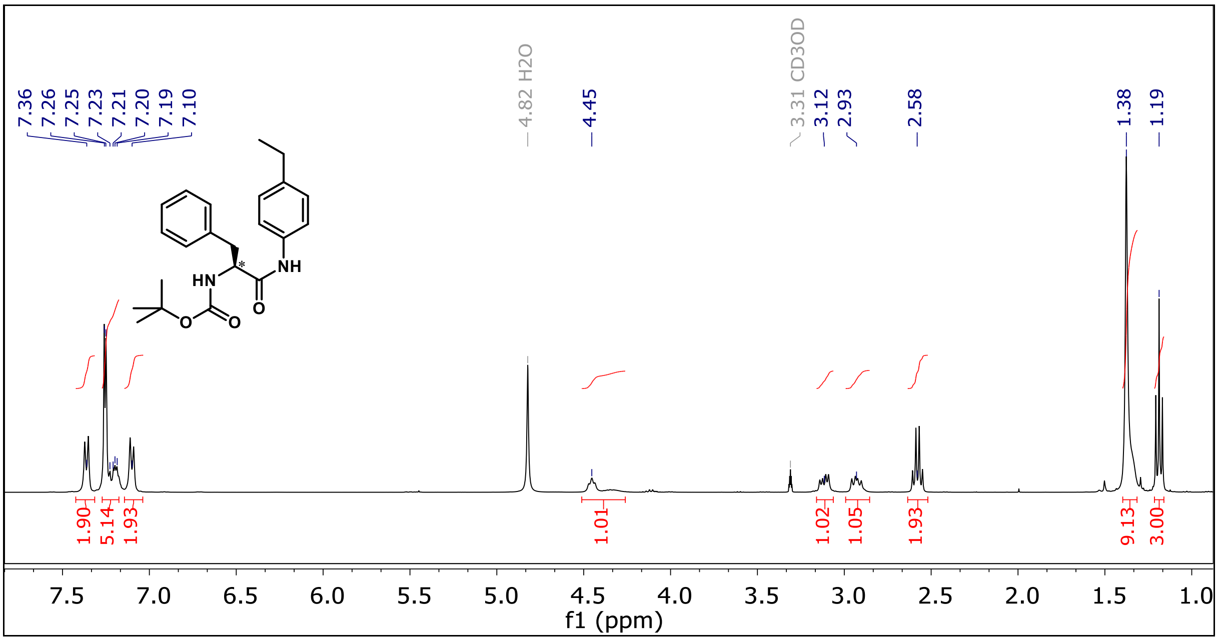

^{13}C NMR (101 MHz, MeOD)

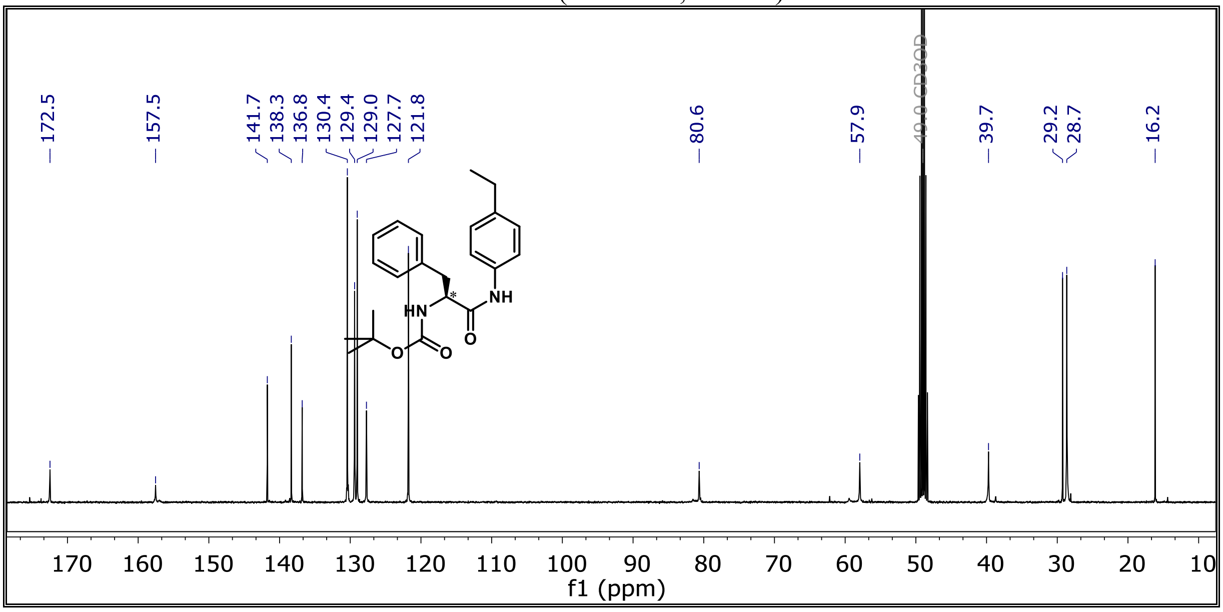

DEPT 135 (101 MHz, MeOD)

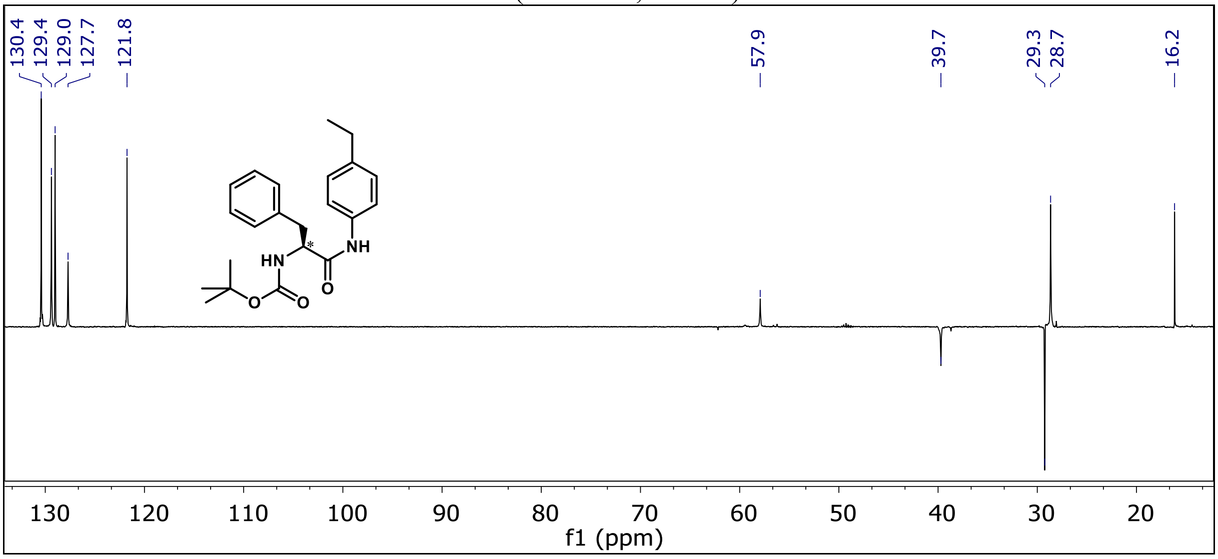

HRMS (ESI)

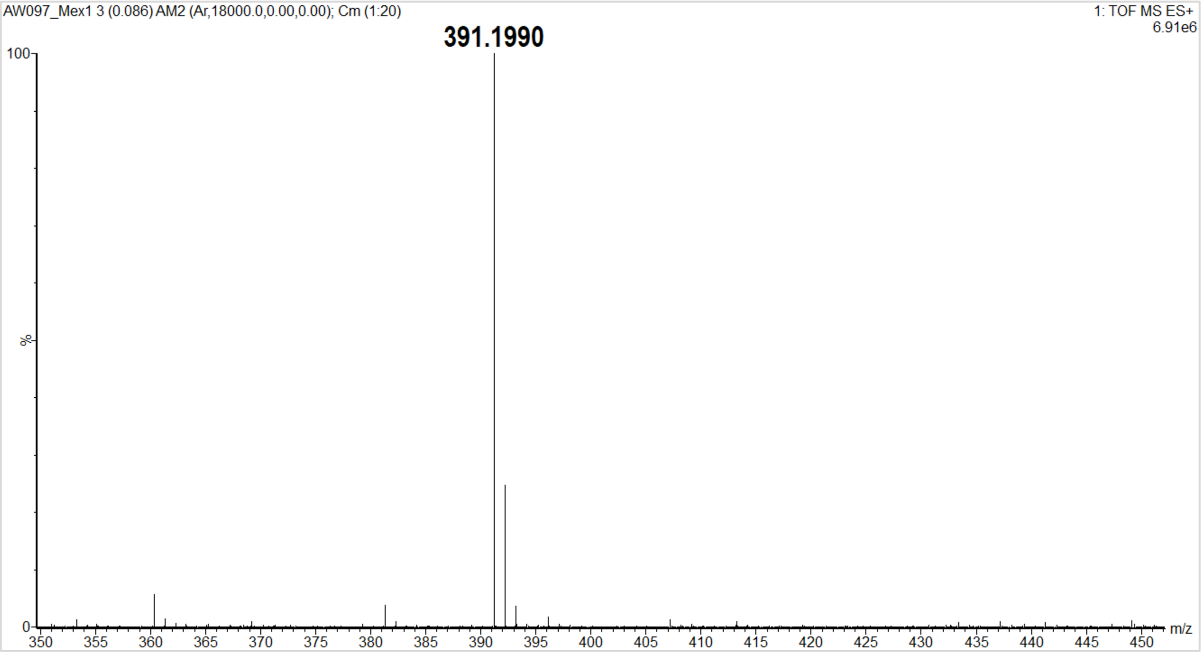

(S)-1-((4-ethylphenyl)amino)-1-oxo-3-phenylpropan-2-aminium trifluoroacetate

(P20L)

^{1}H NMR (400 MHz, MeOD)

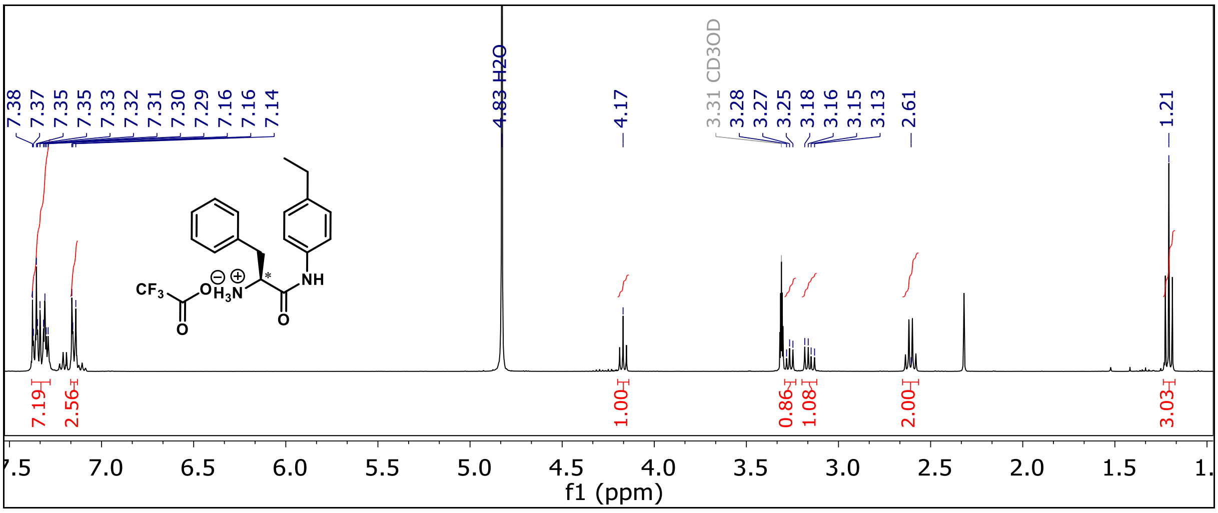

^{13}C NMR (101 MHz, MeOD)

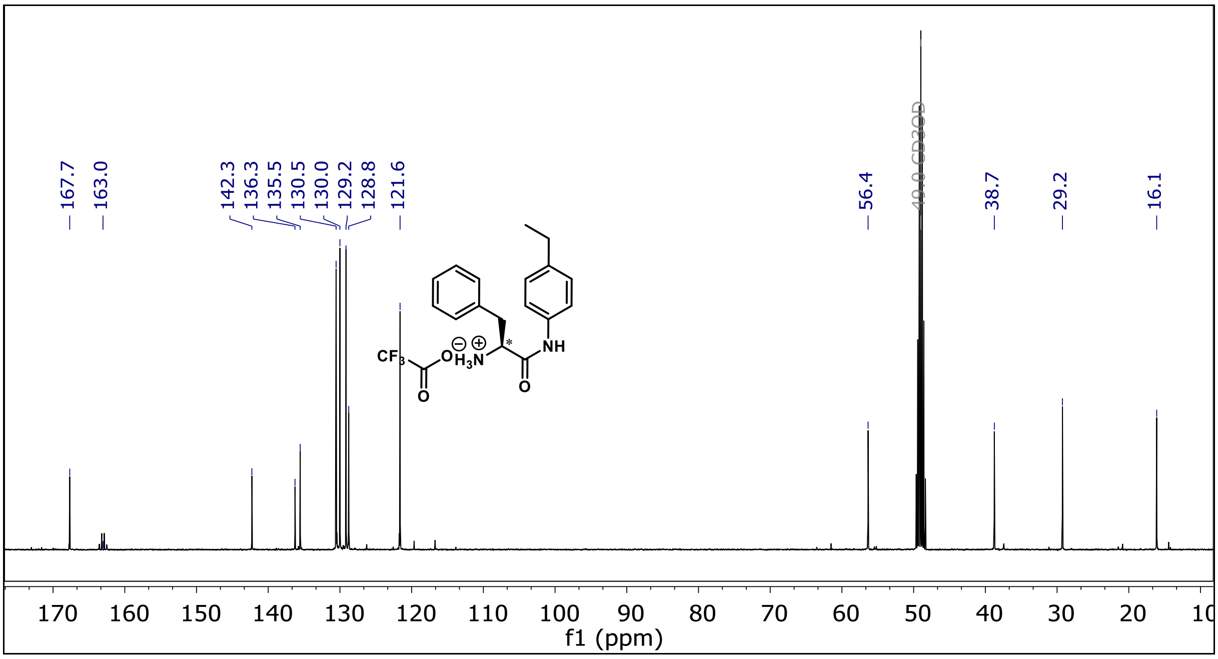

^{19}F NMR (376 MHz, MeOD)

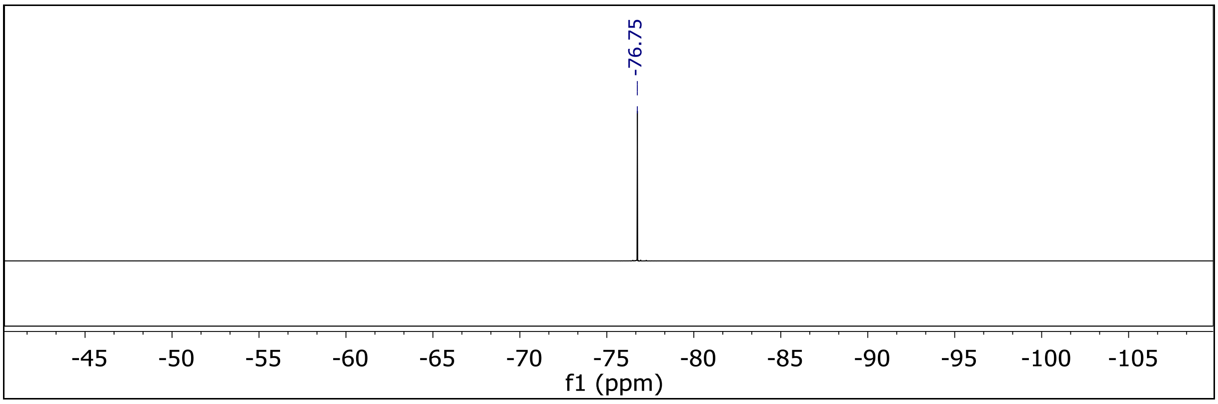

DEPT 135 (101 MHz, MeOD)

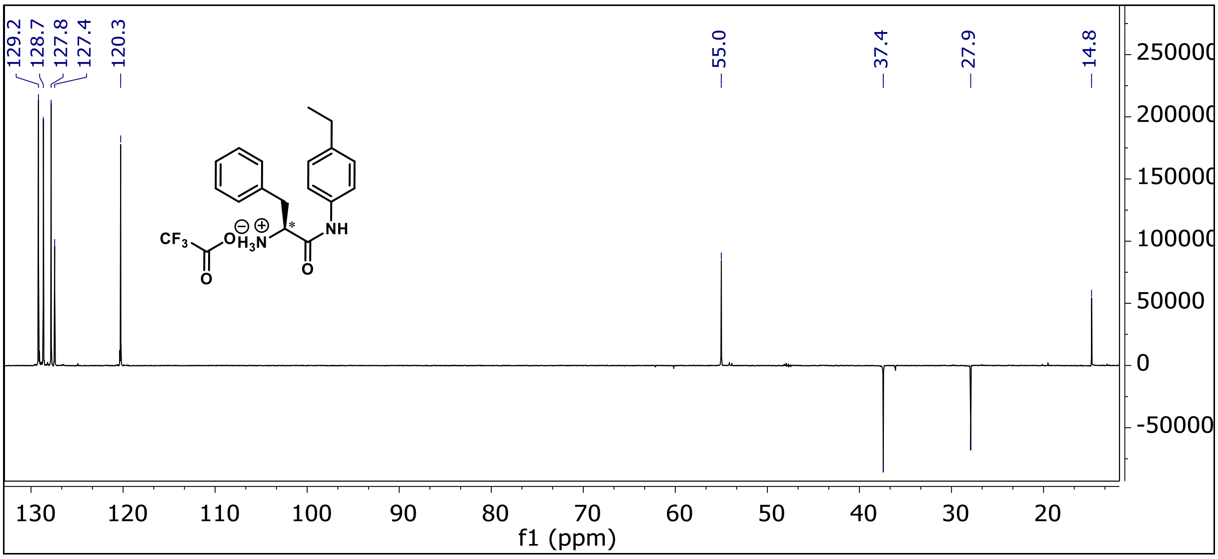

HRMS (ESI)

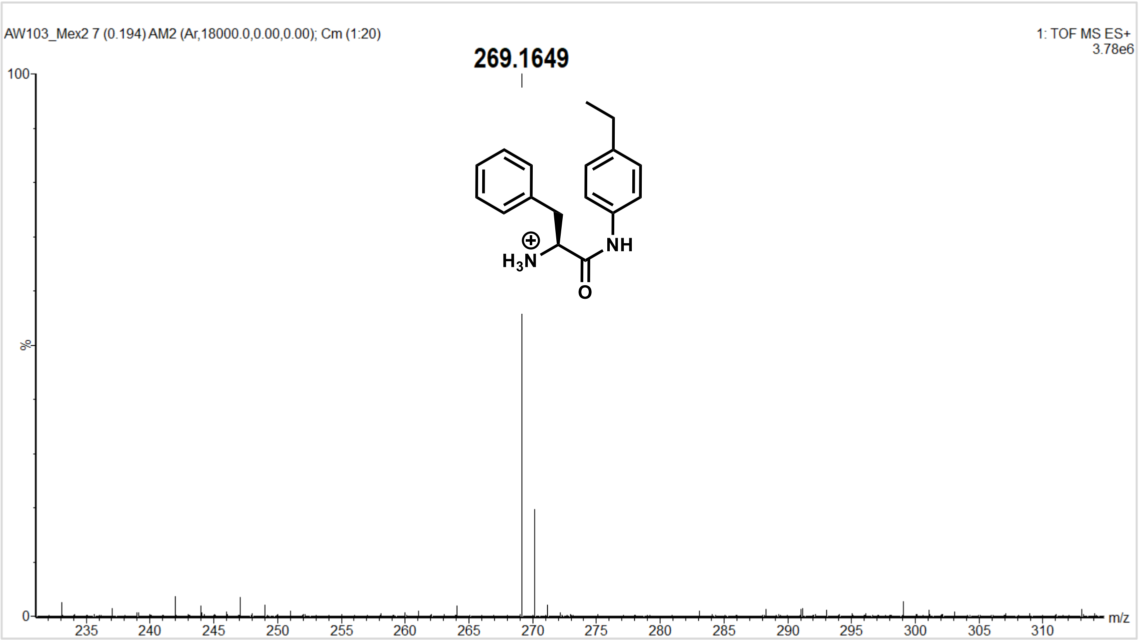

tert-butyl ((S)-1-(((S)-1-(((S)-1-((4-ethylphenyl)amino)-1-oxo-3-phenylpropan-2-yl)amino)-3-methyl-1-oxobutan-2-yl)amino)-1-oxo-3-phenylpropan-2-yl)carbamate

(P21L)

^{1}H NMR (400 MHz, $CDCl_3$)

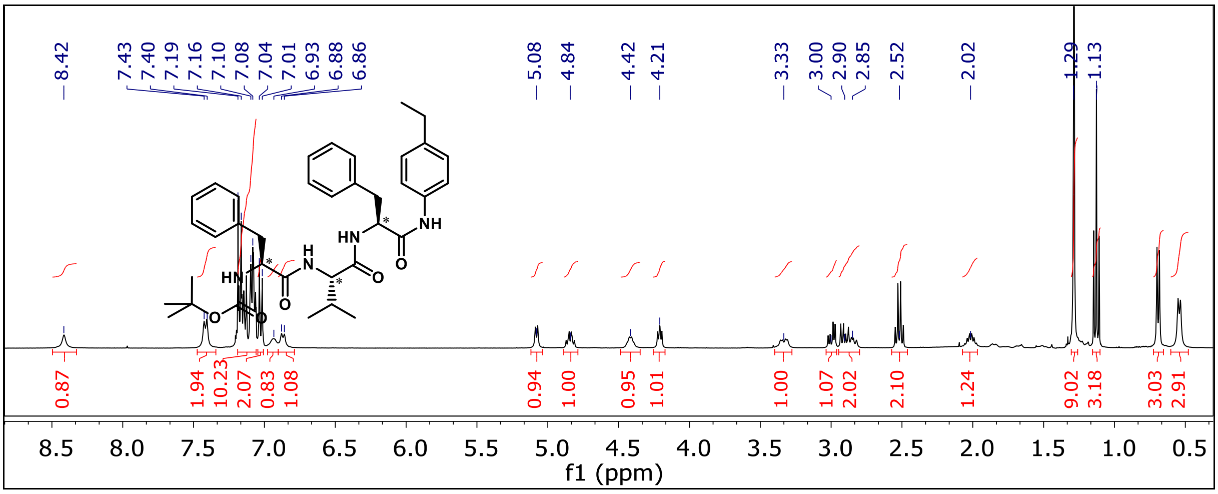

^{13}C NMR (101 MHz, $CDCl_3$)

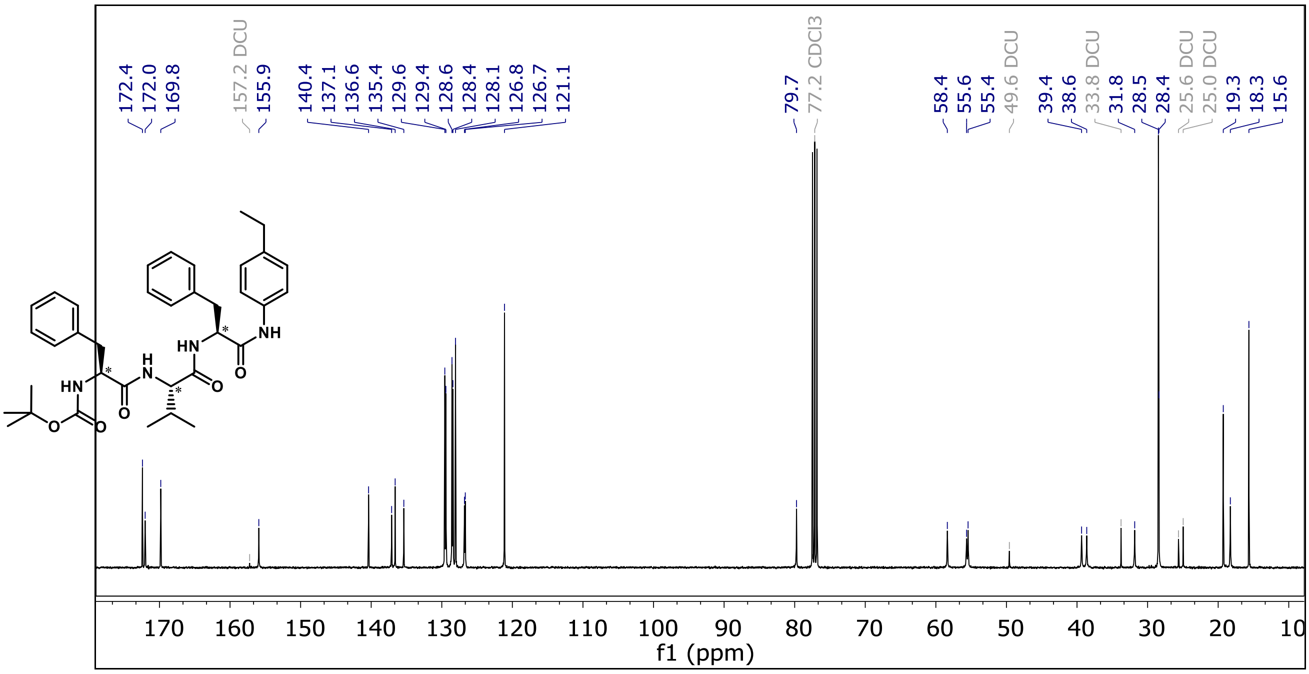

DEPT 135 (101 MHz, $CDCl_3$)

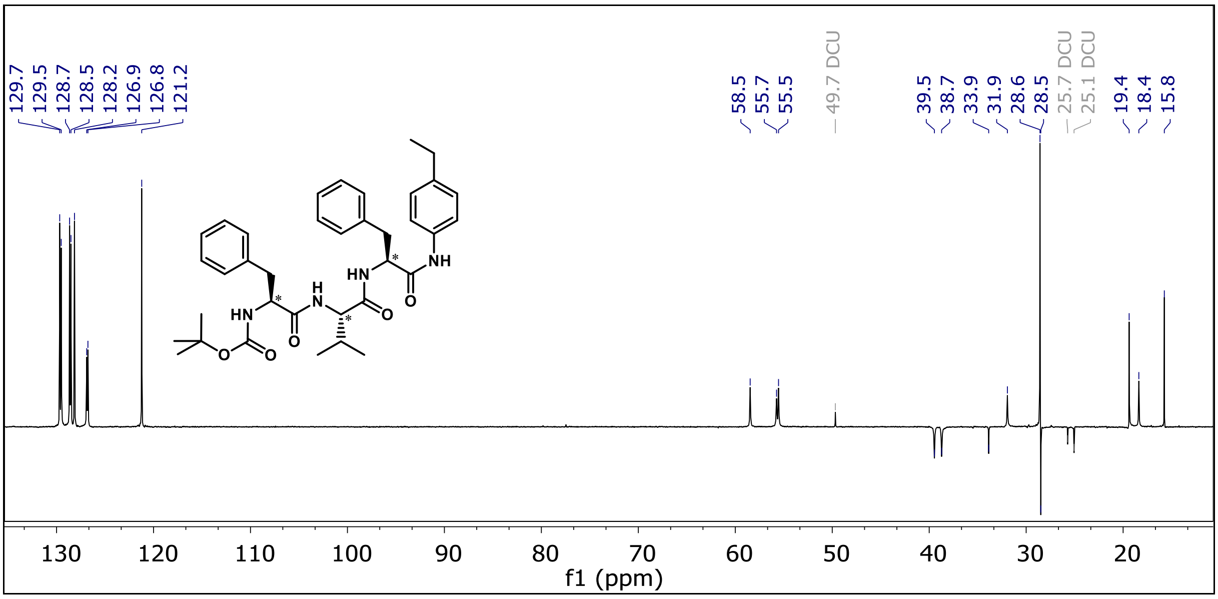

HRMS (ESI)

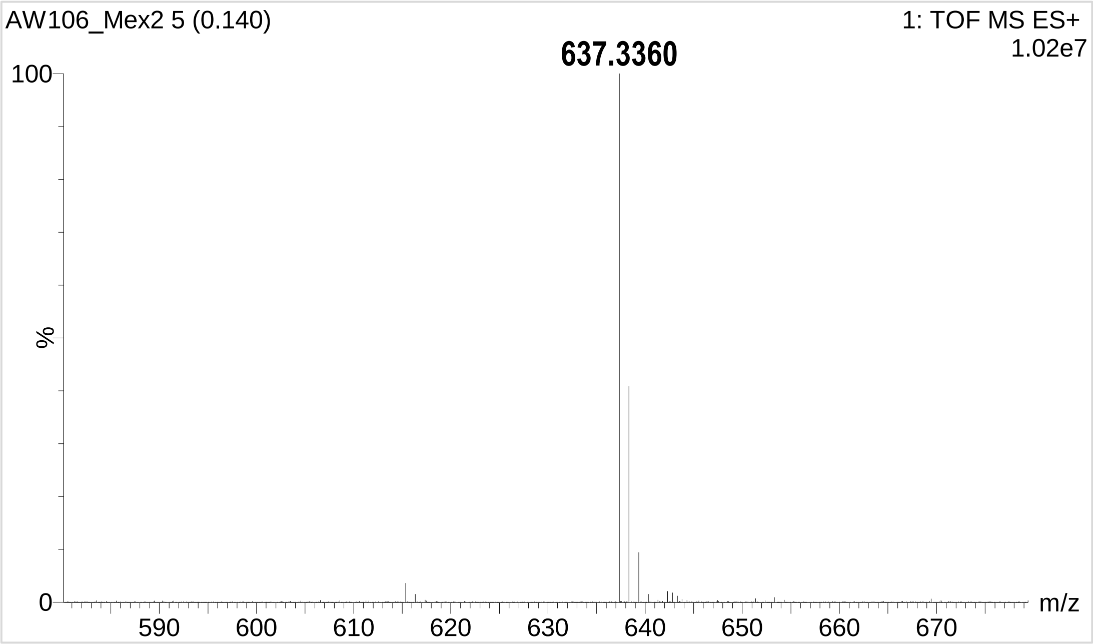

(S)-1-(((S)-1-(((S)-1-((4-ethylphenyl)amino)-1-oxo-3-phenylpropan-2-yl)amino)-3-methyl-1-oxobutan-2-yl)amino)-1-oxo-3-phenylpropan-2-aminium trifluoroacetate

(P22L)

^{1}H NMR (400 MHz, MeOD)

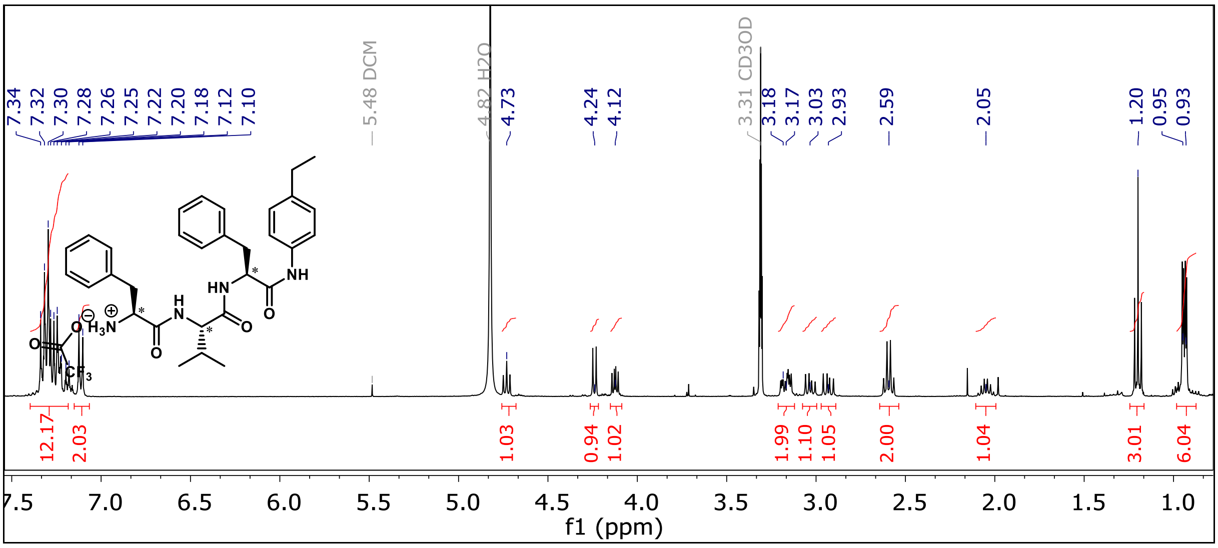

^{13}C NMR (101 MHz, MeOD-$CDCl_3$ (1 : 1 ; v : v))

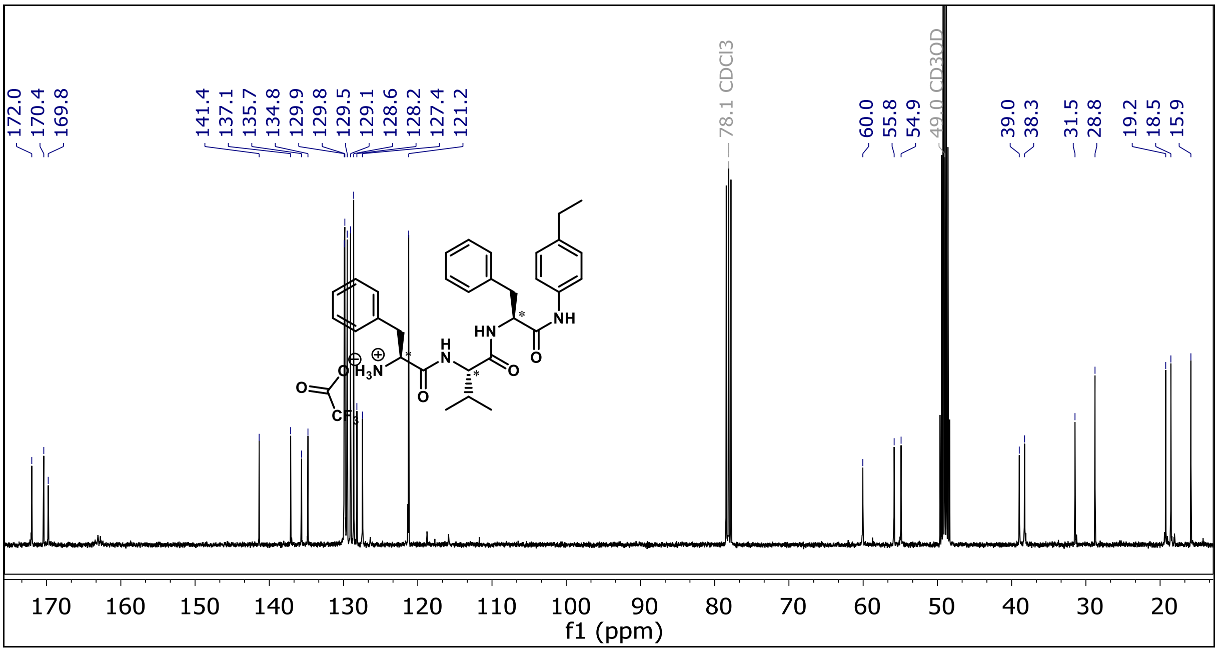

^{19}F NMR (376 MHz, MeOD)

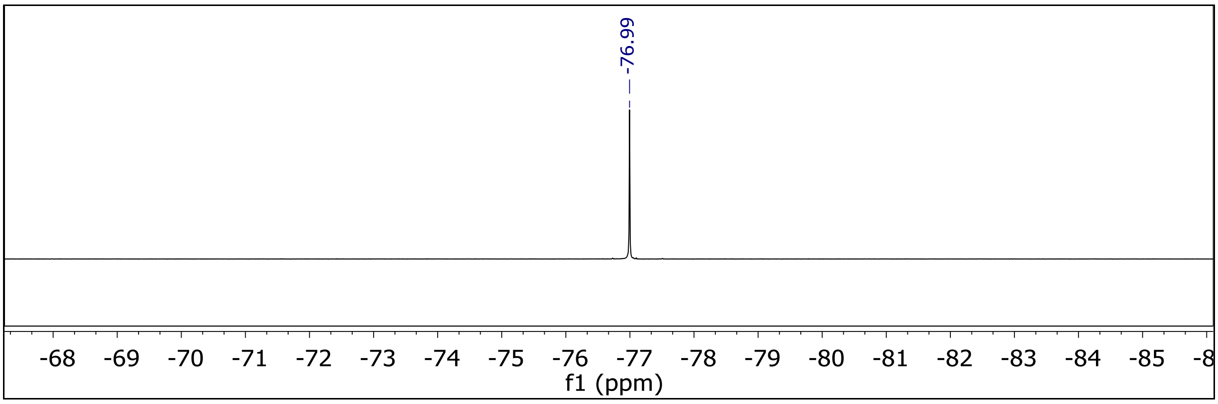

DEPT 135 (101 MHz, MeOD-$CDCl_3$ (1 : 1 ; v : v))

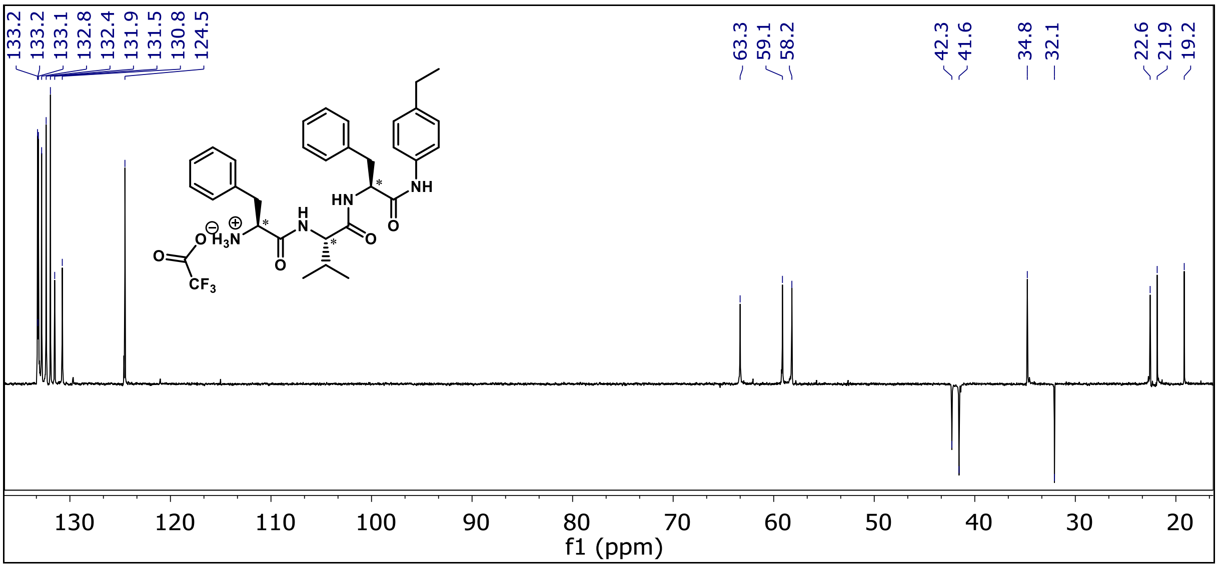

HRMS (ESI)

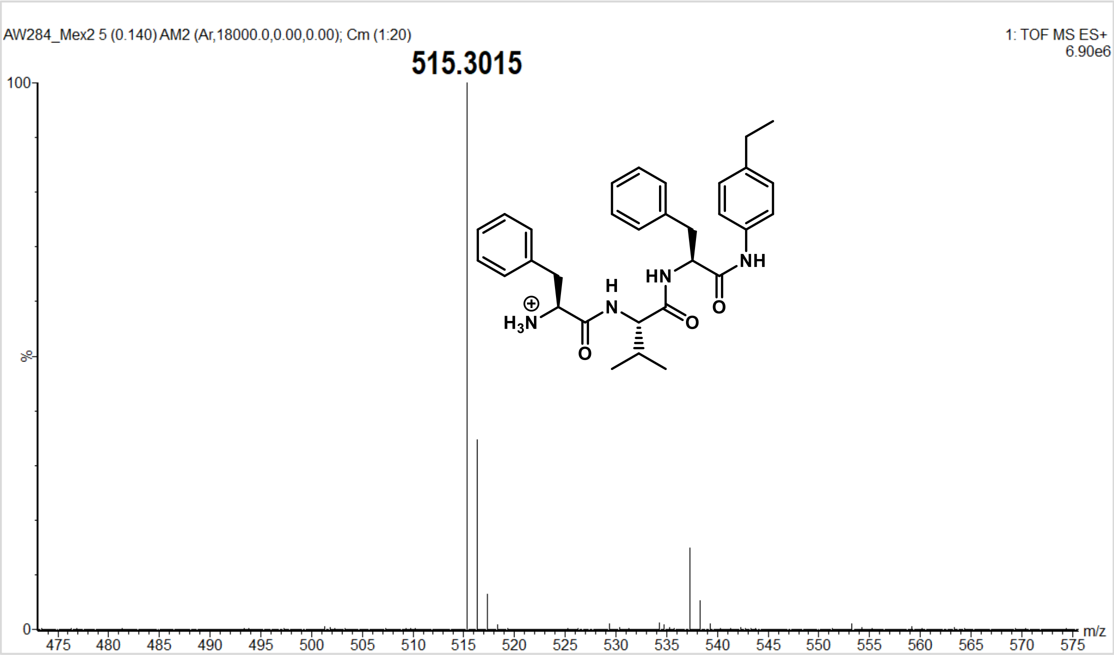

benzyl 4-(((S)-1-(((S)-1-(((S)-1-((4-ethylphenyl)amino)-1-oxo-3-phenylpropan-2-yl)amino)-3-methyl-1-oxobutan-2-yl)amino)-1-oxo-3-phenylpropan-2-yl)amino))-4-oxobutanoate

(P23L)

^{1}H NMR (400 MHz, MeOD)

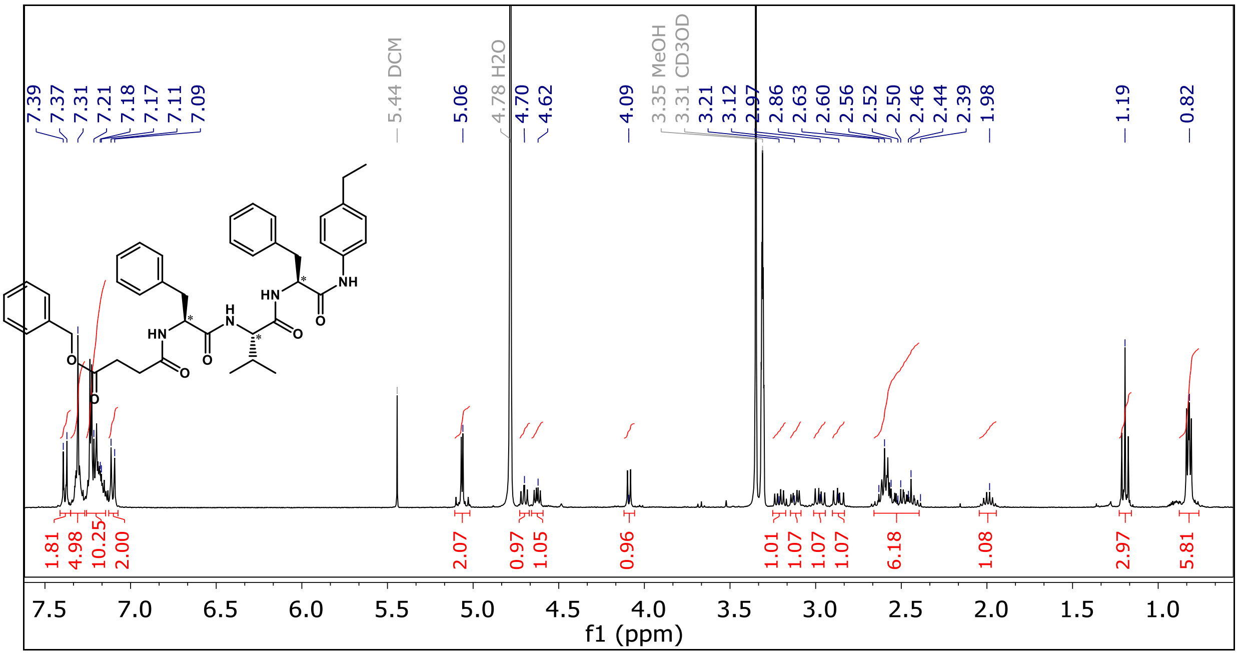

^{13}C NMR (101 MHz, $CDCl_3$)

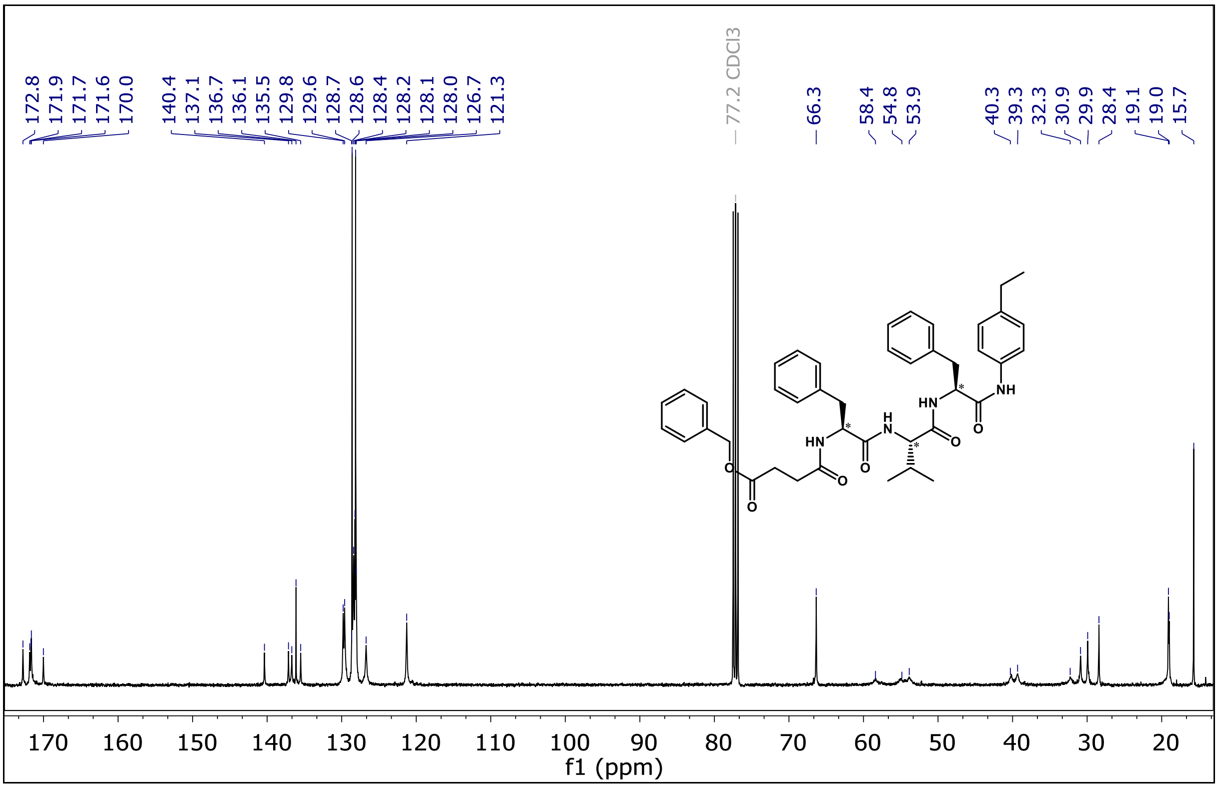

DEPT 135 (101 MHz, $CDCl_3$)

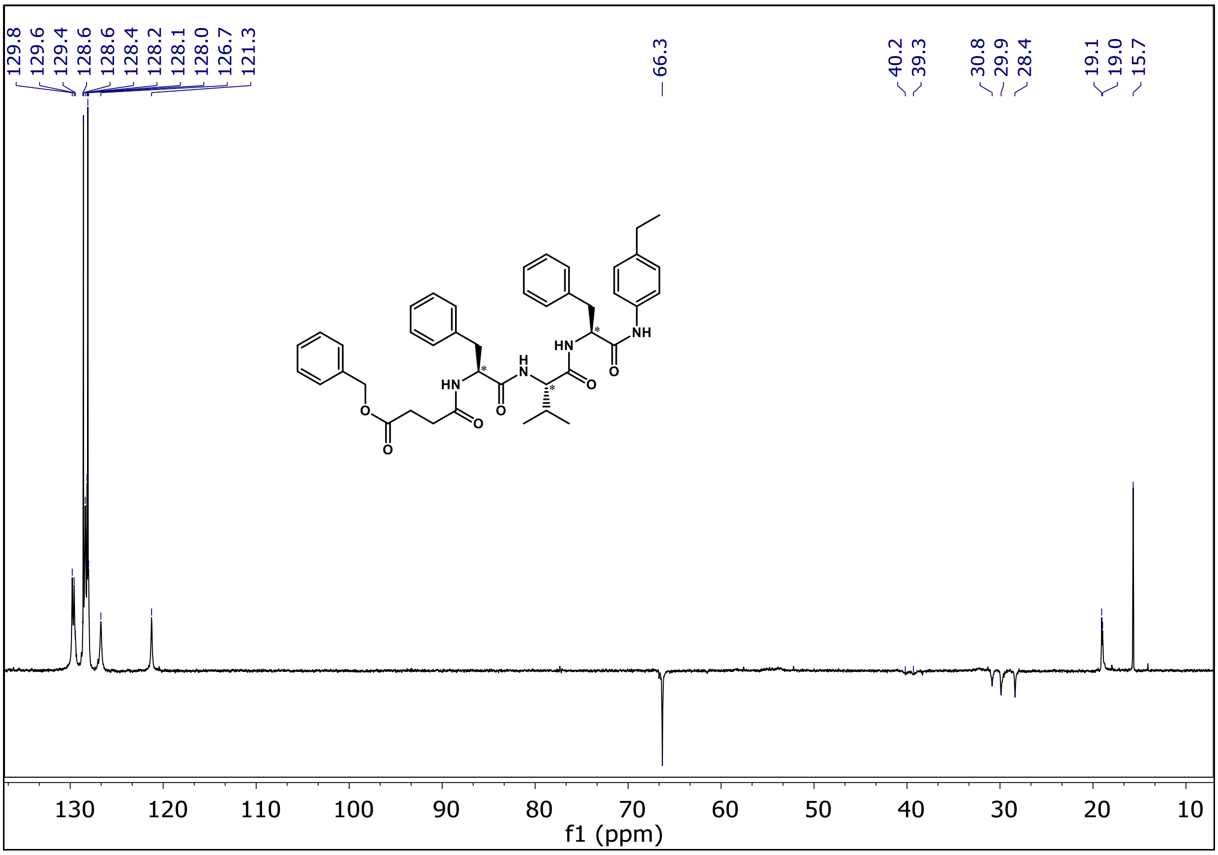

HRMS (ESI)

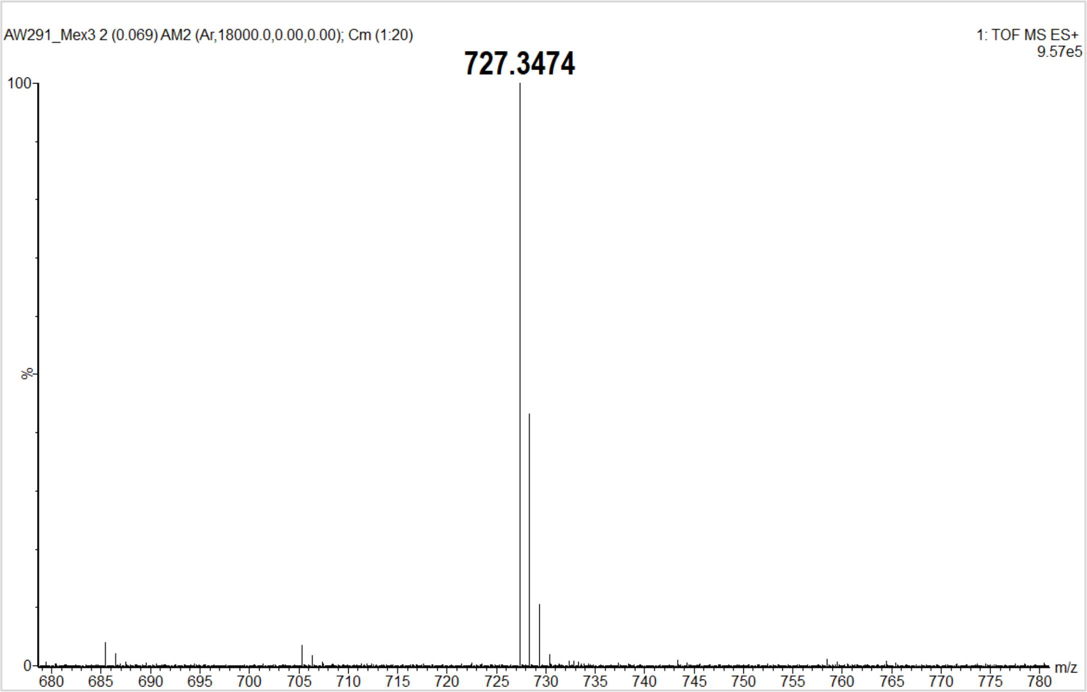
AW291_Mex3 2 (0.069) AM2 (Ar,18000.0,0.00,0.00); Cm (1:20)
1: TOF MS ES+
9.57e5
727.3474
100
%
0
680
685
690
695
700
705
710
715
720
725
730
735
740
745
750
755
760
765
770
775
780
m/z

4-(((S)-1-(((S)-1-(((S)-1-((4-ethylphenyl)amino)-1-oxo-3-phenylpropan-2-yl)amino)-3-methyl-1-oxobutan-2-yl)amino)-1-oxo-3-phenylpropan-2-yl)amino)-4-oxobutanoic acid

(P24L)

^{1}H NMR (400 MHz, MeOD)

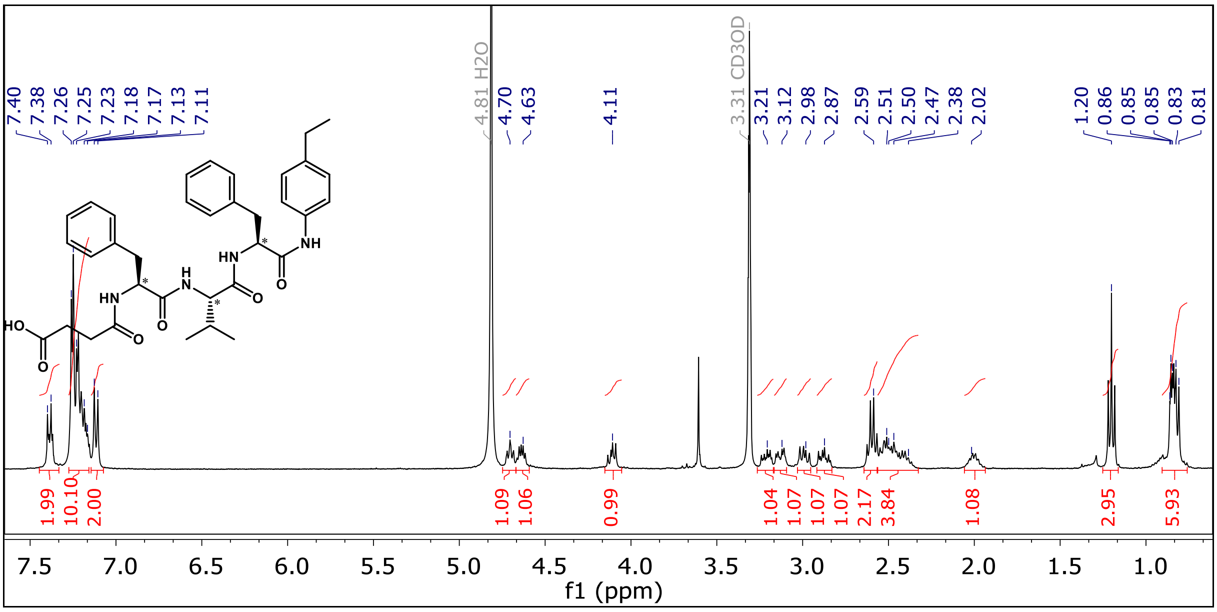

^{13}C NMR (101 MHz, MeOD-$CDCl_3$ (1 : 1 ; v : v))

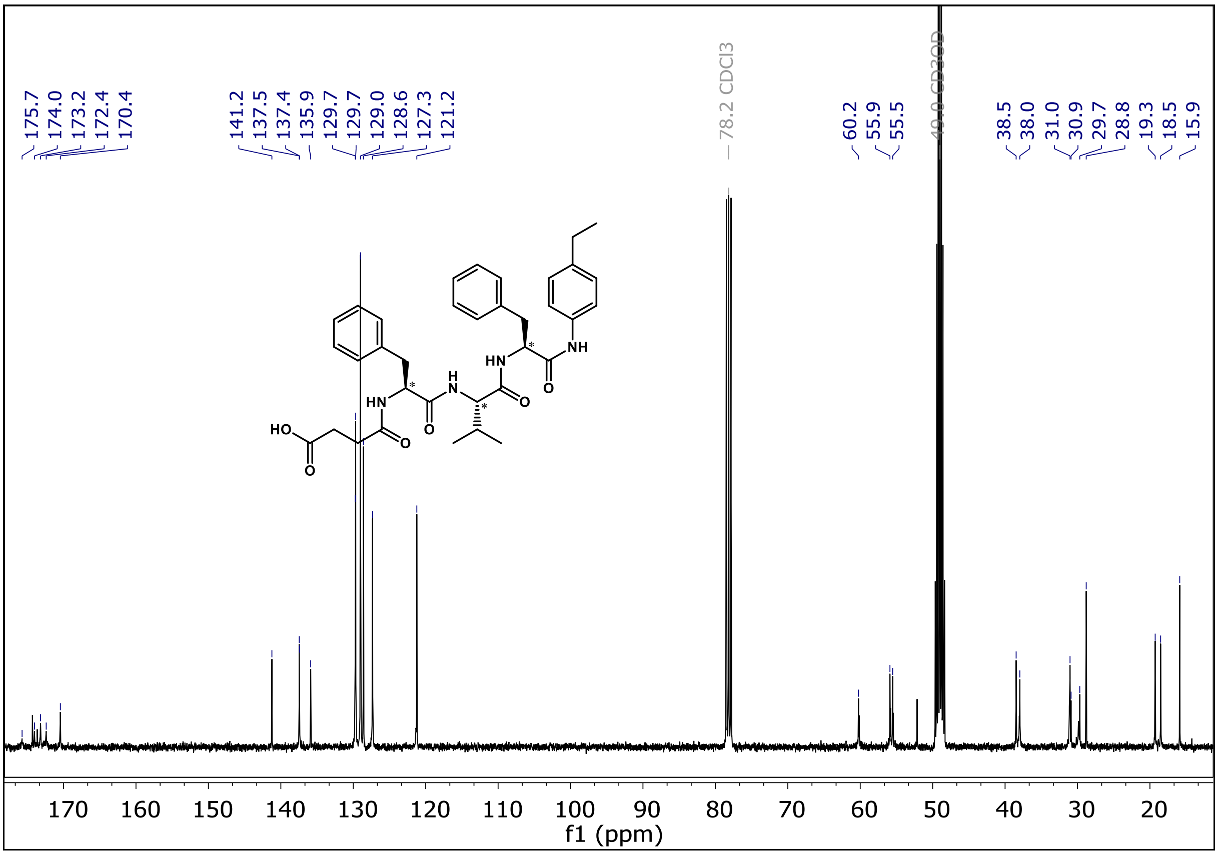

DEPT 135 (101 MHz, MeOD-$CDCl_3$ (1 : 1 ; v : v))

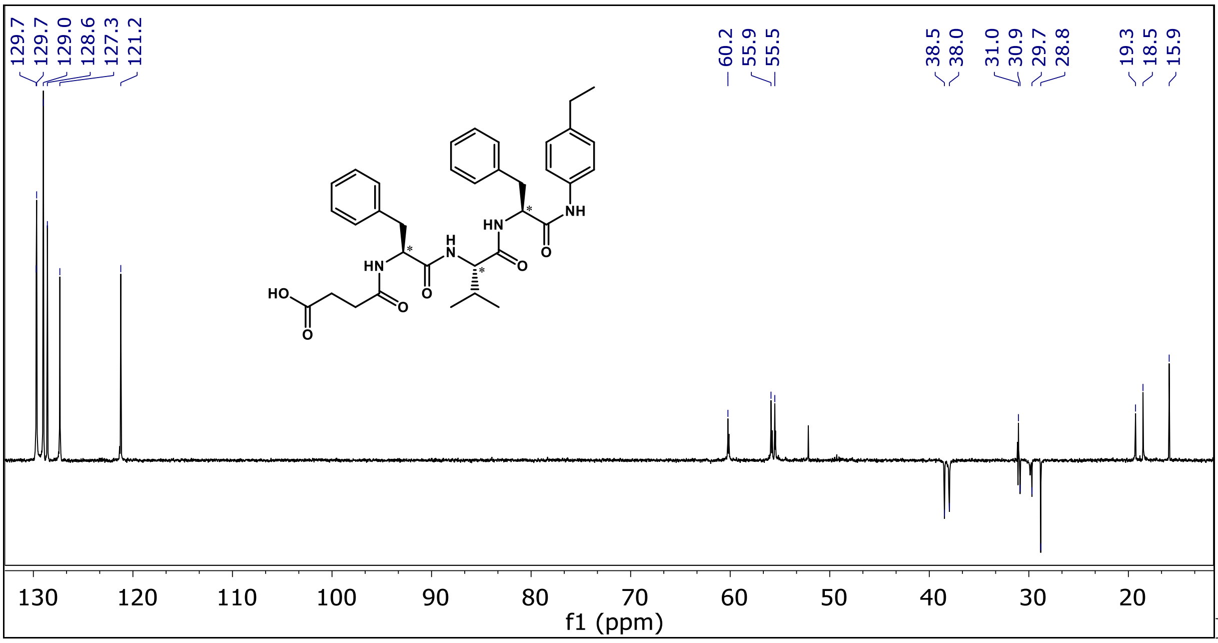
129.7
129.7
129.0
128.6
127.3
121.2
60.2
55.9
55.5
38.5
38.0
31.0
30.9
29.7
28.8
19.3
18.5
15.9
HO
O
HN
O
O
H
N
HN
O
O
NH
130
120
110
100
90
80
70
60
50
40
30
20
f1 (ppm)

HRMS (ESI)

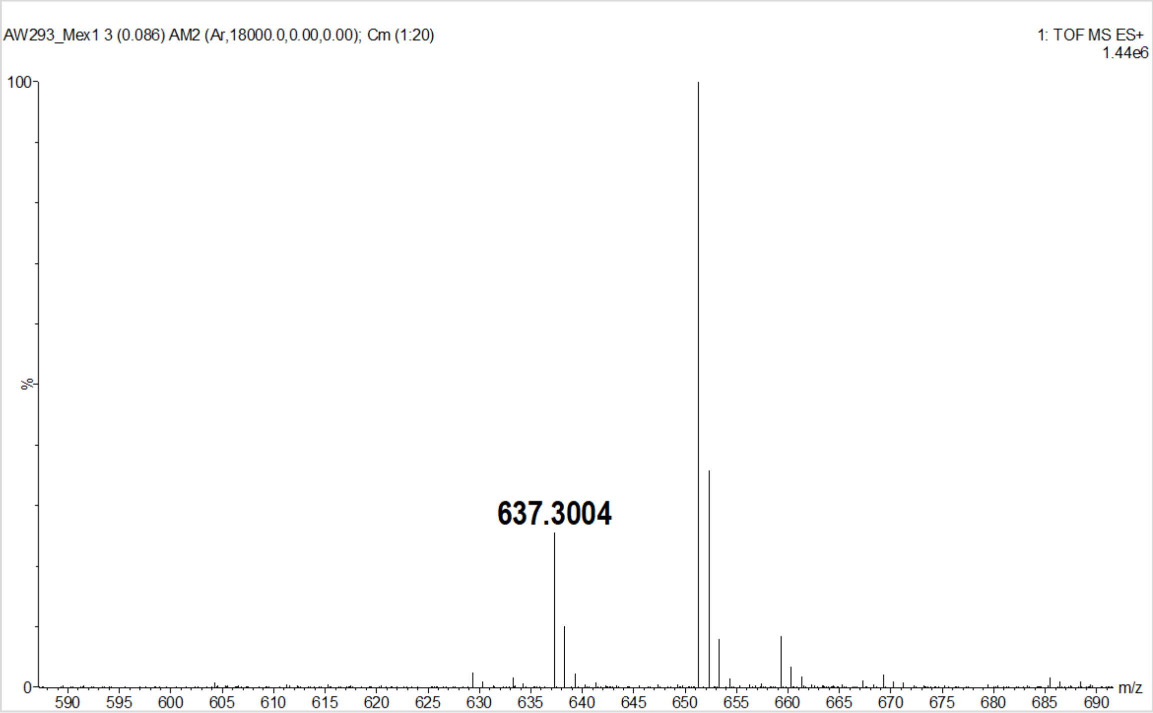
AW293_Mex1 3 (0.086) AM2 (Ar,18000.0,0.00,0.00); Cm (1:20)
1: TOF MS ES+
1.44e6
100
%
0
637.3004
590
595
600
605
610
615
620
625
630
635
640
645
650
655
660
665
670
675
680
685
690
m/z

(S)-2-((S)-2-amino-3-phenylpropanamido)-N-((S)-1-((4-ethylphenyl)amino)-1-oxo-3-phenylpropan-2-yl)-3-methylbutanamide

(P25L)

^{1}H NMR (300 MHz, MeOD)

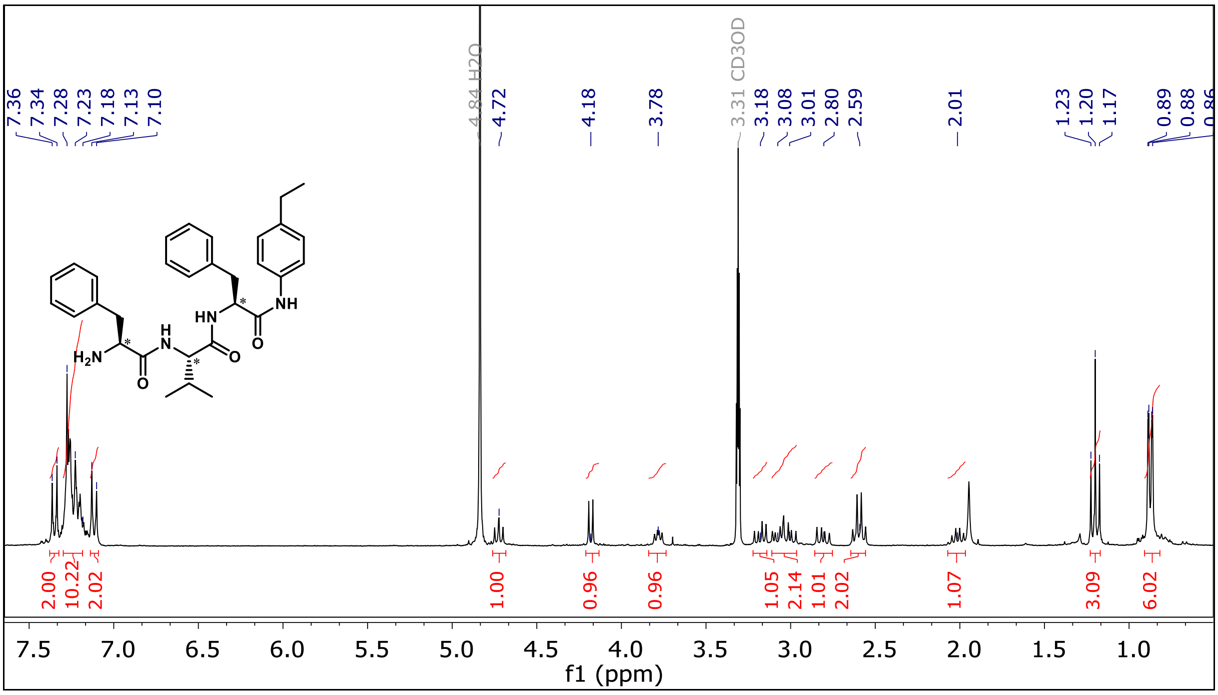

^{13}C NMR (101 MHz, MeOD-$CDCl_3$ (1 : 1 ; v : v))

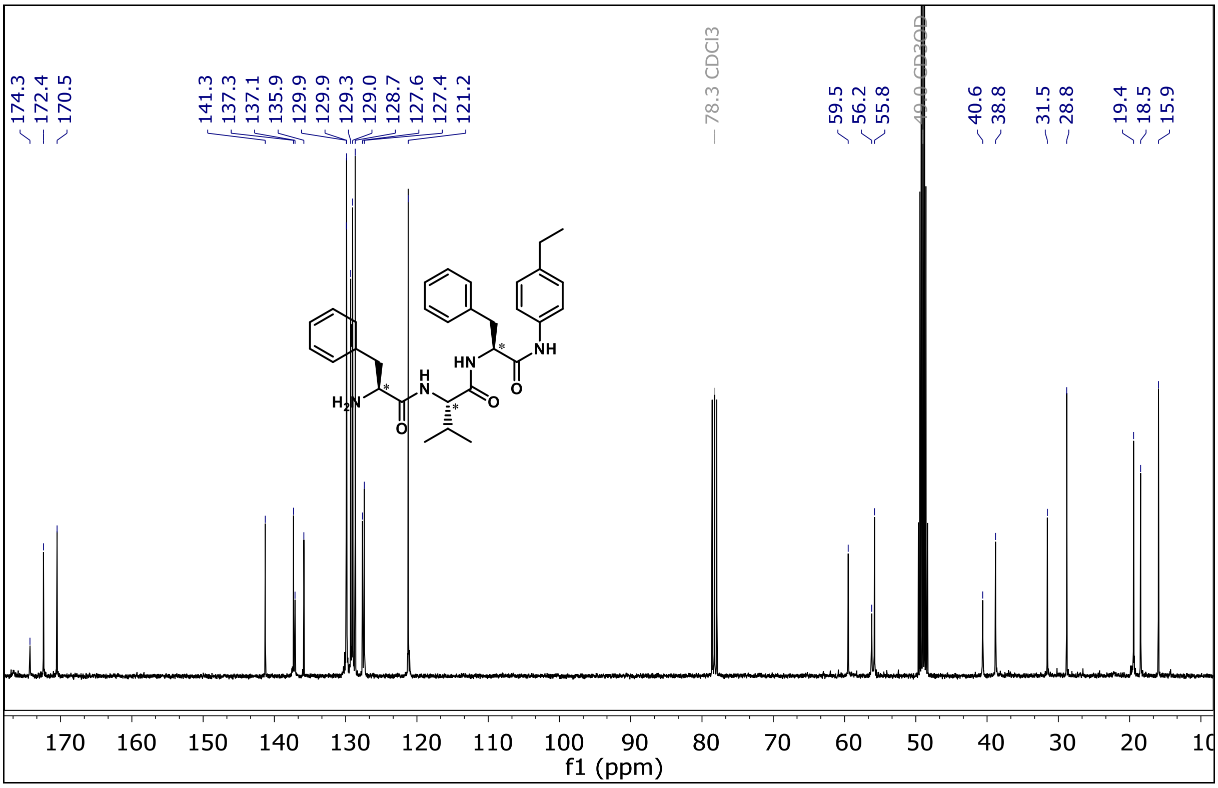

DEPT 135 (101 MHz, MeOD-CDCl3 (1 : 1 ; v : v))

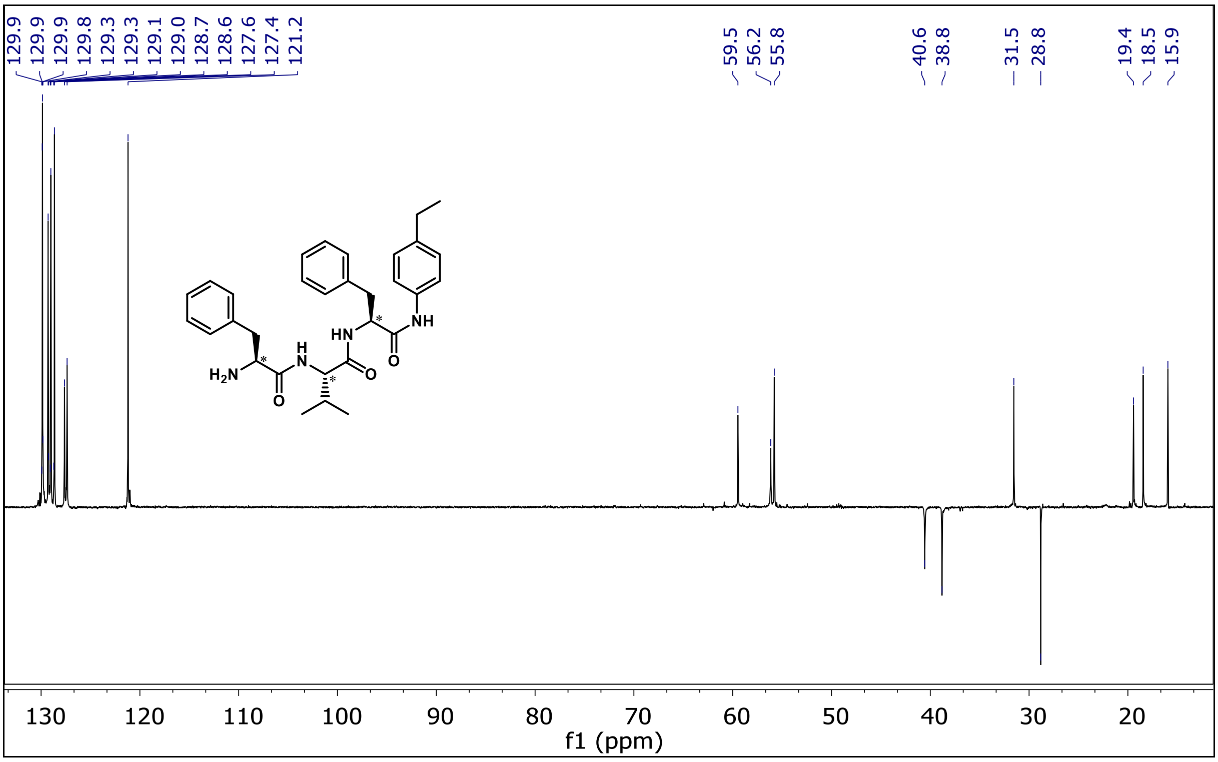

HRMS (ESI)

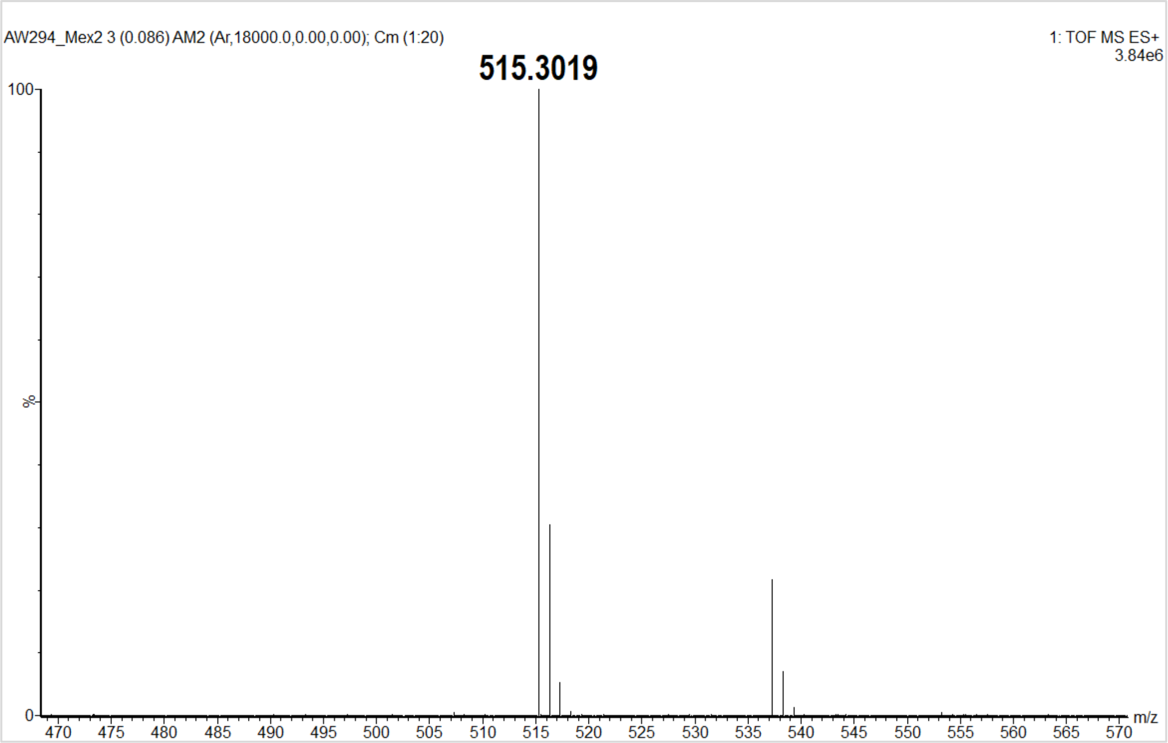

tert-butyl (R)-(1-oxo-3-phenyl-1-((4-vinylphenyl)amino)propan-2-yl)carbamate

(P3D)

^{1}H NMR (300 MHz, MeOD)

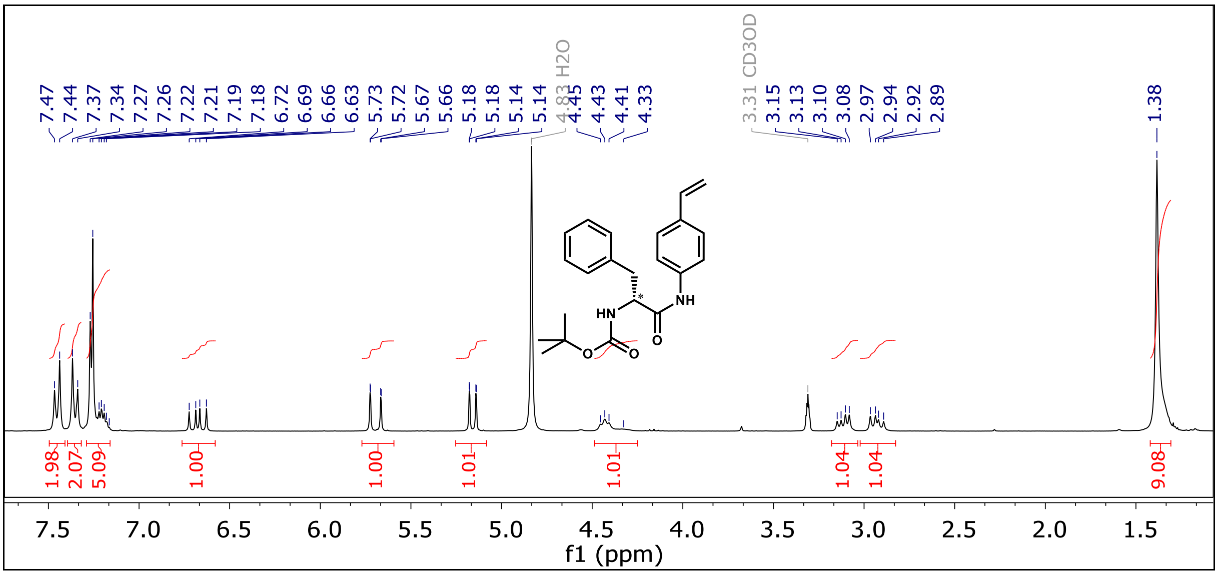

^{13}C NMR (75 MHz, $CDCl_3$)

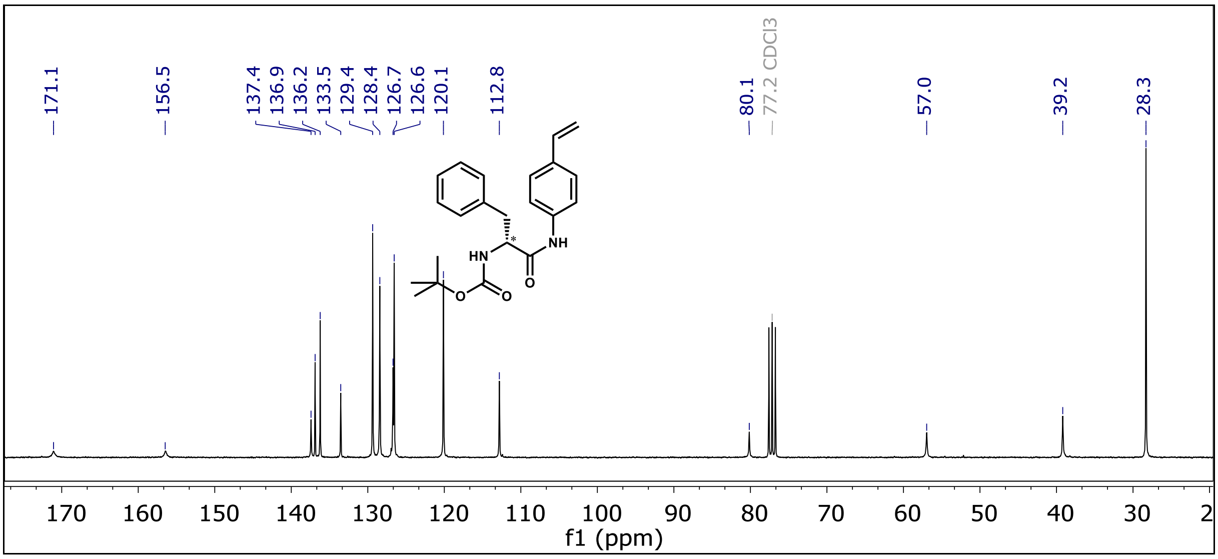

DEPT 135 (75 MHz, $CDCl_3$)

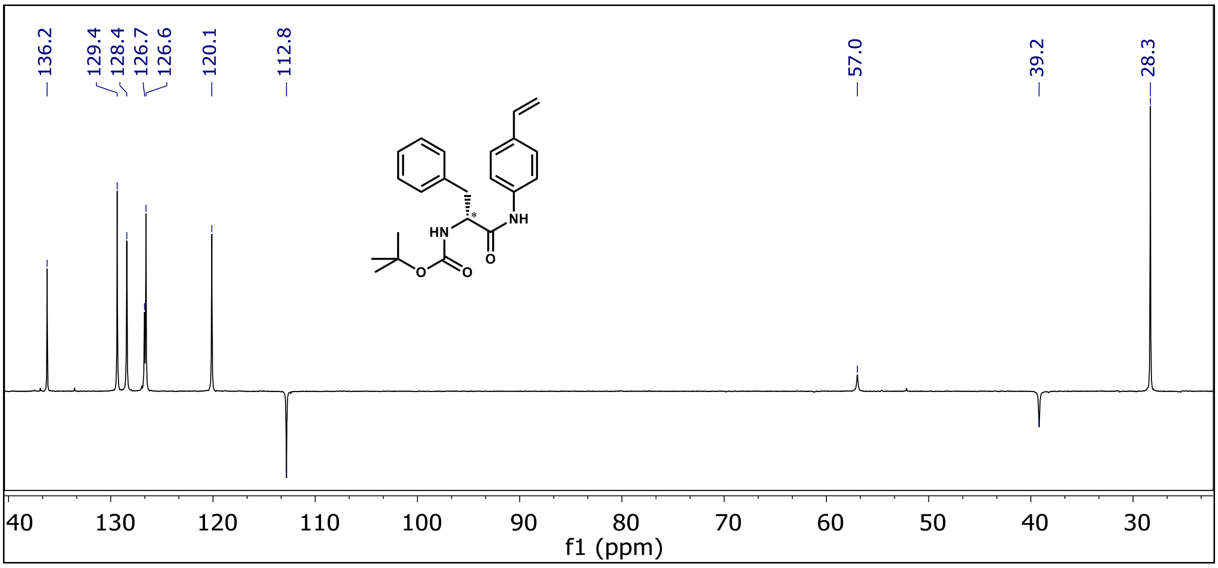
136.2
129.4
128.4
126.7
126.6
120.1
112.8
57.0
39.2
28.3
HN
NH
O
O
O
*
140
130
120
110
100
90
80
70
60
50
40
30
f1 (ppm)

HRMS (ESI)

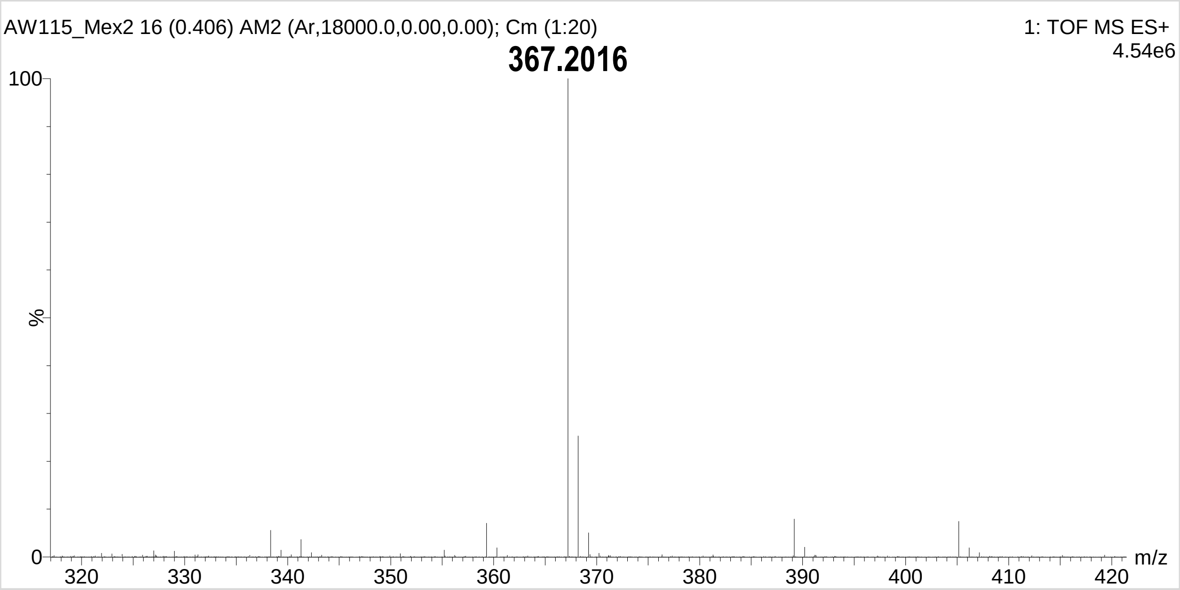
AW115_Mex2 16 (0.406) AM2 (Ar,18000.0,0.00,0.00); Cm (1:20)
1: TOF MS ES+
4.54e6
367.2016
100
%
0
320
330
340
350
360
370
380
390
400
410
420
m/z

benzyl (tert-butoxycarbonyl)-D-phenylalanyl-D-valinate

(P4D)

^{1}H NMR (300 MHz, $CDCl_3$)

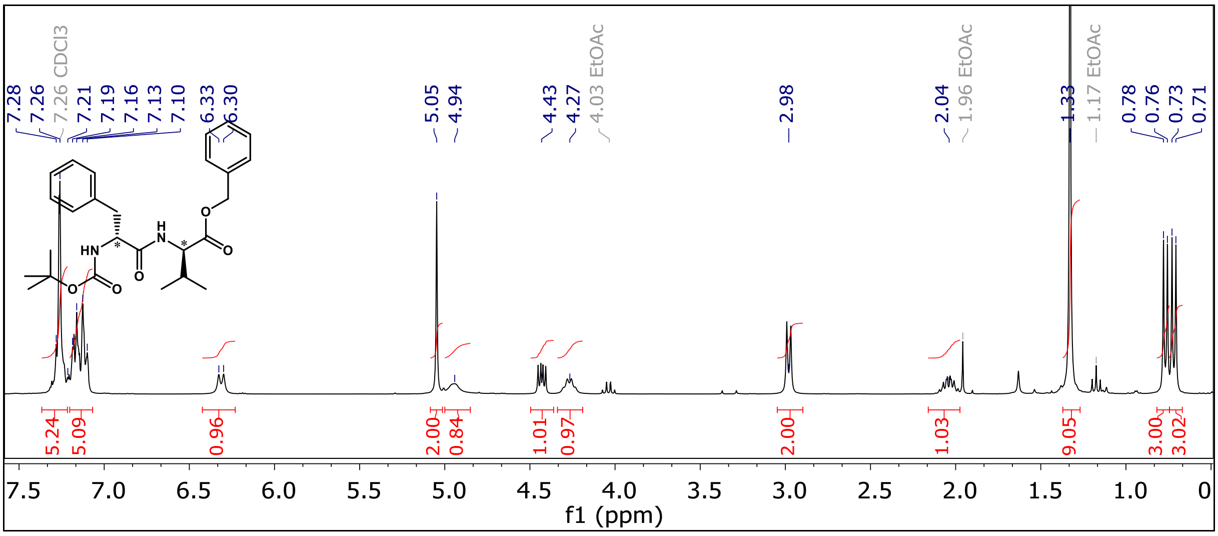

(tert-butoxycarbonyl)-D-phenylalanyl-D-valine

(P5D)

^{1}H NMR (300 MHz, $CDCl_3$)

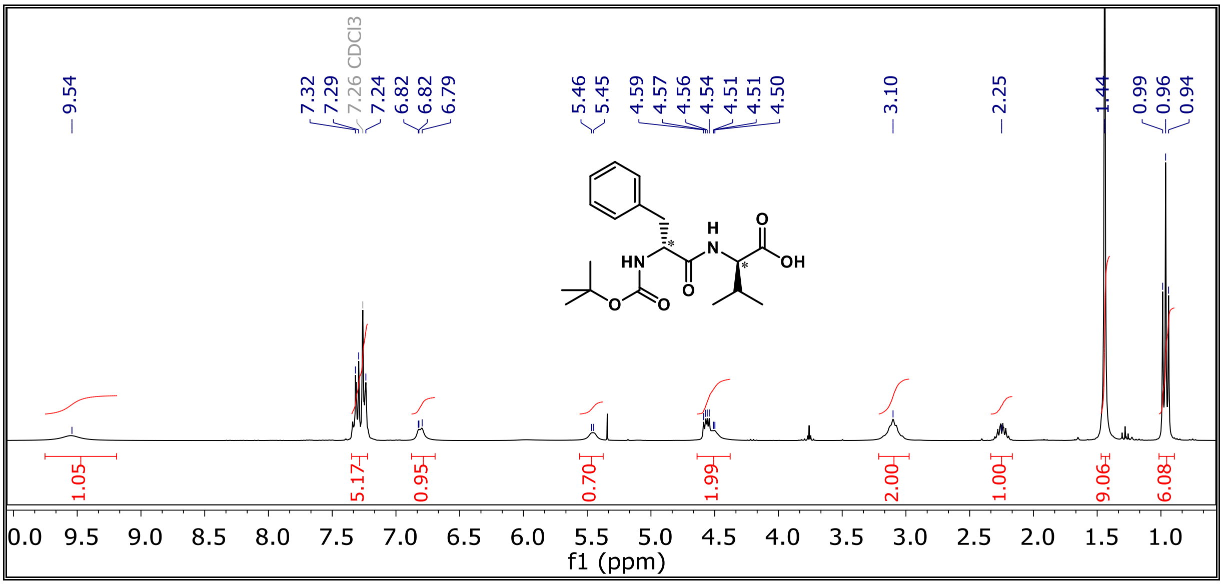

^{13}C NMR (75 MHz, $CDCl_3$)

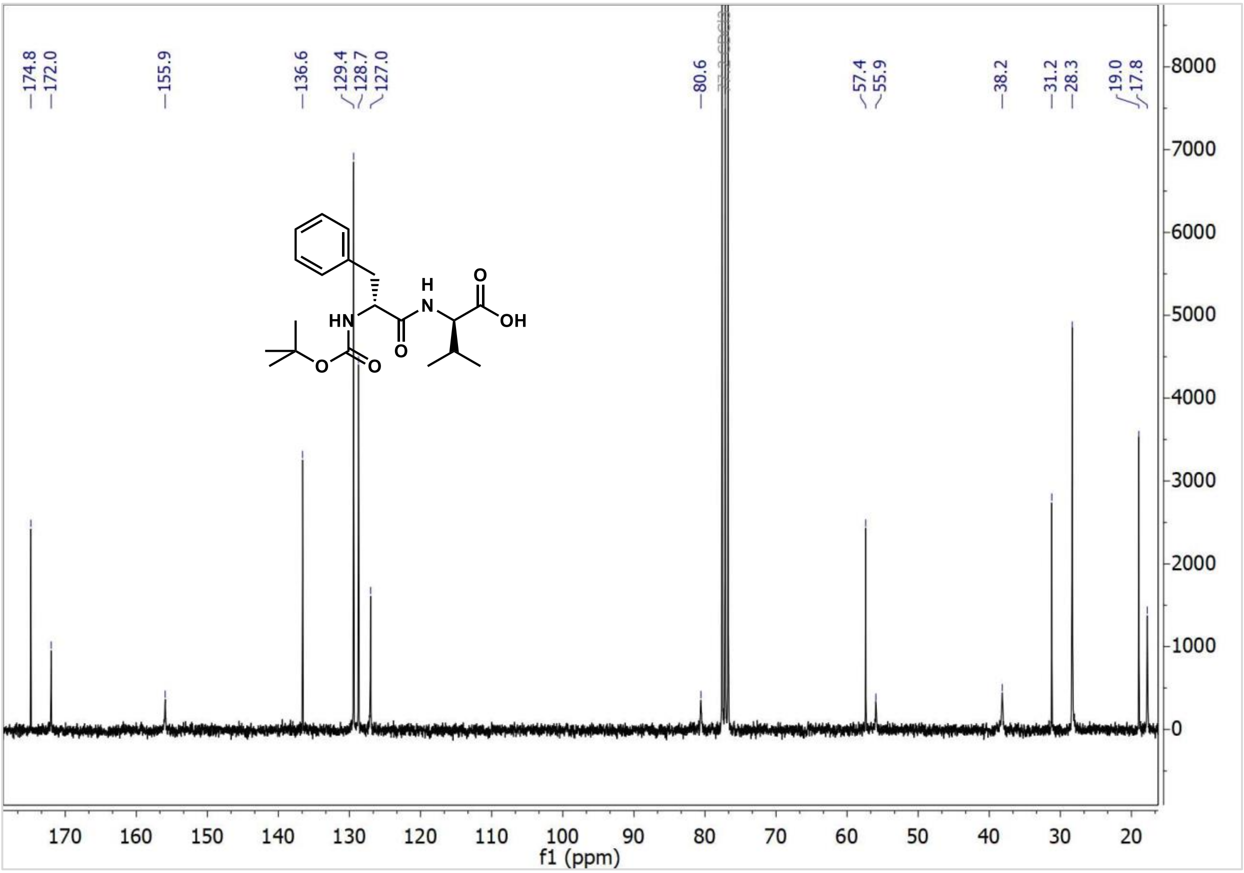

benzyl (tert-butoxycarbonyl)-D-phenylalanyl-D-valyl-D-phenylalaninate

(P6D)

^{1}H NMR (400 MHz, $CDCl_3$)

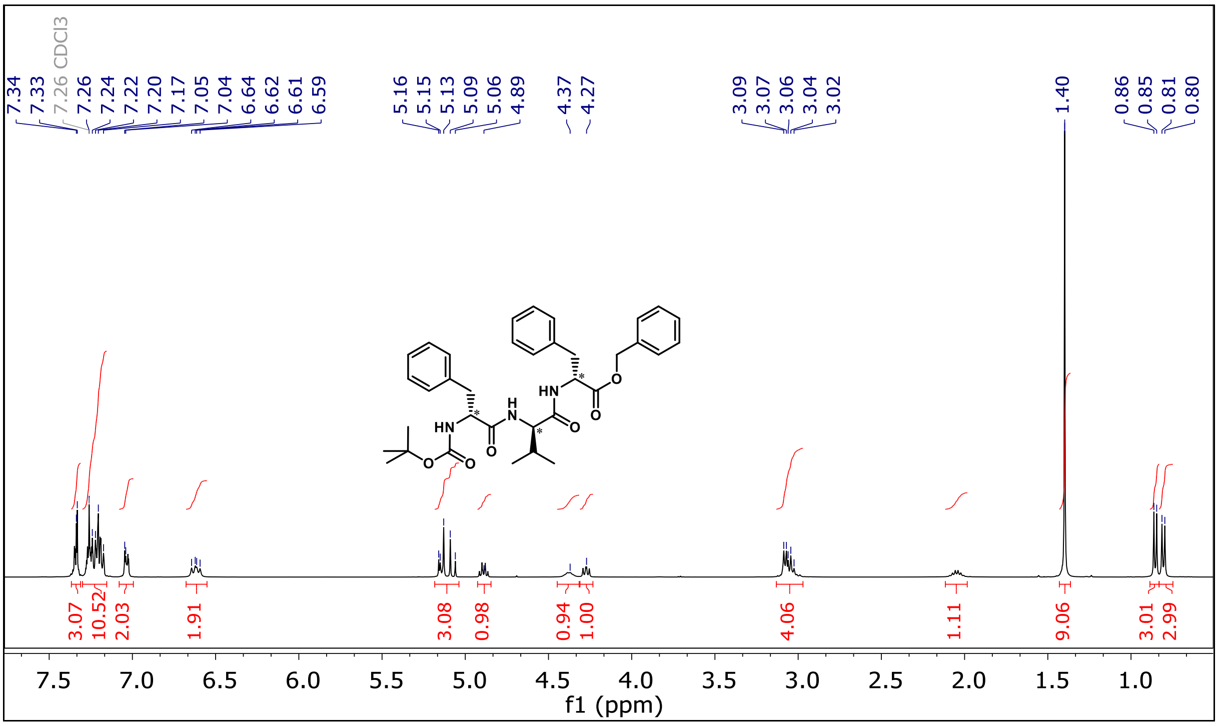

^{13}C NMR (101 MHz, $CDCl_3$)

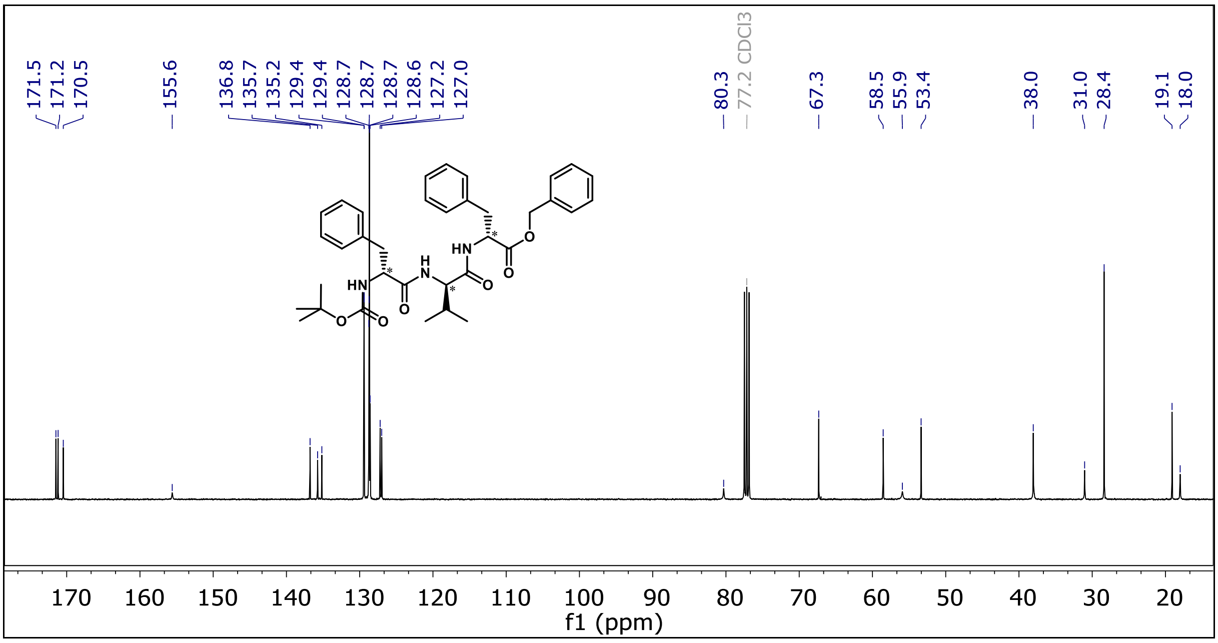

DEPT 135 (101 MHz, $CDCl_3$)

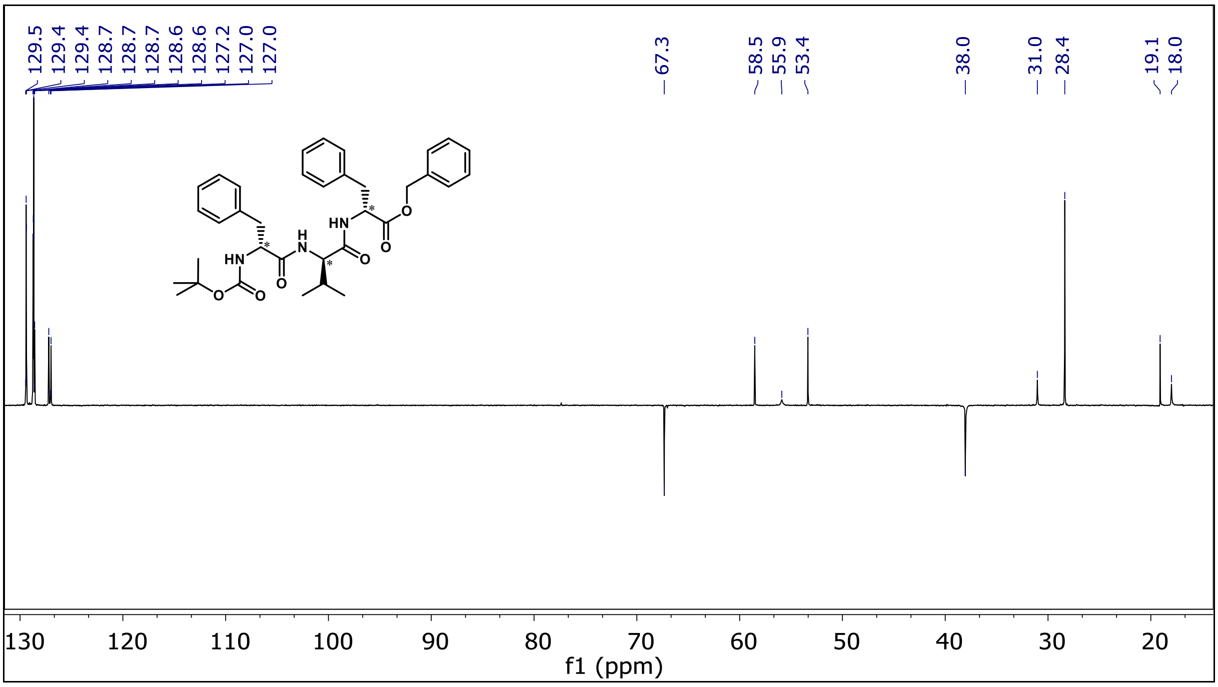
129.5
129.4
129.4
128.7
128.7
128.7
128.6
128.6
127.2
127.0
127.0
67.3
58.5
55.9
53.4
38.0
31.0
28.4
19.1
18.0
HN
H
N
HN
O
130
120
110
100
90
80
70
60
50
40
30
20
f1 (ppm)

HRMS (ESI)

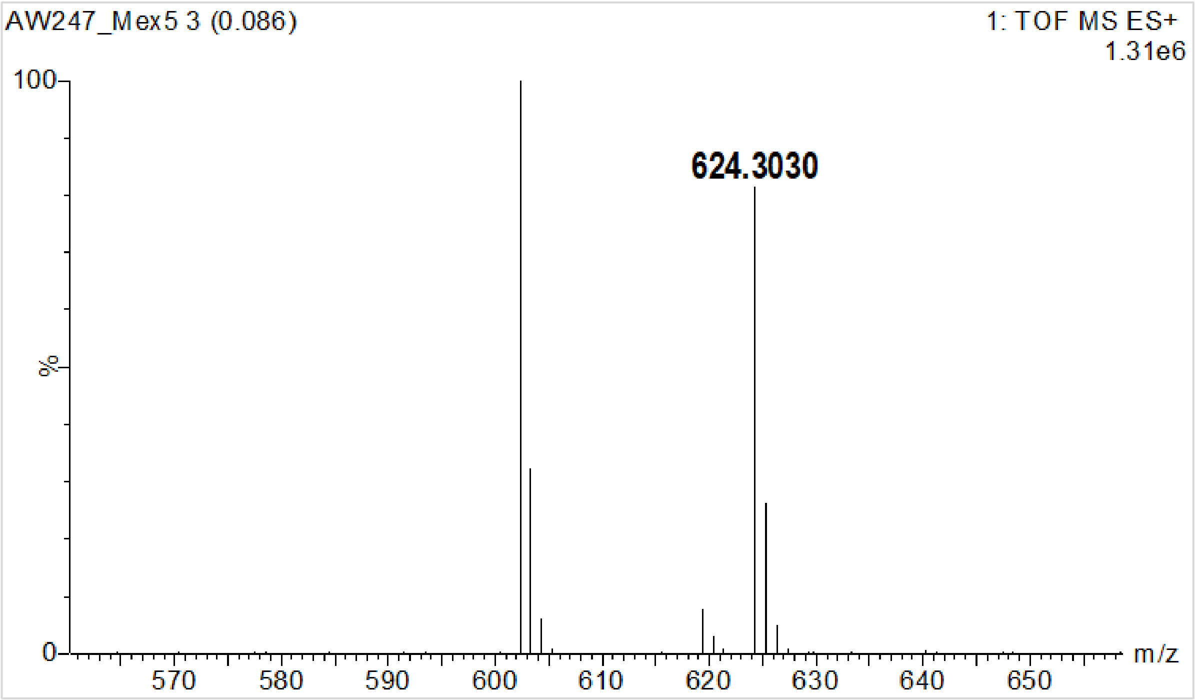
AW247_Mex5 3 (0.086)
1: TOF MS ES+
1.31e6
100
%
0
624.3030
570
580
590
600
610
620
630
640
650
m/z

(tert-butoxycarbonyl)-D-phenylalanyl-D-valyl-D-phenylalanine

(P7D)

^{1}H NMR (400 MHz, $CDCl_3$)

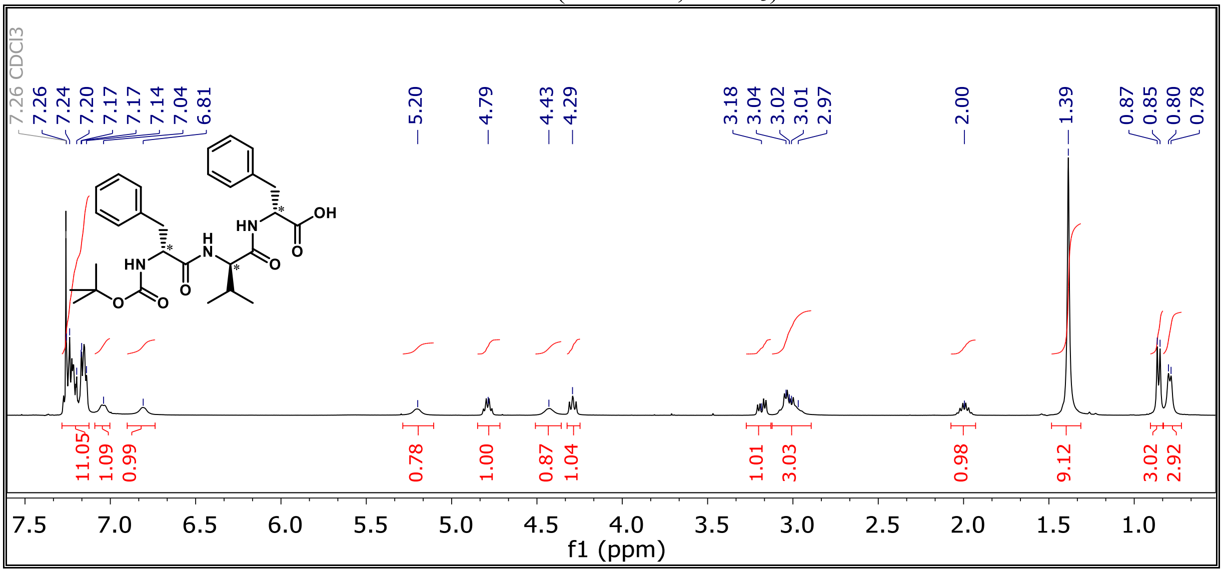

^{13}C NMR (101 MHz, $CDCl_3$)

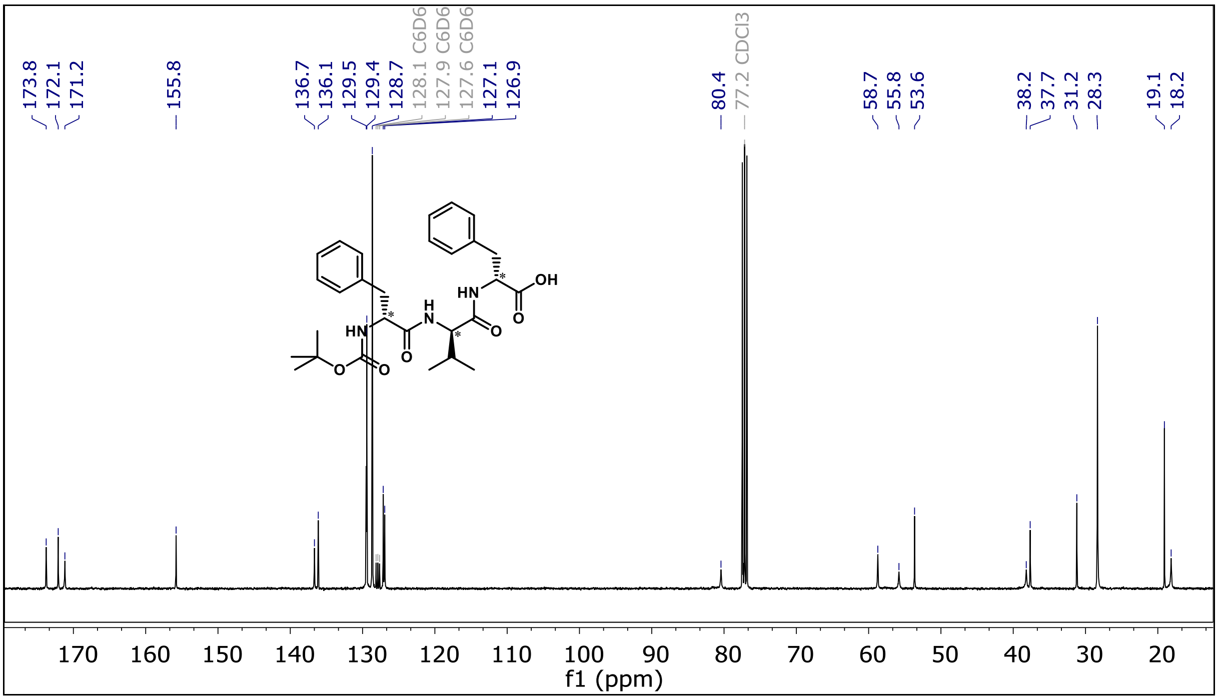

DEPT 135 (101 MHz, $CDCl_3$)

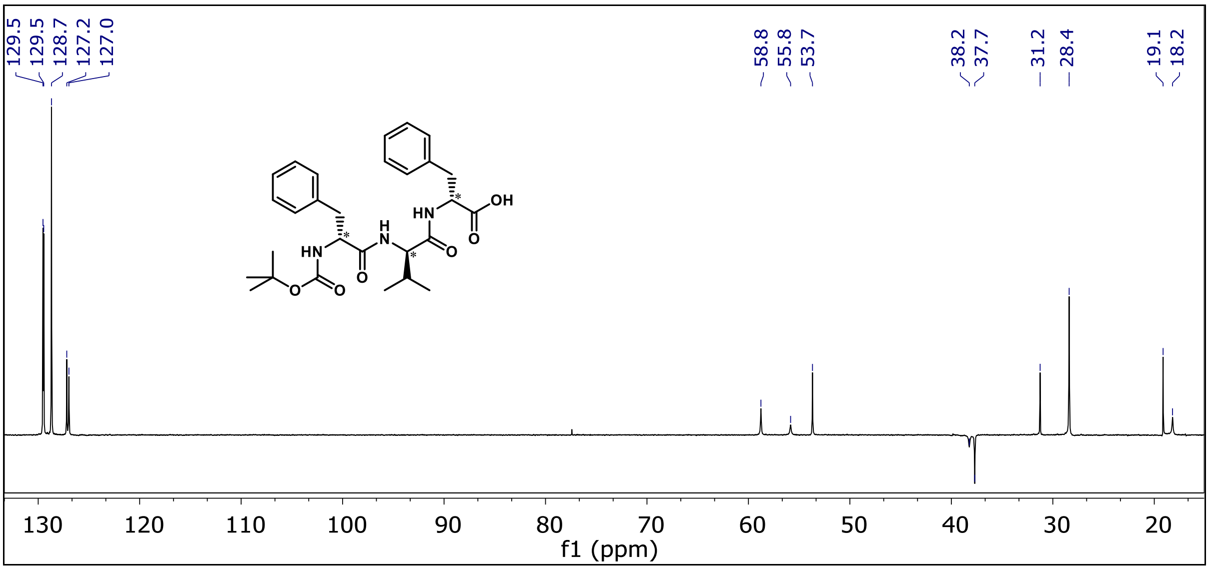

HRMS (ESI)

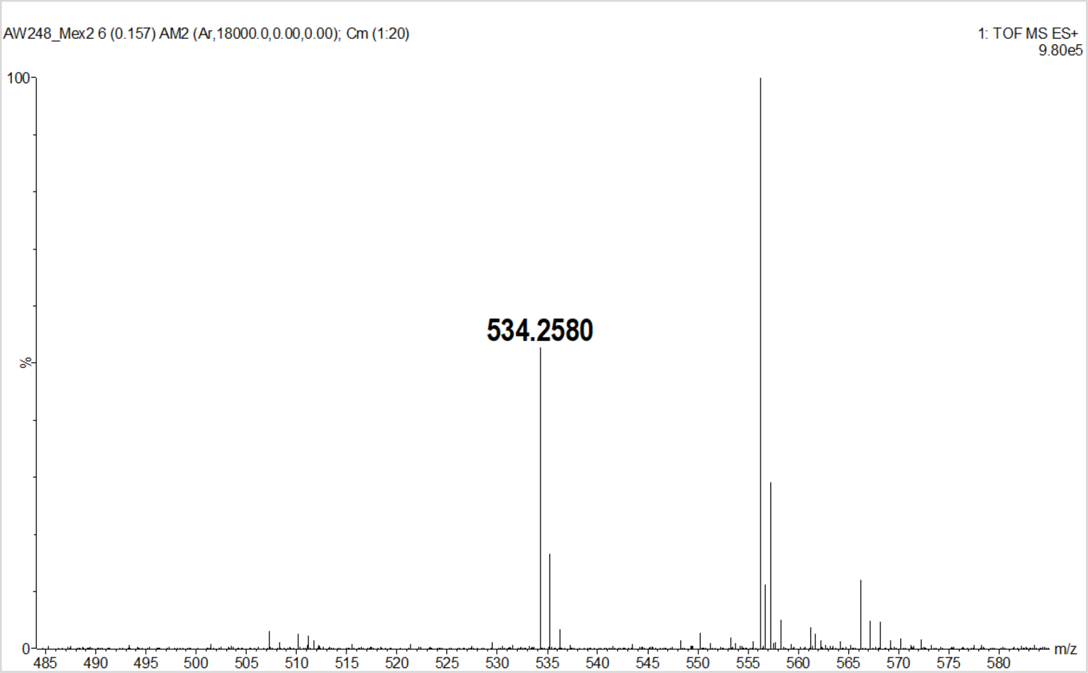

(R)-1-(((R)-1-(((R)-1-(benzyloxy)-1-oxo-3-phenylpropan-2-yl)amino)-3-methyl-1-oxobutan-2-yl)amino)-1-oxo-3-phenylpropan-2-aminium trifluoroacetate

(P8D)

^{1}H NMR (400 MHz, MeOD)

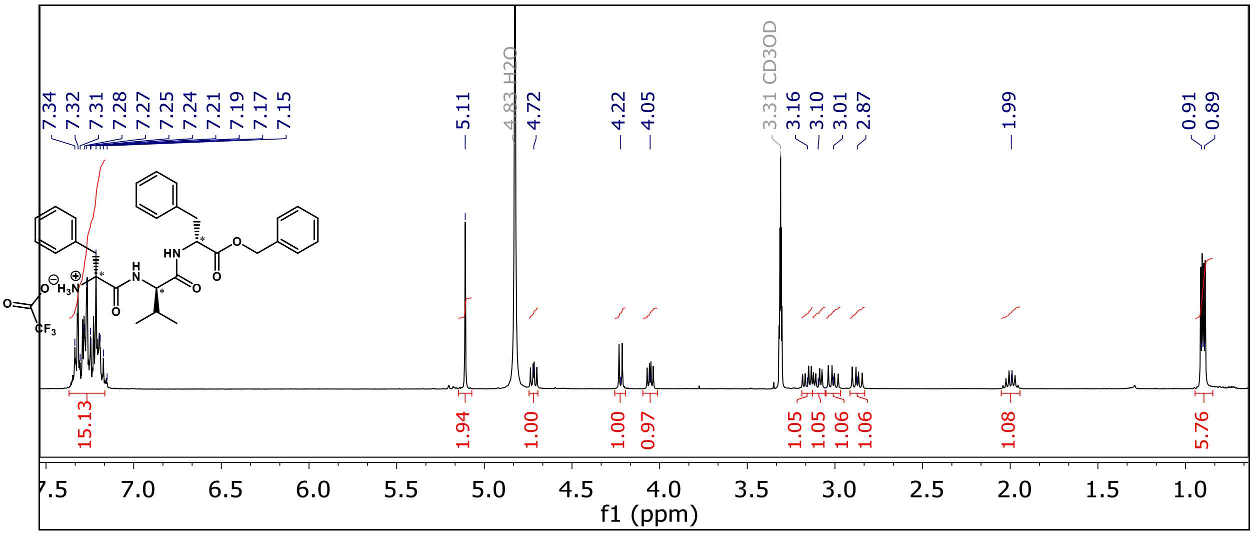

^{19}F NMR (376 MHz, MeOD)

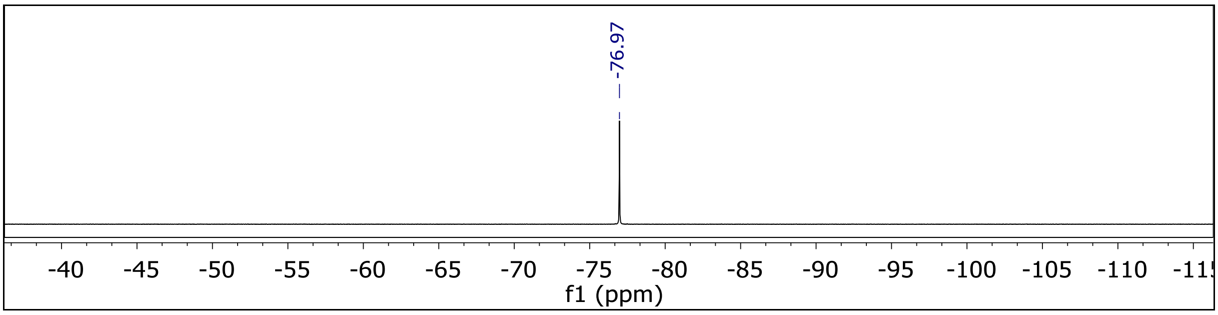

^{13}C NMR (101 MHz, MeOD)

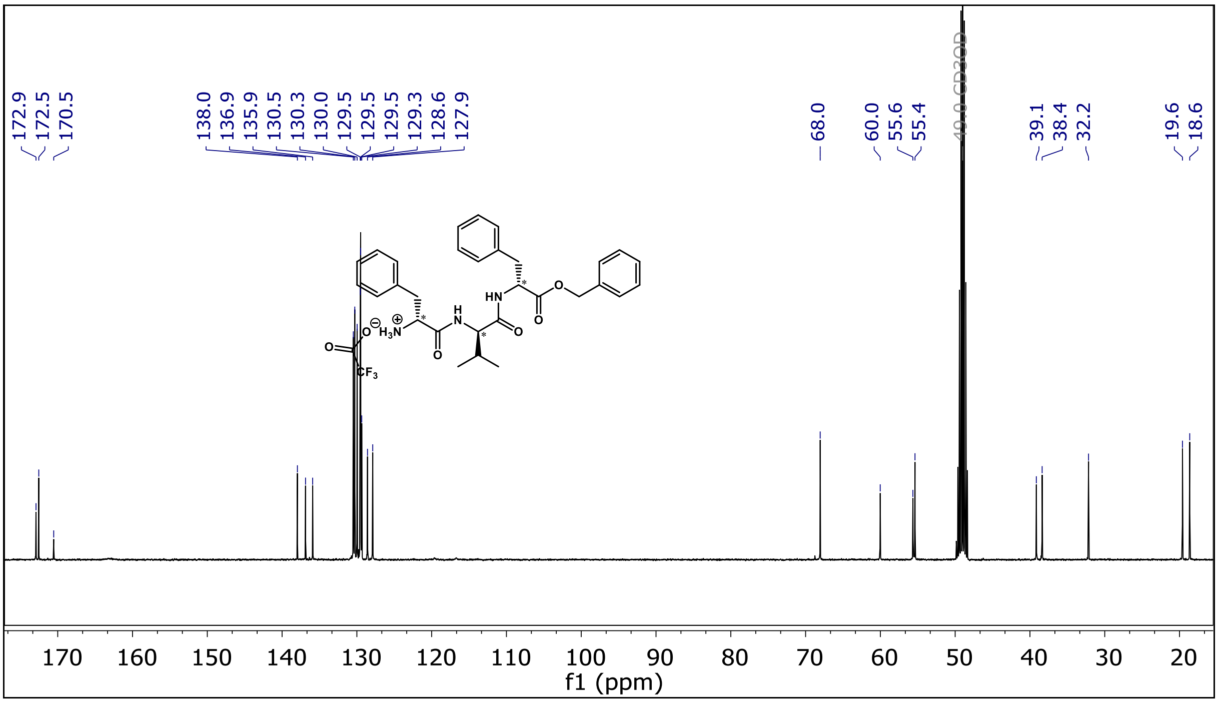

DEPT 135 (101 MHz, MeOD)

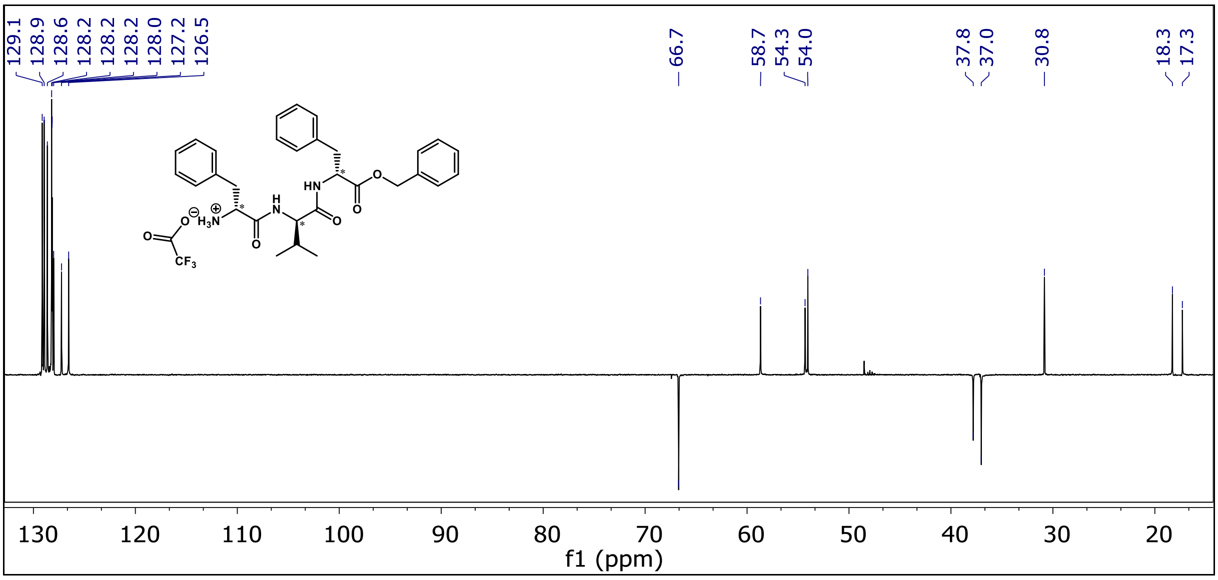

HMRS (ESI)

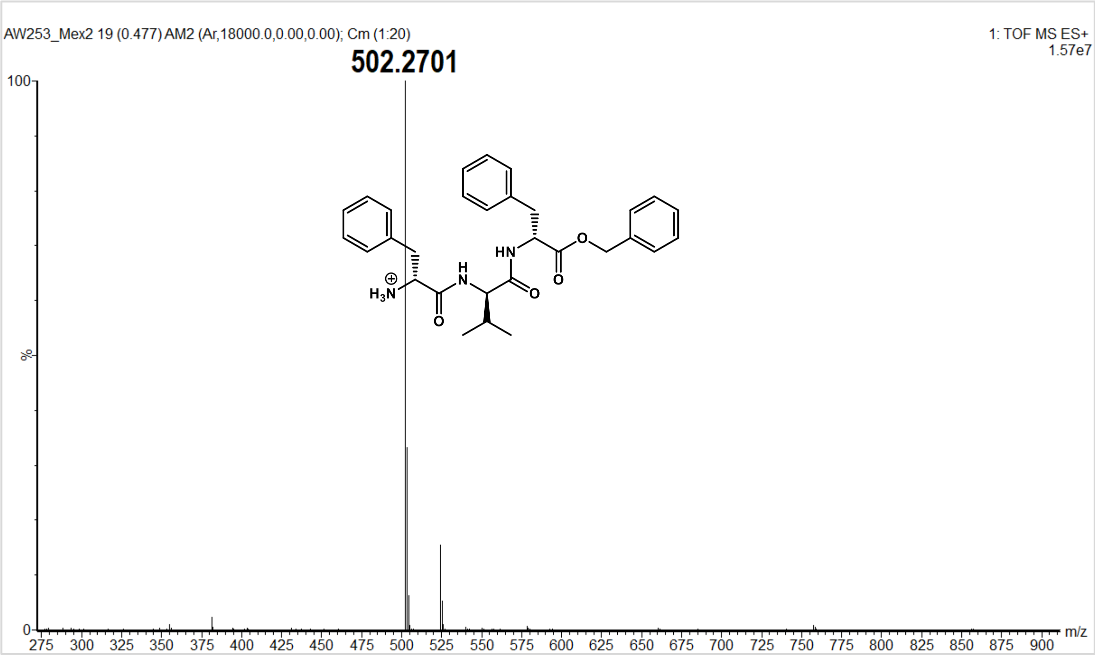

(R)-1-(((R)-1-(((R)-1-carboxy-2-phenylethyl)amino)-3-methyl-1-oxobutan-2-yl)amino)-1-oxo-3-phenylpropan-2-aminium trifluoroacetate

(P11D)

^{1}H NMR (400 MHz, MeOD)

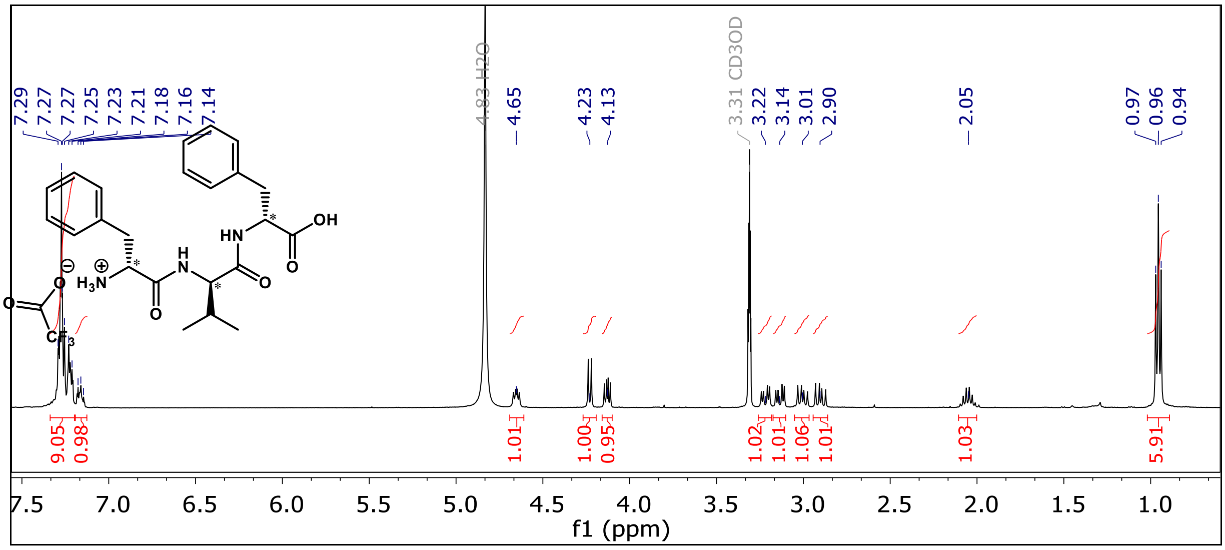

^{19}F NMR (376 MHz, MeOD)

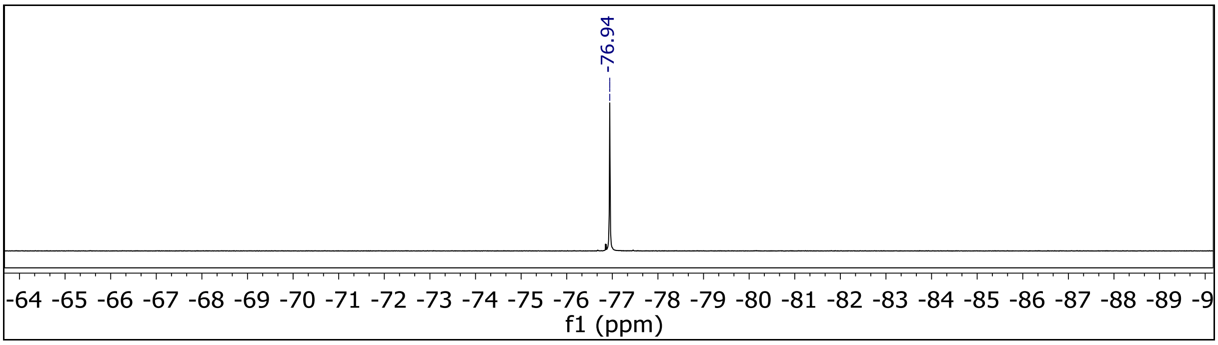

^{13}C NMR (101 MHz, DMSO)

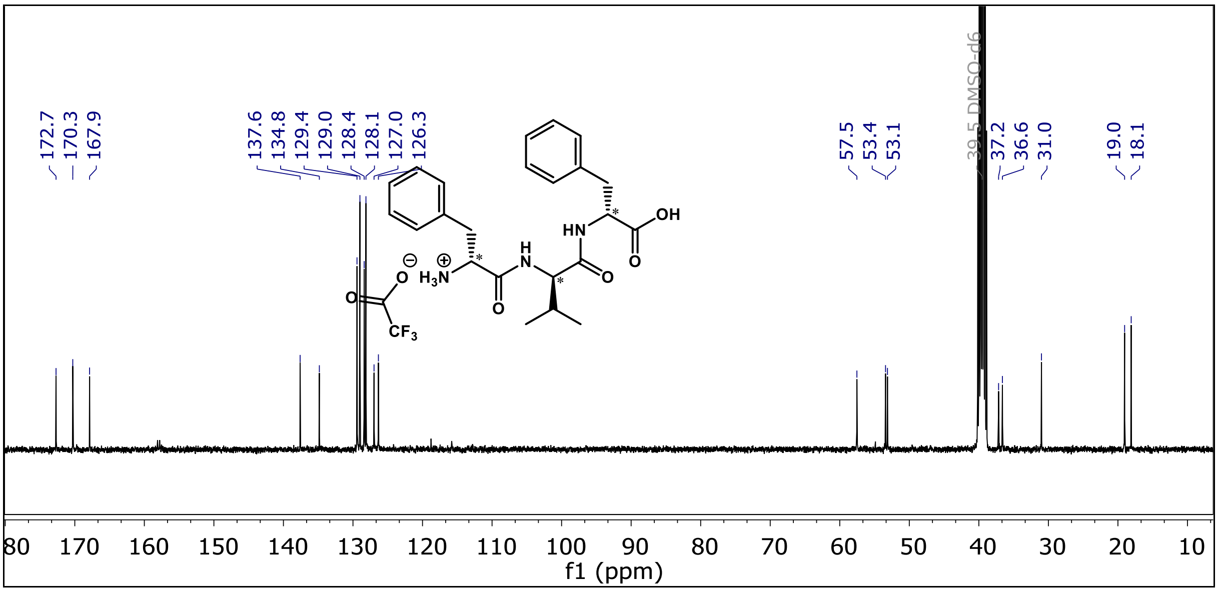

DEPT 135 (101 MHz, DMSO)

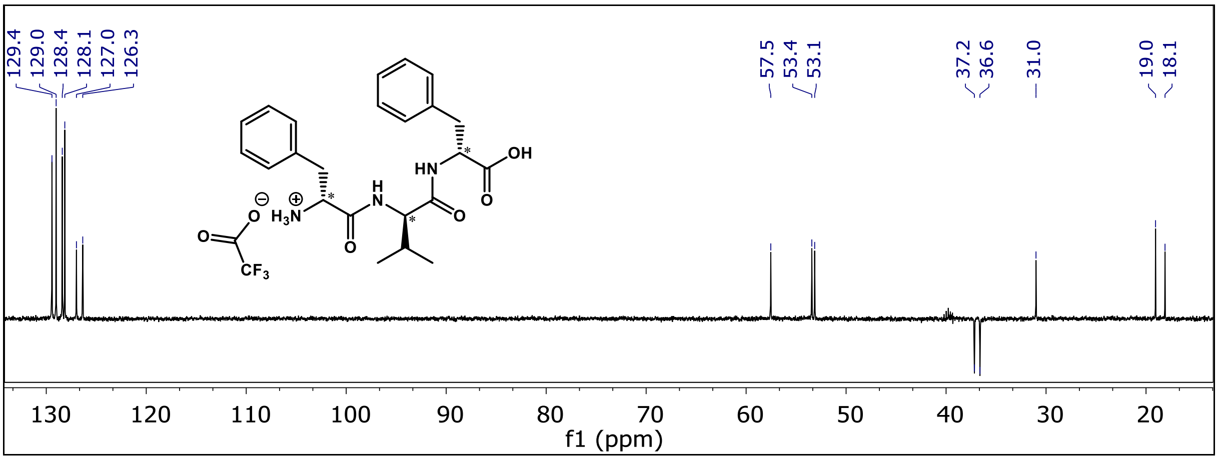

HRMS (ESI)

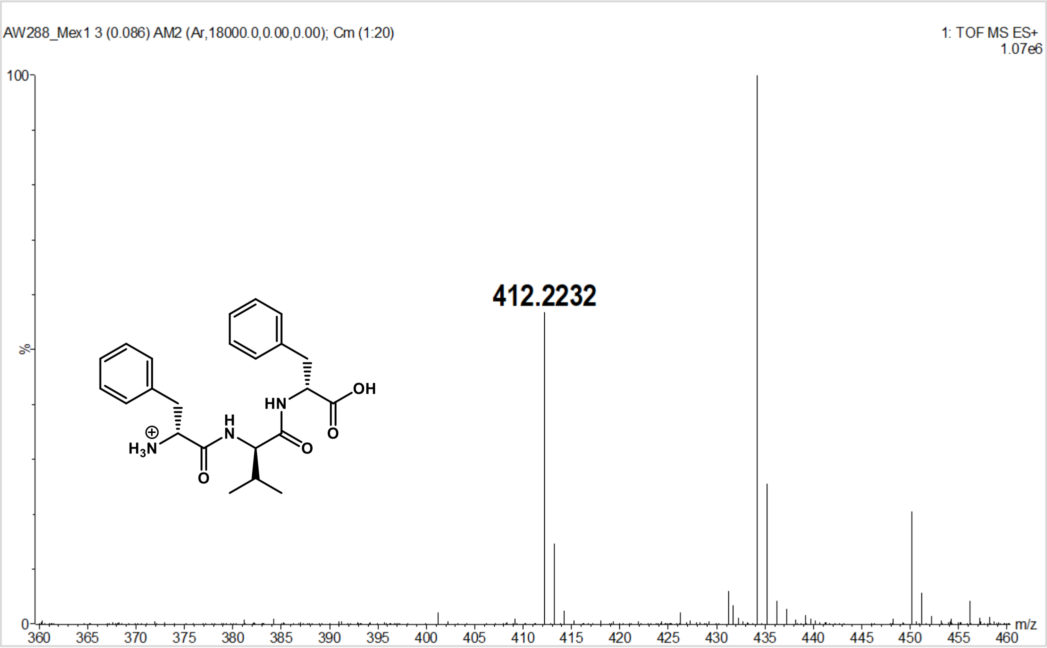

tert-butyl ((R)-1-(((R)-1-(((R)-1-((4-ethylphenyl)amino)-1-oxo-3-phenylpropan-2-yl)amino)-3-methyl-1-oxobutan-2-yl)amino)-1-oxo-3-phenylpropan-2-yl)carbamate

(P21D)

^{1}H NMR (400 MHz, $CDCl_3$)

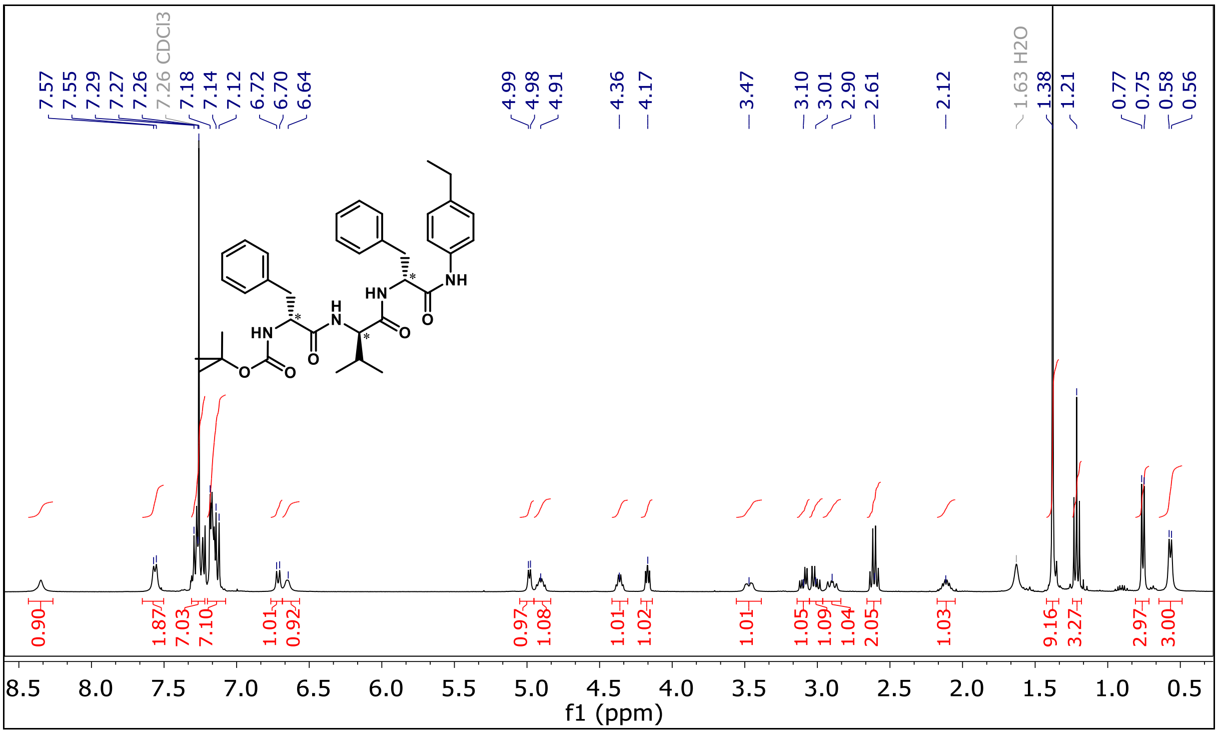

^{13}C NMR (101 MHz, $CDCl_3$)

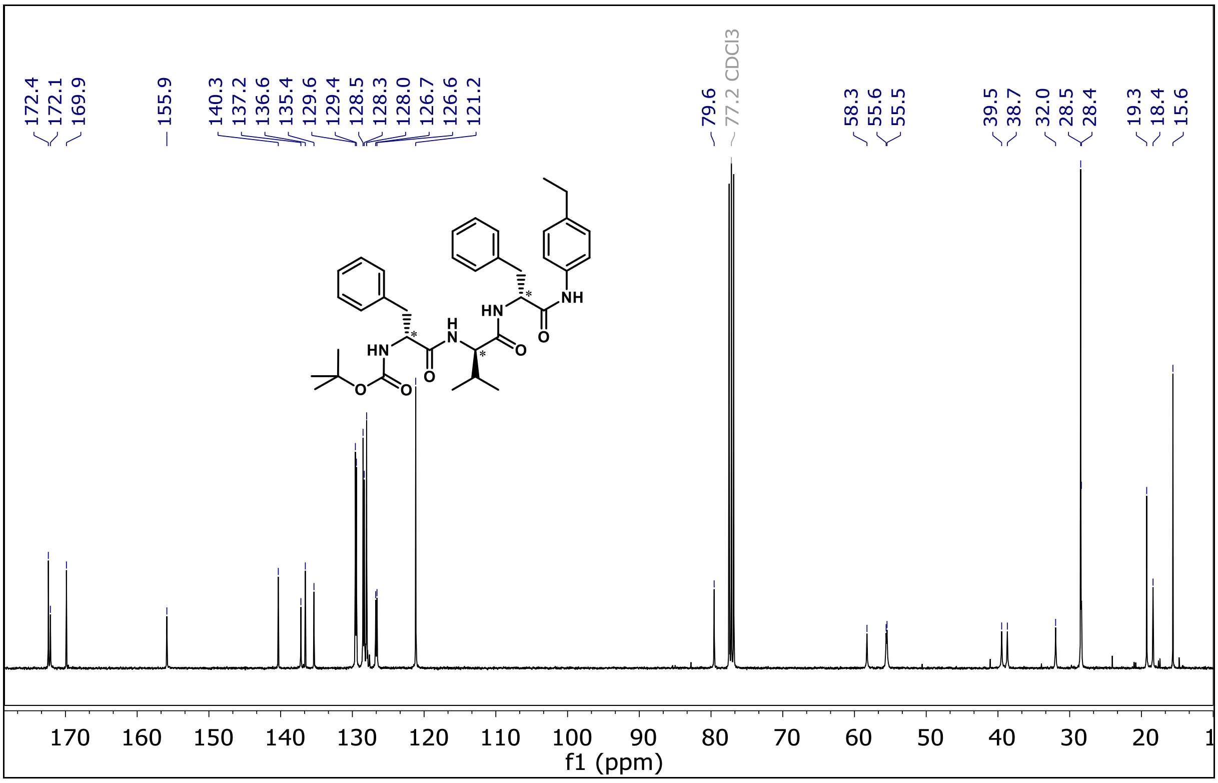

DEPT 135 (101 MHz, $CDCl_3$)

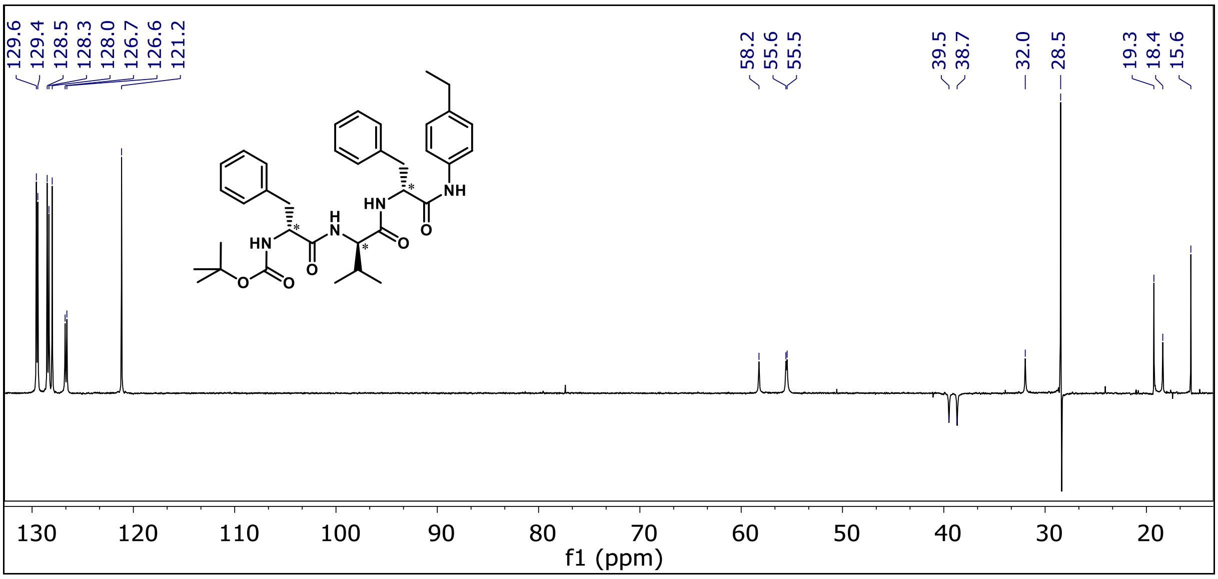

HRMS (ESI)

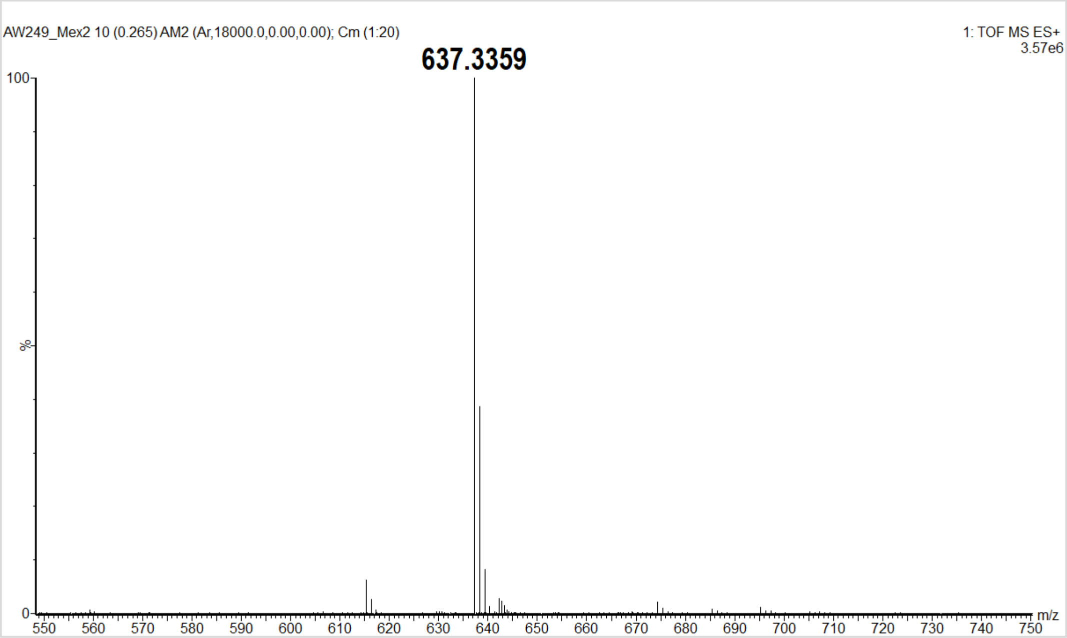

(R)-1-(((R)-1-(((R)-1-((4-ethylphenyl)amino)-1-oxo-3-phenylpropan-2-yl)amino)-3-methyl-1-oxobutan-2-yl)amino)-1-oxo-3-phenylpropan-2-aminium trifluoroacetate

(P22D)

^{1}H NMR (400 MHz, MeOD)

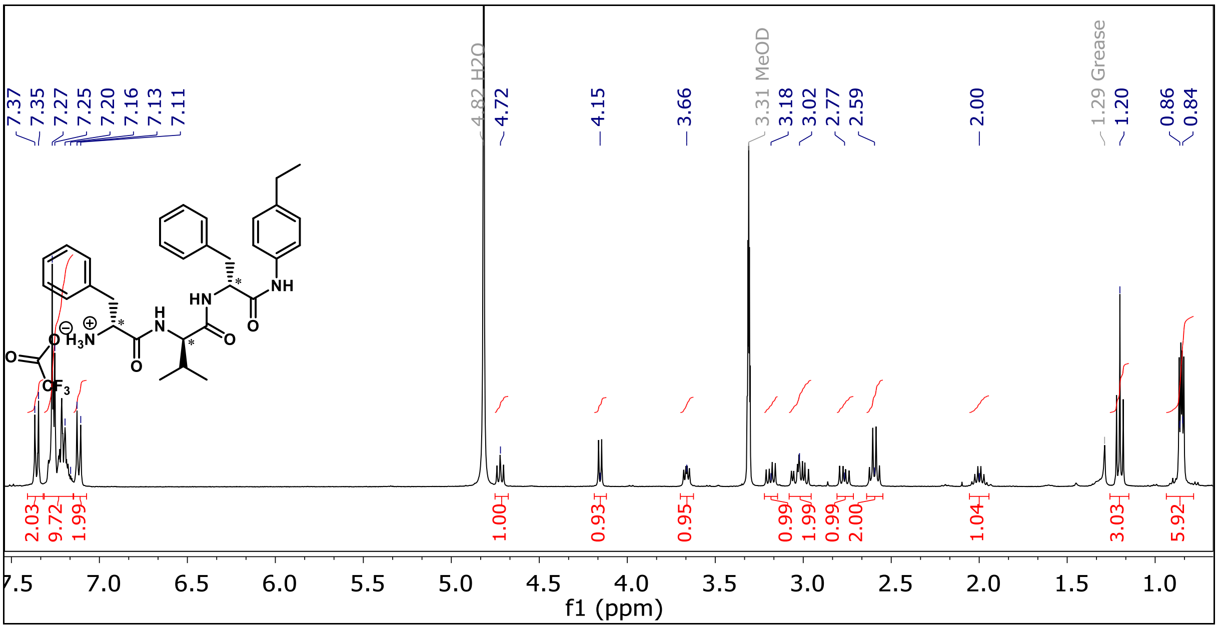

^{19}F NMR (376 MHz, MeOD)

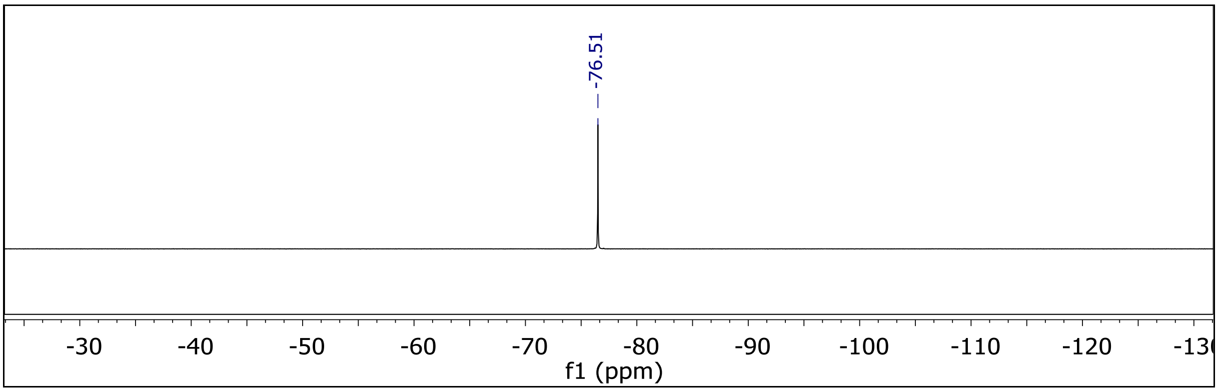

^{13}C NMR (101 MHz, MeOD-$CDCl_3$ (1 : 1 ; v : v))

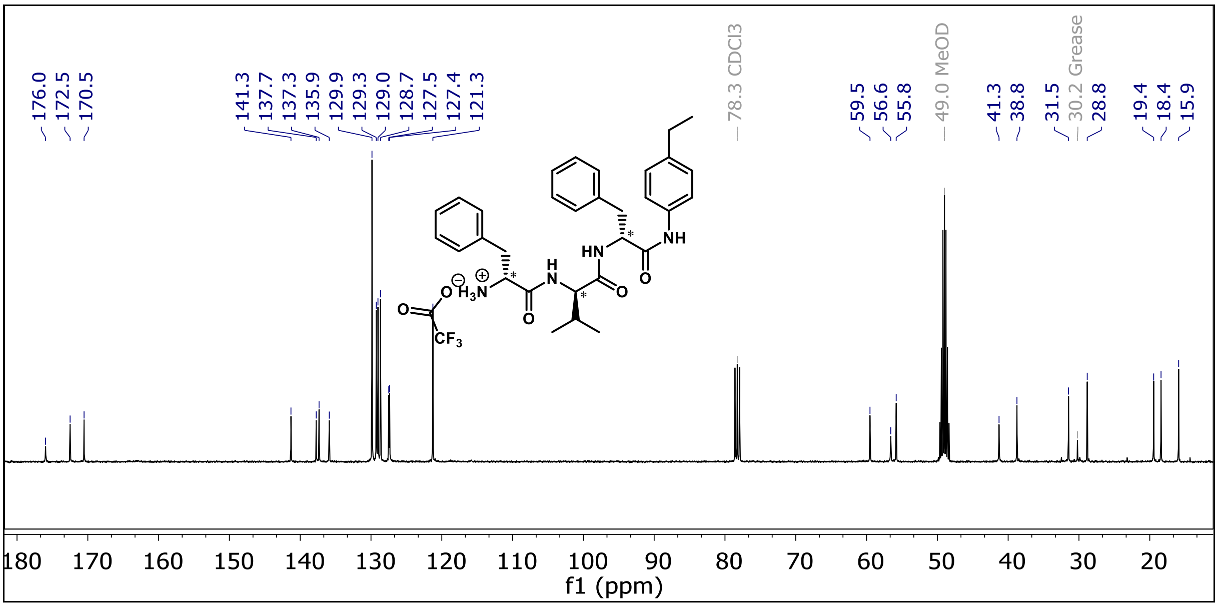

DEPT 135 (101 MHz, MeOD-$CDCl_3$ (1 : 1 ; v : v))

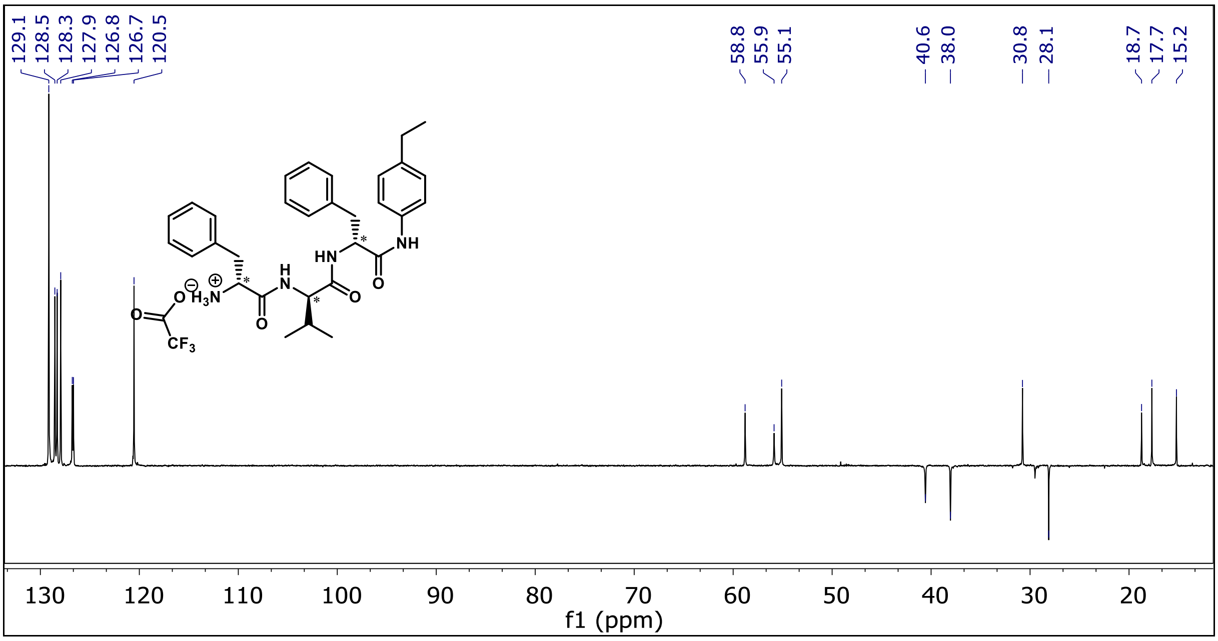

HRMS (ESI)

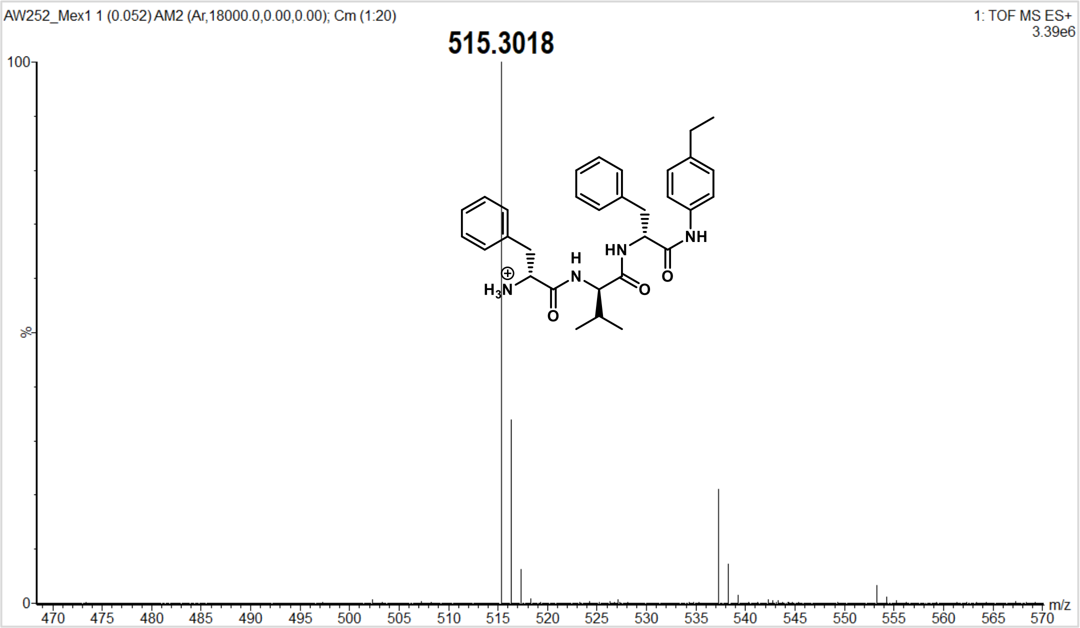

tert-butyl ((2S)-1-oxo-3-phenyl-1-((4-(1-((2,2,6,6-tetramethylpiperidin-1-yl)oxy)ethyl)phenyl)amino)propan-2-yl)carbamate

(A1L)

^{1}H NMR (400 MHz, $CDCl_3$)

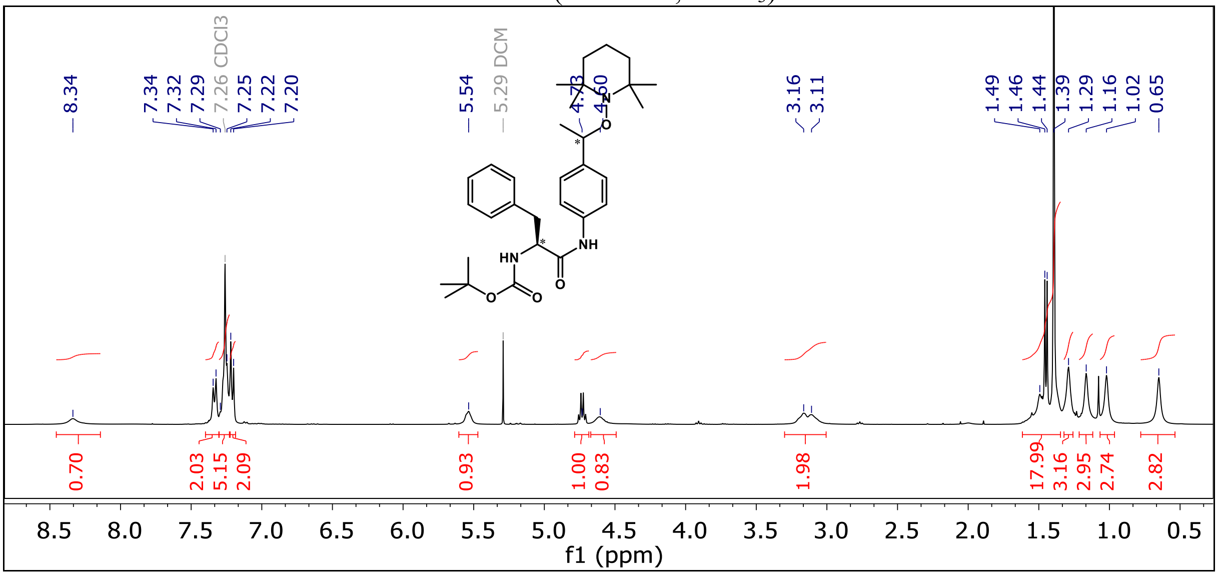

^{13}C NMR (101 MHz, CDCl$_3$)

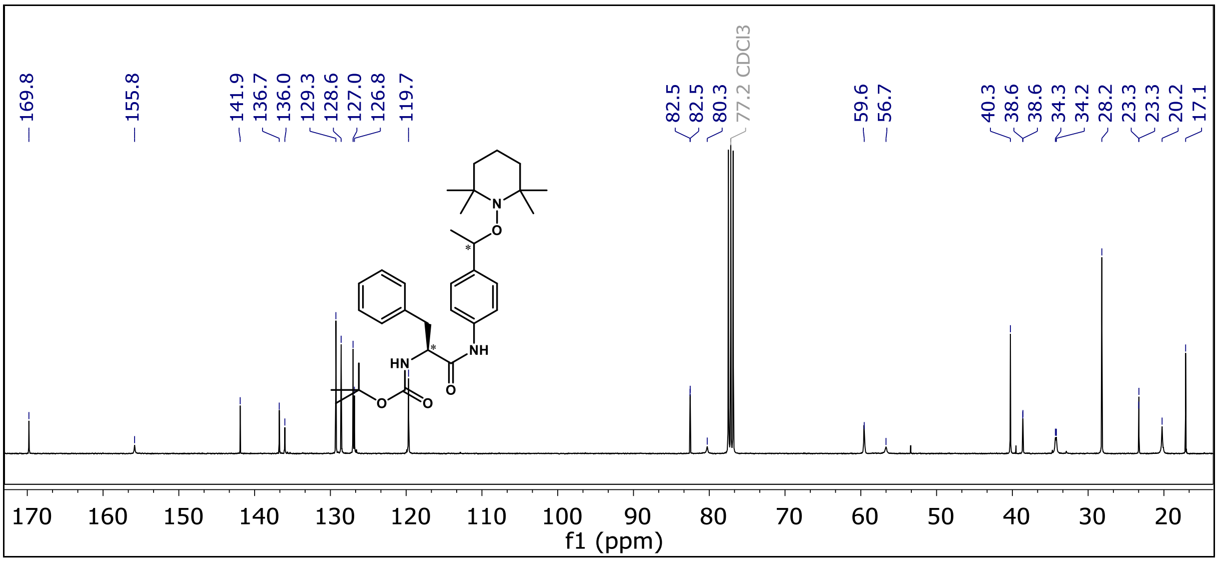

DEPT 135 (101 MHz, CDCl$_3$)

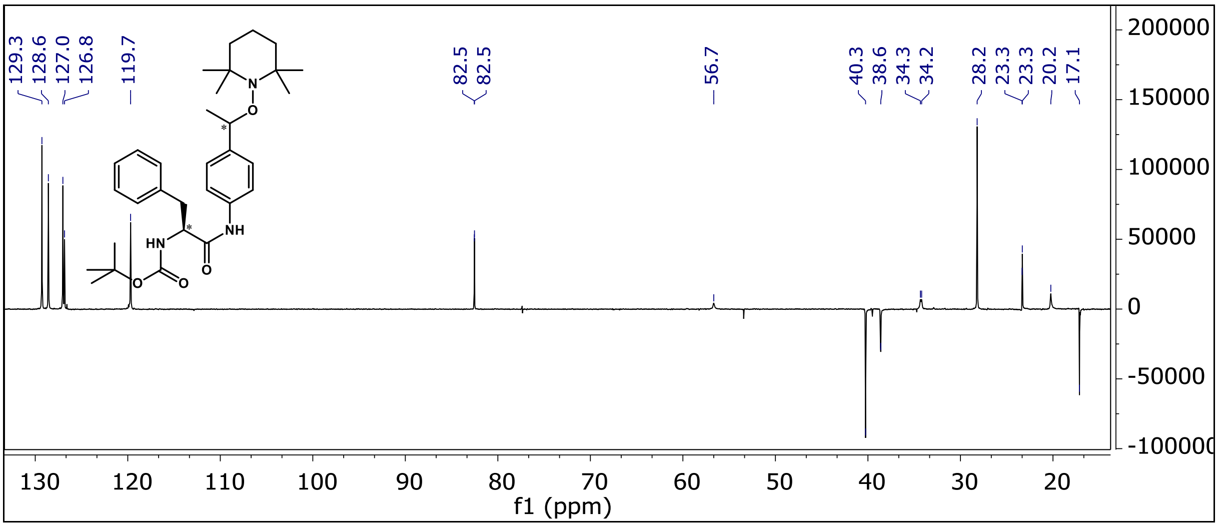

HRMS (ESI)

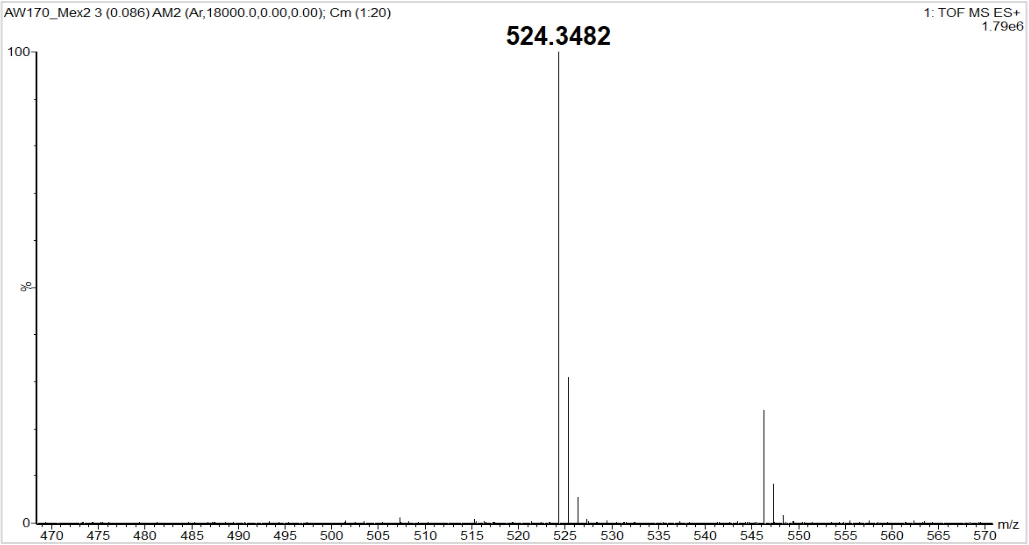

tert-butyl ((2S)-1-(((2S)-3-methyl-1-oxo-1-(((2S)-1-oxo-3-phenyl-1-((4-(1-((2,2,6,6-tetramethylpiperidin-1-yl)oxy)ethyl)phenyl)amino)propan-2-yl)amino)butan-2-yl)amino)-1-oxo-3-phenylpropan-2-yl)carbamate

(A3L)

^{1}H NMR (300 MHz, MeOD)

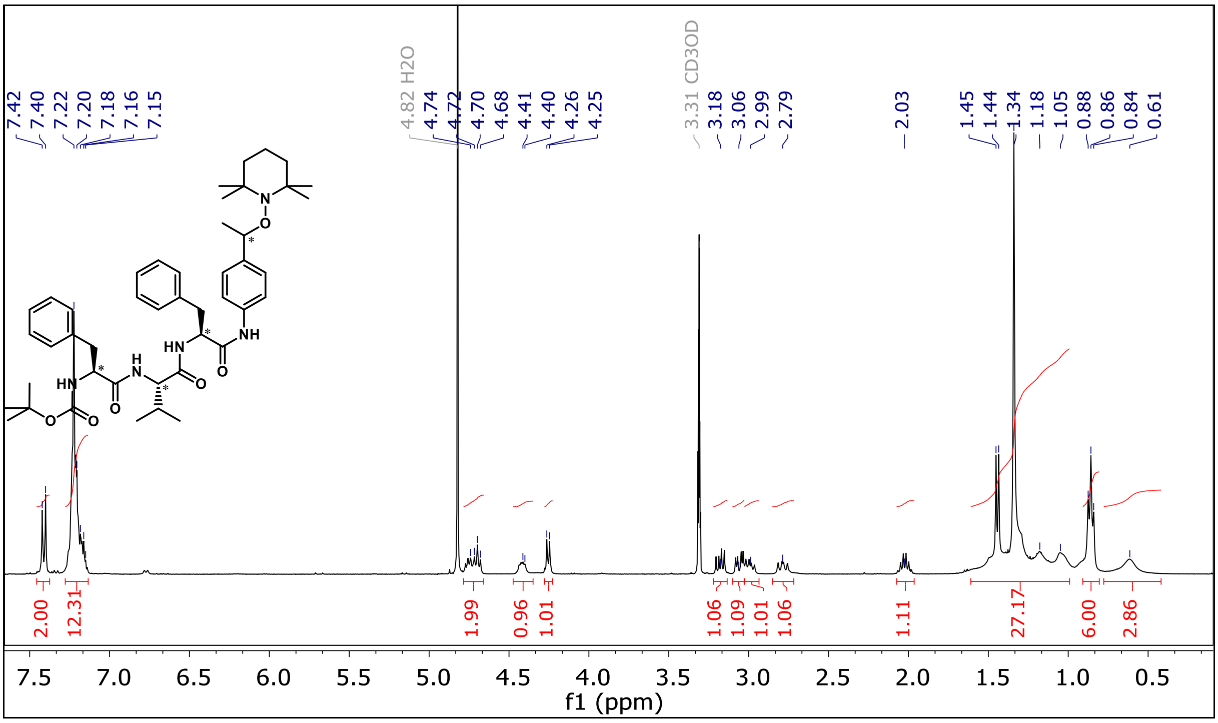

^{13}C NMR (101 MHz, $CDCl_3$)

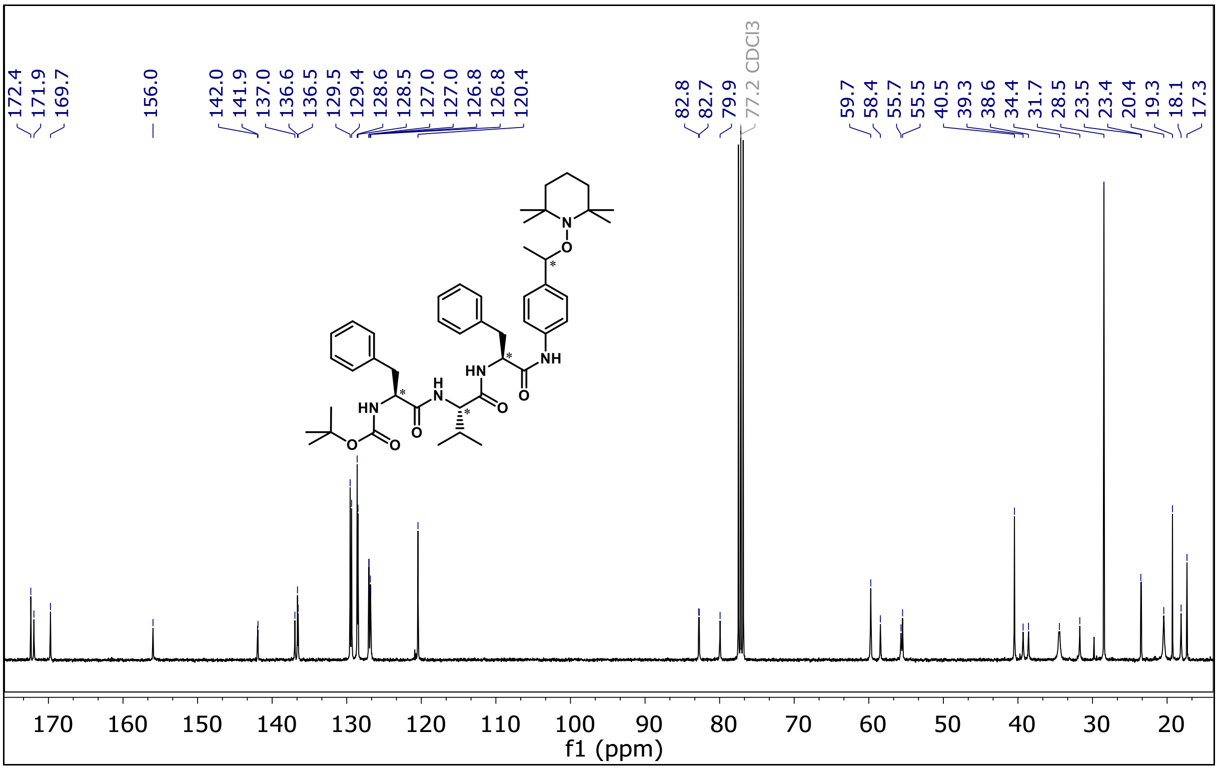

DEPT 135 (101 MHz, $CDCl_3$)

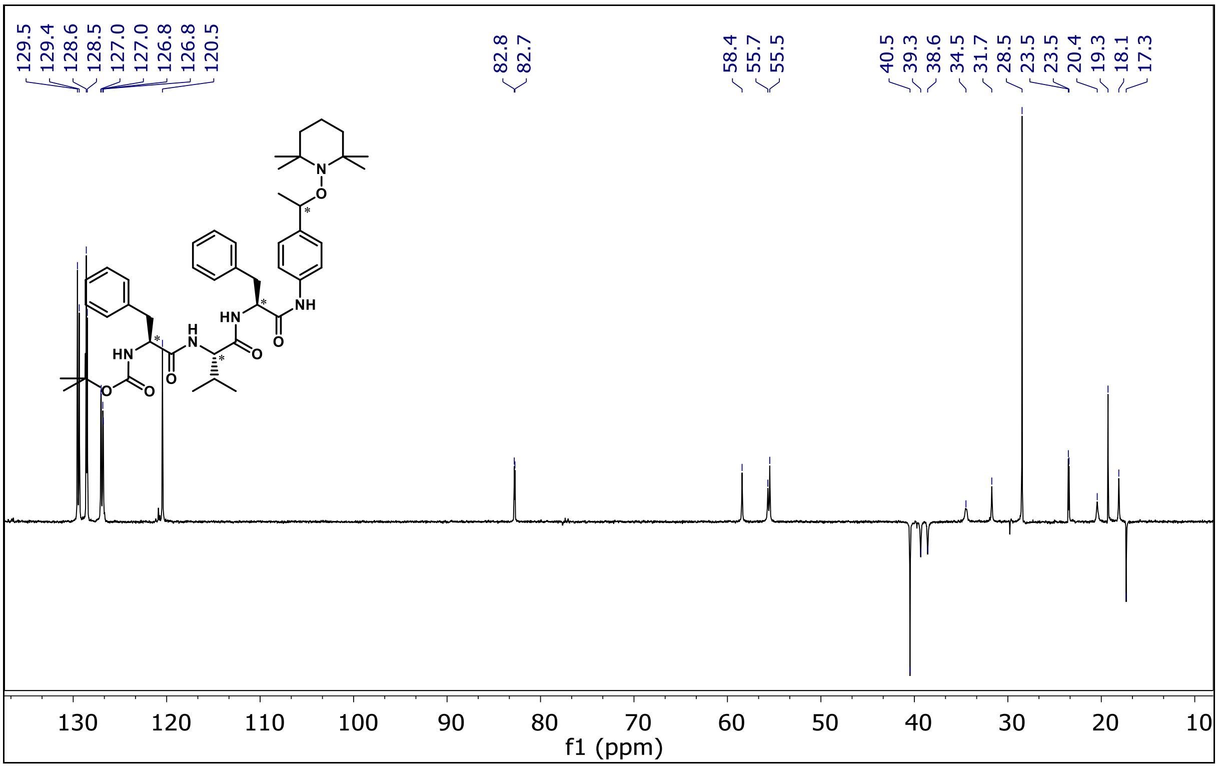
129.5
129.4
128.6
128.5
127.0
127.0
126.8
126.8
120.5
82.8
82.7
58.4
55.7
55.5
40.5
39.3
38.6
34.5
31.7
28.5
23.5
23.5
20.4
19.3
18.1
17.3
130
120
110
100
90
80
70
60
50
40
30
20
10
f1 (ppm)

HRMS (ESI)

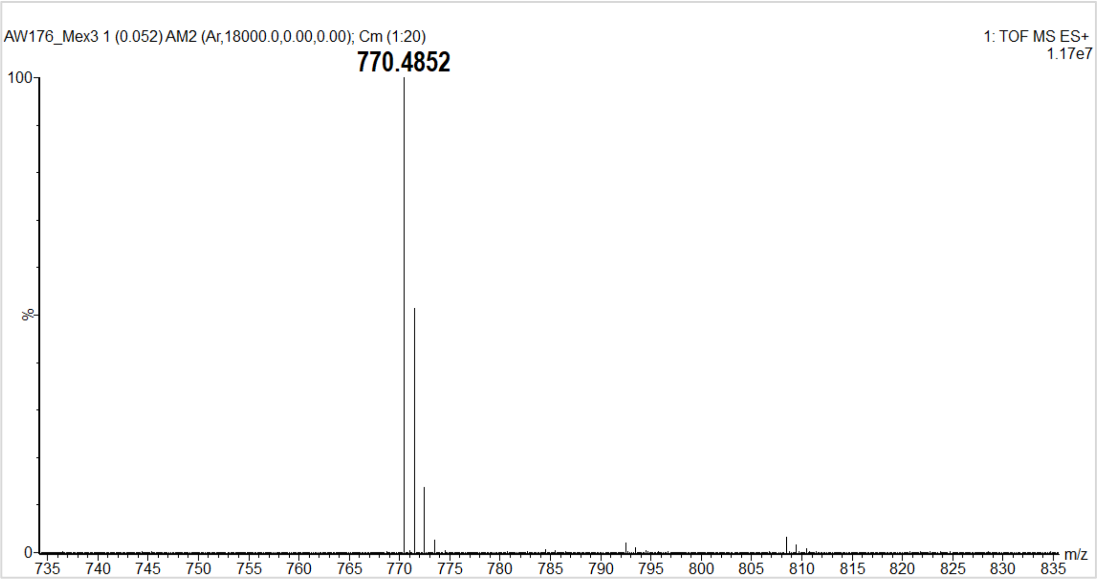
AW176_Mex3 1 (0.052) AM2 (Ar,18000.0,0.00,0.00); Cm (1:20)
1: TOF MS ES+
1.17e7
770.4852
100
%
0
m/z

(2S)-1-(((2S)-3-methyl-1-oxo-1-(((2S)-1-oxo-3-phenyl-1-((4-(1-((2,2,6,6-tetramethylpiperidin-1-yl)oxy)ethyl)phenyl)amino)propan-2-yl)amino)butan-2-yl)amino)-1-oxo-3-phenylpropan-2-aminium trifluoroacetate

(A4L)

^{1}H NMR (300 MHz, MeOD)

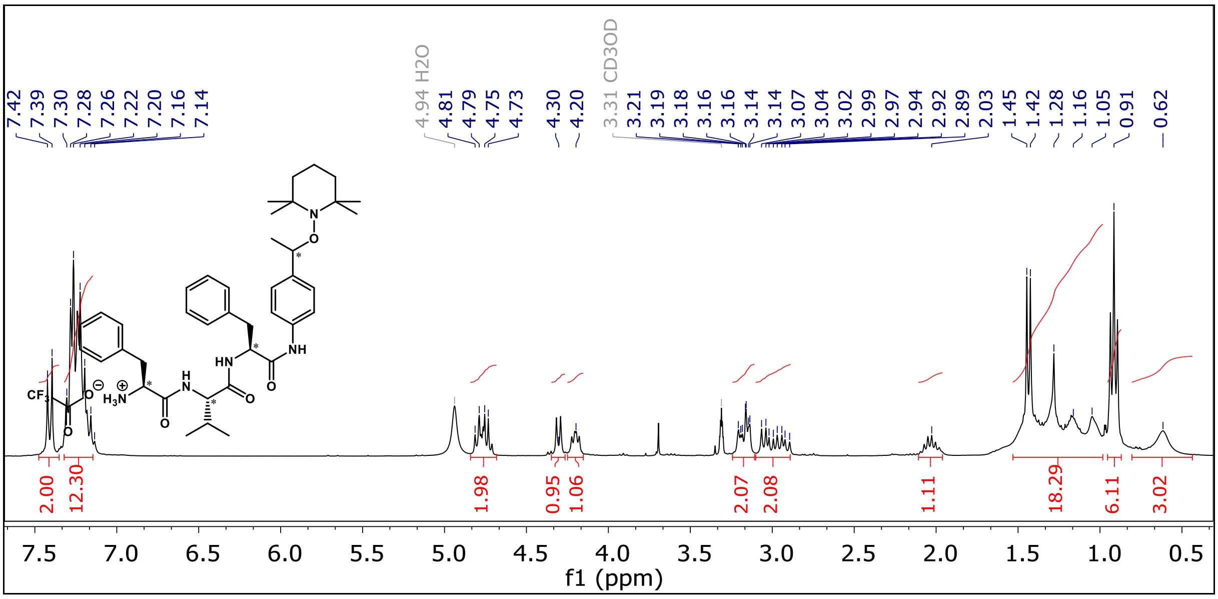

^{13}C NMR (75 MHz, MeOD)

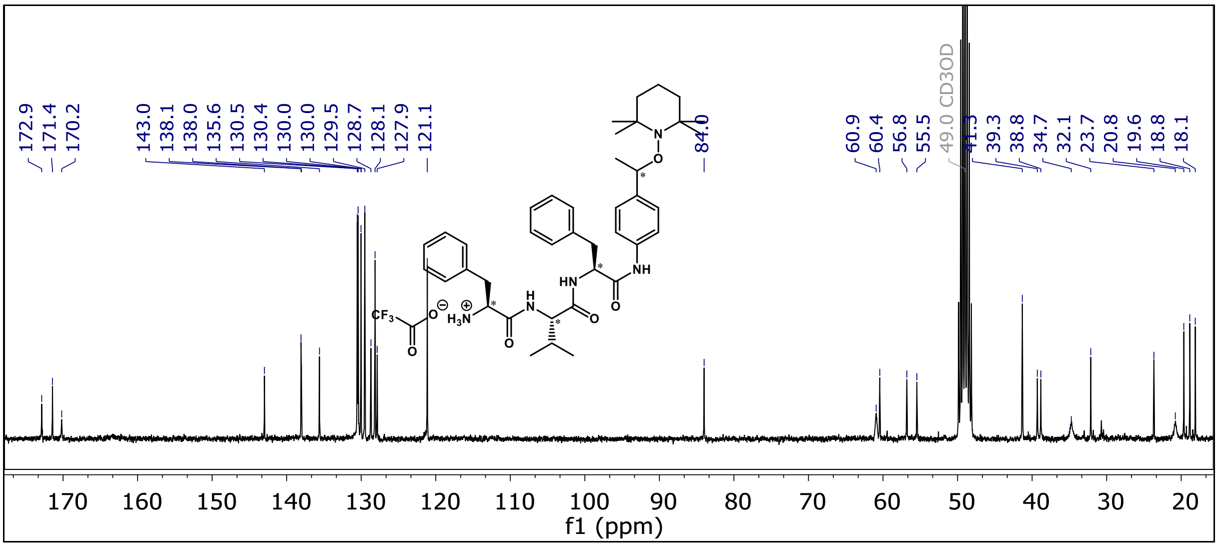

^{19}F NMR (376 MHz, MeOD)

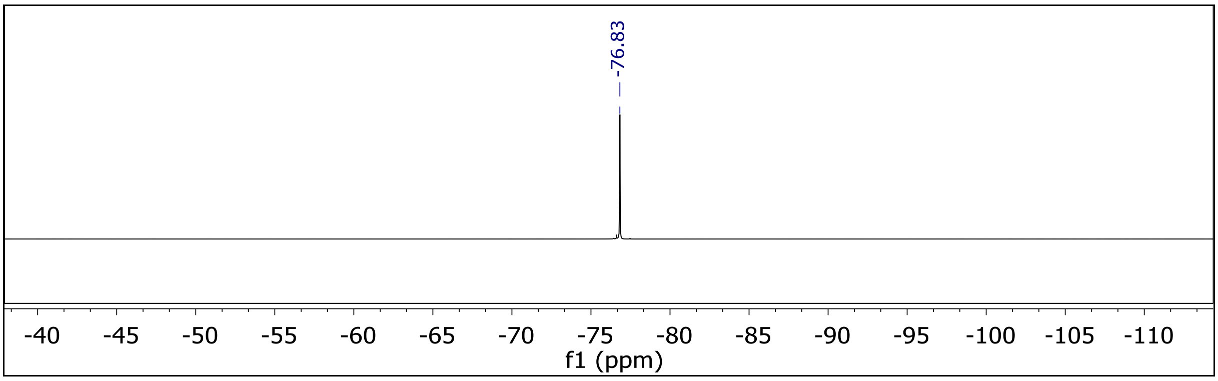

DEPT 135 (75 MHz, MeOD)

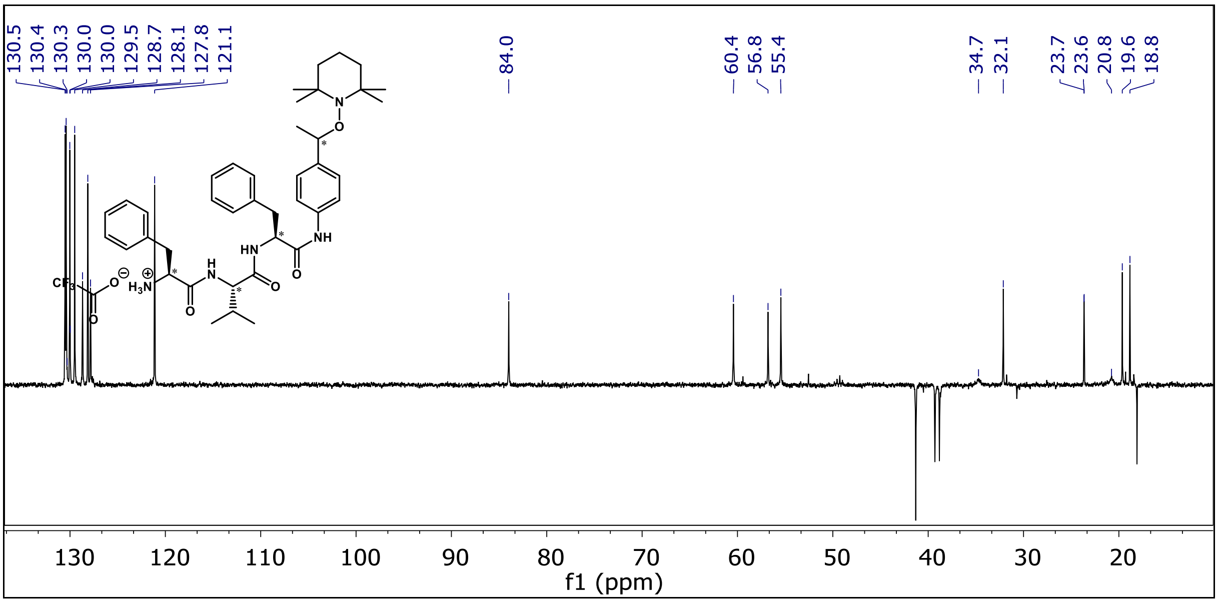

HRMS (ESI)

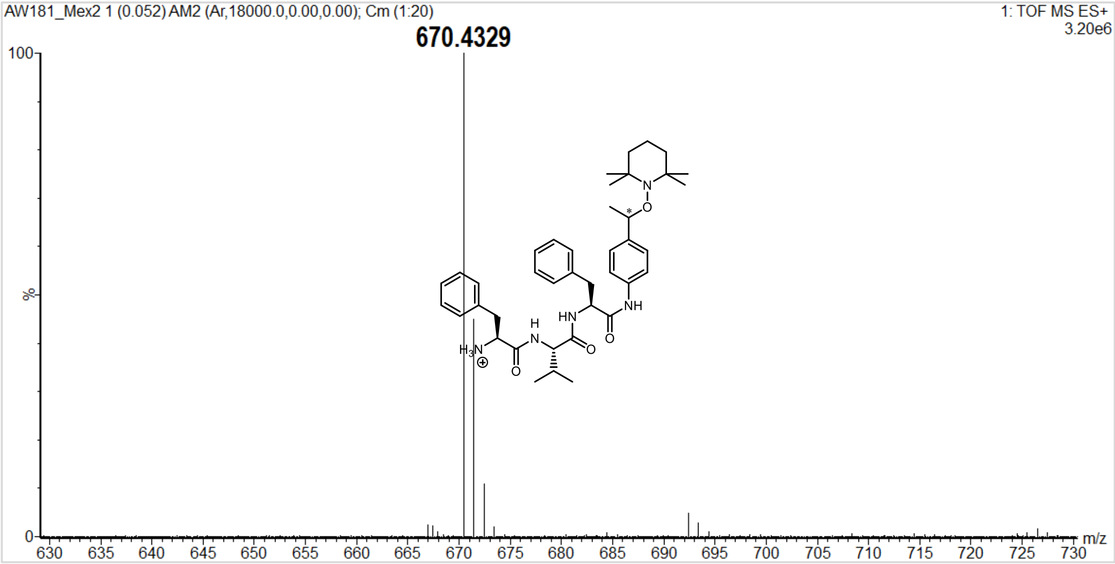

(2S)-2-((S)-2-amino-3-phenylpropanamido)-3-methyl-N-((2S)-1-oxo-3-phenyl-1-((4-(1-((2,2,6,6-tetramethylpiperidin-1-yl)oxy)ethyl)phenyl)amino)propan-2-yl)butanamide

(A5L)

^{1}H NMR (400 MHz, MeOD)

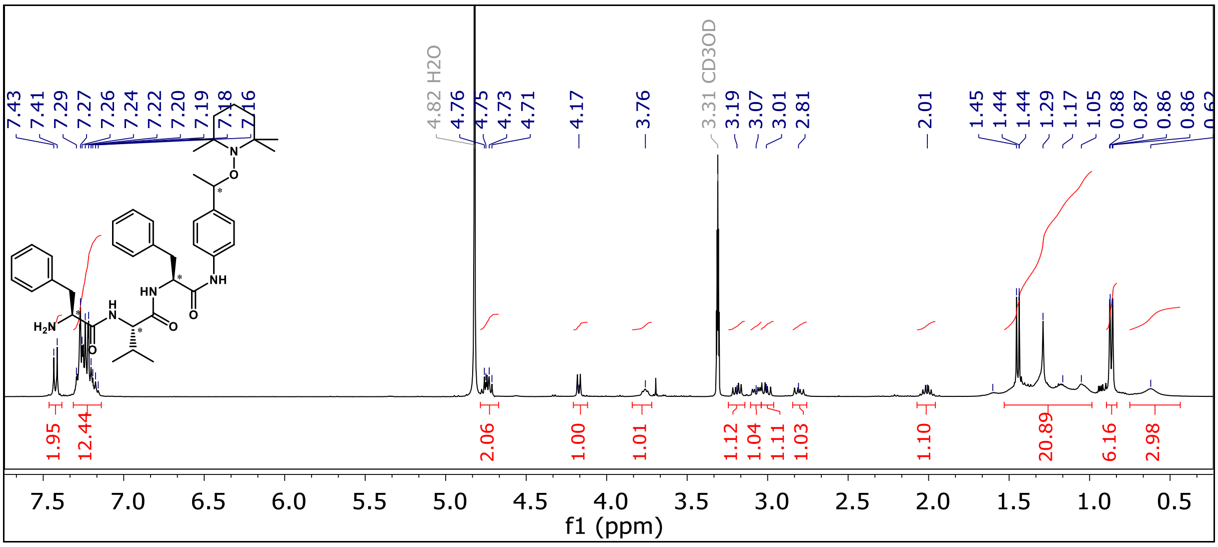

^{13}C NMR (101 MHz, MeOD-$CDCl_3$ (1 : 1 ; v : v))

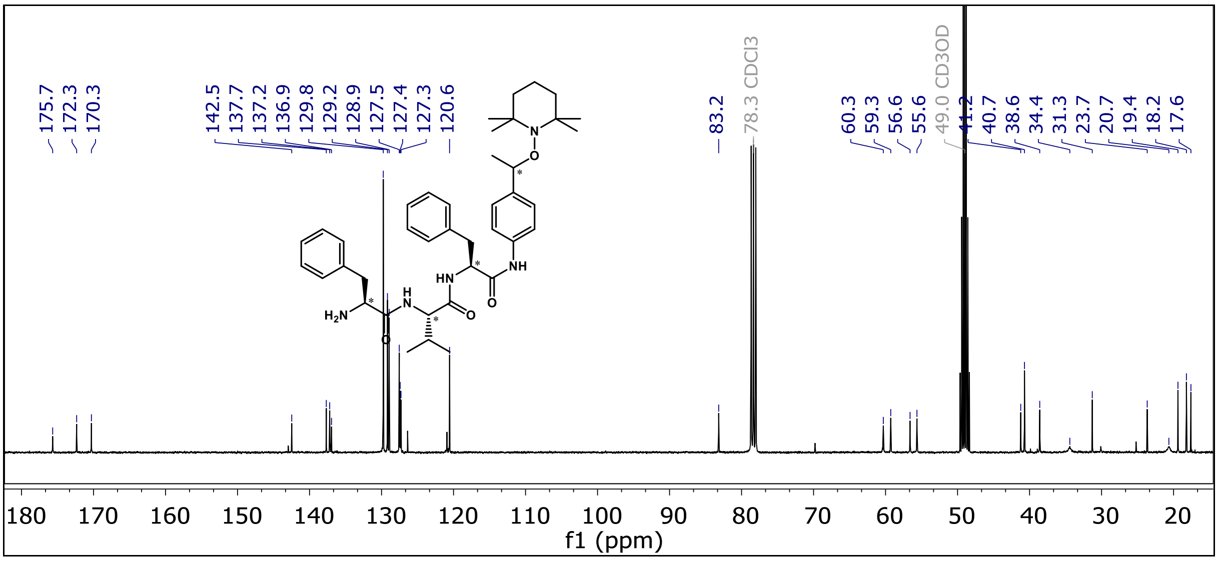

DEPT 135 (101 MHz, MeOD-$CDCl_3$ (1 : 1 ; v : v))

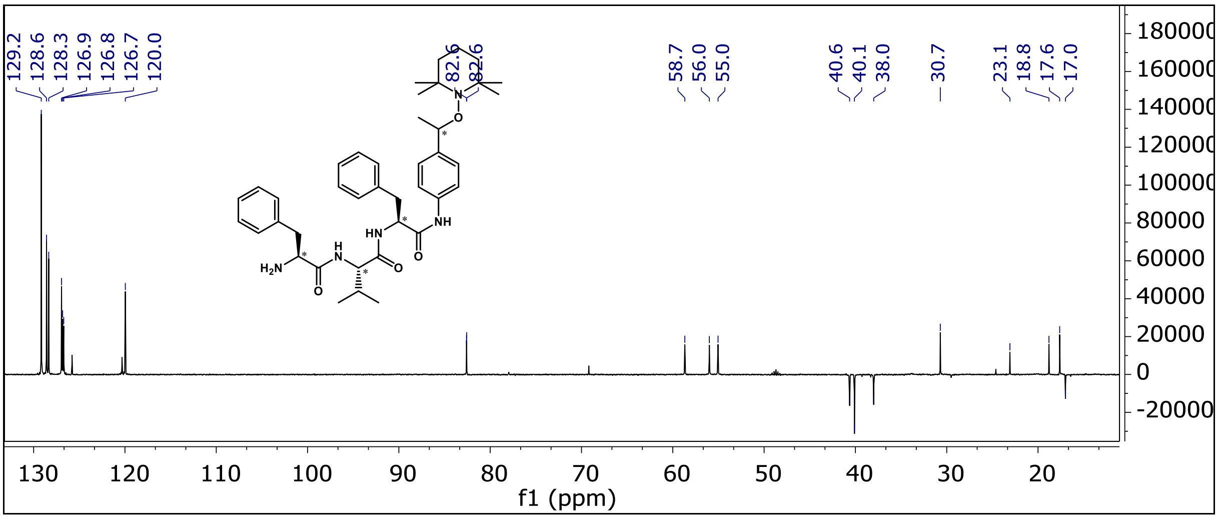

HRMS (ESI)

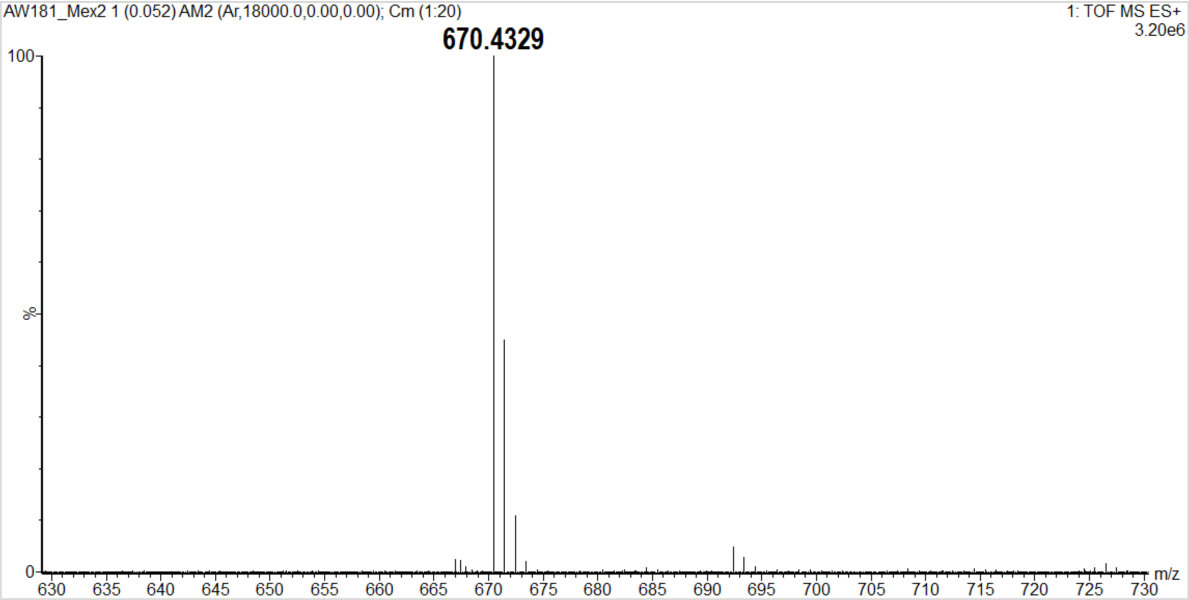
AW181_Mex2 1 (0.052) AM2 (Ar,18000.0,0.00,0.00); Cm (1:20)
1: TOF MS ES+
3.20e6
670.4329
100
%
0
630
635
640
645
650
655
660
665
670
675
680
685
690
695
700
705
710
715
720
725
730
m/z

benzyl 4-(((2S)-1-(((2S)-3-methyl-1-oxo-1-(((2S)-1-oxo-3-phenyl-1-((4-(1-((2,2,6,6-tetramethylpiperidin-1-yl)oxy)ethyl)phenyl)amino)propan-2-yl)amino)butan-2-yl)amino)-1-oxo-3-phenylpropan-2-yl)amino)-4-oxobutanoate

(A6L)

^{1}H NMR (400 MHz, MeOD)

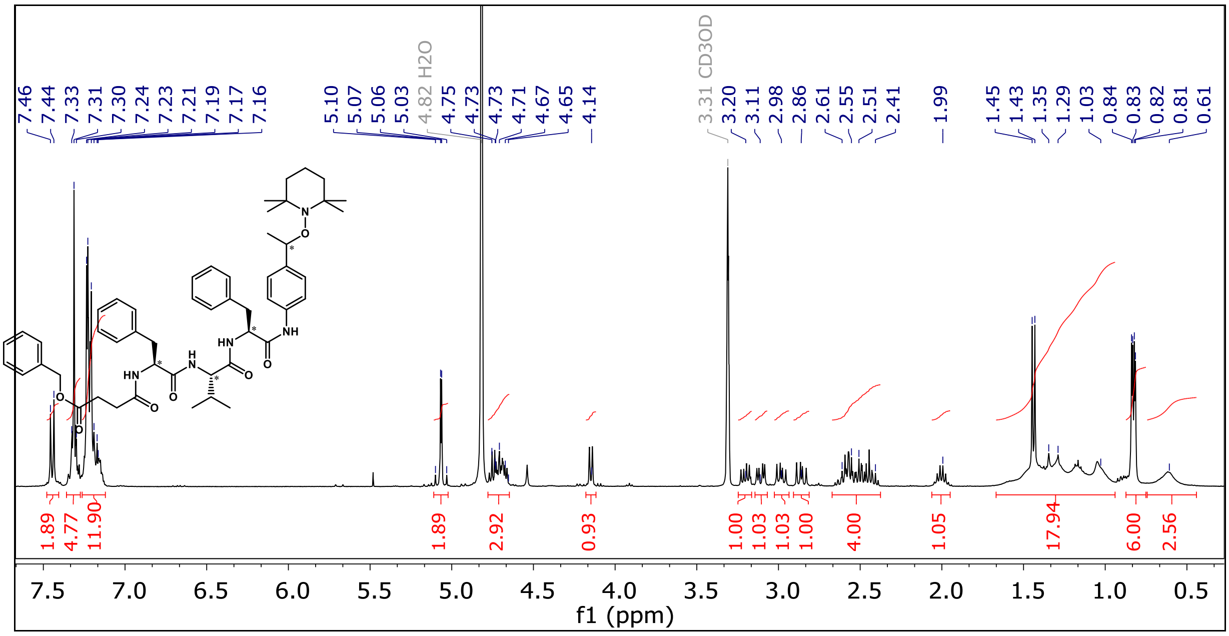

^{13}C NMR (101 MHz, $CDCl_3$ / C_6D_6 : 19 : 1)

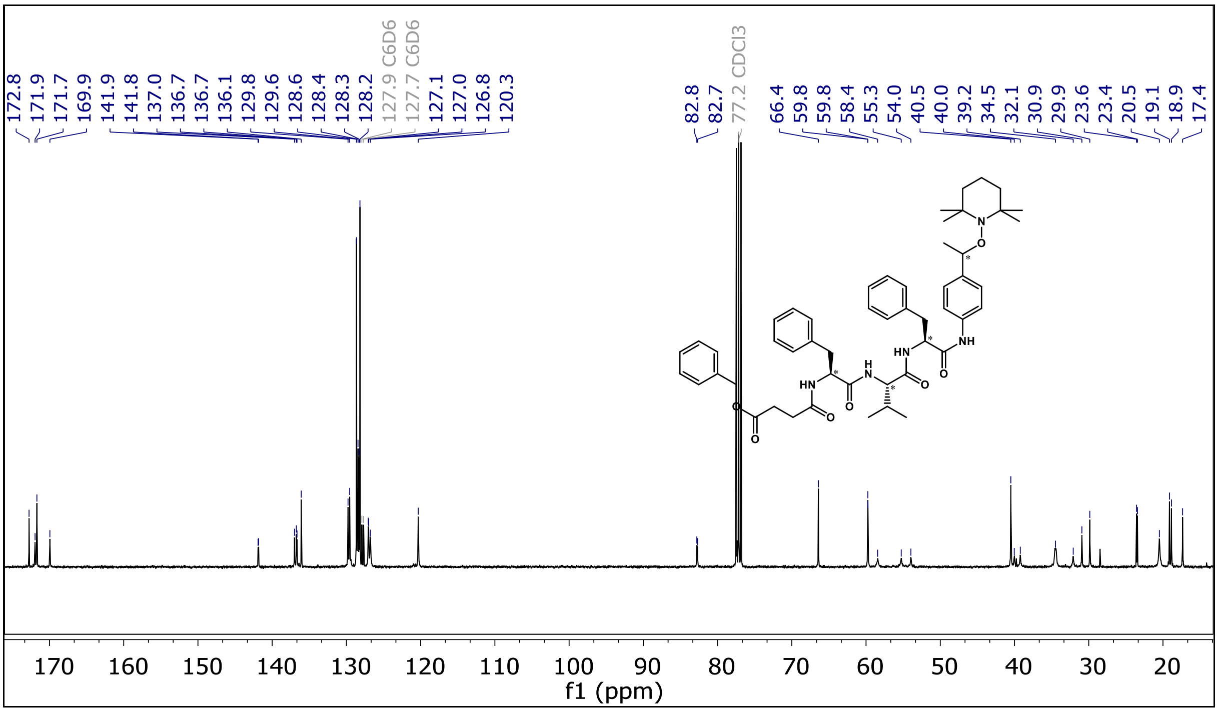

DEPT 135 (101 MHz, $CDCl_3$ / C_6D_6 : 19 : 1)

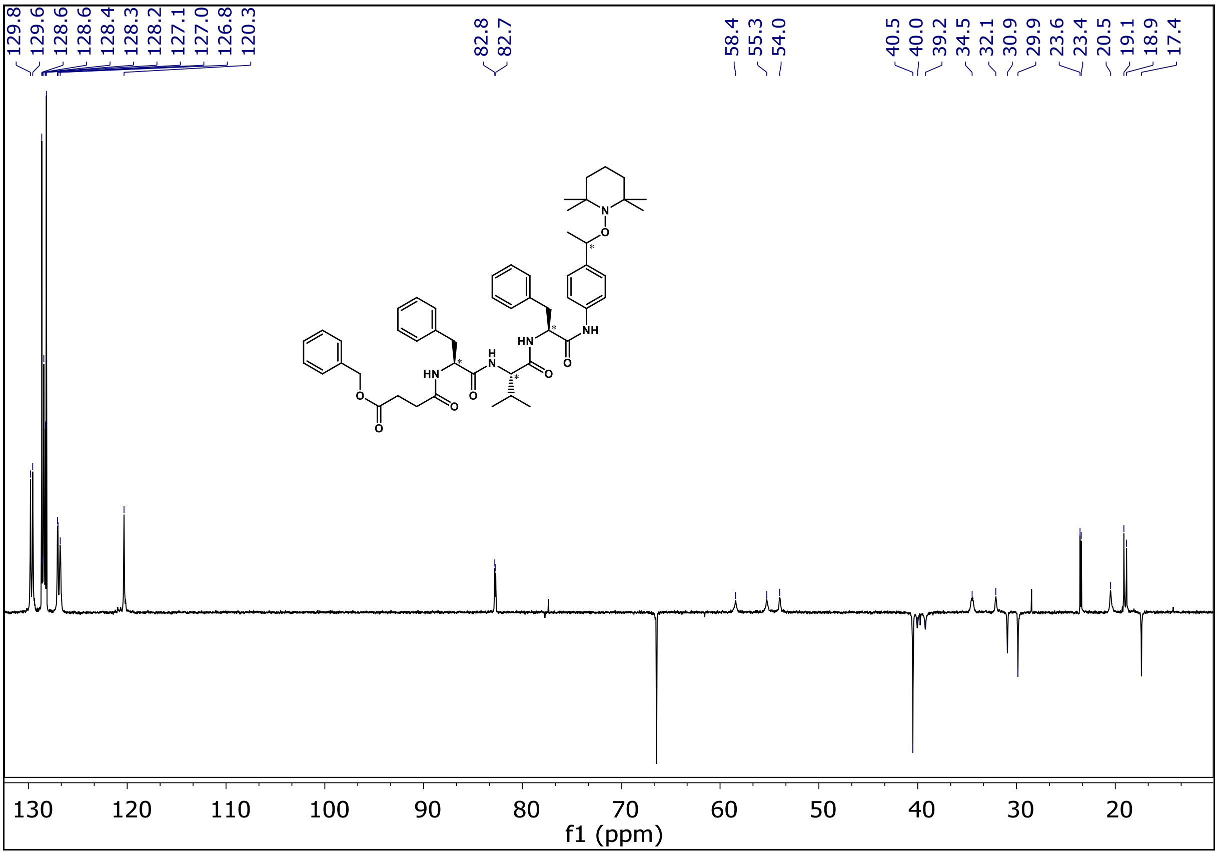

HRMS (ESI)

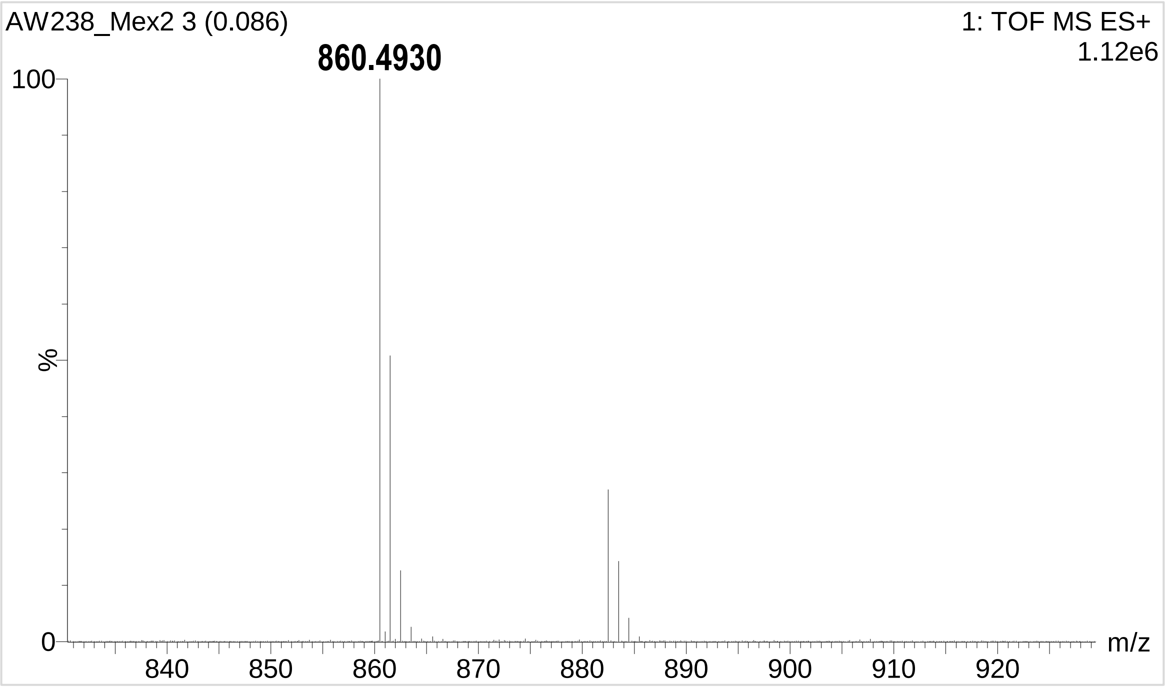

4-(((2S)-1-(((2S)-3-methyl-1-oxo-1-(((2S)-1-oxo-3-phenyl-1-((4-(1-((2,2,6,6-tetramethylpiperidin-1-yl)oxy)ethyl)phenyl)amino)propan-2-yl)amino)butan-2-yl)amino)-1-oxo-3-phenylpropan-2-yl)amino)-4-oxobutanoic acid

(A7L)

^{1}H NMR (400 MHz, MeOD)

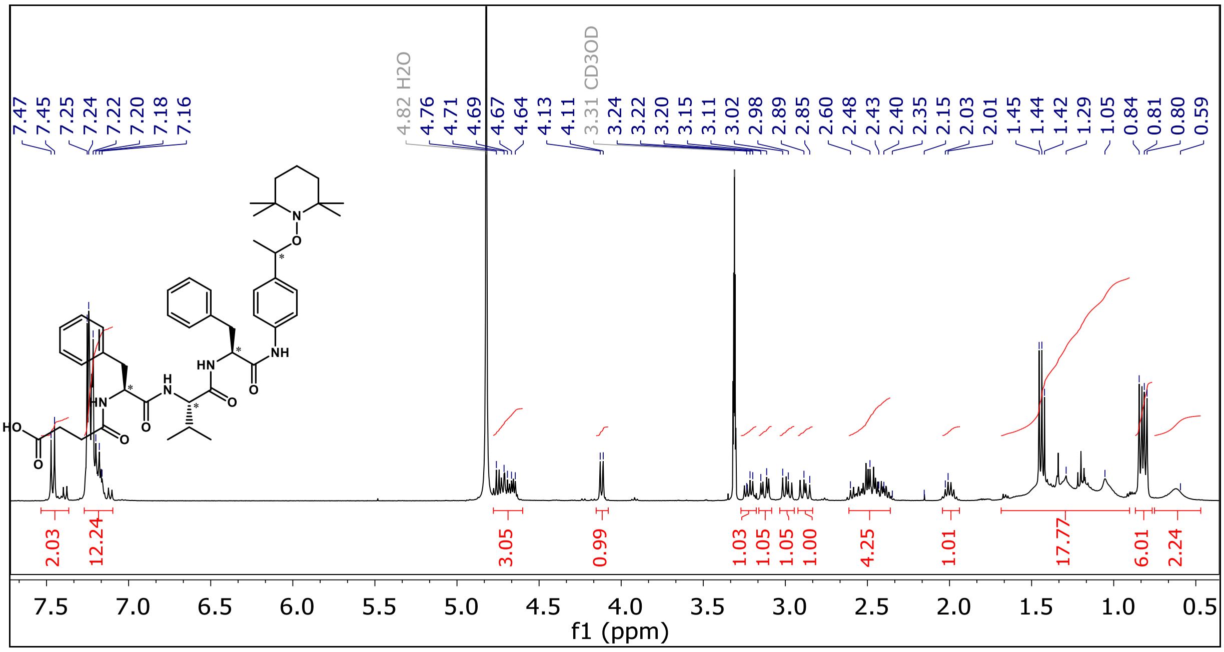

^{13}C NMR (101 MHz, MeOD-$CDCl_3$ (1 : 1 ; v : v))

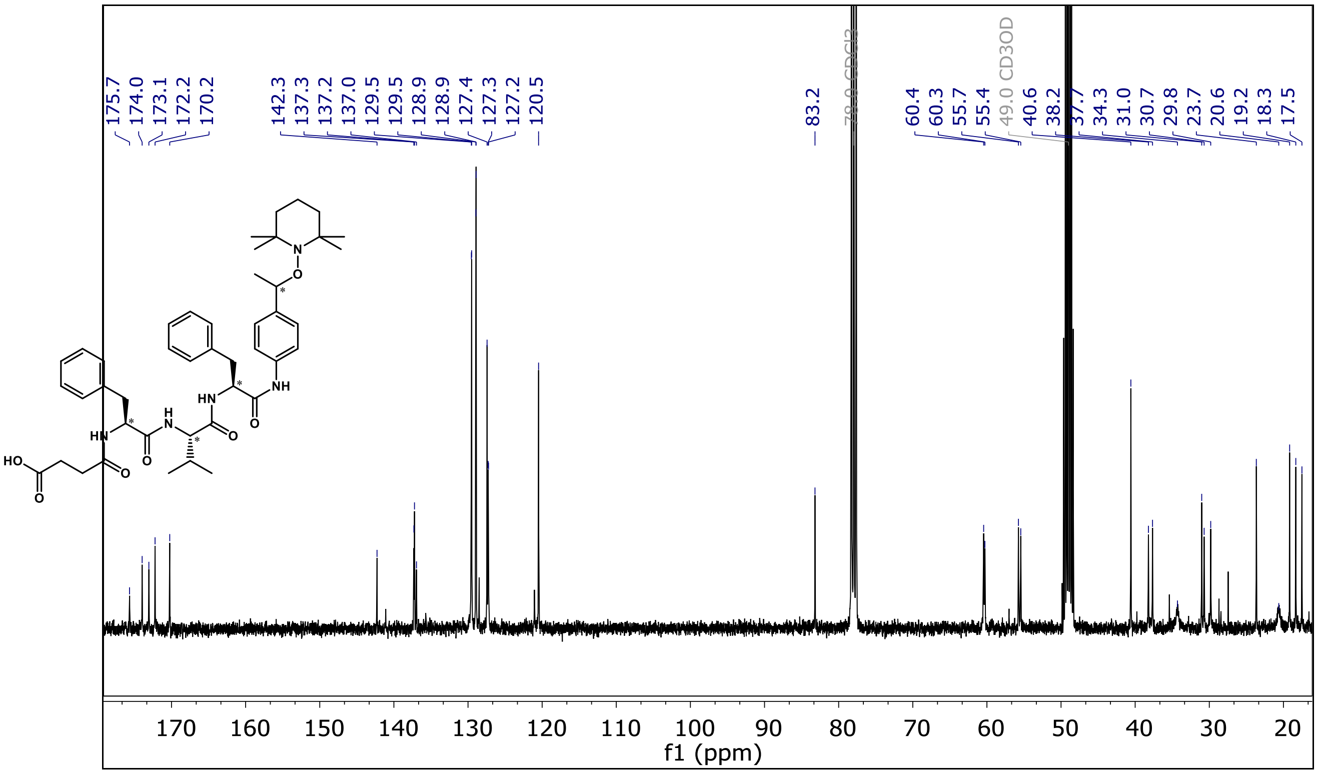

DEPT 135 (101 MHz, MeOD-$CDCl_3$ (1 : 1 ; v : v))

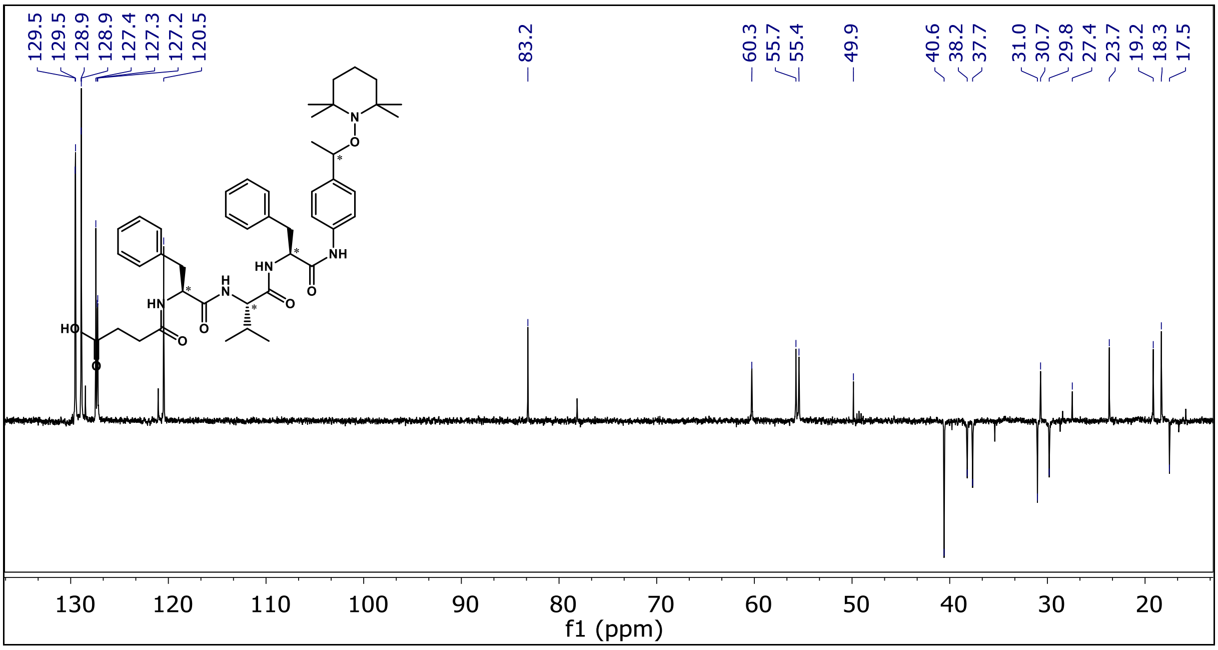
129.5
129.5
128.9
128.9
127.4
127.3
127.2
120.5
83.2
60.3
55.7
55.4
49.9
40.6
38.2
37.7
31.0
30.7
29.8
27.4
23.7
19.2
18.3
17.5
130 120 110 100 90 80 70 60 50 40 30 20
f1 (ppm)

HRMS (ESI)

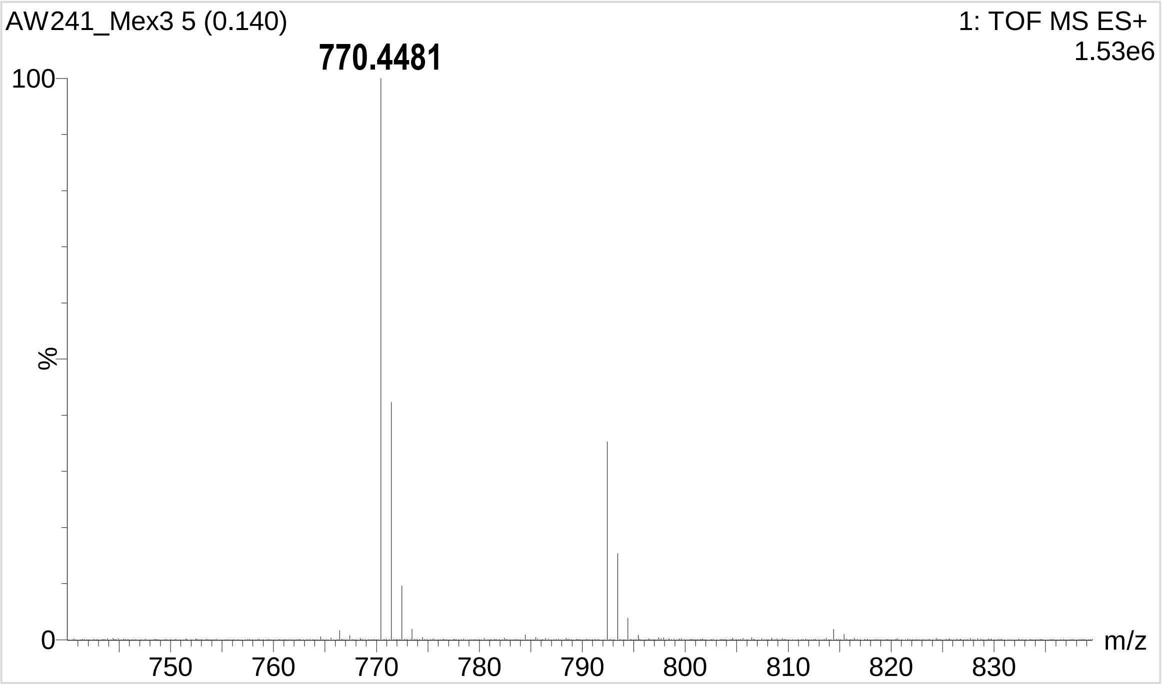
AW241_Mex3 5 (0.140)
1: TOF MS ES+
1.53e6
770.4481
100
%
0
750 760 770 780 790 800 810 820 830
m/z

tert-butyl ((2R)-1-oxo-3-phenyl-1-((4-(1-((2,2,6,6-tetramethylpiperidin-1-yl)oxy)ethyl)phenyl)amino)propan-2-yl)carbamate

(A1D)

Chiral HPLC report

Heptane/ethanol/dichloromethane (80/10/10), 1 mL/min

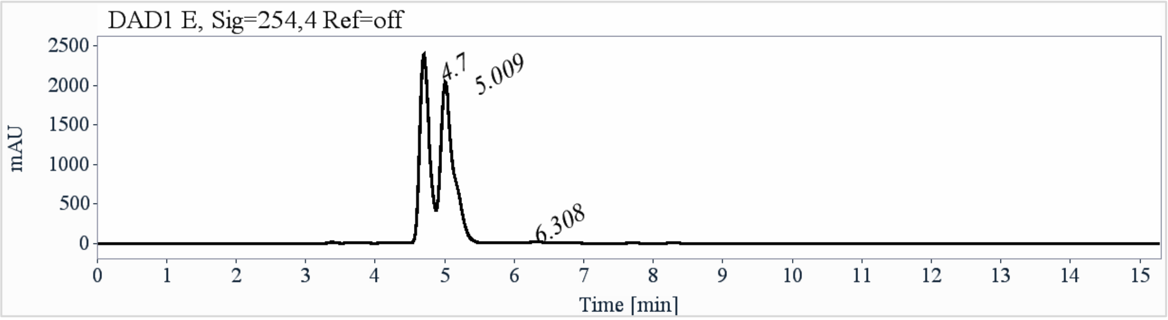

Diastereomers have retention times too close (around 5 min). their separation is difficult.

^{1}H NMR (400 MHz, $CDCl_3$)

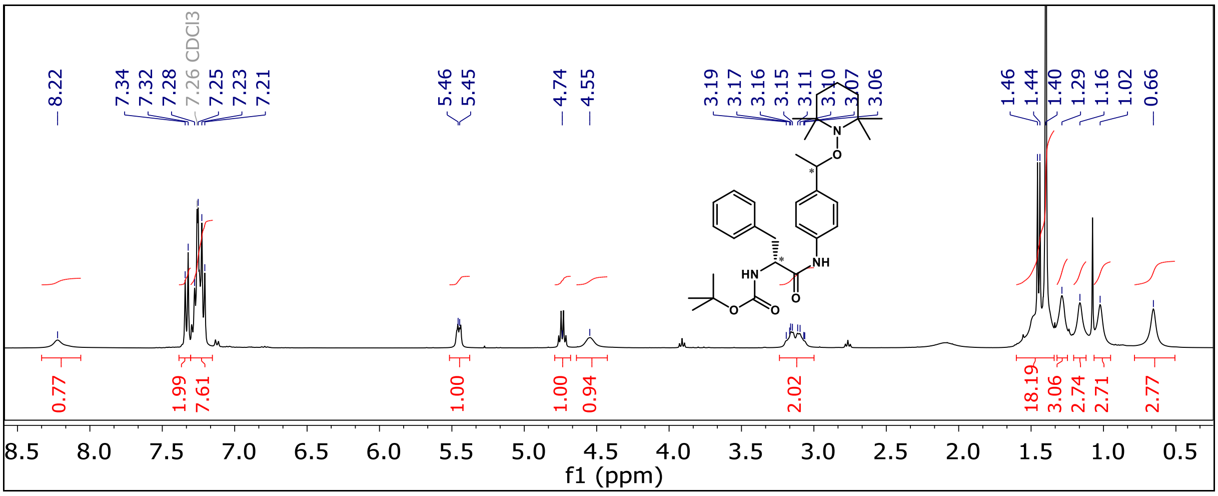

^{13}C NMR (101 MHz, $CDCl_3$)

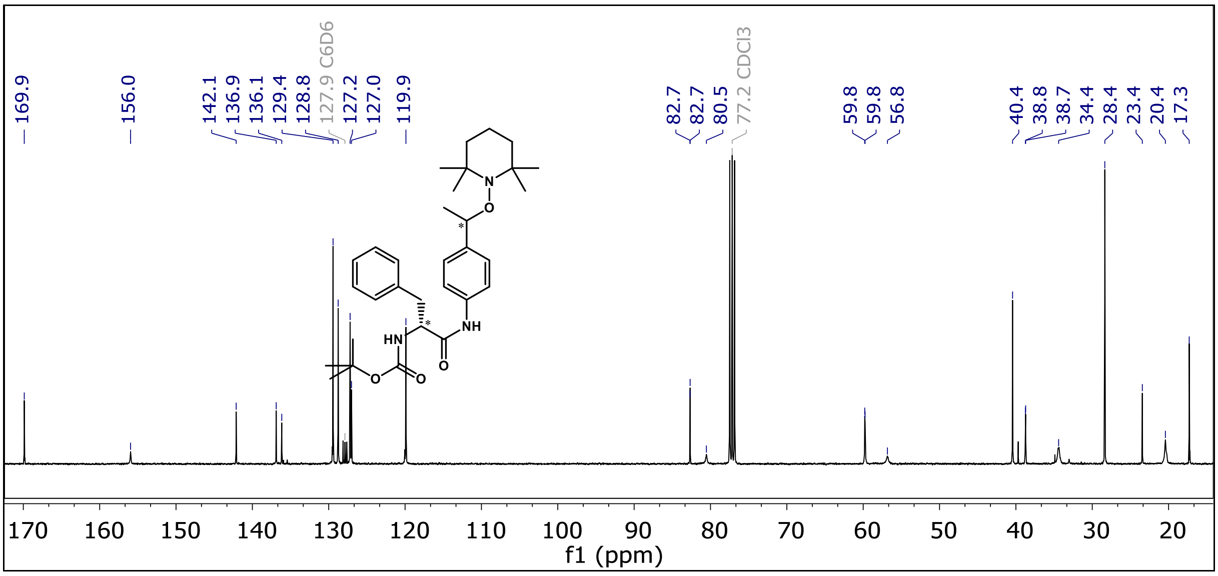

DEPT 135 (101 MHz, $CDCl_3$)

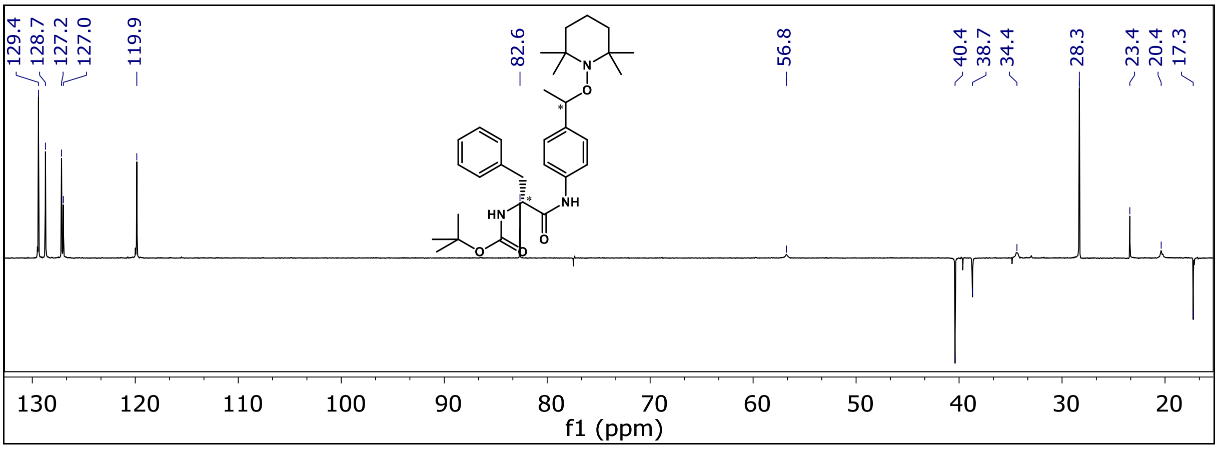

HRMS (ESI)

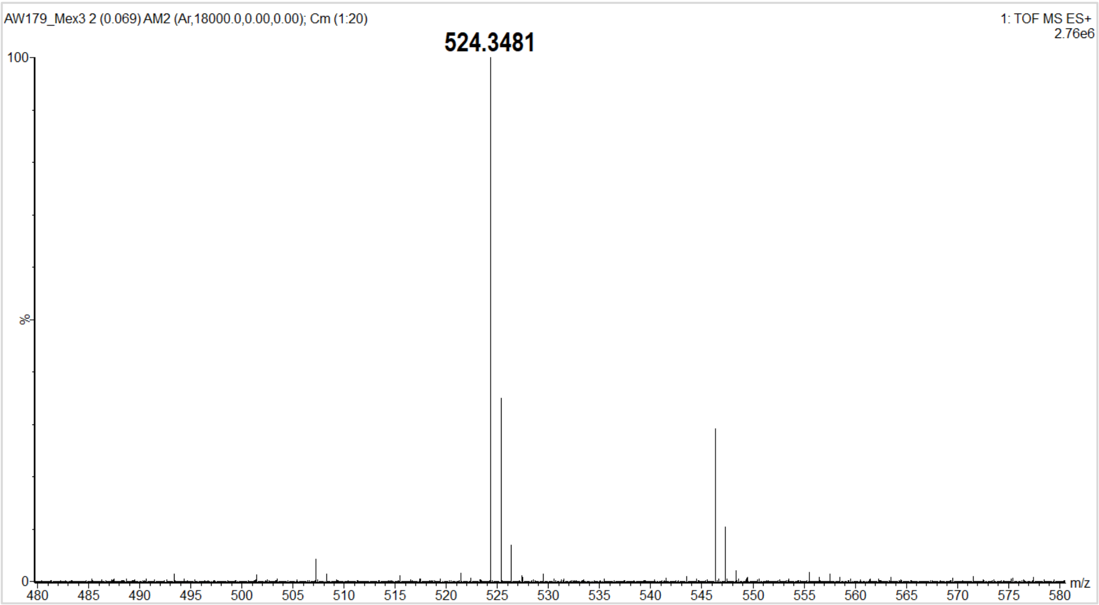

tert-butyl ((2R)-1-(((2R)-3-methyl-1-oxo-1-(((2R)-1-oxo-3-phenyl-1-((4-(1-((2,2,6,6-tetramethylpiperidin-1-yl)oxy)ethyl)phenyl)amino)propan-2-yl)amino)butan-2-yl)amino)-1-oxo-3-phenylpropan-2-yl)carbamate

(A3D)

Chiral HPLC report

Heptane/Ethanol/dichloromethane (60/20/20), 1 mL/min

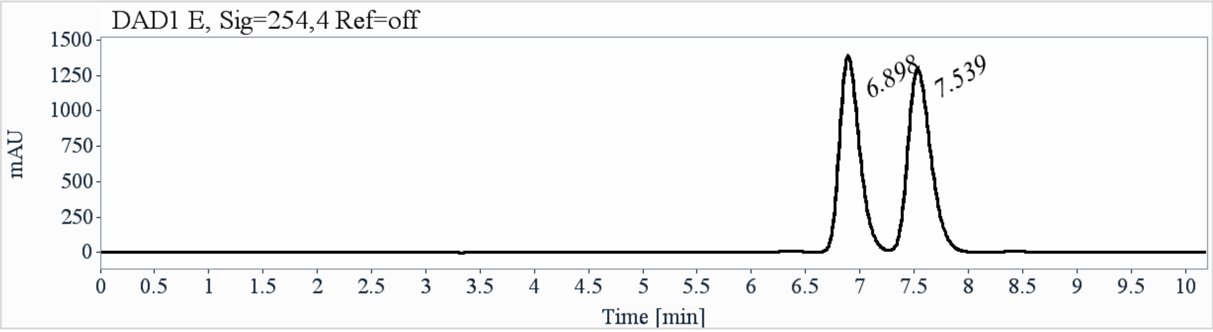

Diastereomers have retention times too close (around 7 min).

^{1}H NMR (400 MHz, MeOD)

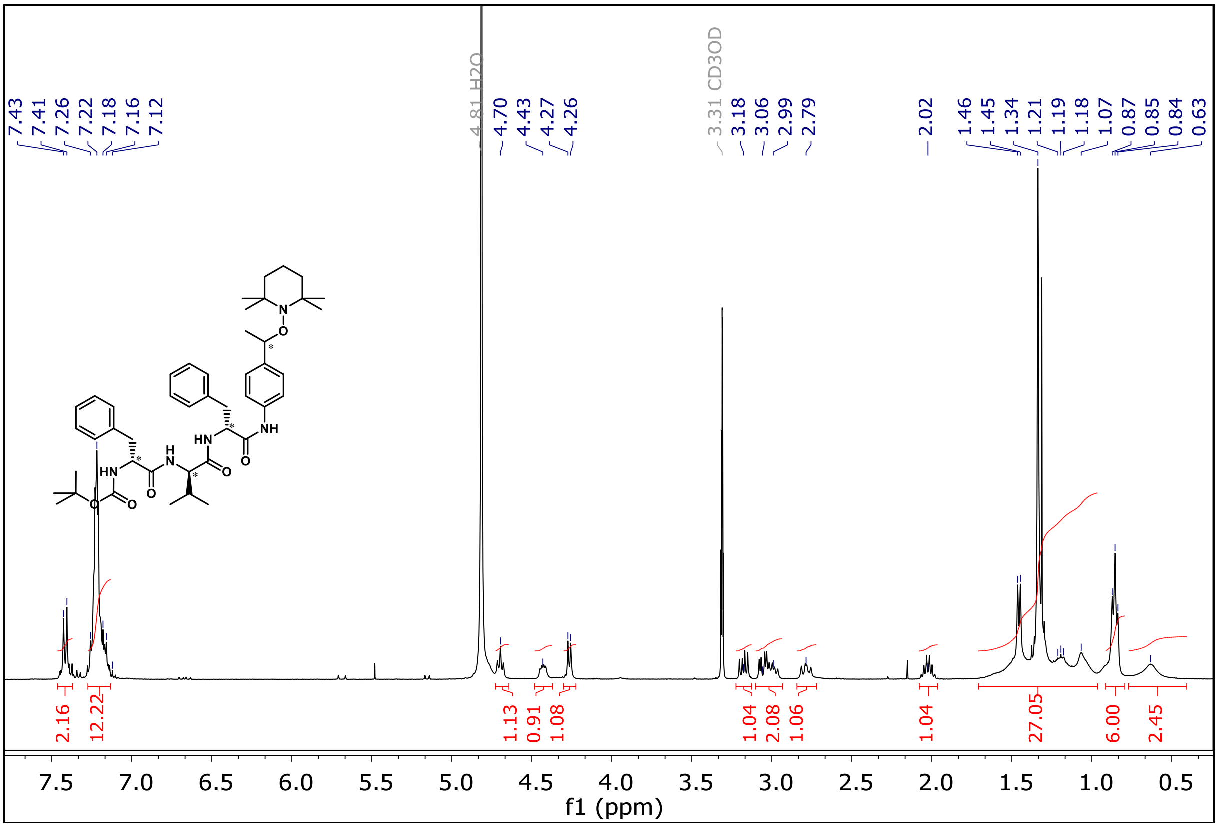

^{13}C NMR (101 MHz, $CDCl_3$)

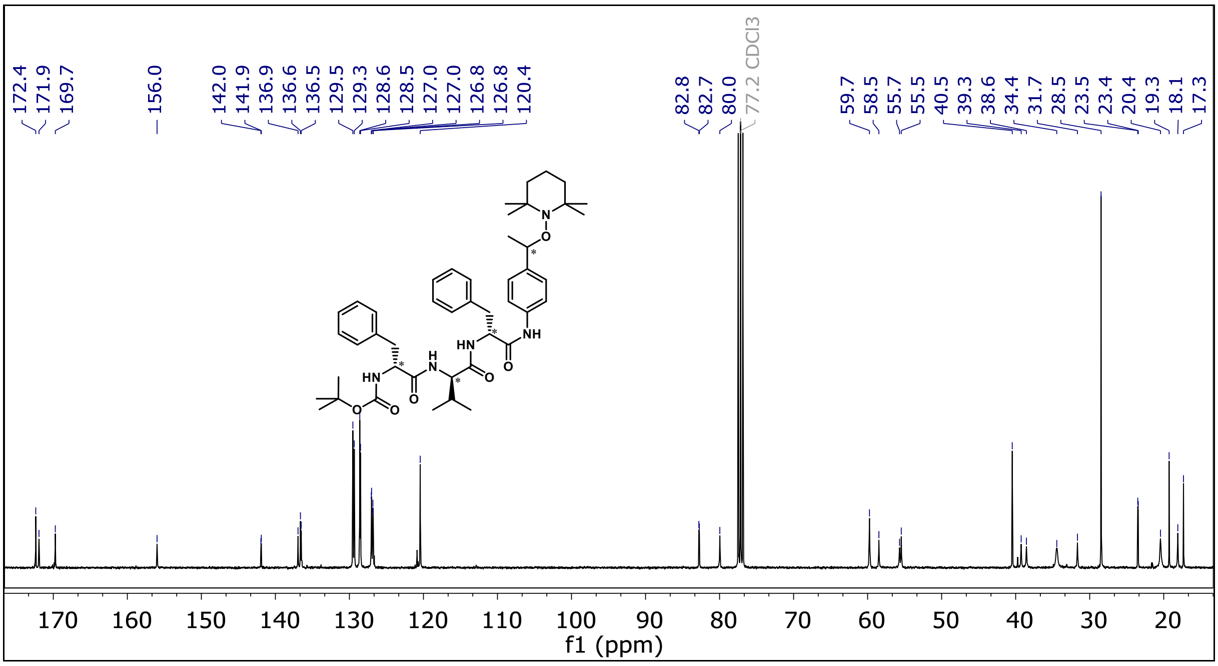

DEPT 135 (101 MHz, $CDCl_3$)

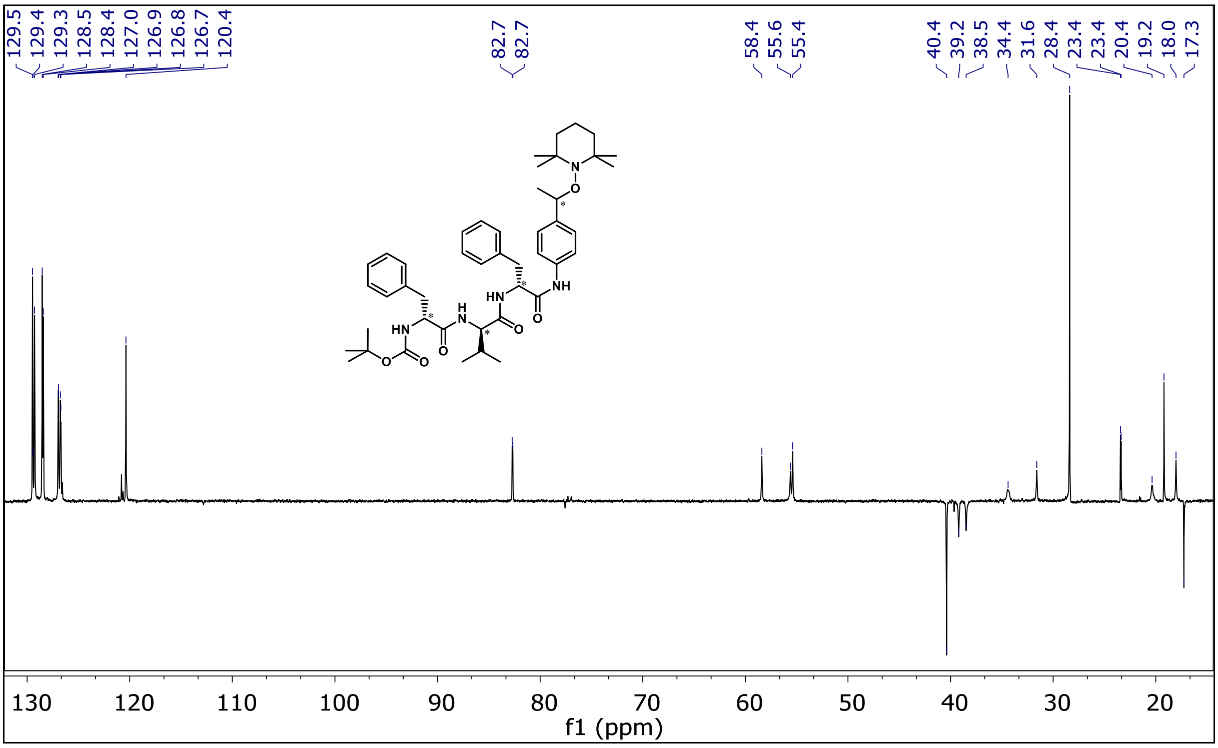

HRMS (ESI)

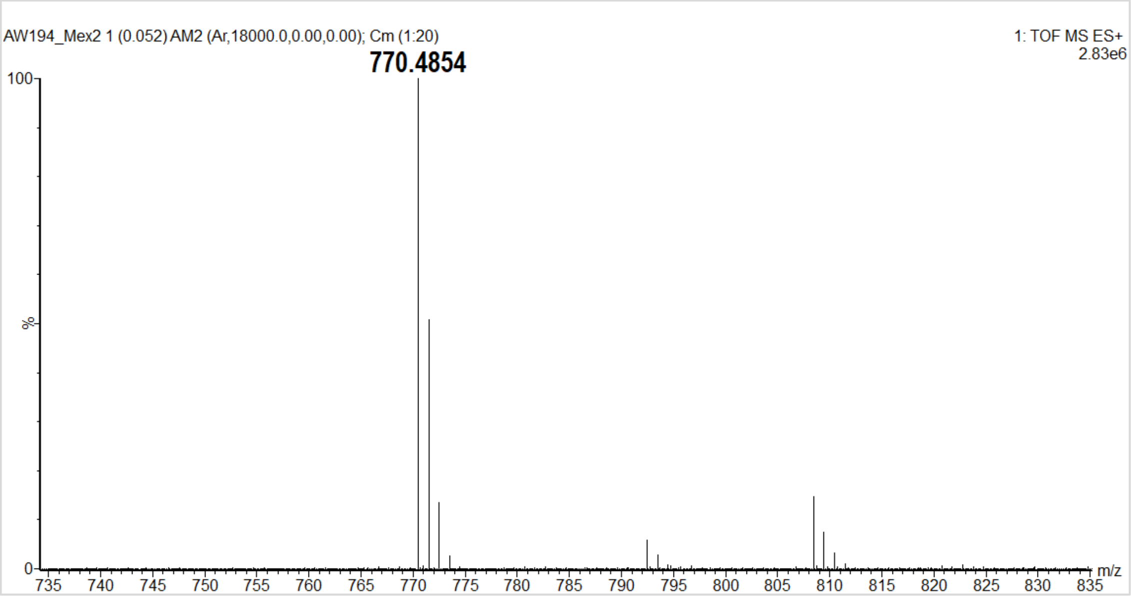

(2R)-1-(((2R)-3-methyl-1-oxo-1-(((2R)-1-oxo-3-phenyl-1-((4-(1-((2,2,6,6-tetramethylpiperidin-1-yl)oxy)ethyl)phenyl)amino)propan-2-yl)amino)butan-2-yl)amino)-1-oxo-3-phenylpropan-2-aminium trifluoroacetate

(A4D)

^{1}H NMR (400 MHz, MeOD)

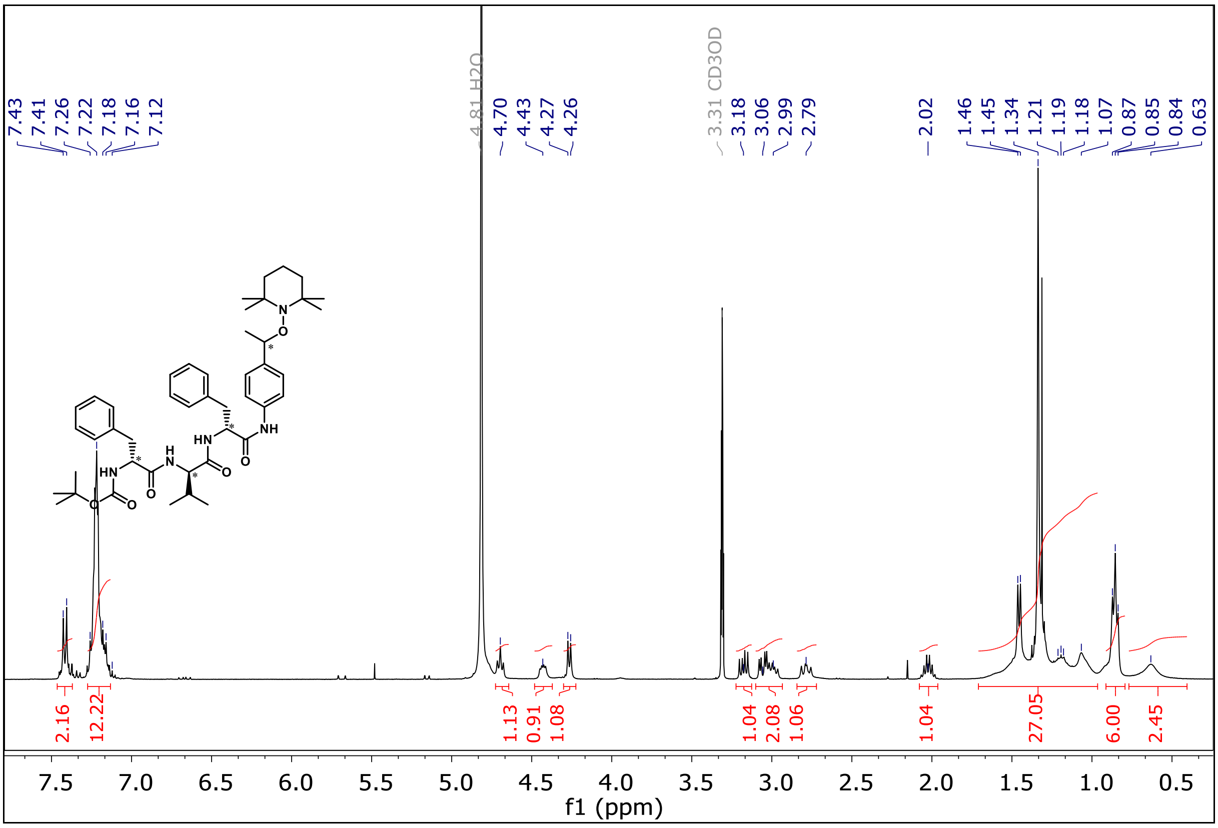

^{13}C NMR (101 MHz, $CDCl_3$)

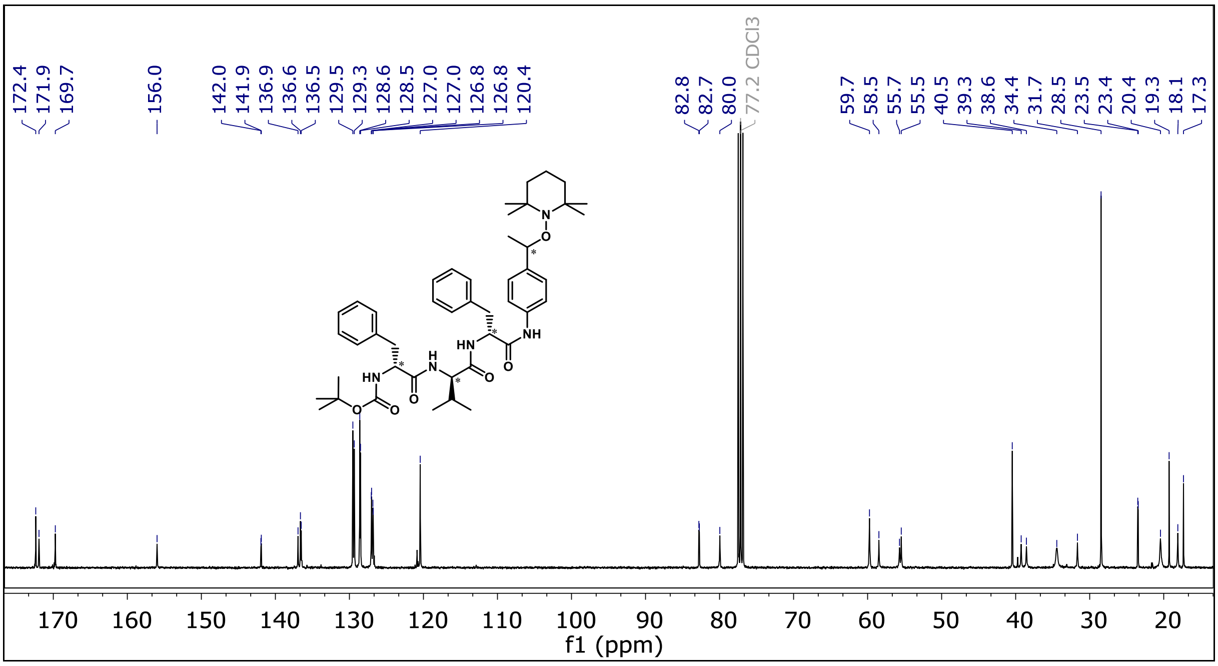

DEPT 135 (101 MHz, $CDCl_3$)

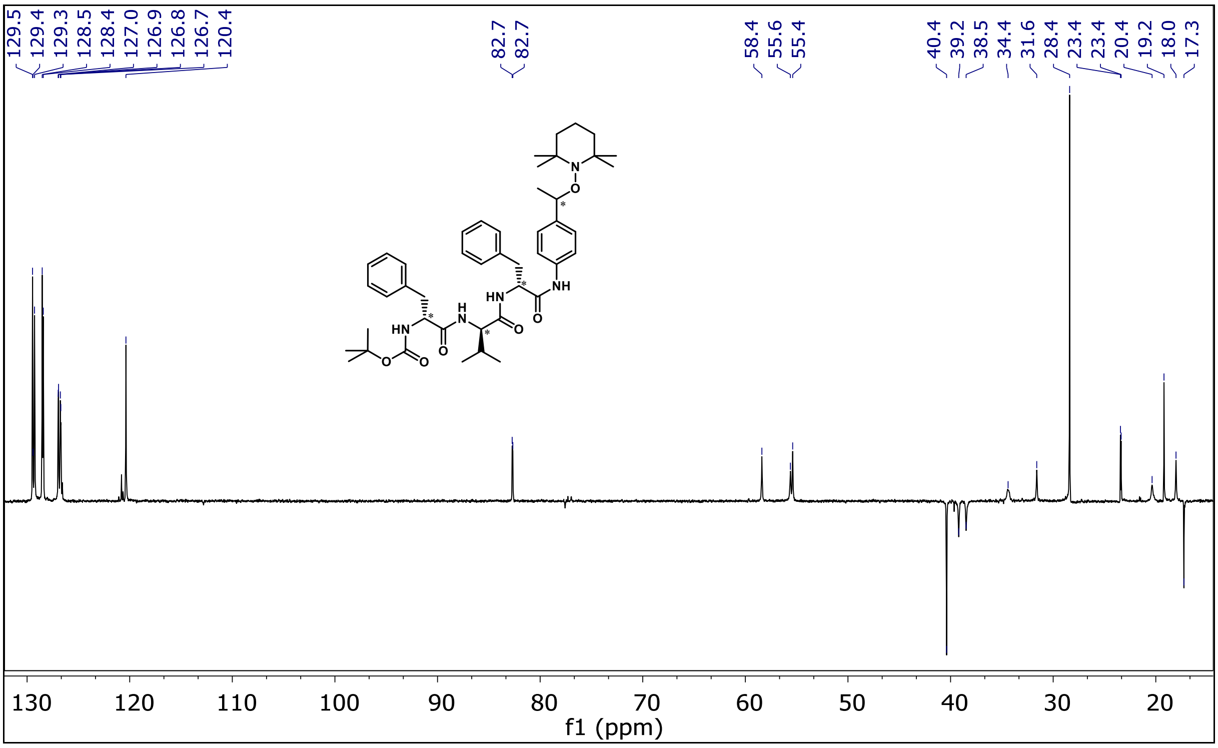

HRMS (ESI)

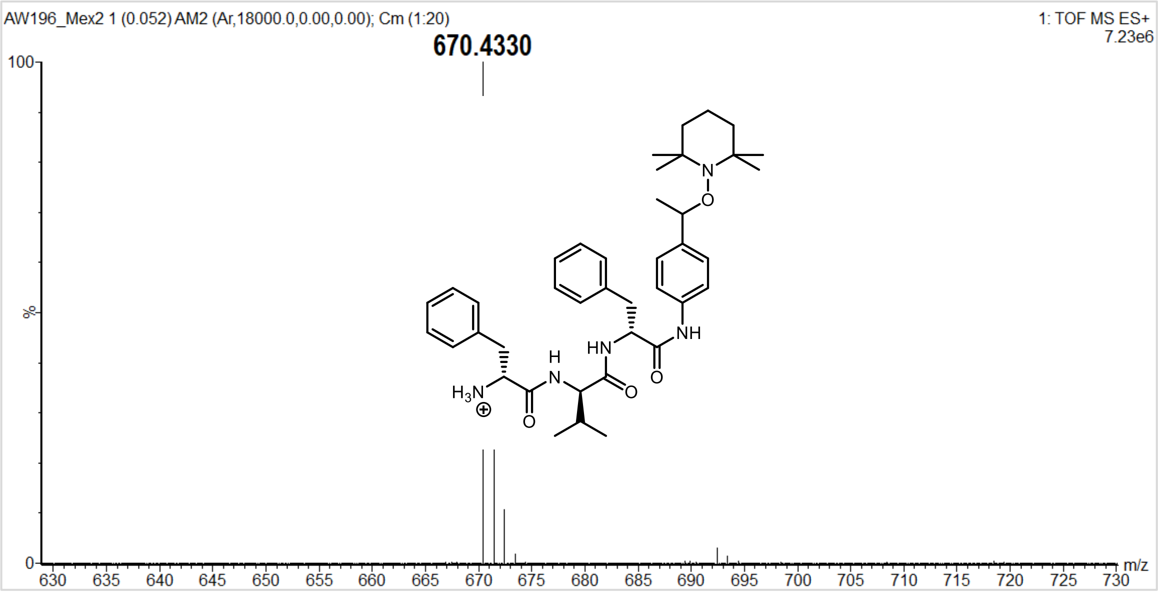

(2R)-2-((R)-2-amino-3-phenylpropanamido)-3-methyl-N-((2R)-1-oxo-3-phenyl-1-((4-(1-((2,2,6,6-tetramethylpiperidin-1-yl)oxy)ethyl)phenyl)amino)propan-2-yl)butanamide

(A5D)

^{1}H NMR (400 MHz, MeOD)

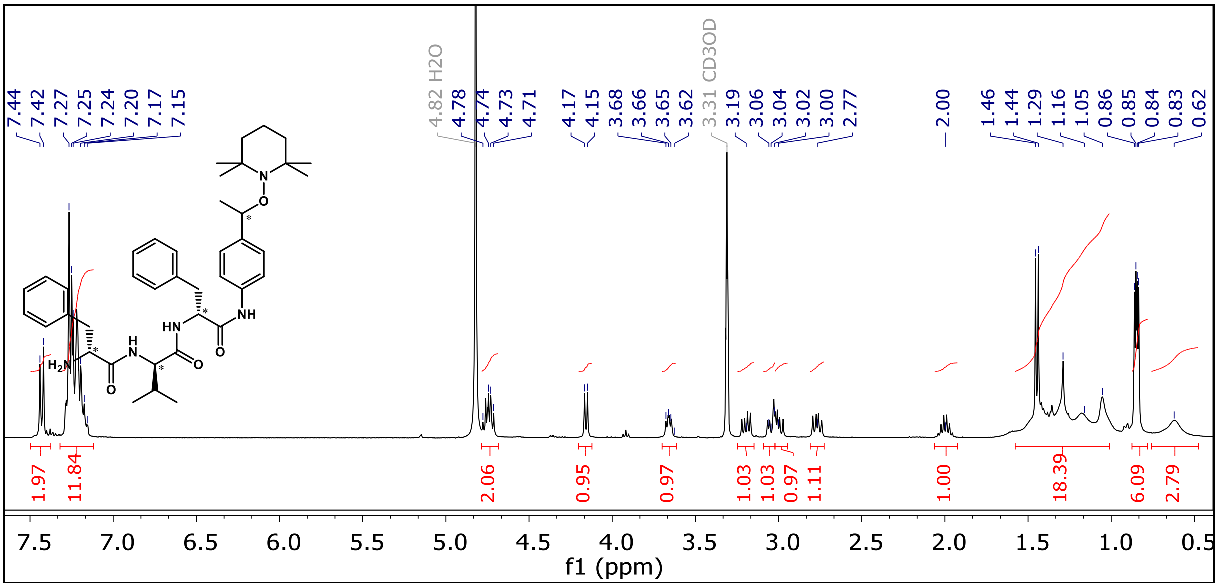

^{13}C NMR (101 MHz, MeOD)

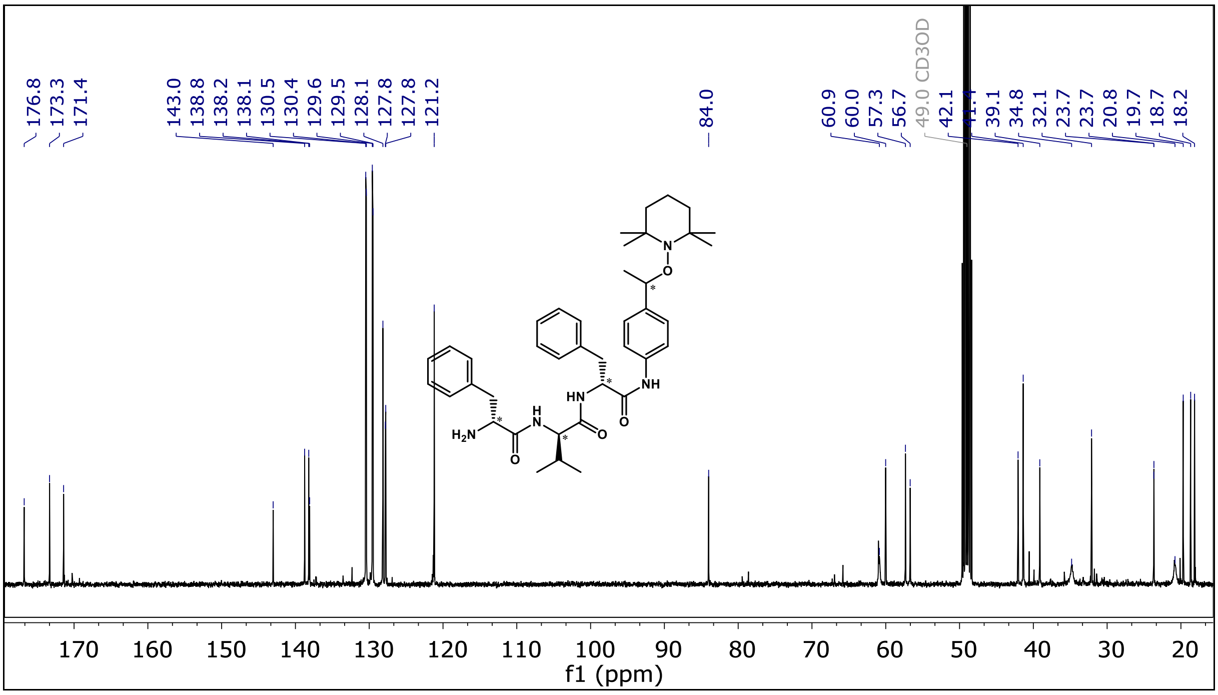

DEPT 135 (101 MHz, MeOD)

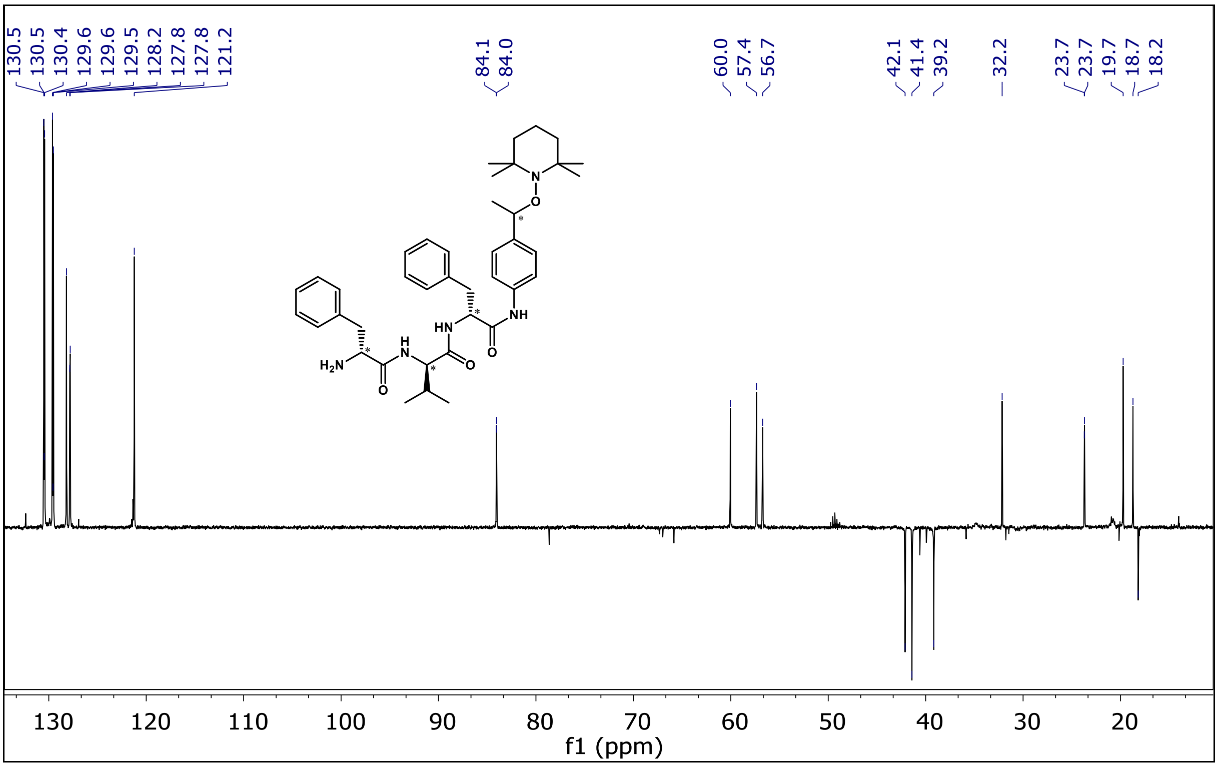

HRMS (ESI)

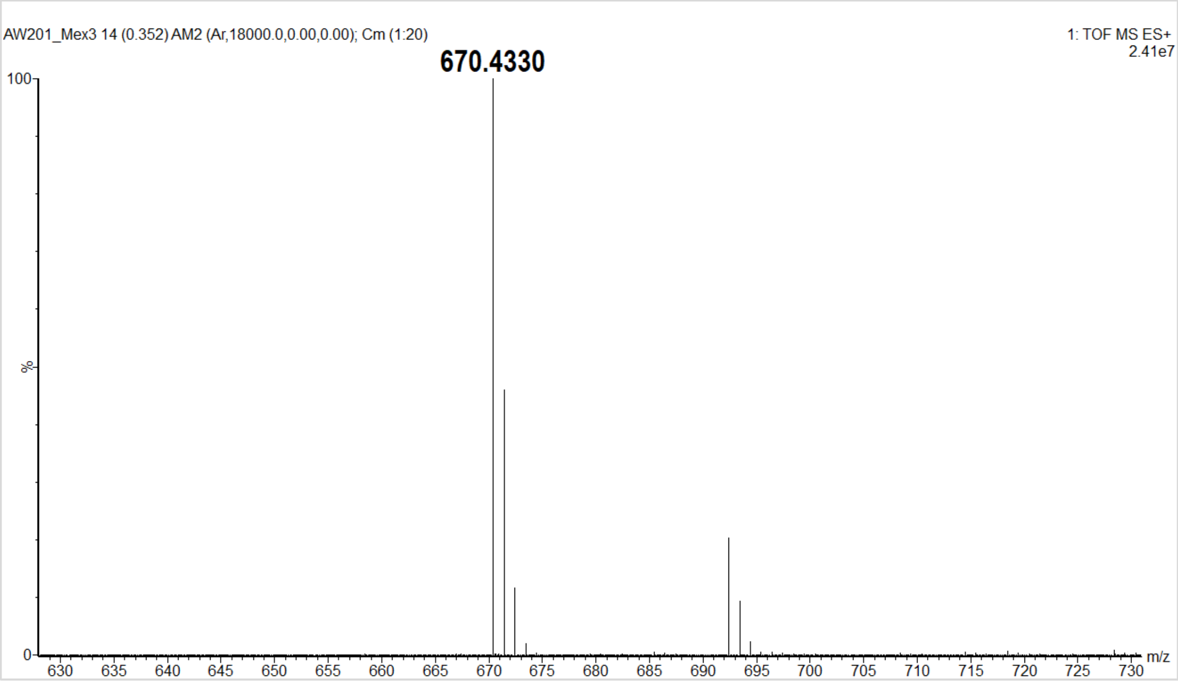
AW201_Mex3 14 (0.352) AM2 (Ar,18000.0,0.00,0.00); Cm (1:20)
1: TOF MS ES+
2.41e7
670.4330
100
%
0
630
635
640
645
650
655
660
665
670
675
680
685
690
695
700
705
710
715
720
725
730
m/z

HPLC of hybrid-alkoxyamines

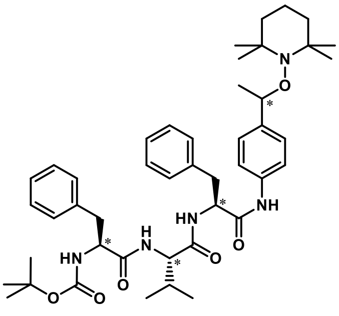

A3L

Chiral HPLC report

Heptane/ethanol/dichloromethane (80/10/10), 1 mL/min

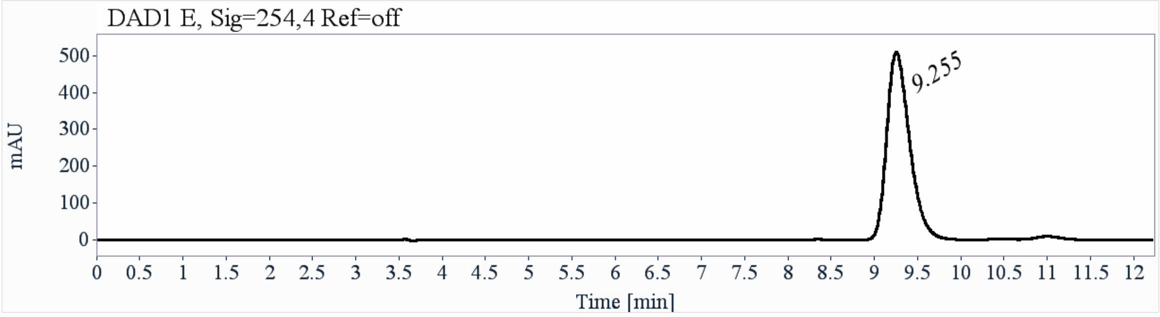

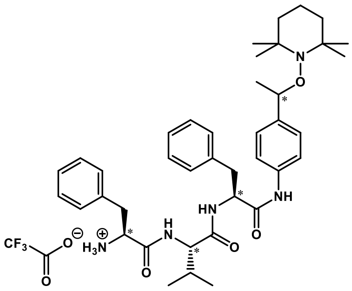

A4L

Chiral HPLC report

Heptane/Ethanol/dichloromethane (60/20/20), 1 mL/min

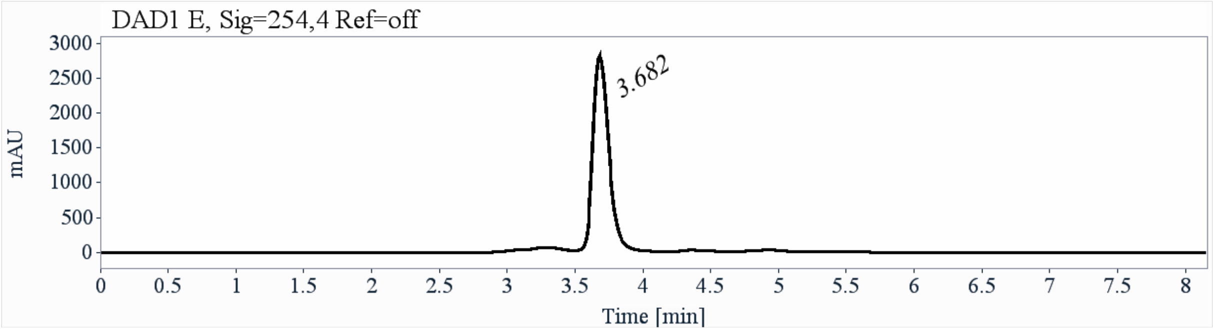

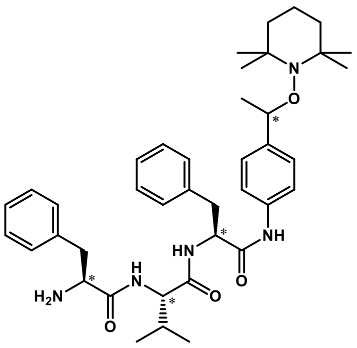

A5L

Chiral HPLC report

Heptane/Ethanol/dichloromethane (60/20/20), 1 mL/min

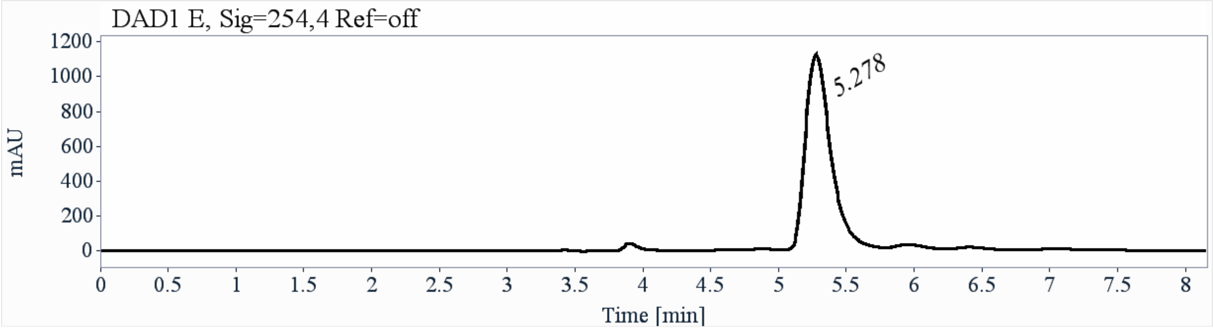

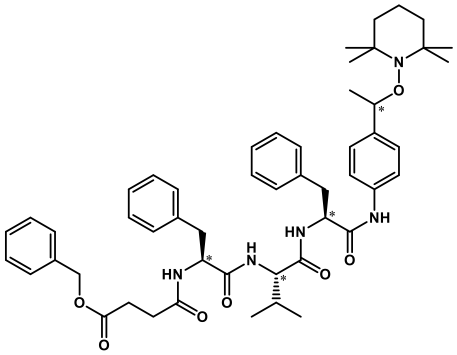

A6L

Chiral HPLC report

Heptane/Ethanol/dichloromethane (60/20/20), 1 mL/min

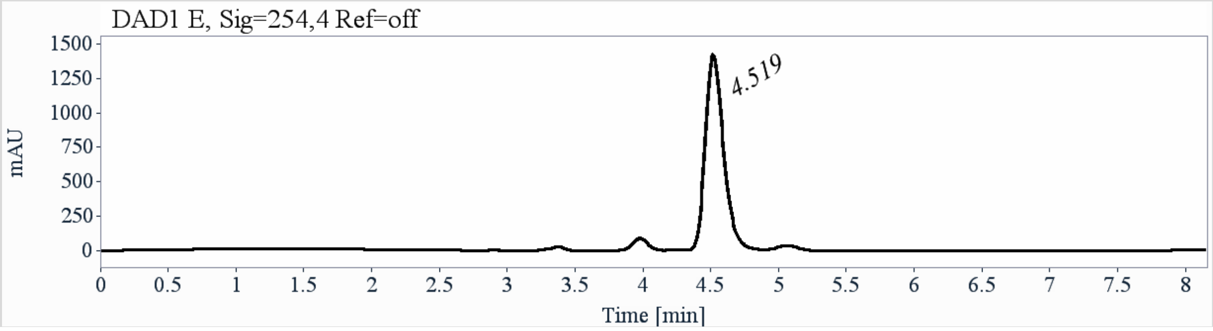

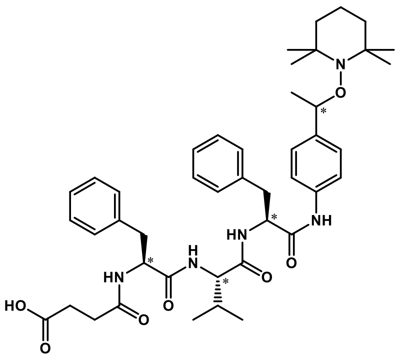

A7L

Chiral HPLC report

Heptane/Ethanol/dichloromethane (60/20/20), 1 mL/min

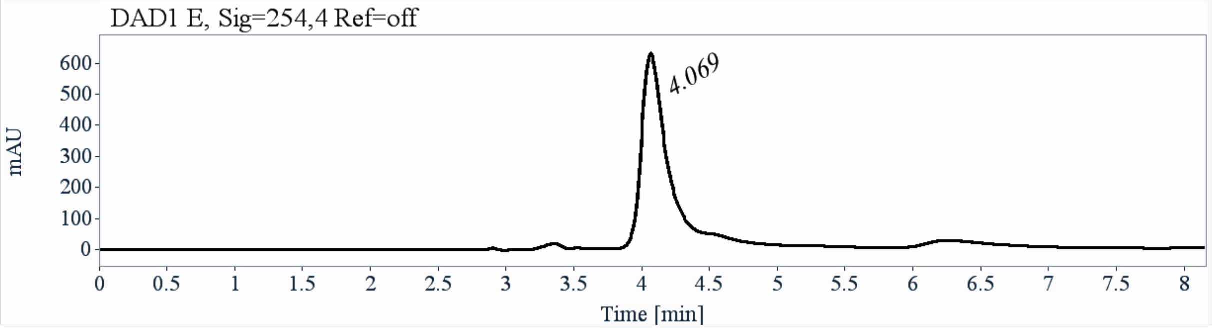

Kinetics and homolysis constants of alkoxyamines A3L

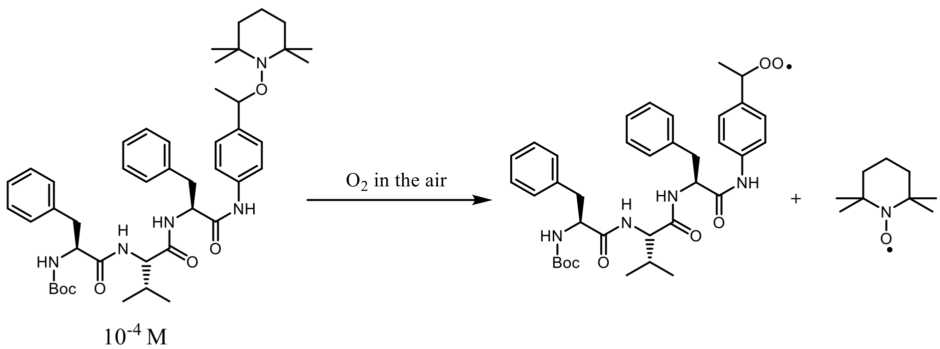

10^{-4} M

Solvent : tBuPh, temperature : 125 °C, k_d = 8.4 x 10^{-4} s^{-1} .

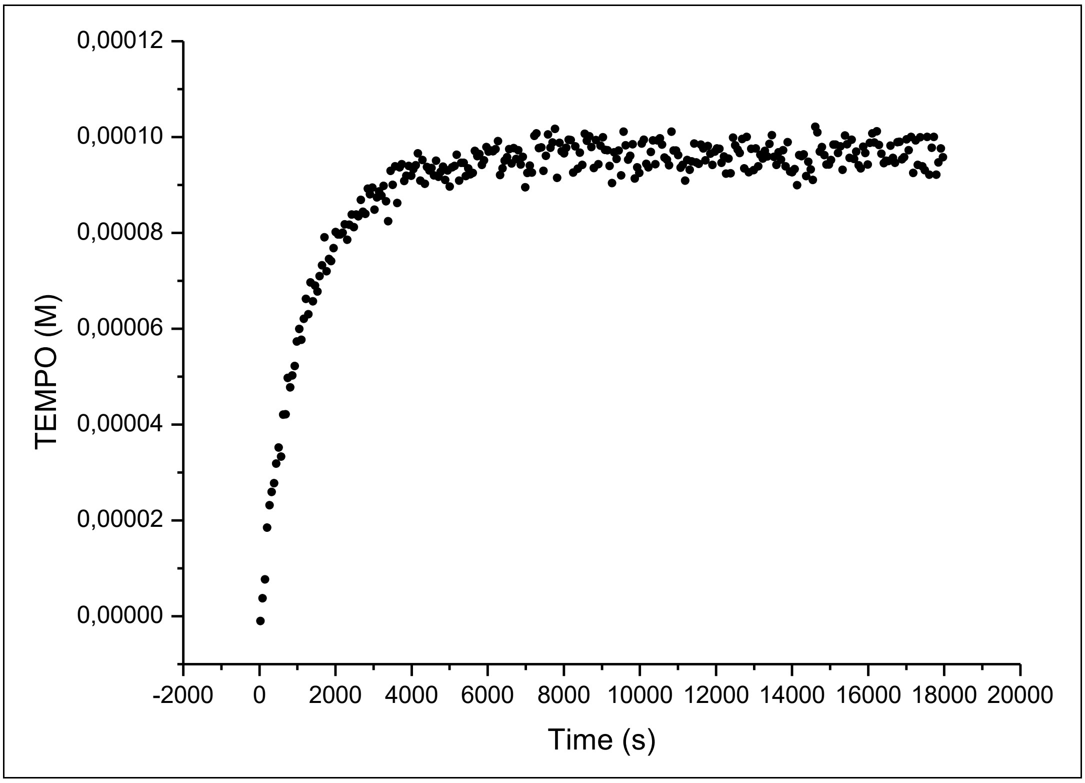

Table S1. Doses of alkoxyamines provided to *Schistosoma* at 37 °C, time to reach the death of half of the population of worms, time to kill all parasites, and their cytotoxicity on Vero cells (CC_{50} in µM).

	Dose at 100 µg/ml[a]		CC_{50} (µM)[b]
**P6L**	NA	NA	> 50
**P8L**	7	24	> 50
**P9L**	3	5	> 50
**A3D**	NA	NA	> 50
**A4D**	2	7	2.6 ± 0.3[c]
**A5D**	1	4	2.2 ± 0.3
**P6D**	4	24	3.2
**P8D**	1	1	> 50
**P21D**	5	24	> 50
**P22D**	4	24	11

a. NA: No Activity. Concentrations span from 100 to 200 µM for alkoxyamines, and from 200 to 400 µM for peptides. [b] Cytotoxicity values correspond to the mean of 2 to 7 independant experiments [c] Correspond to the mean of 7 independant experiments. [d] data from reference [Sun, Q.; Mao, R.; Wang, D.; Hu, C.; Zheng, Y.; Sun, D. The cytotoxicity study of praziquantel enantiomers. *Drug Des. Dev. Ther.* **2016**, *10*, 2061–2068].

Table S2 **:** Biological Testing: Raw Data.

molecule	[C] µg/mL	0	1	2	3	4	5	6	7	8	24	48	72
P21L	100	27	27	27	27	27	27	27	27	27	-	-	-
	10	29	29	29	29	29	29	29	29	29	-	-	-
P6L	100	30	30	30	30	30	30	30	30	30	-	-	-
	10	27	27	27	27	27	27	27	27	27	-	-	-
P7L	100	43	43	43	43	43	43	43	43	43	-	-	-
	10	28	28	28	28	28	28	28	28	28	-	-	-
A3L	100	39	39	39	39	39	39	39	38	38	38	38	38
	10	42	42	42	42	42	42	42	42	42	42	42	42
A4L	100	35	19	17	14	7	7	7	4	4	0	0	0
	10	26	26	26	26	26	26	26	26	26	26	26	26
A3D	100	30	30	30	30	30	30	30	28	28	28	28	28
	10	28	28	28	28	28	28	28	28	28	28	28	28
A4D	100	32	29	17	10	3	3	3	0	0	0	0	0
	10	27	27	27	27	27	27	27	27	27	27	27	27
A5L	100	31	31	15	10	2	2	2	0	0	0	0	0
	10	29	29	29	29	29	29	29	29	29	29	29	29
A5D	100	29	13	4	2	0	0	0	0	0	0	0	0
	10	33	33	33	33	33	33	33	33	30	30	30	30
P7D	100	26	26	26	26	26	26	26	26	26	26	26	26
	10	21	21	21	21	21	21	21	21	21	21	21	21
P11L	100	26	26	26	26	26	26	26	26	26	26	26	26
	10	28	28	28	28	28	28	28	28	28	28	28	28

P11D	100	22	22	22	22	22	22	22	22	22	22	22	22
	10	19	19	19	19	19	19	19	19	19	19	19	19
P12L	100	37	37	37	37	37	37	37	37	37	6	0	0
	10	24	24	24	24	24	24	24	24	24	24	24	24
P22L	100	35	35	35	35	35	35	35	35	35	29	14	0
	10	29	29	29	29	29	29	29	29	29	29	29	29
P23L	100	25	25	25	25	25	25	25	25	25	25	25	25
	10	20	20	20	20	20	20	20	20	20	20	20	20
P24L	100	29	29	29	29	29	29	29	29	29	29	29	29
	10	26	26	26	26	26	26	26	26	26	26	26	26
P25L	100	21	21	21	21	21	21	21	21	21	21	21	21
	10	24	24	24	24	24	24	24	24	24	24	24	24
P18L	100	19	19	19	19	19	19	19	19	19	19	19	19
	10	27	27	27	27	27	27	27	27	27	27	27	27
PZQ	10	22	0	0	0	0	0	0	0	0	0	0	0
Controle		27	27	27	27	27	27	27	27	27	27	27	27

Molécule	[C] µg/mL	T0	1 H	2 H	3 H	4 H	5 H	6 H	7 H	8 H	24 H	48 H	72 H
A6L	100	25	25	25	25	25	25	25	25	25	13	0	0
	10	23	23	23	23	23	23	23	23	23	12	0	0
A7L	100	29	29	29	29	29	29	29	29	29	12	0	0
	10	24	24	24	24	24	24	24	24	24	24	24	24
P21D	100	42	42	42	42	42	23	23	6	6	0	0	0
	10	32	32	32	32	32	32	32	32	32	32	32	32
P22D	100	36	36	36	36	13	13	5	1	1	0	0	0
	10	29	29	29	29	29	29	29	29	29	22	8	0
P6D	100	26	26	26	26	15	8	8	5	5	0	0	0
	10	11	11	11	11	11	11	11	11	11	11	11	11
P8D	100	18	0	0	0	0	0	0	0	0	0	0	0
	10	25	25	25	25	25	25	25	25	25	0	0	0
P8L	100	26	24	24	24	24	24	17	8	8	0	0	0
	10	24	24	24	24	24	24	24	24	24	24	24	24
P9L	100	25	25	25	7	7	0	0	0	0	0	0	0
	10	26	26	26	26	26	26	26	26	26	26	26	26
P10L	100	28	28	28	28	28	28	4	4	4	0	0	0
	10	19	19	19	19	19	19	19	19	19	19	19	19
TFA	100	25	25	25	25	25	19	19	3	3	0	0	0
	10	20	20	20	20	20	20	20	20	20	20	20	20
Contrôle		39	39	39	39	39	39	39	39	39	39	39	39
		36	36	36	36	36	36	36	36	36	36	36	36
PZQ	100	17	0	0	0	0	0	0	0	0	0	0	0
	10	29	0	0	0	0	0	0	0	0	0	0	0

Analytical data

tert-butyl (S)-(1-oxo-3-phenyl-1-((4-vinylphenyl)amino)propan-2-yl)carbamate **P3L**. ^{1}H NMR (400 MHz, $CDCl_3$) δ 8.57 (s, 1H$_{\{9\}}$), 7.23 (Broad *A*B, d, *J* = 8.6 Hz, 2H$_{\{1, 5\}}$), 7.29 – 7.19 (m, 7H$_{\{2, 4, 15\text{-}19\}}$), 6.53 (dd, *J* = 17.6, 10.9 Hz, 1H$_{\{7\}}$), 5.71 – 5.60 (overlapped signals : d, *J* = 17.5 Hz, 1H$_{\{8\}}$ and s, 1H$_{\{20\}}$), 5.08 (d, *J* = 10.8, 1H$_{\{8\}}$), 4.58 (m, 1H$_{\{12\}}$), 3.08 (dd, *J* = 13.7, 6.4 Hz, 1H$_{\{13\}}$), 2.98 (dd, *J* = 13.8, 8.0 Hz, 1H$_{\{13\}}$), 1.29 (s, 9H$_{\{25\text{-}27\}}$). ^{13}C NMR (101 MHz, $CDCl_3$) δ 170.3 (C$_{\{10\}}$), 156.2 (C$_{\{21\}}$), 137.2 (C$_{\{3\}}$), 136.9 (C$_{\{14\}}$), 136.3 (C$_{\{7\}}$), 133.9 (C$_{\{6\}}$), 129.4 (2CH$_{\{16, 18\}}$), 128.7 (2CH$_{\{1, 5\}}$), 127.0 (CH$_{\{17\}}$), 126.7 (2CH$_{\{15, 19\}}$), 120.1 (2CH$_{\{2\text{-}4\}}$), 113.0 (CH$_{2\{8\}}$), 80.6 (C$_{\{24\}}$), 56.9 (CH$_{\{12\}}$), 38.9 (CH$_{2\{13\}}$), 28.4 (3CH$_{3\{25\text{-}27\}}$). HRMS (ESI) : m/z calc for $C_{22}H_{27}N_2O_3^+$: 367.2016 [M+H]$^+$; found : 367.2015.

Benzyl (tert-butoxycarbonyl)-L-phenylalanyl-L-valinate **P4L.** ^{1}H NMR (300 MHz, $CDCl_3$) δ 7.33 – 7.22 (m, 5H$_{\{1\text{-}5\}}$), 7.21 – 7.07 (m, 5H$_{\{21\text{-}25\}}$), 6.39 (d, J = 8.6 Hz, 1H$_{\{12\}}$), 5.15 – 5.03 (overlapped signals : 3H$_{\{7, 26\}}$), 4.44 (dd, J = 8.6, 4.9 Hz, 1H$_{\{10\}}$), 4.29 (q, J = 7.4 Hz, 1H$_{\{18\}}$), 2.98 (m, 2H$_{\{19\}}$), 2.04 (pd, J = 6.9, 4.9 Hz, 1H$_{\{13\}}$), 1.33 (s, 9H$_{\{31\text{-}33\}}$), 0.77 (d, J = 6.9 Hz, 3H$_{\{14\}}$), 0.72 (d, J = 6.9 Hz, 3H$_{\{15\}}$). ^{13}C NMR (75 MHz, $CDCl_3$) δ 171.3 (C$_{\{9\}}$), 171.3 (C$_{\{16\}}$), 155.5 (C$_{\{27\}}$), 136.7 (C$_{\{20\}}$), 135.4 (C$_{\{6\}}$), 129.4 (2CH$_{\{22, 24\}}$), 128.7 (2CH$_{\{2, 4\}}$), 128.7 (2CH$_{\{1, 5\}}$), 128.5 (CH$_{\{3\}}$), 128.4 (2CH$_{\{21, 25\}}$), 127.0 (CH$_{\{23\}}$), 80.3 (C$_{\{30\}}$), 67.0 (CH$_{2\{7\}}$), 57.3 (CH$_{\{10\}}$), 55.9 (CH$_{\{18\}}$), 38.1 (CH$_{2\{19\}}$), 31.4 (CH$_{\{13\}}$), 28.3 (3CH$_{3\{31\text{-}33\}}$), 18.9 (CH$_{3\{14\}}$), 17.7 (CH$_{3\{14\}}$).

(Tert-butoxycarbonyl)-L-phenylalanyl-L-valine **P5L.** Using GP6 with **P4L** (12.00 g, 26.417 mmol, 1.0 eq) affords **P5L** as a white solid (9.62 g, quantitative yield) as described in literature [1]. ^{1}H NMR (400 MHz, MeOD) δ 7.36 – 7.09 (m, 5H$_{\{9\text{-}13\}}$), 4.52 – 4.16 (overlapped signals : m, 2H$_{\{2, 14\}}$), 3.13 (dd, J = 13.9, 5.0 Hz, 1H$_{\{4\}}$), 2.82 (dd, J = 13.9, 9.5 Hz, 1H$_{\{4\}}$), 2.17 (h, J = 6.7 Hz, 1H$_{\{16\}}$), 1.36 (s, 9H$_{\{24\text{-}26\}}$), 1.05 – 0.93 (overlapped signals : m, 6H$_{\{17, 18\}}$). ^{13}C NMR (75 MHz, MeOD) δ 174.5 (C$_{\{15\}}$), 174.4 (C$_{\{3\}}$), 157.6 (C$_{\{7\}}$), 138.5 (C$_{\{8\}}$), 130.3 (2CH$_{\{10, 12\}}$), 129.3 (2CH$_{\{9, 13\}}$), 127.6 (CH$_{\{11\}}$), 80.7 (C$_{\{23\}}$), 58.8 (CH$_{\{14\}}$), 57.2 (CH$_{\{2\}}$), 38.9 (CH$_{2\{4\}}$), 31.9 (CH$_{\{16\}}$), 28.6 (3CH$_{3\{24\text{-}26\}}$), 19.5 (CH$_{3\{17\}}$), 18.2 (CH$_{3\{18\}}$).

Benzyl (tert-butoxycarbonyl)-L-phenylalanyl-L-valyl-L-phenylalaninate **P6L.** ^{1}H NMR (400 MHz, $CDCl_3$) δ 7.35 (m, 3H$_{\{41\text{-}43\}}$), 7.30 – 7.15 (m, 10H$_{\{9\text{-}13, 34\text{-}38\}}$), 7.02 (m, 2H$_{\{40, 44\}}$), 6.50 (d, J = 8.5 Hz, 1H$_{\{6\}}$), 6.32 (d, J = 8.0 Hz, 1H$_{\{17\}}$), 5.12 (q, J = 12.1 Hz, 2H$_{\{1\}}$), 4.98 (m, 1H$_{\{24\}}$), 4.86 (dt, J = 8.0, 6.1 Hz, 1H$_{\{5\}}$), 4.34 (q, J = 6.5 Hz, 1H$_{\{22\}}$), 4.18 (dd, J = 8.5, 6.1 Hz, 1H$_{\{15\}}$), 3.07 (ddd, J = 13.0, 6.6, 3.4 Hz, 4H$_{\{7, 25\}}$), 2.04 (dq, J = 6.7 Hz, 1H$_{\{18\}}$), 1.40 (s, 9H$_{\{31\text{-}33\}}$), 0.84 (d, J = 6.7 Hz, 3H$_{\{20\}}$), 0.77 (d, J = 6.8 Hz, 3H$_{\{19\}}$). ^{13}C NMR (75 MHz, $CDCl_3$) δ 171.5 (C$_{\{3\}}$), 171.2 (C$_{\{21\}}$), 170.6 (C$_{\{14\}}$), 155.6 (C$_{\{27\}}$), 136.8 (CH$_{\{26\}}$), 135.7 (C$_{\{39\}}$), 135.1 (C$_{\{8\}}$), 129.4 (2CH$_{\{35, 37\}}$), 129.4 (2CH$_{\{10, 12\}}$), 128.7 (4CH$_{\{34, 38, 41, 43\}}$), 128.6 (4CH$_{\{9, 13, 40, 44\}}$), 128.6 (CH$_{\{42\}}$), 127.2 (CH$_{\{36\}}$), 126.9 (CH$_{\{11\}}$), 80.2 (C$_{\{30\}}$), 67.3 (CH$_{2\{1\}}$), 58.5 (CH$_{\{15\}}$), 55.9 (CH$_{\{5\}}$), 53.4 (CH$_{\{22\}}$), 38.1 (2CH$_{2\{7, 25\}}$), 31.1 (CH$_{\{18\}}$), 28.3 (3CH$_{3\{31\text{-}33\}}$), 19.1 (CH$_{3\{20\}}$), 18.1 (CH$_{3\{19\}}$). HRMS (ESI) : m/z calc for $C_{35}H_{43}N_3O_6Na^+$: 624.3044 [M+Na]$^+$; found : 624.3052.

(Tert-butoxycarbonyl)-L-phenylalanyl-L-valyl-L-phenylalanine **P7L.** ^{1}H NMR (300 MHz, $CDCl_3$) δ 9.94 (s, 1H$_{\{1\}}$), 7.61 – 6.92 (m, 12H$_{\{5, 16, 8\text{-}12, 33\text{-}37\}}$), 5.46 (d, J = 7.9 Hz, 1H$_{\{23\}}$), 4.88 (q, J = 6.6 Hz, 1H$_{\{4\}}$), 4.53 (m, 1H$_{\{21\}}$), 4.40 (t, J = 8.1 Hz, 1H$_{\{14\}}$), 3.23 (dd, J = 14.0, 5.6 Hz, 1H$_{\{24\}}$), 3.13 – 2.90 (overlapped signals : m, 3H$_{\{6, 24\}}$), 2.03 (h, J = 6.8 Hz, 1H$_{\{17\}}$), 1.42 (s, 9H$_{\{30\text{-}32\}}$), 0.89 (overlapped signals : 2d, J = 6.6 Hz, 6H$_{\{18, 19\}}$). ^{13}C NMR (75 MHz, $CDCl_3$) δ 173.8 (C$_{\{2\}}$), 172.1 (C$_{\{13\}}$), 171.1 (C$_{\{20\}}$), 155.8 (C$_{\{26\}}$), 136.6 (C$_{\{25\}}$), 136.1 (C$_{\{7\}}$), 129.5 (2CH$_{\{34, 36\}}$), 129.4 (2CH$_{\{9, 11\}}$), 128.6 (4CH$_{\{33, 37, 8, 12\}}$), 127.1 (CH$_{\{35\}}$), 126.9 (CH$_{\{10\}}$), 80.4 (C$_{\{29\}}$), 58.7 (CH$_{\{14\}}$), 55.8 (CH$_{\{4\}}$), 53.6 (CH$_{\{21\}}$), 38.2 (CH$_{2\{6\}}$), 37.7 (CH$_{2\{24\}}$), 31.2 (CH$_{\{17\}}$), 28.3 (3CH$_{3\{30\text{-}32\}}$), 19.1 (CH$_{3\{18\}}$), 18.1 (CH$_{3\{19\}}$). HRMS (ESI) : m/z calc for $C_{28}H_{37}N_3O_6Na^+$: 534.2575 [M+Na]$^+$; found : 534.2580.

(S)-1-(((S)-1-(((S)-1-(benzyloxy)-1-oxo-3-phenylpropan-2-yl)amino)-3-methyl-1-oxobutan-2-yl)amino)-1-oxo-3-phenylpropan-2-aminium trifluoroacetate **P8L**. ^{1}H NMR (400 MHz, MeOD) δ 7.39 – 7.12 (m, 15H$_{\{2\text{-}6, 16\text{-}20, 34\text{-}38\}}$), 5.11 (s, 2H$_{\{8\}}$), 4.72 (dd, J = 8.4, 6.1 Hz, 1H$_{\{12\}}$), 4.23 (d, J = 7.3 Hz, 1H$_{\{22\}}$), 4.12 (dd, J = 8.7, 5.4 Hz, 1H$_{\{29\}}$), 3.20 – 3.07 (Overlapped signals : ddd, J = 19.9, 14.1, 5.7 Hz, 2H$_{\{14, 32\}}$), 3.01 (dd, J = 13.9, 8.4 Hz, 1H$_{\{14\}}$), 2.90 (dd, J = 14.4, 8.6 Hz, 1H$_{\{32\}}$), 2.00 (h, J = 6.8 Hz, 1H$_{\{25\}}$), 0.91 (overlapped signals : 2d, J = 6.8 Hz, 6H$_{\{26, 27\}}$). ^{13}C NMR (101 MHz, MeOD-$CDCl_3$ (1 : 1 ; v : v)) δ 172.0 (C$_{\{10\}}$), 171.9 (C$_{\{21\}}$), 169.0 (C$_{\{28\}}$), 136.9 (C$_{\{32\}}$), 135.8 (C$_{\{7\}}$), 134.6 (C$_{\{15\}}$), 129.9 (2CH$_{\{35, 37\}}$), 129.8 (2CH$_{\{17, 19\}}$), 129.6 (2CH$_{\{3, 5\}}$), 129.1 (2CH$_{\{2, 6\}}$), 129.1 (2CH$_{\{34, 38\}}$), 129.0 (2CH$_{\{16, 20\}}$), 129.0 (CH$_{\{4\}}$), 128.3 (CH$_{\{35\}}$), 127.5 (CH$_{\{18\}}$), 67.8 (CH$_{2\{8\}}$), 59.7 (CH$_{\{22\}}$), 54.8 (CH$_{\{29\}}$), 54.5 (CH$_{\{12\}}$), 38.1 (2CH$_{2\{14, 32\}}$), 31.5 (CH$_{\{25\}}$) , 19.3 (CH$_{3\{26\}}$), 18.4 (CH$_{3\{27\}}$). ^{19}F NMR (376 MHz, MeOD) δ -76.30. HRMS (ESI) : m/z calc for cation $C_{30}H_{36}N_3O_4^+$: 502.2700 ; found : 502.2701. m/z calc for anion $C_2F_3O_2^-$: 112.9856 ; found : 112.9860.

Benzyl 4-(((S)-1-(((S)-1-(((S)-1-(benzyloxy)-1-oxo-3-phenylpropan-2-yl)amino)-3-methyl-1-oxobutan-2-yl)amino)-1-oxo-3-phenylpropan-2-yl)amino)-4-oxobutanoate **P9L**. ^{1}H NMR (400 MHz, $CDCl_3$) δ 7.41 – 7.30 (m, 8H$_{\{47\text{-}51, 34\text{-}36\}}$), 7.28 – 7.15 (m, 10H$_{\{8\text{-}12, 26\text{-}30\}}$), 7.08 – 7.00 (m, 2H$_{\{33, 37\}}$), 6.52 (d, J = 8.4 Hz, 1H$_{\{16\}}$), 6.42 (d, J = 7.9 Hz, 1H$_{\{5\}}$), 6.26 (d, J = 7.5 Hz, 1H$_{\{23\}}$), 5.19 – 5.05 (overlapped signals : m, 4H$_{\{31, 45\}}$), 4.86 (dt, J = 7.9, 6.2 Hz, 1H$_{\{4\}}$), 4.67 (q, J = 6.9 Hz, 1H$_{\{21\}}$), 4.18 (dd, J = 8.5, 6.3 Hz, 1H$_{\{14\}}$), 3.14 – 3.00 (overlapped signals : m, 4H$_{\{6, 24\}}$), 2.76 – 2.55 (m, 2H$_{\{41\}}$), 2.50 – 2.38 (m, 2H$_{\{39\}}$), 2.07 (h, J = 6.7 Hz, 1H$_{\{17\}}$), 0.83 (d, J = 6.8 Hz, 3H$_{\{18\}}$), 0.76 (d, J = 6.8 Hz, 3H$_{\{19\}}$). ^{13}C NMR (101 MHz, $CDCl_3$) δ 172.9 (C$_{\{38\}}$), 171.6 (C$_{\{2\}}$), 171.3

(C$_{\{42\}}$), 171.2 (C$_{\{13\}}$), 170.8 (C$_{\{20\}}$), 136.6 (C$_{\{25\}}$), 136.0 (C$_{\{46\}}$), 135.9 (C$_{\{32\}}$), 135.2 (C$_{\{7\}}$), 129.4 (4CH$_{\{27, 29, 9, 11\}}$), 128.6 (8CH$_{\{26, 30, 8, 12, 34, 36, 48, 50\}}$), 128.5 (2CH$_{\{33, 37\}}$), 128.5 (CH$_{\{35\}}$), 128.3 (CH$_{\{49\}}$), 128.2 (2CH$_{\{47, 51\}}$), 127.1 (CH$_{\{28\}}$), 126.9 (CH$_{\{10\}}$), 67.2 (CH$_{2\{31\}}$), 66.6 (CH$_{2\{45\}}$), 58.5 (CH$_{\{14\}}$), 54.3 (CH$_{\{21\}}$), 53.5 (CH$_{\{4\}}$), 38.6 (CH$_{2\{24\}}$), 38.2 (CH$_{2\{6\}}$), 31.2 (CH$_{\{17\}}$), 30.8 (CH$_{2\{41\}}$), 29.6 (CH$_{2\{39\}}$), 19.1 (CH$_{3\{18\}}$), 18.4(CH$_{3\{19\}}$). HRMS (ESI) : m/z calc for $C_{41}H_{45}N_3O_7Na^+$: 714.3150 [M+Na]$^+$; found : 714.3135.

4-(((S)-1-(((S)-1-(((S)-1-carboxy-2-phenylethyl)amino)-3-methyl-1-oxobutan-2-yl)amino)-1-oxo-3-phenylpropan-2-yl)amino)-4-oxobutanoic acid **P10L**. ^{1}H NMR (400 MHz, MeOD) δ 7.31 – 7.04 (m, 10H$_{\{8-12, 26-30\}}$), 4.71 – 4.51 (overlapped signals : m, 2H$_{\{4, 21\}}$), 4.18 (d, J = 7.5 Hz, 1H$_{\{14\}}$), 3.18 (dd, J = 14.0, 5.3 Hz, 1H$_{\{4\}}$), 3.08 (dd, J = 14.1, 5.0 Hz, 1H$_{\{24\}}$), 2.99 (dd, J = 13.9, 8.4 Hz, 1H$_{\{4\}}$), 2.83 (dd, J = 14.1, 9.5 Hz, 1H$_{\{24\}}$), 2.60 – 2.34 (m, 4H$_{\{32, 34\}}$), 2.03 (h, J = 6.9 Hz, 1H$_{\{17\}}$), 0.90 (overlapped signals : t, J = 6.9 Hz, 6H$_{\{18, 19\}}$). ^{13}C NMR (101 MHz, MeOD) δ 176.1 (C$_{\{35\}}$), 174.4 (2C$_{\{2, 31\}}$), 173.5 (C$_{\{13\}}$), 173.1 (C$_{\{20\}}$), 138.5 (C$_{\{25\}}$), 138.3 (C$_{\{7\}}$), 130.3 (2CH$_{\{27, 29\}}$), 130.2 (2CH$_{\{9, 11\}}$), 129.5 (2CH$_{\{26, 30\}}$), 129.4 (2CH$_{\{8, 12\}}$), 127.8 (CH$_{\{28\}}$), 127.6 (CH$_{\{10\}}$), 60.1 (CH$_{\{14\}}$), 55.9 (CH$_{\{4\}}$), 55.1 (CH$_{\{21\}}$), 38.6 (CH$_{2\{6\}}$), 38.5 (CH$_{2\{24\}}$), 32.1 (CH$_{\{17\}}$), 31.5 (CH$_{2\{34\}}$), 30.2 (CH$_{2\{32\}}$), 19.7 (CH$_{3\{18\}}$), 18.7 (CH$_{3\{19\}}$). HRMS (ESI) : m/z calc for $C_{27}H_{32}N_3O_7^-$: 510.2246 [M-H]$^-$; found : 510.2231.

(S)-1-(((S)-1-(((S)-1-carboxy-2-phenylethyl)amino)-3-methyl-1-oxobutan-2-yl)amino)-1-oxo-3-phenylpropan-2-aminium trifluoroacetate **P11L**. ^{1}H NMR (400 MHz, MeOD) δ 7.34 – 7.10 (m, 10H$_{\{8-12, 26-30\}}$), 4.63 (dd, J = 8.2, 5.1 Hz, 1H$_{\{4\}}$), 4.22 (d, J = 7.3 Hz, 1H$_{\{14\}}$), 4.12 (dd, J = 8.9, 5.1 Hz, 1H$_{\{21\}}$), 3.21 (dd, J = 13.9, 5.0 Hz, 1H$_{\{6\}}$), 3.15 (dd, J = 14.3, 5.1 Hz, 1H$_{\{24\}}$), 3.01 (dd, J = 13.9, 8.2 Hz, 1H$_{\{6\}}$), 2.90 (dd, J = 14.4, 9.0 Hz, 1H$_{\{24\}}$), 2.06 (h, J = 6.8 Hz, 1H$_{\{17\}}$), 0.95 (overlapped signals : t, J = 6.6 Hz, 6H$_{\{18, 19\}}$). ^{13}C NMR (101 MHz, DMSO-d_6) δ 172.7 (C$_{\{2\}}$), 170.3 (C$_{\{13\}}$), 167.9 (C$_{\{20\}}$), 137.6 (C$_{\{7\}}$), 134.9 (C$_{\{25\}}$), 129.4 (2CH$_{\{9, 11\}}$), 129.0 (2CH$_{\{27, 29\}}$), 128.4 (2CH$_{\{8, 12\}}$), 128.1 (2CH$_{\{26, 30\}}$), 127.0 (CH$_{\{10\}}$), 126.3 (CH$_{\{28\}}$), 57.5 (CH$_{\{14\}}$), 53.4 (CH$_{\{4\}}$), 53.2 (CH$_{\{21\}}$), 37.2 (CH$_{2\{6\}}$), 36.6 (CH$_{2\{24\}}$), 31.0 (CH$_{\{17\}}$), 19.0 (CH$_{3\{18\}}$), 18.1 (CH$_{3\{19\}}$). ^{19}F NMR (376 MHz, MeOD) δ -76.94. HRMS (ESI) : m/z calc for cation $C_{31}H_{39}N_4O_3^+$: 412.2231 ; found : 412.2231. m/z calc for anion $C_2F_3O_2^-$: 112.9856 ; found : 112.9856.

(4-(benzyloxy)-4-oxobutanoyl)-L-phenylalanyl-L-valyl-L-phenylalanine **P12L.** ^{1}H NMR (400 MHz, MeOD) δ 7.41 – 7.27 (m, 5H$_{\{40-44\}}$), 7.25 – 7.07 (m, 10H$_{\{8-12, 26-30\}}$), 5.12 – 4.93 (m, 2H$_{\{38\}}$), 4.65 (dd, J = 9.8, 4.7 Hz, 1H$_{\{21\}}$), 4.52 (dd, J = 7.9, 5.1 Hz, 1H$_{\{4\}}$), 4.13 (d, J = 7.5 Hz, 1H$_{\{14\}}$), 3.20 (dd, J = 14.0, 5.1 Hz, 1H$_{\{6\}}$), 3.13 (dd, J = 14.2, 4.8 Hz, 1H$_{\{24\}}$), 2.98 (dd, J = 13.9, 7.9 Hz, 1H$_{\{6\}}$), 2.85 (dd, J = 14.2, 9.8 Hz, 1H$_{\{24\}}$), 2.72 – 2.29 (m, 4H$_{\{32, 34\}}$), 2.05 (h, J = 6.8 Hz, 1H$_{\{17\}}$), 0.88 (d, J = 6.8 Hz, 3H$_{\{18\}}$), 0.86 (d, J = 6.8 Hz, 3H$_{\{19\}}$). ^{13}C NMR (101 MHz, MeOD-CDCl$_3$ (1 : 1 ; v : v)) δ 177.8 (C$_{\{2\}}$), 173.9 (C$_{\{31\}}$), 173.7 (C$_{\{35\}}$), 173.4 (C$_{\{13\}}$), 172.2 (C$_{\{20\}}$), 138.3 (C$_{\{7\}}$), 137.2 (C$_{\{25\}}$), 136.1 (C$_{\{39\}}$), 129.6 (2CH$_{\{27, 29\}}$), 129.5 (2CH$_{\{9, 11\}}$), 129.0 (4CH$_{\{40, 41, 43, 44\}}$), 128.9 (2CH$_{\{8, 12\}}$), 128.8 (2CH$_{\{40, 44\}}$), 128.5 (CH$_{\{42\}}$), 127.3 (CH$_{\{28\}}$), 126.9 (CH$_{\{10\}}$), 67.1 (CH$_{2\{38\}}$), 60.6 (CH$_{\{14\}}$), 55.8 (CH$_{\{4\}}$), 55.6 (CH$_{\{21\}}$), 37.9 (CH$_{2\{6\}}$), 37.6 (CH$_{2\{\}}$), 30.9 (CH$_{2\{34\}}$), 30.5 (CH$_{\{17\}}$), 29.9 (CH$_{2\{32\}}$), 19.3 (CH$_{3\{18\}}$), 18.5 (CH$_{3\{19\}}$). HRMS (ESI) : m/z calc for $C_{34}H_{39}N_3O_7Na^+$: 624.2680 [M+Na]$^+$; found : 624.2681.

Benzyl (((9H-fluoren-9-yl)methoxy)carbonyl)-L-phenylalanyl-L-valinate **P14L.** ^{1}H NMR (400 MHz, MeOD) δ 7.77 (d, J = 7.6 Hz, 2H$_{\{32, 33\}}$), 7.56 (broad d, J = 6,9 Hz, 2H$_{\{29, 36\}}$), 7.42 – 7.12 (m, 14H$_{\{30, 31, 34, 35, 9-13, 39-43\}}$), 5.20 – 5.09 (m, 2H$_{\{37\}}$), 4.47 (dd, J = 9.5, 5.2 Hz, 1H$_{\{2\}}$), 4.37 (d, J = 6.1 Hz 1H$_{\{14\}}$), 4.28 (dd, J = 10.3, 7.2 Hz, 1H$_{\{23\}}$), 4.20 (dd, J = 10.3, 7.2 Hz, 1H$_{\{23\}}$), 4.12 (t, J = 7.1 Hz, 1H$_{\{24\}}$), 3.07 (dd, J = 13.9, 5.2 Hz, 1H$_{\{4\}}$), 2.82 (dd, J = 13.8, 9.6 Hz, 1H$_{\{4\}}$), 2.13 (h, J = 6.7 Hz, 1H$_{\{16\}}$), 0.90 (d, J = 6.8 Hz, 6H$_{\{17, 19\}}$). ^{13}C NMR (75 MHz, CDCl$_3$) δ 171.2 (C$_{\{15\}}$), 171.1 (C$_{\{3\}}$), 156.1 (C$_{\{7\}}$), 143.9 (C$_{\{25\}}$), 143.8 (C$_{\{28\}}$), 141.4 (2C$_{\{26, 27\}}$), 136.4 (C$_{\{8\}}$), 135.3 (C$_{\{38\}}$), 129.4 (2CH$_{\{10, 12\}}$), 128.8 (2CH$_{\{9, 13\}}$), 128.7 (2CH$_{\{40, 42\}}$), 128.5 (CH$_{\{41\}}$), 128.4 (2CH$_{\{39, 43\}}$), 127.8 (2CH$_{\{31, 34\}}$), 127.2 (2CH$_{\{32, 33\}}$), 127.1 (CH$_{\{11\}}$), 125.2 (2CH$_{\{30, 35\}}$), 120.0 (2CH$_{\{29, 36\}}$), 67.2 (CH$_{2\{37\}}$), 67.1 (CH$_{2\{23\}}$), 57.4 (CH$_{\{14\}}$), 56.2 (CH$_{\{2\}}$), 47.1 (CH$_{\{24\}}$), 38.6 (CH$_{2\{4\}}$), 31.3(CH$_{\{16\}}$) , 18.9 (CH$_{3\{17\}}$), 17.7 (CH$_{3\{18\}}$). HRMS (ESI) : m/z calc for $C_{36}H_{36}N_2O_5Na^+$: 599.2516 [M+Na]$^+$; found : 599.2518.

(((9H-fluoren-9-yl)methoxy)carbonyl)-L-phenylalanyl-L-valine **P15L.** ^{1}H NMR (400 MHz, MeOD) δ 7.77 (d, *J* = 7.5 Hz, 2H$_{\{32, 33\}}$), 7.56 (dd, *J* = 7.6, 4.3 Hz, 2H$_{\{29, 36\}}$), 7.41 – 7.15 (m, 7H$_{\{30, 31, 34, 35, 9-13\}}$), 4.48 (dd, *J* = 9.7, 5.0 Hz, 1H$_{\{2\}}$), 4.34 (d, *J* = 5.6 Hz, 1H$_{\{14\}}$), 4.28 (dd, *J* = 10.4, 7.2 Hz, 1H$_{\{23\}}$), 4.21 (dd, *J* = 10.5, 7.0 Hz, 1H$_{\{23\}}$), 4.13 (t, *J* = 7.0 Hz, 1H$_{\{24\}}$), 3.15 (dd, *J* = 14.0, 5.0 Hz, 1H$_{\{4\}}$), 2.86 (dd, *J* = 13.9, 9.7 Hz, 1H$_{\{4\}}$), 2.17 (dq, *J* = 13.8, 6.7 Hz, 1H$_{\{16\}}$), 0.96 (overlapped signals : 2d, *J* = 6.9 Hz, 6H$_{\{17, 18\}}$). ^{13}C NMR (101 MHz, MeOD) δ 174.5 (C$_{\{15\}}$), 174.2 (C$_{\{3\}}$), 158.2 (C$_{\{7\}}$), 145.2 (C$_{\{25\}}$), 145.1 (C$_{\{28\}}$), 142.5 (2C$_{\{26, 27\}}$), 138.5 (C$_{\{8\}}$), 130.4 (2CH$_{\{10, 12\}}$), 129.4 (2CH$_{\{9, 13\}}$), 128.7 (2CH$_{\{31, 34\}}$), 128.1 (2CH$_{\{32, 33\}}$), 127.7 (CH$_{\{11\}}$), 126.2 (CH$_{\{30\}}$), 126.2 (2CH$_{\{35\}}$), 120.8 (2CH$_{\{29, 36\}}$), 68.0 (CH$_{2\{23\}}$), 59.0 (CH$_{\{14\}}$), 57.6 (CH$_{\{2\}}$), 48.3 (CH$_{\{24\}}$), 39.0 (CH$_{2\{4\}}$), 31.9 (CH$_{\{16\}}$), 19.5 (CH$_{3\{17\}}$), 18.3 (CH$_{3\{18\}}$).

Benzyl (((9H-fluoren-9-yl)methoxy)carbonyl)-L-phenylalanyl-L-valyl-L-phenylalaninate **P16L.** ^{1}H NMR (400 MHz, CDCl$_3$) δ 7.79 – 7.70 (m, 2H$_{\{43, 44\}}$), 7.58 – 7.48 (m, 2H$_{\{40, 47\}}$), 7.44 – 7.11 (m, 17H$_{\{9-13, 25-29, 41, 42, 45, 46, 51-53\}}$), 7.05 – 6.95 (m, 2H$_{\{50, 54\}}$), 6.38 (m, 1H$_{\{5\}}$), 6.25 – 6.13 (m, 1H$_{\{19\}}$), 5.30 (m, 1H$_{\{1\}}$), 5.19 – 5.03 (m, 2H$_{\{48\}}$), 4.94 – 4.81 (m, H$_{\{21\}}$), 4.43 (overlapped signals : dd, J = 10.6, 7.0 Hz, 2H$_{\{14, 35\}}$), 4.32 (m, 1H$_{\{2\}}$), 4.18 (m, 2H$_{\{34\}}$), 3.06 (m, 4H$_{\{4, 23\}}$) 2.01 (m, 1H$_{\{16\}}$), 0.82 (d, J = 6.8

Hz, 3H$_{\{17\}}$), 0.76 (d, J = 6.8 Hz, 3H$_{\{18\}}$). ^{13}C NMR (101 MHz, $CDCl_3$) δ 171.2 (C$_{\{21\}}$), 171.1 (C$_{\{15\}}$), 170.3 (C$_{\{3\}}$), 156.1 (C$_{\{7\}}$), 143.9 (C$_{\{36\}}$), 143.8 (C$_{\{39\}}$), 141.4 (2C$_{\{37, 38\}}$), 136.4 (C$_{\{8\}}$), 135.7 (C$_{\{(C\{24\})\}}$), 135.1 (C$_{\{49\}}$), 129.4 (2CH$_{\{10, 12\}}$), 129.4 (2CH$_{\{26, 28\}}$), 128.9 (2CH$_{\{9, 13\}}$), 128.8 (2CH$_{\{25, 29\}}$), 128.7 (4CH$_{\{50, 51, 53, 54\}}$), 128.6 (CH$_{\{52\}}$), 127.9 (2CH$_{\{42, 45\}}$), 127.3 (CH$_{\{11\}}$), 127.2 (CH$_{\{27\}}$), 127.2 (2CH$_{\{43, 44\}}$), 125.2 (CH$_{\{41\}}$), 125.2 (CH$_{\{46\}}$), 120.1 (2CH$_{\{40, 47\}}$), 67.4 (CH$_{2\{48\}}$), 67.3 (CH$_{2\{34\}}$), 58.6 (CH$_{\{14\}}$), 56.3 (CH$_{\{2\}}$), 53.4 (CH$_{\{21\}}$), 47.3 (CH$_{\{35\}}$), 38.4 (CH$_{2\{4\}}$), 38.1 (CH$_{2\{23\}}$), 31.2 (CH$_{\{16\}}$), 19.1 (CH$_{3\{17\}}$), 18.1 (CH$_{3\{18\}}$). HRMS (ESI) : m/z calc for $C_{45}H_{45}N_3O_6Na^+$: 746.3201 [M+Na]$^+$; found : 746.3205.

L-phenylalanyl-L-valyl-L-phenylalanine **P18L.** ^{1}H NMR (400 MHz, D_2O-DMSO-*d*$_6$, 5% DCl) δ 6.96 – 6.71 (m, 8H$_{\{8, 9, 11, 12, 26, 27, 29, 30\}}$), 6.68 – 6.50 (m, 2H$_{\{10, 28\}}$), 4.11 (dd, J = 9.0, 5.3 Hz, 1H$_{\{4\}}$), 3.83 (t, J = 7.1 Hz, 1H$_{\{21\}}$), 3.64 (d, J = 8.0 Hz, 1H$_{\{14\}}$), 2.73 (dd, J = 14.0, 5.3 Hz, 1H$_{\{6\}}$), 2.67 – 2.33 (m, 3H$_{\{6, 24\}}$), 1.46 (h, J = 6.8 Hz, 1H$_{\{17\}}$), 0.40 (overlapped signals : dd, J = 6.8 Hz, 6H$_{\{18, 19\}}$). ^{13}C NMR (101 MHz, D_2O-DMSO-*d*$_6$, 5% DCl) δ 175.9 (C$_{\{4\}}$), 173.6 (C$_{\{21\}}$), 170.1 (C$_{\{13\}}$), 138.1 (C$_{\{7\}}$), 135.1 (C$_{\{25\}}$), 131.0 (2CH$_{\{9, 11\}}$), 130.9 (2CH$_{\{27, 29\}}$), 130.7 (2CH$_{\{8, 12\}}$), 130.4 (2CH$_{\{26, 30\}}$), 129.5 (CH$_{\{10\}}$), 128.8 (CH$_{\{28\}}$), 60.8 (CH$_{\{4\}}$), 55.7 (CH$_{\{21\}}$), 38.5 (CH$_{2\{6\}}$), 38.1 (CH$_{2\{24\}}$), 32.0 (CH$_{\{17\}}$), 19.9 (CH$_{3\{18\}}$), 19.4 (CH$_{3\{19\}}$). HRMS (ESI) : m/z calc for $C_{23}H_{29}N_3O_4Na^+$: 434.2050 [M+Na]$^+$; found : 434.2050.

tert-butyl (S)-(1-oxo-3-phenyl-1-((4-vinylphenyl)amino)propan-2-yl)carbamate **P19L**. ^{1}H NMR (400 MHz, MeOD) δ 7.36 (Broad A*B*, d, *J* = 8.0 Hz, 2H$_{\{2, 4\}}$), 7.29 – 7.15 (m, 5H$_{\{22-26\}}$), 7.10 (Broad *A*B, d, *J* = 8.0 Hz, 2H$_{\{1, 5\}}$), 4.50 – 4.24 (m, 1H$_{\{11\}}$), 3.12 (dd, *J* = 13.7, 6.4 Hz, 1H$_{\{12\}}$), 2.93 (dd, *J* = 13.7, 8.3 Hz, 1H$_{\{12\}}$), 2.58 (q, *J* = 7.6 Hz, 2H$_{\{7\}}$), 1.38 (s, 9H$_{\{19-21\}}$), 1.19 (t, *J* = 7.6 Hz, 3H$_{\{27\}}$). ^{13}C NMR (101 MHz, MeOD) δ 172.4 (C$_{\{9\}}$), 157.5 (C$_{\{15\}}$), 141.7 (C$_{\{6\}}$), 138.3 (C$_{\{13\}}$), 136.8 (C$_{\{3\}}$), 130.4 (2CH$_{\{23, 25\}}$), 129.4 (2CH$_{\{1, 5\}}$), 129.0 (2CH$_{\{22, 26\}}$), 127.7 (CH$_{\{24\}}$), 121.7 (2CH$_{\{2, 4\}}$), 80.6 (C$_{\{18\}}$), 57.9 (CH$_{\{11\}}$), 39.7 (CH$_{2\{12\}}$), 29.2 (CH$_{2\{7\}}$), 28.6 (3CH$_{3\{19-21\}}$), 16.1 (CH$_{3\{27\}}$). HRMS (ESI) : m/z calc for $C_{22}H_{28}N_2O_3Na^+$: 391.1992 [M+Na]$^+$; found : 391.1990.

(S)-1-((4-ethylphenyl)amino)-1-oxo-3-phenylpropan-2-aminium trifluoroacetate **P20L:** ^{1}H NMR (400 MHz, MeOD) δ 7.39 – 7.28 (m, 7H$_{\{2, 4, 14-18\}}$), 7.16 – 7.13 (m, 2H$_{\{1, 5\}}$), 4.17 (t, J = 7.4 Hz, 1H$_{\{11\}}$), 3.27 (dd, J = 13.8, 7.2 Hz, 1H$_{\{12\}}$), 3.16 (dd, J = 13.8, 7.5 Hz, 1H$_{\{12\}}$), 2.61 (q, J = 7.6 Hz, 2H$_{\{7\}}$), 1.21 (t, J = 7.6 Hz, 3H$_{\{20\}}$). ^{13}C NMR (101 MHz, MeOD) δ 167.7 (C$_{\{9\}}$), 163,0 (q, J_{C-F} = 34.6 Hz, CF_3), 142.3 (C$_{\{6\}}$), 136.3 (C$_{\{13\}}$), 135.5 (C$_{\{3\}}$), 130.5 (2CH$_{\{15, 17\}}$), 130.0 (2CH$_{\{1, 5\}}$), 129.1 (2CH$_{\{14, 18\}}$), 128.8 (CH$_{\{16\}}$), 121.6 (2CH$_{\{2,4\}}$), 56.3 (CH$_{\{11\}}$), 38.7 (CH$_{2\{12\}}$), 29.2 (CH$_{2\{7\}}$), 16.1 (CH$_{3\{20\}}$). ^{19}F NMR (376 MHz, MeOD) δ -76.75. HRMS (ESI) : m/z calc for cation $C_{17}H_{21}N_2O^+$: 269.1648 ; found : 269.1649. m/z calc for anion $C_2F_3O_2^-$: 112.9856 ; found : 112.9856.

tert-butyl ((S)-1-(((S)-1-(((S)-1-((4-ethylphenyl)amino)-1-oxo-3-phenylpropan-2-yl)amino)-3-methyl-1-oxobutan-2-yl)amino)-1-oxo-3-phenylpropan-2-yl)carbamate **P21L**. ^{1}H NMR (400 MHz, $CDCl_3$) δ 8.41 (s, 1H$_{\{9\}}$), 7.42 (broad, A*B*, d, J = 8.0 Hz, 2H$_{\{2, 4\}}$), 7.20 – 7.05 (m, 10H$_{\{16-20, 41-45\}}$), 7.02 (*A*B, d, J = 8.1 Hz, 2H$_{\{1, 5\}}$), 6.94 (s, 1H$_{\{13\}}$), 6.87 (d, J = 8.3 Hz, 1H$_{\{24\}}$), 5.08 (d, J = 6.5 Hz, 1H$_{\{31\}}$), 4.84 (td, J = 8.6, 8.6, 6.0 Hz, 1H$_{\{12\}}$), 4.42 (q, J = 6.7 Hz, 1H$_{\{29\}}$), 4.21 (dd, J = 7.0, 5.2 Hz, 1H$_{\{22\}}$), 3.33 (dd, J = 14.5, 5.7 Hz, 1H$_{\{32\}}$), 3.00 (dd, J = 13.9, 5.7 Hz, 1H$_{\{14\}}$), 2.95 – 2.80 (overlapped signals, 2dd, 2H$_{\{14, 32\}}$), 2.52 (q, J = 7.6 Hz, 2H$_{\{7\}}$), 2.02 (dq, J = 13.7, 6.7 Hz, 1H$_{\{25\}}$), 1.29 (s, 9H$_{\{38-40\}}$), 1.13 (t, J = 7.6 Hz, 3H$_{\{46\}}$), 0.69 (d, J = 6.8 Hz, 3H$_{\{27\}}$), 0.54 (d, J = 6.8 Hz, 3H$_{\{26\}}$). ^{13}C NMR (101 MHz, $CDCl_3$) δ 172.4 (C$_{\{10\}}$), 172.0 (C$_{\{21\}}$), 169.8 (C$_{\{28\}}$), 155.9 (C$_{\{34\}}$), 140.4 (C$_{\{6\}}$), 137.1 (C$_{\{33\}}$), 136.6 (C$_{\{15\}}$), 135.4 (C$_{\{3\}}$), 129.6 (2CH$_{\{42, 44\}}$), 129.4 (2CH$_{\{17, 19\}}$), 128.6 (2CH$_{\{41, 45\}}$), 128.4 (2CH$_{\{16, 20\}}$), 128.1 (2CH$_{\{1, 5\}}$), 126.8 (CH$_{\{43\}}$), 126.7 (CH$_{\{18\}}$), 121.1 (2CH$_{\{2, 4\}}$), 79.7 (C$_{\{37\}}$), 58.4 (CH$_{\{22\}}$), 55.7 (CH$_{\{12\}}$), 55.4 (CH$_{\{29\}}$), 39.4 (CH$_{2\{14\}}$), 38.6 (CH$_{2\{32\}}$), 31.8 (CH$_{\{25\}}$), 28.5 (3CH$_{3\{38-40\}}$) , 28.4 (CH$_{2\{7\}}$), 19.3 (CH$_{3\{27\}}$), 18.3 (CH$_{3\{26\}}$), 15.7 (CH$_{3\{46\}}$). HRMS (ESI) : m/z calc for $C_{36}H_{46}N_4O_5Na^+$: 637.3360 [M+Na]$^+$; found : 637.3360.

(S)-1-(((S)-1-(((S)-1-((4-ethylphenyl)amino)-1-oxo-3-phenylpropan-2-yl)amino)-3-methyl-1-oxobutan-2-yl)amino)-1-oxo-3-phenylpropan-2-aminium trifluoroacetate **P22L**. ^{1}H NMR (400 MHz, MeOD) δ 7.39 – 7.17 (m, 12H$_{\{16-20, 34-38, 2, 4\}}$), 7.11 (Broad A*B*, d, J = 8.5 Hz, 2H$_{\{1, 5\}}$), 4.73 (dd, J = 7.8, 7.0 Hz, 1H$_{\{12\}}$), 4.24 (d, J = 7.5 Hz, 1H$_{\{22\}}$), 4.12 (dd, J = 8.6, 5.4 Hz, 1H$_{\{29\}}$), 3.21 – 3.13 (overlapped signals : ddd, J = 13.7, 6.2, 3.4 Hz, 2H$_{\{32, 14\}}$), 3.03 (dd, J = 13.7, 7.8 Hz, 1H$_{\{14\}}$), 2.93 (dd, J = 14.3, 8.6 Hz, 1H$_{\{32\}}$), 2.59 (q, J = 7.6 Hz, 2H$_{\{7\}}$), 2.05 (dq, J = 13.8, 6.9 Hz, 1H$_{\{25\}}$), 1.20 (t, J = 7.6 Hz, 3H$_{\{8\}}$), 0.94 (Overlapped signals : 2d, J = 6.8 Hz, 6H$_{\{26, 27\}}$). ^{13}C NMR (101 MHz, MeOD-$CDCl_3$ (1 : 1 ; v : v)) δ 172.0 (C$_{\{10\}}$), 170.4 (C$_{\{21\}}$), 169.8 (C$_{\{28\}}$), 141.4 (C$_{\{6\}}$), 137.1 (C$_{\{33\}}$), 135.6 (C$_{\{3\}}$), 134.8 (C$_{\{15\}}$), 129.9 (2CH$_{\{35, 37\}}$), 129.8 (2CH$_{\{17, 19\}}$), 129.5 (2CH$_{\{1, 5\}}$), 129.0 (2CH$_{\{34, 38\}}$), 128.6 (2CH$_{\{16, 20\}}$), 128.2 (CH$_{\{36\}}$), 127.4 (CH$_{\{18\}}$), 121.2 (2CH$_{\{2, 4\}}$), 60.0 (CH$_{\{22\}}$), 55.8 (CH$_{\{29\}}$), 54.9 (CH$_{\{12\}}$), 39.0 (CH$_{2\{32\}}$), 38.3 (CH$_{2\{14\}}$), 31.5 (CH$_{\{25\}}$), 28.8 (CH$_{\{7\}}$), 19.2 (CH$_{3\{26\}}$), 18.5 (CH$_{3\{27\}}$), 15.8 (CH$_{3\{8\}}$). ^{19}F NMR (376 MHz, MeOD) δ -76.99. HRMS (ESI) : m/z calc for cation $C_{31}H_{39}N_4O_3^+$: 515.3017 ; found : 515.3015. m/z calc for anion $C_2F_3O_2^-$: 112.9856 ; found : 112.9858.

Benzyl 4-(((S)-1-(((S)-1-(((S)-1-((4-ethylphenyl)amino)-1-oxo-3-phenylpropan-2-yl)amino)-3-methyl-1-oxobutan-2-yl)amino)-1-oxo-3-phenylpropan-2-yl)amino)-4-oxobutanoate **P23L**. ^{1}H NMR (400 MHz, MeOD) δ 7.43 – 7.35 (Broad *A*B, J = 8.5 Hz, 2H$_{\{2, 4\}}$), 7.35 – 7.27 (m, 5H$_{\{48-52\}}$), 7.27 – 7.14 (m, 10H$_{\{15-19, 33-37\}}$), 7.10 (Broad A*B*, J = 8.5 Hz, 2H$_{\{1, 5\}}$), 5.12 – 5.00 (m, 2H$_{\{46\}}$),

4.70 (dd, J = 8.3, 6.7 Hz, 1H$_{\{11\}}$), 4.63 (dd, J = 9.2, 5.2 Hz, 1H$_{\{28\}}$), 4.09 (d, J = 7.2 Hz, 1H$_{\{21\}}$), 3.21 (dd, J = 13.8, 6.7 Hz, 1H$_{\{13\}}$), 3.12 (dd, J = 14.2, 5.3 Hz, 1H$_{\{46\}}$), 2.97 (dd, J = 13.8, 8.3 Hz, 1H$_{\{13\}}$), 2.87 (dd, J = 14.1, 9.2 Hz, 1H$_{\{31\}}$), 2.70 – 2.36 (overlapped signals : m, 6H$_{\{7, 40, 42\}}$), 1.99 (h, J = 6.9 Hz, 1H$_{\{24\}}$), 1.19 (t, J = 7.6 Hz, 3H$_{\{38\}}$), 0.83 (d, J = 6.8 Hz, 3H$_{\{25\}}$), 0.81 (d, J = 6.8 Hz, 3H$_{\{26\}}$). ^{13}C NMR (101 MHz, CDCl$_3$) δ 172.8 (C$_{\{39\}}$), 171.8 (C$_{\{9\}}$), 171.7 (C$_{\{43\}}$), 171.6 (C$_{\{20\}}$), 170.0 (C$_{\{27\}}$), 140.4 (C$_{\{6\}}$), 137.1 (C$_{\{32\}}$), 136.7 (C$_{\{14\}}$), 136.1 (C$_{\{47\}}$), 135.5 (C$_{\{3\}}$), 129.8 (2CH$_{\{34, 36\}}$), 129.6 (2CH$_{\{16, 18\}}$), 128.8 – 128.0 (overlapped signals : 11CH$_{\{48-52, 33, 35, 37, 15, 17, 19\}}$), 126.7 (2CH$_{\{1, 5\}}$), 121.3 (2CH$_{\{2, 4\}}$), 66.3 (CH$_{2\{46\}}$), 58.4 (CH$_{\{21\}}$), 54.8 (CH$_{\{11\}}$), 53.9 (CH$_{\{28\}}$), 40.3 (CH$_{2\{13\}}$), 39.3 (CH$_{2\{31\}}$), 32.3 (CH$_{2\{24\}}$), 30.9 (CH$_{2\{42\}}$), 29.9 (CH$_{2\{40\}}$), 28.4 (CH$_{2\{7\}}$), 19.1 (CH$_{3\{25\}}$), 19.0 (CH$_{3\{26\}}$), 15.7 (CH$_{3\{38\}}$). HRMS (ESI) : m/z calc for $C_{42}H_{48}N_4O_6Na^+$: 727.3466 [M+Na]$^+$; found : 727.3474.

4-(((S)-1-(((S)-1-(((S)-1-carboxy-2-phenylethyl)amino)-3-methyl-1-oxobutan-2-yl)amino)-1-oxo-3-phenylpropan-2-yl)amino)-4-oxobutanoic acid **P24L**. ^{1}H NMR (400 MHz, MeOD) δ 7.31 – 7.04 (m, 10H$_{\{8-12, 26-30\}}$), 4.71 – 4.51 (overlapped signals : m, 2H$_{\{4, 21\}}$), 4.18 (d, J = 7.5 Hz, 1H$_{\{14\}}$), 3.18 (dd, J = 14.0, 5.3 Hz, 1H$_{\{4\}}$), 3.08 (dd, J = 14.1, 5.0 Hz, 1H$_{\{24\}}$), 2.99 (dd, J = 13.9, 8.4 Hz, 1H$_{\{4\}}$), 2.83 (dd, J = 14.1, 9.5 Hz, 1H$_{\{24\}}$), 2.60 – 2.34 (m, 4H$_{\{32, 34\}}$), 2.03 (h, J = 6.9 Hz, 1H$_{\{17\}}$), 0.90 (overlapped signals : t, J = 6.9 Hz, 6H$_{\{18, 19\}}$). ^{13}C NMR (101 MHz, MeOD) δ 176.1 (C$_{\{35\}}$), 174.4 (2C$_{\{2, 31\}}$), 173.5 (C$_{\{13\}}$), 173.1 (C$_{\{20\}}$), 138.5 (C$_{\{25\}}$), 138.3 (C$_{\{7\}}$), 130.3 (2CH$_{\{27, 29\}}$), 130.2 (2CH$_{\{9, 11\}}$), 129.5 (2CH$_{\{26, 30\}}$), 129.4 (2CH$_{\{8, 12\}}$), 127.8 (CH$_{\{28\}}$), 127.6 (CH$_{\{10\}}$), 60.1 (CH$_{\{14\}}$), 55.9 (CH$_{\{4\}}$), 55.1 (CH$_{\{21\}}$), 38.6 (CH$_{2\{6\}}$), 38.5 (CH$_{2\{24\}}$), 32.1 (CH$_{\{17\}}$), 31.5 (CH$_{2\{34\}}$), 30.2 (CH$_{2\{32\}}$), 19.7 (CH$_{3\{18\}}$), 18.7 (CH$_{3\{19\}}$). HRMS (ESI) : m/z calc for $C_{27}H_{32}N_3O_7^-$: 510.2246 [M-H]$^-$; found : 510.2231.

(S)-2-((S)-2-amino-3-phenylpropanamido)-N-((S)-1-((4-ethylphenyl)amino)-1-oxo-3-phenylpropan-2-yl)-3-methylbutanamide **P25L**. ^{1}H NMR (300 MHz, MeOD) δ 7.35 (Broad *A*B d, J = 8.5 Hz, 2H$_{\{2, 4\}}$), 7.32 – 7.15 (m, 10H$_{\{15-19, 33-37\}}$), 7.12 (Broad A*B* d, J = 8.5 Hz, 2H$_{\{1, 5\}}$), 4.72 (dd, J = 8.0, 6.9 Hz, 1H$_{\{11\}}$), 4.18 (d, J = 7.2 Hz, 1H$_{\{21\}}$), 3.78 (dd, J = 8.2, 5.1 Hz, 1H$_{\{28\}}$), 3.18 (dd, J = 13.6, 6.9 Hz, 1H$_{\{13\}}$), 3.12 – 2.96 (m, 2H$_{\{31\}}$), 2.81 (dd, J = 13.8, 8.2 Hz, 1H$_{\{13\}}$), 2.60 (q, J = 7.6 Hz, 2H$_{\{7\}}$), 2.02 (h, J = 6.8 Hz, 1H$_{\{24\}}$), 1.20 (t, J = 7.6 Hz, 3H$_{\{38\}}$), 0.87 (overlapped signals : 2d, J = 6.8 Hz, 6H$_{\{25, 26\}}$). ^{13}C NMR (101 MHz, MeOD-CDCl$_3$ (1 : 1 ; v : v)) δ 174.3 (C$_{\{27\}}$), 172.4 (C$_{\{9\}}$), 170.5 (C$_{\{20\}}$), 141.3 (C$_{\{6\}}$), 137.3 (C$_{\{32\}}$), 137.1 (C$_{\{14\}}$), 135.9 (C$_{\{3\}}$), 129.9 (2CH$_{\{34, 36\}}$), 129.9 (2CH$_{\{16, 18\}}$), 129.3 (2CH$_{\{33, 37\}}$), 129.0 (2CH$_{\{1, 5\}}$), 128.7 (2CH$_{\{15, 19\}}$), 127.6 (CH$_{\{35\}}$), 127.4 (CH$_{\{17\}}$), 121.2 (2CH$_{\{2, 4\}}$), 59.5 (CH$_{\{21\}}$), 56.2 (CH$_{\{28\}}$), 55.8 (CH$_{\{11\}}$), 40.6 (CH$_{2\{31\}}$), 38.8 (CH$_{2\{13\}}$), 31.5 (CH$_{\{24\}}$), 28.8 (CH$_{2\{7\}}$), 19.4 (CH$_{3\{25\}}$), 18.4 (CH$_{3\{26\}}$), 15.9 (CH$_{3\{38\}}$). HRMS (ESI) : m/z calc for $C_{40}H_{56}N_5O_4^+$: 515.3017 [M+H]$^+$; found : 515.3019.

tert-butyl (R)-(1-oxo-3-phenyl-1-((4-vinylphenyl)amino)propan-2-yl)carbamate **P3D**. ^{1}H NMR (300 MHz, MeOD) δ 7.45 (Broad *A*B, d, J = 8.6 Hz, 2H$_{\{1, 5\}}$), 7.35 (Broad A*B*, d, J = 8.6 Hz, 2H$_{\{2, 4\}}$), 7.29 – 7.16 (m, 5H$_{\{15-19\}}$), 6.68 (dd, J = 17.6, 11.0 Hz, 1H$_{\{7\}}$), 5.70 (dd, J = 18.38, 17.6 Hz, 1H$_{\{8\}}$), 5.16 (dd, J = 11.70, 10.9 Hz, 1H$_{\{8\}}$), 4.43 (m, 1H$_{\{12\}}$), 3.11 (dd, J = 13.6, 6.4 Hz, 1H$_{\{13\}}$), 2.93 (dd, J = 13.5, 8.4 Hz, 1H$_{\{13\}}$), 1.38 (s, 9H$_{\{25-27\}}$). ^{13}C NMR (75 MHz, CDCl$_3$) δ 171.1 (C$_{\{10\}}$), 156.5 (C$_{\{21\}}$), 137.4 (C$_{\{3\}}$), 136.9 (C$_{\{14\}}$), 136.2 (CH$_{\{7\}}$), 133.5 (C$_{\{6\}}$), 129.4 (2CH$_{\{16, 18\}}$), 128.4 (2CH$_{\{1, 5\}}$), 126.7 (CH$_{\{17\}}$), 126.5 (2CH$_{\{15, 19\}}$), 120.1 (2CH$_{\{2-4\}}$), 112.8 (CH$_{2\{8\}}$), 80.1 (C$_{\{24\}}$), 57.0 (CH$_{\{12\}}$), 39.2 (CH$_{2\{13\}}$), 28.3 (3CH$_{3\{25-27\}}$). HRMS (ESI) : m/z calc for $C_{22}H_{27}N_2O_3^+$: 367.2016 [M+H]$^+$; found : 367.2016.

Benzyl (tert-butoxycarbonyl)-D-phenylalanyl-D-valinate **P4D.** ^{1}H NMR (300 MHz, CDCl$_3$) δ 7.35 – 7.19 (m, 5H$_{\{1-5\}}$), 7.19 – 7.04 (m, 5H$_{\{21-25\}}$), 6.31 (d, J = 8.6 Hz, 1H$_{\{12\}}$), 5.05 (s, 2H$_{\{7\}}$), 4.92 (m, 1H$_{\{26\}}$), 4.43 (dd, J = 8.6, 4.9 Hz, 1H$_{\{10\}}$), 4.27 (q, J = 7.3 Hz, 1H$_{\{18\}}$), 2.98 (d, J = 6.9 Hz, 2H$_{\{19\}}$), 2.18 – 1.97 (m, 1H$_{\{13\}}$), 1.33 (s, 9H$_{\{31-33\}}$), 0.77 (d, J = 6.9 Hz, 3H$_{\{14\}}$), 0.72 (d, J = 6.9 Hz, 3H$_{\{15\}}$).

(Tert-butoxycarbonyl)-D-phenylalanyl-D-valine **P5D**. ^{1}H NMR (300 MHz, CDCl$_3$) δ 9.55 (s, 1H$_{\{19\}}$), 7.37 – 7.11 (m, 5H$_{\{9-13\}}$), 6.87 – 6.69 (m, 1H$_{\{5\}}$), 5.58 – 5.38 (m, 1H$_{\{1\}}$), 4.62 – 4.41 (overlapped signals : m, 2H$_{\{2, 14\}}$), 3.20 – 2.99 (m, 2H$_{\{4\}}$), 2.25 (h, J = 6.9 Hz, 1H$_{\{16\}}$), 1.44 (s, 9H$_{\{24-26\}}$), 0.96 (overlapped signals, t, J = 7.0 Hz, 6H$_{\{17,18\}}$). ^{13}C NMR (75 MHz, CDCl$_3$) δ 174.8 (C$_{\{15\}}$), 172.0 (C$_{\{3\}}$), 155.9 (C$_{\{7\}}$), 136.6 (C$_{\{8\}}$), 129.4 (2CH$_{\{10, 12\}}$), 128.7 (2CH$_{\{9, 13\}}$), 127.0 (CH$_{\{11\}}$), 80.6 (C$_{\{23\}}$), 57.4 (CH$_{\{14\}}$), 55.9 (CH$_{\{2\}}$), 38.2 (CH$_{2\{4\}}$), 31.2 (CH$_{\{16\}}$), 28.3 (3CH$_{3\{24-26\}}$), 19.0 (CH$_{3\{17\}}$), 17.8 (CH$_{3\{18\}}$).

Benzyl (tert-butoxycarbonyl)-D-phenylalanyl-D-valyl-D-phenylalaninate **P6D**. ^{1}H NMR (400 MHz, CDCl$_3$) δ 7.39 – 7.31 (m, 3H$_{\{41, 43\}}$), 7.29 – 7.15 (m, 10H$_{\{8-13, 34-38\}}$), 7.08 – 7.00 (m, 2H$_{\{40-44\}}$), 6.62 (overlapped signals : 2d, J = 9.0, 8.3 Hz, 2H$_{\{6, 17\}}$), 5.19 – 5.04 (overlapped signals : , 3H$_{\{24, 1\}}$), 4.89 (dt, J = 8.0, 6.2 Hz, 1H$_{\{5\}}$), 4.38 (d, J = 7.3 Hz, 1H$_{\{22\}}$), 4.27 (dd, J = 8.6, 6.4 Hz, 1H$_{\{15\}}$), 3.14 – 2.97 (m, 4H$_{\{7, 25\}}$), 2.05 (h, J = 6.7 Hz, 1H$_{\{18\}}$), 1.40 (s, 9H$_{\{31-33\}}$), 0.85 (d, J = 6.8 Hz, 3H$_{\{19\}}$), 0.81 (d, J = 6.8 Hz, 3H$_{\{20\}}$). ^{13}C NMR (101 MHz, CDCl$_3$) δ 171.5 (C$_{\{3\}}$), 171.2 (C$_{\{14\}}$), 170.1 (C$_{\{21\}}$), 155.6 (C$_{\{27\}}$), 136.8 (CH$_{\{26\}}$), 135.7 (CH$_{\{39\}}$), 135.2 (CH$_{\{8\}}$), 129.4 (2CH$_{\{35, 37\}}$), 129.4 (2CH$_{\{10, 12\}}$), 128.8 (2CH$_{\{34, 38\}}$), 128.7 (2CH$_{\{9, 13\}}$), 128.6 (4CH$_{\{40, 41, 43, 45\}}$), 128.6 (CH$_{\{42\}}$), 127.2 (CH$_{\{36\}}$), 127.0 (2CH$_{\{11\}}$), 80.3 (C$_{\{30\}}$), 67.3 (CH$_{2\{1\}}$), 58.5 (CH$_{\{15\}}$), 55.9 (CH$_{\{5\}}$), 53.4 (CH$_{\{22\}}$), 38.0 (2CH$_{2\{7, 25\}}$), 31.0 (CH$_{\{18\}}$), 28.4 (3CH$_{3\{31-33\}}$), 19.1 (CH$_{3\{19\}}$), 18.0 (CH$_{3\{20\}}$). HRMS (ESI) : m/z calc for $C_{35}H_{43}N_3O_6Na^+$: 624.3044 [M+Na]$^+$; found : 624.3052.

(Tert-butoxycarbonyl)-D-phenylalanyl-D-valyl-D-phenylalanine ^{1}H NMR (400 MHz, CDCl$_3$) δ 7.42 – 7.11 (m, 10H$_{\{8-12, 26-30\}}$), 7.04 (d, J = 8.7 Hz, 1H$_{\{16\}}$), 6.81 (broad s, 1H$_{\{5\}}$), 5.20 (s, 1H$_{\{23\}}$), 4.79 (q, J = 6.7 Hz, 1H$_{\{4\}}$), 4.43 (broad s, 1H$_{\{21\}}$), 4.29 (dd, J = 8.8, 6.9 Hz, 1H$_{\{14\}}$), 3.18 (dd, J = 14.0, 5.7 Hz, 1H$_{\{24\}}$), 3.10 – 2.90 (overlapped signals : m, 3H$_{\{24, 6\}}$), 2.00 (h, J = 6.8 Hz, 1H$_{\{17\}}$), 1.39 (s, 9H$_{\{35-37\}}$), 0.86 (d, J = 6.7 Hz, 3H$_{\{19\}}$), 0.79 (d, J = 6.7 Hz, 3H$_{\{18\}}$). ^{13}C NMR (101 MHz, CDCl$_3$) δ 173.8 (C$_{\{2\}}$), 172.1 (C$_{\{13\}}$), 171.2 (C$_{\{20\}}$), 155.8 (C$_{\{31\}}$), 136.7 (C$_{\{25\}}$), 136.1 (C$_{\{7\}}$), 129.5 (2CH$_{\{27, 29\}}$), 129.4 (2CH$_{\{9, 11\}}$), 128.6 (4CH$_{\{26, 30, 8, 12\}}$), 127.1 (CH$_{\{28\}}$), 126.9 (CH$_{\{10\}}$), 80.4 (C$_{\{34\}}$), 58.7 (CH$_{\{14\}}$), 55.8 (CH$_{\{4\}}$), 53.6 (CH$_{\{21\}}$), 38.2 (CH$_{2\{24\}}$), 37.7 (CH$_{2\{6\}}$), 31.2 (CH$_{\{17\}}$), 28.3 (3CH$_{3\{35-37\}}$), 19.1 (CH$_{3\{19\}}$), 18.2 (CH$_{3\{18\}}$). HRMS (ESI) : m/z calc for $C_{28}H_{37}N_3O_6Na^+$: 534.2575 [M+Na]$^+$; found : 534.2580

(R)-1-(((R)-1-(((R)-1-(benzyloxy)-1-oxo-3-phenylpropan-2-yl)amino)-3-methyl-1-oxobutan-2-yl)amino)-1-oxo-3-phenylpropan-2-aminium trifluoroacetate **P8D**. ^{1}H NMR (400 MHz, MeOD) δ 7.37 – 7.15 (m, 15H$_{\{2-6, 16-20, 34-38\}}$), 5.11 (s, 2H$_{\{8\}}$), 4.72 (dd, J = 8.4, 6.1 Hz, 1H$_{\{12\}}$), 4.22 (d, J = 7.2 Hz, 1H$_{\{22\}}$), 4.05 (dd, J = 8.6, 5.3 Hz, 1H$_{\{29\}}$), 3.16 (dd, J = 13.9, 6.1 Hz, 1H$_{\{14\}}$), 3.10 (dd, J = 14.2, 5.3 Hz, 1H$_{\{32\}}$), 3.01 (dd, J = 13.9, 8.4 Hz, 1H$_{\{14\}}$), 2.87 (dd, J = 14.3, 8.6 Hz, 1H$_{\{32\}}$), 2.00 (dq, J = 13.8, 6.9 Hz, 1H$_{\{25\}}$), 0.90 (overlapped signals : 2d, J = 6.8 Hz, 6H$_{\{26, 27\}}$). ^{13}C NMR (101 MHz, MeOD) δ 172.9 (C$_{\{10\}}$), 172.5 (C$_{\{21\}}$), 170.5 (C$_{\{28\}}$), 137.9 (C$_{\{32\}}$), 136.9 (C$_{\{7\}}$), 135.9 (C$_{\{15\}}$), 130.5 (2CH$_{\{35, 37\}}$), 130.3 (2CH$_{\{17, 19\}}$), 130.0 (2CH$_{\{3, 5\}}$), 129.5 (2CH$_{\{34, 38\}}$), 129.5 (2CH$_{\{2, 6\}}$), 129.4 (2CH$_{\{16, 20\}}$), 129.3 (CH$_{\{4\}}$), 128.6 (CH$_{\{36\}}$), 127.9 (CH$_{\{18\}}$), 68.0 (CH$_{2\{8\}}$), 60.0 (CH$_{\{22\}}$), 55.6 (CH$_{\{29\}}$), 55.4 (CH$_{\{12\}}$), 39.1 (CH$_{2\{32\}}$), 38.4 (CH$_{\{25\}}$), 32.1 (CH$_{2\{8\}}$), 19.6 (CH$_{\{26\}}$), 18.6 (CH$_{2\{27\}}$). ^{19}F NMR (376 MHz, MeOD) δ -76.97. HRMS (ESI) : m/z calc for cation $C_{30}H_{36}N_3O_4^+$: 502.2700 ; found : 502.2701. m/z calc for anion $C_2F_3O_2^-$: 112.9856 ; found : 112.9856.

(R)-1-(((R)-1-(((R)-1-carboxy-2-phenylethyl)amino)-3-methyl-1-oxobutan-2-yl)amino)-1-oxo-3-phenylpropan-2-aminium trifluoroacetate **P11D**. ^{1}H NMR (400 MHz, MeOD) δ 7.33 – 7.07 (m, 10H$_{\{16-20, 34-38\}}$), 4.65 (dd, J = 8.5, 5.0 Hz, 1H$_{\{12\}}$), 4.23 (d, J = 7.4 Hz, 1H$_{\{22\}}$), 4.13 (dd, J = 8.8, 5.2 Hz, 1H$_{\{29\}}$), 3.22 (dd, J = 14.0, 5.0 Hz, 1H$_{\{14\}}$), 3.13 (dd, J = 14.4, 5.2 Hz, 1H$_{\{32\}}$), 3.00 (dd, J = 14.0, 8.5 Hz, 1H$_{\{14\}}$), 2.90 (dd, J = 14.4, 8.8 Hz, 1H $_{\{32\}}$), 2.05 (h, J = 6.8 Hz, 1H$_{\{25\}}$), 0.96 (overlapped signals : 2d, J = 6.8 Hz, 6H$_{\{26, 27\}}$). ^{13}C NMR (101 MHz, DMSO) δ 172.7 (C$_{\{10\}}$), 170.3 (C$_{\{21\}}$), 167.9 (C$_{\{28\}}$), 137.6 (C$_{\{15\}}$), 134.8 (C$_{\{32\}}$), 129.4 (2CH$_{\{17, 19\}}$), 129.0 (2CH$_{\{35, 37\}}$), 128.4 (2CH$_{\{16, 20\}}$), 128.1 (2CH$_{\{34, 38\}}$), 127.0 (CH$_{\{18\}}$), 126.3 (CH$_{\{36\}}$), 57.5 (CH$_{\{22\}}$), 53.4 (CH$_{\{12\}}$), 53.1 (CH$_{\{29\}}$), 37.2 (CH$_{2\{14\}}$), 36.6 (CH$_{2\{32\}}$), 31.0 (CH$_{\{25\}}$), 19.0 (CH$_{3\{26\}}$), 18.1 (CH$_{3\{27\}}$). ^{19}F NMR (376 MHz, MeOD) δ -76.94. HRMS (ESI) : m/z calc for cation $C_{31}H_{39}N_4O_3^+$: 412.2231 ; found : 412.2232. m/z calc for anion $C_2F_3O_2^-$: 112.9856 ; found : 112.9859.

Tert-butyl ((R)-1-(((R)-1-(((R)-1-((4-ethylphenyl)amino)-1-oxo-3-phenylpropan-2-yl)amino)-3-methyl-1-oxobutan-2-yl)amino)-1-oxo-3-phenylpropan-2-yl)carbamate **P21D**. ^{1}H NMR (400 MHz, CDCl$_3$) δ 8.35 (s, 1H$_{\{9\}}$), 7.56 (Broad d, J = 7.9 Hz, 2H$_{\{2, 4\}}$), 7.33 – 7.20 (m, 5H$_{\{16-20\}}$), 7.20 – 7.06 (m, 7H$_{\{1, 5, 41-45\}}$), 6.71 (d, J = 8.3 Hz, 1H$_{\{13\}}$), 6.65 (s, 1H$_{\{24\}}$), 4.98 (d, J = 5.8 Hz, 1H$_{\{31\}}$), 4.90 (td, J = 8.7, 5.7 Hz, 1H$_{\{12\}}$), 4.36 (q, J = 6.4 Hz, 1H$_{\{29\}}$), 4.17 (dd, J = 6.4, 4.7 Hz, 1H$_{\{22\}}$), 3.47 (Broad d, J = 14.2 Hz, 1H$_{\{32\}}$), 3.10 (dd, J = 14.0, 6.1 Hz, 1H$_{\{14\}}$), 3.01 (dd, J = 14.0, 7.3 Hz, 1H$_{\{14\}}$), 2.90 (dd, J = 14.6, 9.6 Hz, 1H$_{\{32\}}$), 2.61 (q, J = 7.6 Hz, 2H $_{\{7\}}$), 2.11 (ddd, J = 13.8, 9.4, 5.9 Hz, 1H$_{\{25\}}$), 1.38 (s, 9H$_{\{38-40\}}$), 1.21 (t, J = 7.6 Hz, 3H$_{\{46\}}$), 0.76 (d, J = 6.9 Hz, 3H$_{\{26\}}$), 0.57 (d, J = 6.9 Hz, 3H$_{\{27\}}$). ^{13}C NMR (101 MHz, CDCl$_3$) δ 172.4 (C$_{\{10\}}$), 172.1 (C$_{\{21\}}$), 169.9 (C$_{\{28\}}$), 155.9 (C$_{\{34\}}$), 140.3 (C$_{\{6\}}$), 137.2 (C$_{\{33\}}$), 136.6 (C$_{\{15\}}$), 135.3 (C$_{\{3\}}$), 129.6 (2CH$_{\{42, 44\}}$), 129.4 (2CH$_{\{17, 19\}}$), 128.5 (2CH$_{\{41, 45\}}$), 128.3 (2CH$_{\{16, 20\}}$), 128.0 (2CH$_{\{1, 5\}}$), 126.7 (CH$_{\{43\}}$), 126.6 (CH$_{\{18\}}$), 121.2 (2CH$_{\{2, 4\}}$), 79.6 (C$_{\{37\}}$), 58.3 (CH$_{\{22\}}$), 55.6 (CH$_{\{12\}}$), 55.5 (CH$_{\{29\}}$), 39.5 (CH$_{2\{14\}}$), 38.7 (CH$_{2\{32\}}$), 32.0 (CH$_{\{25\}}$), 28.5 (CH$_{3\{38-40\}}$), 28.4 (CH$_{2\{7\}}$), 19.3 (CH$_{3\{26\}}$), 18.4 (CH$_{3\{27\}}$), 15.6 (CH$_{3\{46\}}$). HRMS (ESI) : m/z calc for $C_{36}H_{46}N_4O_5Na^+$: 637.3360 [M+Na]$^+$; found : 637.3359.

(R)-1-(((R)-1-(((R)-1-((4-ethylphenyl)amino)-1-oxo-3-phenylpropan-2-yl)amino)-3-methyl-1-oxobutan-2-yl)amino)-1-oxo-3-phenylpropan-2-aminium trifluoroacetate **P22D**. ^{1}H NMR (400 MHz, MeOD) δ 7.36 (Broad *A*B, d, J = 8.5 Hz, 2H$_{\{2, 4\}}$), 7.31 – 7.15 (m, 10H$_{\{16-20, 34-38\}}$), 7.12 (d, J = 8.5 Hz, 2H$_{\{1, 5\}}$), 4.72 (dd, J = 8.1, 6.9 Hz, 1H$_{\{12\}}$), 4.15 (d, J = 7.1 Hz, 1H$_{\{22\}}$), 3.66 (dd, J = 8.0, 5.1 Hz, 1H$_{\{29\}}$), 3.18 (dd, J = 13.7, 6.9 Hz, 1H$_{\{14\}}$), 3.08 – 2.95 (overlapped signals : dd, J = 13.7, 5.0 Hz, 1H$_{\{32\}}$, dd, J = 13.5, 7.9 Hz, 1H$_{\{32\}}$), 2.77 (dd, J = 13.6, 8.0 Hz, 1H$_{\{14\}}$), 2.60 (q, J = 7.6 Hz, 2H$_{\{7\}}$), 2.00 (h, J = 6.8 Hz, 1H$_{\{25\}}$), 1.20 (t, J = 7.6 Hz, 3H$_{\{8\}}$), 0.85 (overlapped signals : 2d, J = 6.8 Hz, 6H$_{\{26, 27\}}$). ^{13}C NMR (101 MHz, MeOD-CDCl$_3$ (1 : 1 ; v : v)) δ 176.0 (C$_{\{10\}}$), 172.5 (C$_{\{21\}}$), 170.5 (C$_{\{28\}}$), 141.3 (C$_{\{6\}}$), 137.7 (C$_{\{33\}}$), 137.3 (C$_{\{15\}}$), 135.9 (C$_{\{3\}}$), 129.9 (4CH$_{\{17, 19, 35, 37\}}$), 129.2 (2CH$_{\{1, 5\}}$), 129.0 (2CH$_{\{34, 38\}}$), 128.7 (2CH$_{\{16, 20\}}$), 127.5 (CH$_{\{36\}}$), 127.4 (CH$_{\{18\}}$), 121.3 (2CH$_{\{2, 4\}}$), 59.5 (CH$_{\{22\}}$), 56.6 (CH$_{\{29\}}$), 55.8 (CH$_{\{12\}}$), 41.3 (CH$_{2\{32\}}$), 38.8 (CH$_{2\{14\}}$), 31.5 (CH$_{\{25\}}$), 28.8 (CH$_{2\{7\}}$), 19.4 (CH$_{3\{26\}}$), 18.4 (CH$_{3\{27\}}$), 15.9 (CH$_{3\{8\}}$). ^{19}F NMR (376 MHz, MeOD) δ -76.51. HRMS (ESI) : m/z calc for cation $C_{31}H_{39}N_4O_3^+$: 515.3017 ; found : 515.3017. m/z calc for anion $C_2F_3O_2^-$: 112.9856 ; found : 112.9856.

tert-butyl ((2S)-1-oxo-3-phenyl-1-((4-(1-((2,2,6,6-tetramethylpiperidin-1-yl)oxy)ethyl)phenyl)amino)propan-2-yl)carbamate **A1L**. ^{1}H NMR (400 MHz, CDCl$_3$) δ 8.34 (s, 1H$_{\{9\}}$) 7.33 (Broad A*B*, d, J = 8.1 Hz, 2H$_{\{2, 4\}}$), 7.30 – 7.22 (m, 5H$_{\{15-19\}}$), 7.20

(Broad *A*B, d, J = 8.2 Hz, $2H_{\{1, 5\}}$), 5.54 (m, $1H_{\{20\}}$), 4.73 (q, J = 6.6 Hz, $1H_{\{7\}}$), 4.65 – 4.55 (m, $1H_{\{12\}}$), 3.23 – 3.02 (m, $2_{\{13\}}$), 1.60 – 1.33 (overlapped signals : m, $6H_{\{32, 35, 38\}}$, d, J = 6.6 Hz, $3H_{\{28\}}$, s, $9H_{\{25-27\}}$), 1.29 (s, $3H_{\{33\}}$), 1.16 (s, $3H_{\{36\}}$), 1.02 (s, $3H_{\{34\}}$), 0.65 (s, $3H_{\{37\}}$). ^{13}C NMR (101 MHz, $CDCl_3$) δ 169.8 ($C_{\{10\}}$), 155.8 ($C_{\{21\}}$), 141.9 ($C_{\{3\}}$), 136.7 ($C_{\{14\}}$), 136.0 ($C_{\{6\}}$), 129.3 ($2CH_{\{16, 18\}}$), 128.6 ($2CH_{\{1, 5\}}$), 127.0 ($2CH_{\{15, 19\}}$), 126.8 ($CH_{\{17\}}$), 119.7 ($2CH_{\{2, 4\}}$), 82.5 ($CH_{\{7\}}$), 80.3 ($C_{\{24\}}$), 59.5 ($C_{\{30, 31\}}$), 56,7 ($CH_{\{12\}}$), 40.3 ($2CH_{2\{32, 35\}}$), 38.6 ($CH_{2\{13\}}$), 34.3 ($CH_{3\{33\}}$), 34.3 ($CH_{3\{36\}}$), 28.2 ($3CH_{3\{25-27\}}$), 23.3 ($CH_{3\{28\}}$), 20.2 ($2CH_{3\{34,37\}}$), 17.1 ($CH_{2\{38\}}$). HRMS (ESI) : m/z calc for $C_{31}H_{46}N_3O_4^+$: 524.3483 $[M+H]^+$; found : 524.3482

(2S)-1-oxo-3-phenyl-1-((4-(1-((2,2,6,6-tetramethylpiperidin-1-yl)oxy)ethyl)phenyl)amino)propan-2-aminium trifluoroacetate **A2L**. By using GP3 with **A1L** (0.86 g, 1.643 mmol, 1.0 eq) afforded **A2L** as a white solid (0.71 g, 81 %). The product was directly used in the coupling reaction without characterization.

tert-butyl ((2S)-1-(((2S)-3-methyl-1-oxo-1-(((2S)-1-oxo-3-phenyl-1-((4-(1-((2,2,6,6-tetramethylpiperidin-1-yl)oxy)ethyl)phenyl)amino)propan-2-yl)amino)butan-2-yl)amino)-1-oxo-3-phenylpropan-2-yl)carbamate **A3L**. ^1H NMR (300 MHz, MeOD) δ 7.44 – 7.39 (m, $2H_{\{2, 4\}}$), 7.30 – 7.12 (m, $12H_{\{1, 5, 16-20, 41-45\}}$), 4.79 – 4.66 (overlapped signals, m, $2H_{\{7, 12\}}$), 4.42 (dd, J = 9.9, 5.0 Hz, $1H_{\{29\}}$), 4.25 (d, J = 7.1 Hz, $1H_{\{22\}}$), 3.18 (dd, J = 13.6, 7.1 Hz, $1H_{\{14\}}$), 3.06 (dd, J = 14.0, 5.1 Hz, $1H_{\{32\}}$), 2.99 (dd, J = 13.7, 8.0 Hz $1H_{\{14\}}$), 2.79 (dd, J = 13.7, 9.4 Hz, $1H_{\{32\}}$), 2.03 (dq, J = 6.8 Hz, $1H_{\{25\}}$), 1.60 – 0.97 (overlapped signals : m, $15H_{\{50-54, 56\}}$, d, J = 6.7 Hz, $3H_{\{46\}}$, s, $9H_{\{38-40\}}$), 0.90 – 0.80 (overlapped signals, m, $6H_{\{26, 27\}}$), 0.61 (s, $3H_{\{55\}}$). ^{13}C NMR (101 MHz, $CDCl_3$) δ 172.4 ($C_{\{10\}}$), 172.0 ($C_{\{21\}}$), 169.7 ($C_{\{28\}}$), 156.0 ($C_{\{34\}}$), 142.0 ($C_{\{3\}}$, dia 1), 141.9 ($C_{\{3\}}$, dia 2), 136.9 ($C_{\{33\}}$), 136.6 ($C_{\{15\}}$), 136.5 ($C_{\{6\}}$), 129.5 ($2CH_{\{42, 44\}}$), 129.3 ($2CH_{\{17, 19\}}$), 128.6 ($2CH_{\{41, 45\}}$), 128.5 ($2CH_{\{16, 20\}}$), 127.1 ($CH_{\{43\}}$), 127.0 ($CH_{\{18\}}$), 126.8 ($CH_{\{1\}}$), 126.7 ($CH_{\{5\}}$), 120.4 ($2CH_{\{2, 4\}}$), 82.8 ($CH_{\{7\}}$, dia 1), 82.7 ($CH_{\{43\}}$, dia 2), 79.9 ($C_{\{37\}}$), 59.7 ($2C_{\{48, 49\}}$), 58.4 ($CH_{\{22\}}$), 55.7 ($CH_{\{12\}}$), 55.5 ($CH_{\{29\}}$), 40.5 ($2CH_{2\{51, 53\}}$), 39.3 ($CH_{2\{32\}}$), 38.6 ($CH_{2\{14\}}$), 34.4 ($2CH_{3\{50, 52\}}$), 31.7 ($CH_{\{25\}}$), 28.5 ($3CH_{3\{38-40\}}$), 23.5 ($CH_{3\{46\}}$, dia 1), 23.4 ($CH_{3\{46\}}$, dia 2), 20.4 ($2CH_{3\{54, 55\}}$), 19.3 ($CH_{3\{26\}}$), 18.1 ($CH_{3\{27\}}$), 17.3 ($CH_{2\{56\}}$). HRMS (ESI) : m/z calc for $C_{45}H_{64}N_5O_6^+$: 770.4851 $[M+H]^+$; found : 770.4852.

(2S)-1-(((2S)-3-methyl-1-oxo-1-(((2S)-1-oxo-3-phenyl-1-((4-(1-((2,2,6,6-tetramethylpiperidin-1-yl)oxy)ethyl)phenyl)amino)propan-2-yl)amino)butan-2-yl)amino)-1-oxo-3-phenylpropan-2-aminium trifluoroacetate **A4L**. ^1H NMR (300 MHz, MeOD) δ 7.41 (Broad *A*B, d, *J* = 8.5 Hz, $2H_{\{2, 4\}}$), 7.36 – 7.09 (m, $12H_{\{1, 5, 16-20, 34-38\}}$), 4.76 (overlapped signal : t, *J* = 7.4 Hz, $1H_{\{12\}}$ q, *J* = 6.6 Hz, $1H_{\{7\}}$), 4.30 (d, *J* = 7.6 Hz, $1H_{\{22\}}$), 4.20 (dd, *J* = 8.4, 5.4 Hz, $1H_{\{29\}}$), 3.24 – 3.10 (m, $2H_{\{14, 32\}}$), 3.03 (dd, *J* = 13.7, 8.0 Hz, $1H_{\{14\}}$), 2.93 (dd, *J* = 14.3, 8.5 Hz, $1H_{\{32\}}$), 2.03 (dq, *J* = 12.4, *J* = 6.1 Hz, $1H_{\{25\}}$), 1.62 – 1.00 (overlapped signals : m, $6H_{\{44, 46, 49\}}$, d, *J* = 6.6 Hz, $3H_{\{39\}}$, m, $9H_{\{43, 45, 47\}}$), 0.95 – 0.85 (Overlapped signals: d, J = 6.6 Hz, $3H_{\{27\}}$, d, *J* = 6.6 Hz, $3H_{\{26\}}$), 0.62 (s, $3H_{\{48\}}$). ^{13}C NMR (75 MHz, MeOD) δ 172.9 ($C_{\{10\}}$), 171.4 ($C_{\{21\}}$), 170.2 ($C_{\{28\}}$), 143.0 ($C_{\{3\}}$), 138.1 ($C_{\{33\}}$), 138.0 ($C_{\{15\}}$), 135.6 ($C_{\{6\}}$), 130.5 ($2CH_{\{35, 37\}}$), 130.4 ($2CH_{\{17, 19\}}$), 130.0 ($2CH_{\{34, 38\}}$), 129.5 ($2CH_{\{16, 20\}}$), 128.7 ($CH_{\{36\}}$), 128.1 ($2CH_{\{1, 5\}}$), 127.9 ($CH_{\{18\}}$), 121.1 ($2CH_{\{2, 4\}}$), 84.0 ($CH_{\{7\}}$), 60.9 ($2C_{\{41, 42\}}$), 60.4 ($CH_{\{22\}}$), 56.8 ($CH_{\{29\}}$), 55.5 ($CH_{\{12\}}$), 41.3 ($2CH_{2\{44, 46\}}$), 39.3 ($CH_{2\{32\}}$), 38.8 ($CH_{2\{14\}}$), 34.7 ($2CH_{3\{43, 45\}}$), 32.1 ($CH_{\{25\}}$), 23.7 ($CH_{3\{39\}}$), 20.8 ($2CH_{3\{47, 48\}}$), 19.6 ($CH_{3\{27\}}$) , 18.5 ($CH_{3\{26\}}$), 18.1 ($CH_{2\{49\}}$). ^{19}F NMR (376 MHz, MeOD) δ -76.83. HRMS (ESI) : m/z calc for cation $C_{40}H_{56}N_5O_4^+$: 670.4327 ; found : 670.4329. m/z calc for anion $C_2F_3O_2^-$: 112.9856 ; found : 112.9857.

(2S)-2-((S)-2-amino-3-phenylpropanamido)-3-methyl-N-((2S)-1-oxo-3-phenyl-1-((4-(1-((2,2,6,6-tetramethylpiperidin-1-yl)oxy)ethyl)phenyl)amino)propan-2-yl)butanamide **A5L**. ^1H NMR (400 MHz, MeOD) δ 7.42 (broad *A*B d, J = 8.5 Hz, $2H_{\{2, 4\}}$), 7.32 – 7.12 (m, $12H_{\{1, 5, 16-20, 34-38\}}$), 4.78 – 4.67 (overlapped signals : m, $2H_{\{12, 7\}}$), 4.17 (d, J = 7.2 Hz, $1H_{\{22\}}$), 3.77 (m,$1H_{\{29\}}$), 3.19 (dd, J = 13.6, 6.9 Hz, $1H_{\{32\}}$), 3.07 (dd, J = 13.8, 5.1 Hz, $1H_{\{32\}}$), 3.01 (dd, J = 13.7, 8.2 Hz, $1H_{\{14\}}$), 2.81 (dd, J = 13.8, 8.2 Hz, $1H_{\{14\}}$), 2.01 (h, J = 6.8 Hz, $1H_{\{25\}}$), 1.69 – 0.98 (overlapped signals : m, $6H_{\{44, 46, 49\}}$, d, J = 6.6 Hz, $3H_{\{39\}}$, s, $3H_{\{43\}}$, s, $3H_{\{45\}}$, s, $3H_{\{47\}}$), 0.89 – 0.84 (overlapped signals : d, J = 6.7 Hz, $3H_{\{27\}}$, d, J = 6.6 Hz, $3H_{\{26\}}$), 0.62 (s, $3H_{\{48\}}$). ^{13}C NMR (101 MHz, MeOD-$CDCl_3$ (1 : 1 ; v : v)) δ 175.7 ($C_{\{28\}}$), 172.3 ($C_{\{10\}}$), 170.3 ($C_{\{21\}}$), 142.5 ($C_{\{3\}}$), 137.7 ($C_{\{33\}}$), 137.2 ($C_{\{15\}}$), 136.9 ($C_{\{6\}}$), 129.8 ($4CH_{\{17, 19, 35, 37\}}$), 129.2 ($2CH_{\{34, 38\}}$), 128.9 ($2CH_{\{16, 20\}}$), 127.5 ($2CH_{\{1, 5\}}$), 127.4 ($CH_{\{36\}}$), 127.3 ($2CH_{\{18\}}$), 120.5 ($2CH_{\{2, 4\}}$), 83.2 ($CH_{\{7\}}$), 60.3 ($2C_{\{41, 42\}}$), 59.3 ($CH_{\{22\}}$), 56.6 ($CH_{\{29\}}$), 55.6 ($CH_{\{12\}}$), 41.2 ($CH_{2\{32\}}$), 40.7 ($2CH_{2\{44, 46\}}$), 38.6 ($CH_{2\{14\}}$), 34.4 ($2CH_{3\{43, 45\}}$), 31.3 ($CH_{\{25\}}$), 23.7 ($CH_{3\{39\}}$), 20.7 ($2CH_{3\{47, 48\}}$), 19.4 ($CH_{3\{26\}}$), 18.2 ($CH_{3\{27\}}$), 17.6 ($CH_{2\{49\}}$). HRMS (ESI) : m/z calc for $C_{40}H_{56}N_5O_4^+$: 670.4327 $[M+H]^+$; found : 670.4329.

Benzyl 4-(((2S)-1-(((2S)-3-methyl-1-oxo-1-(((2S)-1-oxo-3-phenyl-1-((4-(1-((2,2,6,6-tetramethylpiperidin-1-yl)oxy)ethyl)phenyl)amino)propan-2-yl)amino)butan-2-yl)amino)-1-oxo-3-phenylpropan-2-yl)amino)-4-oxobutanoate **A6L**. ^1H NMR (400 MHz, MeOD) δ 7.45 (broad *A*B, d, J = 8.5 Hz, $2H_{\{2, 4\}}$), 7.36 – 7.27 (m, $5H_{\{59-63\}}$), 7.26 – 7.12 (m, $12H_{\{1,5, 16-20, 34-38\}}$), 5.12 – 5.01 (m, $2H_{\{57\}}$), 4.79 – 4.64 (overlapped signals : m, $3H_{\{7, 12, 29\}}$), 4.15 (d, J = 7.1 Hz, $1H_{\{22\}}$), 3.20 (dd, J = 13.7, 6.8 Hz, $1H_{\{14\}}$), 3.11 (dd, J = 14.1, 5.2 Hz, $1H_{\{32\}}$), 2.98 (dd, J = 13.7, 8.2 Hz, $1H_{\{14\}}$), 2.86 (dd, J = 14.1, 9.4 Hz, $1H_{\{32\}}$), 2.67 – 2.37 (m, $4H_{\{51, 53\}}$), 2.00 (h, J = 6.8 Hz, $1H_{\{25\}}$), 1.70 – 0.93 (overlapped signals : $6H_{\{44, 46, 49\}}$, d, J = 6.7 Hz, $3H_{\{39\}}$, three broad s, $9H_{\{45, 47, 48\}}$), 0.83 (overlapped signals : 2d, J = 6.8 Hz, $6H_{\{26, 27\}}$), 0.62 (s, $3H_{\{43\}}$). ^{13}C NMR (101 MHz, $CDCl_3$ /

C_6D_6 : 19 : 1) δ 172.7 (C$_{\{50\}}$), 171.9 (C$_{\{10\}}$), 171.7 (2C$_{\{21, 54\}}$), 169.9 (C$_{\{28\}}$), 141.9 (C$_{\{3\}}$, dia 1), 141.8 (C$_{\{3\}}$, dia 2), 137.0 (C$_{\{33\}}$), 136.8 (C$_{\{58\}}$), 136.7 (C$_{\{15\}}$), 136.1 (C$_{\{6\}}$), 129.8 (2CH$_{\{35, 37\}}$), 129.6 (2CH$_{\{17, 19\}}$), 128.6 (2CH$_{\{60, 62\}}$), 128.4 (4CH$_{\{16, 20, 34, 38\}}$), 128.3 (CH$_{\{61\}}$), 128.2 (2CH$_{\{59, 63\}}$) , 127.1 (CH$_{\{36\}}$), 127.0 (CH$_{\{18\}}$), 126.8 (2CH$_{\{1, 5\}}$), 120.3 (2CH$_{\{2, 4\}}$), 82.8 (CH$_{\{7\}}$, dia 1), 82.7 (CH$_{\{7\}}$, dia 2), 66.4 (CH$_{2\{57\}}$), 59.8 (C$_{\{41\}}$), 59.7 (C$_{\{42\}}$), 58.4 (CH$_{\{22\}}$), 55.3 (CH$_{\{12\}}$), 54.0 (CH$_{\{29\}}$), 40.5 (2CH$_{2\{44, 46\}}$), 40.0 (CH$_{2\{14\}}$), 39.2 (CH$_{2\{32\}}$), 34.5 (2CH$_{3\{43, 45\}}$), 32.1 (CH$_{\{25\}}$), 30.9 (CH$_{2\{53\}}$), 29.8 (CH$_{2\{51\}}$), 23.6 (CH$_{3\{39\}}$, dia 1), 23.4 (CH$_{3\{39\}}$, dia 2), 20.5 (2CH$_{3\{47, 48\}}$), 19.1 (CH$_{3\{26\}}$), 18.9 (CH$_{3\{27\}}$), 17.4 (CH$_{2\{49\}}$). HRMS (ESI) : m/z calc for $C_{51}H_{66}N_5O_7^+$: 860.4957 [M+H]$^+$; found : 860.4930.

4-(((2S)-1-(((2S)-3-methyl-1-oxo-1-(((2S)-1-oxo-3-phenyl-1-((4-(1-((2,2,6,6-tetramethylpiperidin-1-yl)oxy)ethyl)phenyl)amino)propan-2-yl)amino)butan-2-yl)amino)-1-oxo-3-phenylpropan-2-yl)amino)-4-oxobutanoic acid **A7L**. ^{1}H NMR (400 MHz, MeOD) δ 7.52 – 7.41 (m, 2H$_{\{2, 4\}}$), 7.28 – 7.05 (overlapped signals : m, 12H$_{\{1, 5, 16\text{-}20, 34\text{-}38\}}$), 4.79 – 4.59 (overlapped signals : m, 3H$_{\{7, 14, 32\}}$), 4.12 (d, J = 7.3 Hz, 1H$_{\{22\}}$), 3.22 (dd, J = 13.7, 6.6 Hz, 1H$_{\{14\}}$), 3.13 (dd, J = 14.1, 5.1 Hz, 1H$_{\{32\}}$), 2.99 (dd, J = 13.7, 8.4 Hz, 1H$_{\{14\}}$), 2.88 (dd, J = 14.1, 9.4 Hz, 1H$_{\{32\}}$), 2.63 – 2.31 (m, 4H$_{\{51, 53\}}$), 2.00 (h, J = 6.8 Hz, 1H$_{\{25\}}$), 1.68 – 0.90 (overlapped signals : m, 6H$_{\{44, 46, 49\}}$, d, J = 6.8 Hz, 3H$_{\{39\}}$, 3broads s, 9H$_{\{43, 47, 48\}}$), 0.84 (d, J = 6.7 Hz, 3H$_{\{26\}}$), 0.81 (d, J = 6.8 Hz, 3H$_{\{27\}}$), 0.62 (s, 3H$_{\{45\}}$). ^{13}C NMR (101 MHz, MeOD-$CDCl_3$ (1 : 1 ; v : v)) δ 175.7 (C$_{\{54\}}$), 174.0 (C$_{\{50\}}$), 173.0 (C$_{\{10\}}$), 172.2 (C$_{\{21\}}$), 170.2 (C$_{\{28\}}$), 142.3 (C$_{\{3\}}$), 137.3 (C$_{\{33\}}$), 137.2 (C$_{\{15\}}$), 136.9 (C$_{\{6\}}$), 129.5 (2CH$_{\{35, 37\}}$), 129.5 (2CH$_{\{17, 19\}}$), 129.0 (4CH$_{\{16, 20, 34, 38\}}$), 127.4 (2CH$_{\{1, 5\}}$), 127.3 (CH$_{\{36\}}$), 127.2 (CH$_{\{18\}}$), 120.5 (2CH$_{\{2, 4\}}$), 83.2 (CH$_{\{7\}}$), 60.4 (2C$_{\{41, 42\}}$), 60.3 (CH$_{\{22\}}$), 55.7 (CH$_{\{12\}}$), 55.4 (CH$_{\{29\}}$), 40.6 (2CH$_{2\{44, 46\}}$), 38.2 (CH$_{2\{14\}}$), 37.7 (CH$_{2\{32\}}$), 34.3 (2CH$_{3\{43, 45\}}$), 31.0 (CH$_{\{53\}}$), 30.7 (CH$_{2\{25\}}$), 29.8 (CH$_{2\{51\}}$), 23.7 (CH$_{3\{39\}}$), 20.6 (2CH$_{3\{47, 48\}}$), 19.2 (CH$_{3\{26\}}$), 18.3 (CH$_{3\{27\}}$), 17.5 (CH$_{2\{49\}}$). HRMS (ESI) : m/z calc for $C_{44}H_{60}N_5O_7^+$: 770.4487 [M+H]$^+$; found : 770.4481.

tert-butyl ((2R)-1-oxo-3-phenyl-1-((4-(1-((2,2,6,6-tetramethylpiperidin-1-yl)oxy)ethyl)phenyl)amino)propan-2-yl)carbamate **A1D**.. ^{1}H NMR (400 MHz, $CDCl_3$) δ 8.22 (s, 1H$_{\{9\}}$), 7.33 (Broad A*B*, d, J = 8.3 Hz, 2H$_{\{2, 4\}}$), 7.29 – 7.19 (m, 7H$_{\{1, 5, 15\text{-}19\}}$), 5.44 (m, 1H$_{\{20\}}$), 4.73 (q, J = 6.6 Hz, 1H$_{\{7\}}$), 4.53 (s, 1H$_{\{12\}}$), 3.21 – 3.01 (m, 2H$_{\{13\}}$), 1.59 – 1.34 (overlapped signals : m, 6H$_{\{32, 35, 38\}}$, d, J = 6.6 Hz, 3H$_{\{28\}}$, s, 9H$_{\{25\text{-}27\}}$), 1.29 (s, 3H$_{\{33\}}$), 1.15 (s, 2H$_{\{36\}}$), 1.03 (s, 3H$_{\{34\}}$), 0.65 (s, 3H$_{\{37\}}$). ^{13}C NMR (101 MHz, $CDCl_3$) δ 169.9 (C$_{\{10\}}$), 155.9 (C$_{\{21\}}$), 142.1 (C$_{\{3\}}$), 136.9 (C$_{\{14\}}$), 136.1 (C$_{\{6\}}$), 129.4 (2CH$_{\{16, 18\}}$), 128.8 (2CH$_{\{1, 5\}}$), 127.2 (2CH$_{\{15, 19\}}$), 127.0 (CH$_{\{17\}}$), 119.9 (2CH$_{\{2, 4\}}$), 82.7 (CH$_{\{7\}}$), 80.5 (C$_{\{24\}}$), 59.8 (C$_{\{30, 31\}}$), 56.8 (CH$_{\{12\}}$), 40.4 (2CH$_{2\{32, 35\}}$), 38.7 (CH$_{2\{13\}}$), 34.4 (2CH$_{3\{33, 36\}}$), 28.4 (3CH$_{3\{25\text{-}27\}}$), 23.5 (CH$_{3\{28\}}$), 20.4 (2CH$_{3\{34,37\}}$), 17.3 (CH$_{2\{38\}}$). Triplet at 127,9 ppm is solvent C_6D_6. HRMS (ESI) : m/z calc for $C_{31}H_{46}N_3O_4^+$: 524.3483 [M+H]$^+$; found: 524.3481.

(2R)-1-oxo-3-phenyl-1-((4-(1-((2,2,6,6-tetramethylpiperidin-1-yl)oxy)ethyl)phenyl)amino)propan-2-aminium trifluoroacetate **A2D**. By usingGP3 with **A1D** (1.50 g, 2.866 mmol, 1.0 eq) afforded **A2D** as a white solid (1.33 g, yield: 86 %). The product was used in coupling reaction without characterization.

tert-butyl ((2R)-1-(((2R)-3-methyl-1-oxo-1-(((2R)-1-oxo-3-phenyl-1-((4-(1-((2,2,6,6-tetramethylpiperidin-1-yl)oxy)ethyl)phenyl)amino)propan-2-yl)amino)butan-2-yl)amino)-1-oxo-3-phenylpropan-2-yl)carbamate **A3D**. ^{1}H NMR (400 MHz, MeOD) δ 7.42 (Broad, *A*B, d, J = 8.6 Hz, 2H$_{\{2, 4\}}$), 7.30 – 7.12 (m, 12H$_{\{1, 5, 16\text{-}20, 41\text{-}45\}}$), 4.80 (overlapped signal : water and q, 1H$_{\{7\}}$), 4.69 (t, J = 7.5 Hz, 1H$_{\{12\}}$), 4.42 (dd, J = 10.0, 4.6 Hz, 1H$_{\{29\}}$), 4.25 (d, J = 7.0 Hz, 1H$_{\{22\}}$), 3.18 (dd, J = 13.7, 7.2 Hz, 1H$_{\{14\}}$), 3.06 (dd, J = 13.9, 4.9 Hz, 1H$_{\{32\}}$), 2.99 (dd, J = 13.6, 7.9 Hz, 1H$_{\{14\}}$), 2.79 (dd, J = 14.2, 10.0 Hz, 1H$_{\{32\}}$), 2.02 (dq, J = 6.8 Hz, 1H$_{\{25\}}$), 1.51 – 1.00 (overlapped signals : m, 6H$_{\{51, 53, 56\}}$, d, J = 6.7 Hz, 3H$_{\{46\}}$, s, 9H$_{\{38\text{-}40\}}$ and s, 3H$_{\{52\}}$, s, 3H$_{\{50\}}$, s, 3H$_{\{54\}}$), 0.89 – 0.81 (overlapped signals : 2d, J = 6.9 Hz, 6H$_{\{26, 27\}}$), 0.61 (s, 3H$_{\{55\}}$). ^{13}C NMR (101 MHz, $CDCl_3$) δ 172.4 (C$_{\{10\}}$), 171.9 (C$_{\{21\}}$), 169.7 (C$_{\{28\}}$), 156.0 (C$_{\{34\}}$), 142.0 (C$_{\{3\}}$), 137.0 (C$_{\{33\}}$), 136.6 (C$_{\{15\}}$), 136.5 (C$_{\{6\}}$), 129.5 (2CH$_{\{42, 44\}}$), 129.3 (2CH$_{\{17, 19\}}$), 128.6 (2CH$_{\{41, 45\}}$), 128.5 (2CH$_{\{16, 20\}}$), 127.1 (CH$_{\{43\}}$), 127.0 (CH$_{\{18\}}$), 126.9 (CH$_{\{1\}}$), 126.8 (CH$_{\{5\}}$), 120.4 (2CH$_{\{2, 4\}}$), 82.8 (CH$_{\{7\}}$), 80.0 (C$_{\{37\}}$), 59.8 (2C$_{\{48, 49\}}$), 58.5 (CH$_{\{22\}}$), 55.7 (CH$_{\{12\}}$), 55.4 (CH$_{\{29\}}$), 40.5 (2CH$_{2\{51, 53\}}$), 39.3 (CH$_{2\{32\}}$), 38.5 (CH$_{2\{14\}}$), 34.4 (2CH$_{3\{50, 52\}}$), 31.6 (CH$_{\{25\}}$), 28.5 (3CH$_{3\{38\text{-}40\}}$), 23.5 (CH$_{3\{46\}}$), 20.4 (2CH$_{3\{54, 55\}}$), 19.3 (CH$_{\{26\}}$), 18.1(CH$_{\{27\}}$), 17.3 (CH$_{2\{56\}}$). HRMS (ESI) : m/z calc for $C_{45}H_{64}N_5O_6^+$: 770.4851 [M+H]$^+$; found : 770.4854

(2R)-1-(((2R)-3-methyl-1-oxo-1-(((2R)-1-oxo-3-phenyl-1-((4-(1-((2,2,6,6-tetramethylpiperidin-1-yl)oxy)ethyl)phenyl)amino)propan-2-yl)amino)butan-2-yl)amino)-1-oxo-3-phenylpropan-2-aminium trifluoroacetate **A4D**. ^{1}H NMR (400 MHz, MeOD) δ 7.48 – 7.41 (m, 2H$_{\{2, 4\}}$), 7.34 – 7.14 (m, 12H$_{\{1, 5, 16\text{-}20, 34\text{-}38\}}$), 4.99 – 4.89 (m, 1H$_{\{7\}}$), 4.74 (dd, J = 7.9, 7.0 Hz, 1H$_{\{112\}}$), 4.25 (d, J = 7.5 Hz, 1H$_{\{22\}}$), 4.16 (dd, J = 8.5, 5.4 Hz, 1H$_{\{29\}}$), 3.18 (ddd, J = 14.5, 6.2, 4.2 Hz, 2H$_{\{32\}}$), 3.04 (dd, J = 13.7, 7.9 Hz, 1H$_{\{14\}}$), 2.95 (dd, J = 14.3, 8.6 Hz, 1H$_{\{14\}}$), 2.05 (dq, J = 13.1, 6.5 Hz, 1H$_{\{25\}}$), 1.77 – 1.45 (overlapped signals : m, 6H$_{\{44, 46, 49\}}$, d, J = 6.6 Hz, 3H$_{\{39\}}$), 1.38 (s, 3H$_{\{43\}}$), 1.29 (s, 3H$_{\{45\}}$), 1.13 (s, 3H$_{\{47\}}$), 0.98 – 0.92 (overlapped signals : d, J = 6.7 Hz, 3H$_{\{27\}}$, d, J = 6.7 Hz, 3H$_{\{26\}}$), 0.71 (s, 3H$_{\{48\}}$). ^{13}C NMR (75 MHz, MeOD) δ 172.8 (C$_{\{10\}}$), 171.5 (C$_{\{28\}}$), 169.7 (C$_{\{21\}}$), 141.3 (C$_{\{3\}}$), 138.6 (C$_{\{33\}}$), 138.0 (C$_{\{15\}}$), 135.3 (C$_{\{6\}}$), 130.5 (2CH$_{\{35, 37\}}$), 130.4 (2CH$_{\{17, 19\}}$), 130.0 (2CH$_{\{34, 38\}}$), 129.5 (2CH$_{\{16, 20\}}$), 128.7 (CH$_{\{36\}}$), 128.2 (2CH$_{\{1, 5\}}$), 127.8 (CH$_{\{18\}}$), 121.2 (2CH$_{\{2, 4\}}$), 85.1 (CH$_{\{7\}}$), 64.2 (2C$_{\{41, 42\}}$), 60.4 (CH$_{\{22\}}$), 56.8

(CH{29}), 55.3 (CH{12}), 40.3 ($2CH_{2}${44, 46}), 39.7 (CH_{2}{32}), 38.6 (CH_{2}{14}), 33.1 ($2CH_{3}${43, 45}), 32.1 (CH{25}), 23.6 (CH_{3}{39}), 20.8 ($2CH_{3}${47, 48}), 19.7 (CH_{3}{27}), 18.9 (CH_{3}{26}), 17.5 (CH_{2}{49}). ^{19}F NMR (376 MHz, MeOD) δ -76.96. HRMS (ESI) : m/z calc for cation $C_{40}H_{56}N_{5}O_{4}^{+}$: 670.4327 ; found : 670.4330. m/z calc for anion $C_{2}F_{3}O_{2}^{-}$: 112.9856 ; found : 112.9856.

(2R)-2-((R)-2-amino-3-phenylpropanamido)-3-methyl-N-((2R)-1-oxo-3-phenyl-1-((4-(1-((2,2,6,6-tetramethylpiperidin-1-yl)oxy)ethyl)phenyl)amino)propan-2-yl)butanamide **A5D**. ^{1}H NMR (400 MHz, MeOD) δ 7.43 (broad *A*B, d, J = 8.5 Hz, 2H{2, 4}), 7.32 – 7.14 (m, 12H{1, 5, 16-20, 34-38}), 4.78 – 4.68 (overlapped signals : m, 2H{12, 7}), 4.16 (d, J = 7.1 Hz, 1H{22}), 3.66 (dd, J = 8.0, 5.1 Hz, 1H{29}), 3.20 (dd, J = 13.7, 6.8 Hz, 1H{14}), 3.09 – 2.94 (m, 2H{32}), 2.77 (dd, J = 13.6, 8.1 Hz, 1H{14}), 2.00 (h, J = 6.8 Hz, 1H{25}), 1.62 – 0.93 (overlapped signals : m 6H{44, 46, 49}, d, J = 6.6 Hz, 3H{{39}, three Broad s, 9H{43, 47, 48}), 0.85 (overlapped signals : 2d, J = 6.8 Hz, 6H{26, 27}), 0.62 (s, 3H{45}). ^{13}C NMR (101 MHz, MeOD) δ 176.7 (C{28}), 173.3 (C{10}), 171.4 (C{21}), 143.0 (C{3}), 138.7 (C{33}), 138.2 (C{15}), 138.1 (C{6}), 130.5 (2CH{35, 37}), 130.4 (2CH{17, 19}), 129.6 (2CH{34, 38}), 129.5 (2CH{16, 20}), 128.1 (2CH{1, 5}), 127.8 (CH{36}), 127.8 (CH{18}), 121.2 (2CH{2, 4}), 84.0 (CH{7}), 60.9 (2C{41, 42}), 60.0 (CH{22}), 57.3 (CH{29}), 56.7 (CH{12}), 42.1 (CH_{2}{32}), 41.4 ($2CH_{2}${44, 46}), 39.1 (CH_{2}{14}), 34.8 ($2CH_{3}${43, 45}), 32.1 (CH{25}), 23.7 (CH_{3}{39}), 20.8 ($2CH_{3}${47, 48}), 19.7 (CH_{3}{26}), 18.7 (CH_{3}{27}), 18.1 (CH_{2}{49}). HRMS (ESI) : m/z calc for $C_{40}H_{56}N_{5}O_{4}^{+}$: 670.4327 $[M+H]^{+}$; found : 670.4330.

*4-(Benzyloxy)-4-oxobutanoic acid***.** ^{1}H NMR (400 MHz, MeOD) δ 7.51 – 7.15 (m, 5H{3-7}), 5.13 (s, 2H{1}), 2.75 – 2.46 (m, 4H{10, 12}).
